# An interpretable learning framework for exploring superelastic degradation of NiTi shape memory alloys using multimodal data

**DOI:** 10.1038/s41467-026-77250-4

**Published:** 2026-08-31

**Authors:** Yuejie Hu, Haiyang Wang, Ming Chen, Gang Chen, Chuanjie Wang, Bin Guo, M. W. Fu

**Affiliations:** 1 School of Materials Science and Engineering, Harbin Institute of Technology at Weihai, Weihai, China; 2 State Key Laboratory of Metal Matrix Composites, Shanghai Jiao Tong University, Shanghai, China; 3 School of Materials Science and Engineering, Shanghai Jiao Tong University, Shanghai, China; 4 Key Laboratory of Micro-Systems and Micro-Structures Manufacturing (Harbin Institute of Technology), Ministry of Education, Harbin, China; 5 Department of Mechanical Engineering, The Hong Kong Polytechnic University, Hung Hom, Kowloon, Hong Kong, China

**Keywords:** Computational methods, Metals and alloys

## Abstract

Superelastic degradation (SED), a progressive loss of functionality in NiTi shape memory alloys under cyclic loading, remains challenging to characterize precisely, thereby constraining their engineering applications and broader adoption. In this study, an interpretable learning framework was proposed to predict the SED of NiTi alloys and uncover the degradation mechanisms using interpretability analysis methods. The framework incorporates multi-source microstructure and loading conditions through a multi-branch architecture that effectively decouples and integrates heterogeneous features, achieving an *R*^2^ of 0.981. The competition between slip and transformation was identified: at high amplitudes, SED is dominated by transformation regions with high Schmid factors, whereas at low amplitudes, dislocation slip on the {011}〈001〉 and {011}〈111〉 systems prevails. Subsequently, the influence of Ni_4_Ti_3_ precipitates was quantified, revealing a loading-dependent and non-uniformly beneficial role. The results highlight the potential of interpretable machine learning in exploring the cyclic deformation process and pave the way for AI-driven research on smart materials.

## Abstract (web summary)

This manuscript highlights an interpretable learning framework to explore superelastic degradation in NiTi alloys, revealing mechanisms linked to grain orientation and Ni4Ti3 precipitates, and promoting data-driven design of shape memory alloys.

## Introduction

Superelasticity, the hallmark functional response of shape memory alloys (SMAs), originates from the highly reversible stress-induced martensitic transformation (SIMT). Under cyclic loading, however, this reversibility progressively weakens, manifested by residual strain accumulation, narrowing transformation hysteresis, and the diminishing transformation driving force^1,2^. Such degradation occurs during both early conditioning and prolonged service and, in either case, proceeds in an intractable and uncontrollable manner. Consequently, a robust physical understanding and precise characterization of superelastic degradation (SED) remain central challenges for the further engineering implementation of SMAs.

Conventional accounts ascribe SED to dislocation accumulation and irreversible transformation^3–8^. Nonetheless, their relative contributions and the specific degradation mechanisms remain poorly resolved. The activation of slip and transformation is governed by the initial microstructures, in particular, grain size, internal stress state, and precipitate characteristics. In practice, a nominally single microstructural variation is rarely observed in isolation but is accompanied by the concerted evolution of other attributes. For instance, precipitation induces adjustments in matrix composition, transformation temperature, and stress plateau^9^. Moreover, such changes are intricately associated with the loading conditions, and the interplay thereby alters the dominant deformation pathway^10^. The multifactor coupling severely complicates the separation of individual variables, hence restricting the depth of quantitative mechanistic insights in previous studies.

To describe cyclic stress-strain behavior, phenomenological or semi-empirical models based on parameterized constitutive equations have been widely adopted^11–14^. These approaches are typically constructed within the framework of continuum mechanics, building upon predefined physical assumptions and requiring extensive experimental calibration and parameter optimization. However, as noted above, variable control in these studies is constrained. More importantly, parameter fitting often requires manual feature extraction or spatial averaging of microstructural images^15^ to obtain usable inputs. While this digitization simplifies the modeling process, it frequently introduces subjective bias and compromises the integrity of spatial structural information.

Multimodal machine learning (ML) represents a new paradigm for linking microstructure and properties in materials science, with feature representations directly extracted from images and remarkable performance in predicting static properties (yield strength, tensile strength, etc.)^16–18^. Recent works have also demonstrated the potential of interpretative techniques in mechanistic exploration^19,20^. Despite the achievements, current frameworks still lack systematic methods for fusing heterogeneous information, and redundancy and scale mismatch between modalities often undermine their ability to capture complex correlations. Notably, most prior efforts have focused on microstructure and composition^21^, whereas external factors, particularly loading, have been largely overlooked, even though SED in SMAs is essentially a manifestation of functional degradation under cyclic loading. Overall, existing multimodal research on materials leaves considerable room for improvement in architectural design and interpretability.

**Fig. 1:**
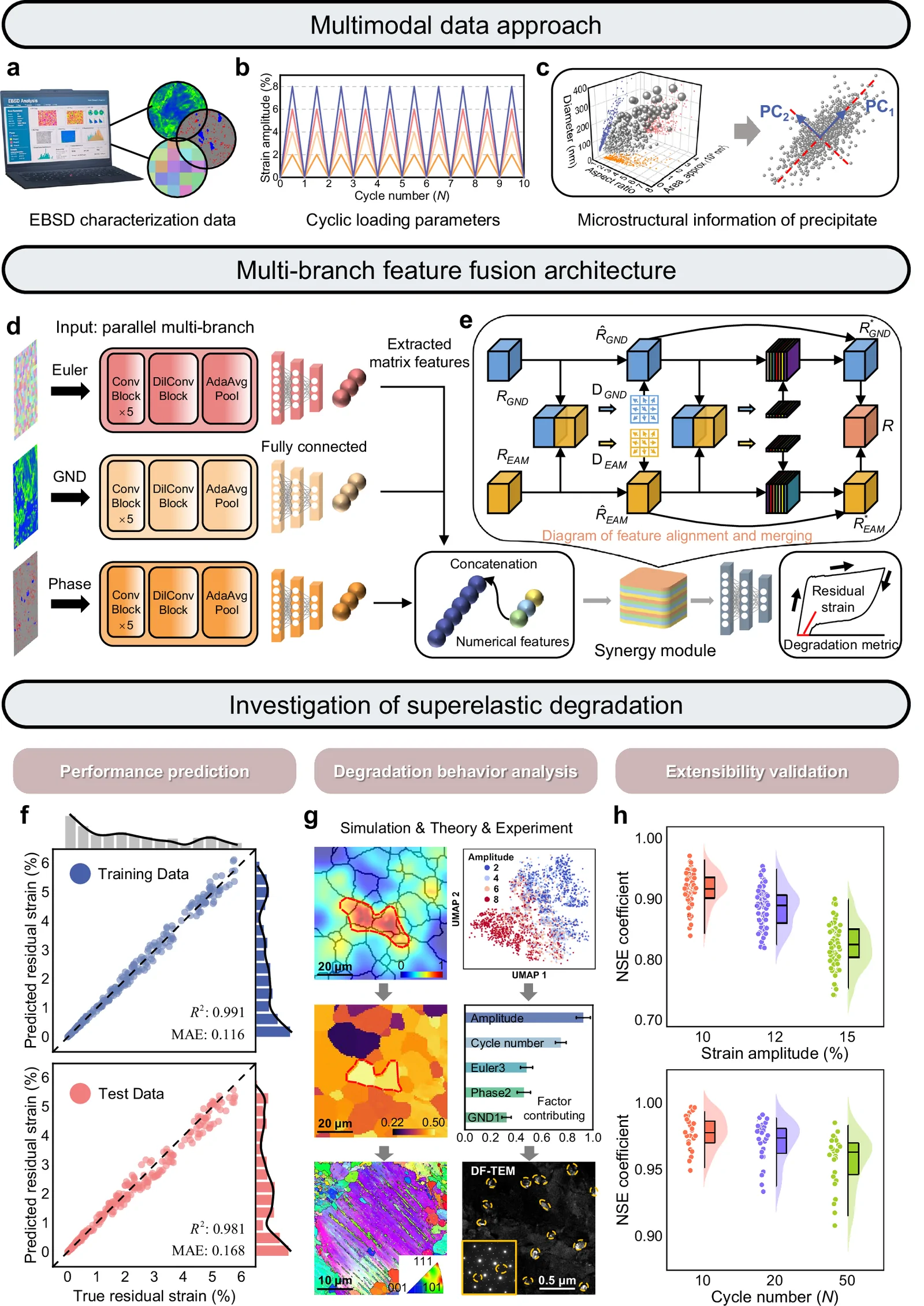
Schematic overview of the study. The workflow comprises three stages: data construction (**a**–**c**), architecture design (**d**, **e**), and degradation analysis and validation (**f**–**h**). The multimodal dataset integrates EBSD microstructural information (**a**), loading parameters (**b**), and statistics of nanoscale precipitates (**c**). **d** To characterize the relationships among heterogeneous inputs, a multi-branch feature fusion architecture was developed. Structural features, including Euler matrices, phase distributions, and GND maps, are extracted through independent channels and concatenated with numerical variables. **e** The feature synergy module aligns modality-specific representations and merges complementary information. **f** Predictive performance is illustrated by the correlation between experimental and predicted residual strains, with marginal histograms showing data distributions. This approach enables accurate prediction of superelastic degradation with an *R*^2^ of 0.981. **g** When coupled with manifold learning, feature attribution methods, and molecular dynamics simulations, it elucidates the roles of grain orientation and precipitates in the degradation process. **h** Extensibility validation encompasses strain amplitude and cycle number extrapolations, alongside generalization assessments under diverse loading conditions (e.g., strain rate and temperature), further demonstrating the practical relevance of the framework.

This work presents an interpretable multimodal learning framework for exploring the SED of NiTi alloys. When building the relationship among loading, microstructure, and properties, we wanted to ensure the solution was comprehensive, simple, interpretable, and extensible. More specifically, the framework incorporates EBSD data, including Euler orientation matrices, phase maps, and geometrically necessary dislocation (GND) density maps, together with loading parameters and statistics on Ni_4_Ti_3_ nanoprecipitates (Fig. 1a–c). A customized multi-branch feature fusion architecture (MBFFA) enables deep extraction and synergy of cross-modal features, thereby fully exploiting heterogeneous information (Fig. 1d, e). On this basis, primary mechanisms associated with microstructural features can be identified and quantified while controlling for relevant variables. We visualize the competition between slip and SIMT across different strain amplitudes and cyclic stages, and further clarify the dual role of Ni_4_Ti_3_ precipitates in either promoting or suppressing SED. Finally, a model is extensible if it can be effectively adapted to related scenarios (Fig. 1h). Our study delineates a promising avenue for AI-driven assessment and interpretation of functional stability in SMAs, and orients future explorations in this emerging direction of materials research.

## Results

### Dataset generation

A multimodal dataset was constructed to predict the SED of NiTi under cyclic tensile loading-unloading at room temperature. The material used was a 500 μm thick commercial NiTi sheet exhibiting a B2 austenitic phase at ambient temperature. Six initial microstructures were prepared by short-time annealing at 500, 600, and 700 °C for 10 or 30 min. At 500 °C, fine Ni_4_Ti_3_ precipitates were predominant; at 600 °C, Ni_4_Ti_3_ and NiTi_2_ coexisted in a multiphase structure; and at 700 °C, NiTi_2_ became the prevailing phase (Supplementary Fig. 15). The annealing duration mainly influenced the size and spatial distribution of precipitates.

Each sequence contained three aligned EBSD mappings: (1) Euler matrix: The Euler angles (*Φ*_1_, *Φ*, *Φ*_2_) of each pixel were encoded into a three-channel *H* × *W* × 3 orientation field. Unlike inverse pole figure maps, which are limited by color mapping, this representation preserves the complete crystallographic orientation. Although Euler angles may exhibit intrinsic redundancy and discontinuities, they are treated here as spatially structured fields. By employing local convolutions, the model primarily captures orientation gradients and their spatial correlations, thereby mitigating the impact of such discontinuities on feature learning. (2) Phase map: Distinguishes the matrix from precipitates. (3) GND map: Quantifies the spatial distribution of geometrically necessary dislocation density, reflecting local lattice curvature and crystallographic heterogeneity within the initial microstructure.

Loading parameters included cycle number and strain amplitude. The precipitation state was represented by a single numerical descriptor using principal component analysis (PCA)^22^ of Ni_4_Ti_3_ precipitate statistics obtained from transmission electron microscopy. The first principal component, which captures 83.45% of the standardized variance, was used as the precipitate descriptor in the numerical branch. The target label was the residual strain measured at the end of each cycle, which was adopted as the degradation metric because it provides a direct and consistently extractable measure of the accumulated irreversibility during cyclic SED.

### Model evaluation

**Fig. 2:**
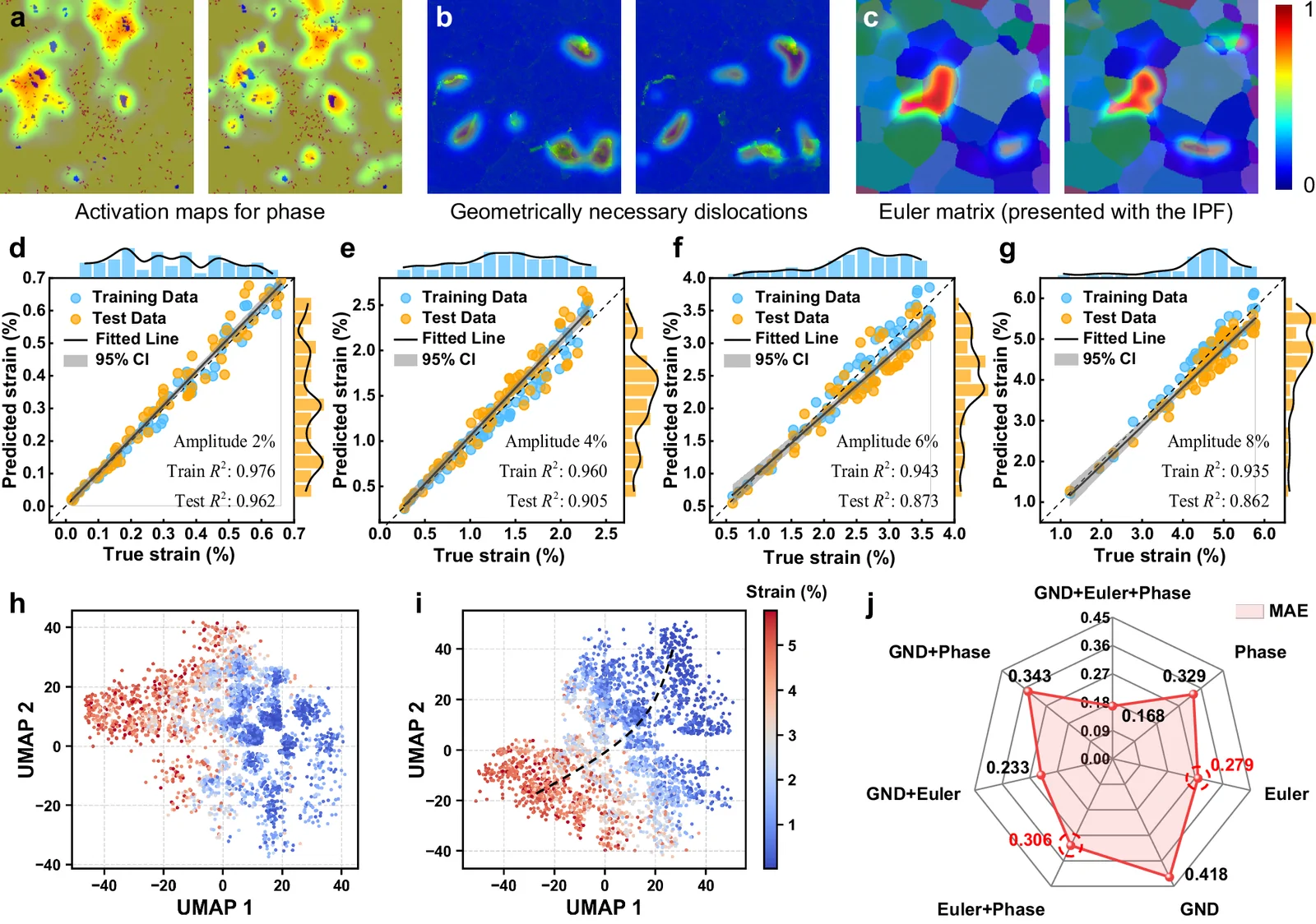
Structural evaluation, predictive performance and interpretability of MBFFA. Grad-CAM visualizations comparing feature extraction strategies between MBFFA (left) and the channel stacking approach^16^ (right) across (**a**) phase images, (**b**) GND maps, and (**c**) Euler angle matrices. Activation intensities are normalized between 0 and 1 for each map, where higher intensities denote greater predictive contributions. MBFFA exhibits superior localization on physically meaningful features, such as phase boundaries, localized lattice curvature, and orientation discontinuities. **d**–**g** Predicted versus experimental residual strain under cyclic strain amplitudes of 2%, 4%, 6% and 8%, respectively. Points indicate averaged training predictions and held-out test predictions from grouped tenfold cross-validation over the 240 independent labels. Dashed diagonals represent ideal predictions; top and right marginal plots display the distributions of true and predicted values. **h**, **i** UMAP projections of high-dimensional latent feature vectors from the 3360 original, non-augmented training samples (**h**) before and (**i**) after the synergy module, colored by experimental residual strain. After fusion, the latent space exhibits a more orderly structure with distinct continuous strain gradients. **j** MAE radar plot comparing different EBSD input combinations. A lower MAE indicates better predictive accuracy, highlighting the advantage of multi-source inputs and underscoring the necessity of a targeted feature fusion architecture.

The modeling strategy draws inspiration from the concept of feature decoupling in DL. In object detection tasks, researchers often handle classification and localization features separately through a dual stream architecture to reduce feature entanglement and enhance recognition accuracy^23–25^. This design is analogous to the dual-pathway processing in the human visual system (Supplementary Fig. 27), which remains abstract for materials research. To address this, we employ Grad-CAM visualization^26^ (Fig. 2a–c), which reveals that the MBFFA produces concentrated and convergent responses in regions of phase boundaries, lattice distortion, and orientation discontinuities, whereas the channel stacking approach^16^ yields dispersed and offset hotspot distributions. Phase, GND maps, and Euler matrices are regarded as complementary channels with distinct physical interpretations. Independent characterization followed by integration avoids semantic interference caused by simple superposition.

At low strain amplitudes of 2% and 4%, the data exhibit greater dispersion, with narrower ranges of prediction residuals. The training *R*^2^ values 0.976 and 0.960, while the corresponding test *R*^2^ values are 0.962 and 0.905, respectively. The regression line lies slightly above the reference, indicating a mild positive bias. As amplitudes increase to 6% and 8%, the data gradually concentrate in regions of higher residual strain, resulting in a tighter distribution. The training *R*^2^ decrease slightly to 0.943 and 0.935, and the test *R*^2^ decrease to 0.873 and 0.862, respectively. The regression line shifts downward, revealing a negative bias. Across all cases, outliers remain below 1.2%, thus demonstrating stable predictions with low overall error. UMAP visualizations (Fig. 2h, i and Supplementary Fig. 40) show that the feature synergy module produces a more organized latent space with a distinct continuous gradient of true residual strain. Combined with the cosine similarity analysis, this indicates that the module effectively facilitates cross-modal representation alignment and promotes a more coherent encoding of deformation-related information.

Input configurations from EBSD sources were evaluated. Among single-source settings, the Euler matrix performed best (MAE = 0.279), outperforming methods using only the phase image (MAE = 0.329) or the GND image (MAE = 0.418). Dual inputs delivered significant improvements: MAE values of 0.343, 0.306, and 0.233 for GND + phase, Euler + phase, and Euler + GND, respectively. Incorporating three modalities also improved effectiveness. Nevertheless, combined inputs did not consistently outperform a single source, regardless of information type. For example, compared with the Euler matrix alone, adding the phase input increased MAE (from 0.279 to 0.306), suggesting that an additional channel can introduce redundancy or noise. The result underscores the necessity of rational feature decoupling and integration under multi-source data scenarios.

### Fusion strategy assessment

When comparing fusion strategies, direct multi-channel concatenation was first considered, which is adopted by most existing methods (MCC-Net)^27^. Alternatively, early cross-modal matrix fusion incorporates numerical feature matrices as inputs and jointly encodes them with images (ECMMF-Net)^28^. This method, however, captures interaction insufficiently and performs poorly on this task. To clarify the independent contribution of the synergy module, an ablation version (Base-w/oSM) was developed. In addition, the multi-branch network was preserved, and numerical information was injected into each branch (MBNI-Net).

**Fig. 3:**
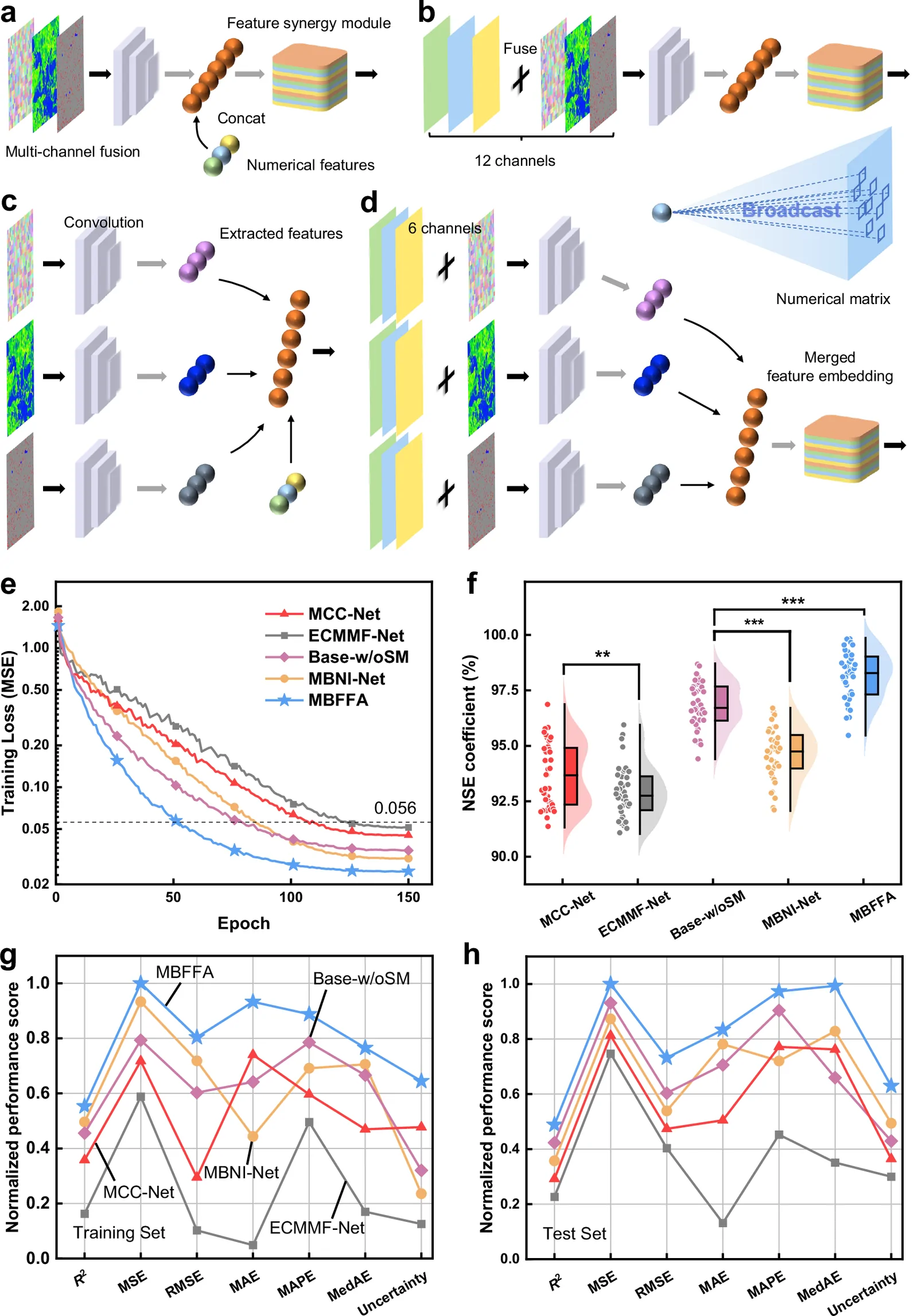
Design and comparison of feature fusion strategies. **a** MCC-Net applies direct concatenation of channel features. **b** ECMMF-Net incorporates the matrix representations of the numerical features at the early stage. **c** Base-w/oSM serves as an ablation version to assess the effect of the synergy module. **d** MBNI-Net embeds numerical information within each branch to strengthen interactions among modalities. **e** Training mean squared error (MSE) loss over 150 epochs for the five fusion strategies. MBFFA converges fastest during training and reaches the lowest final loss of 0.0249. **f** Distribution of test NSE coefficients across 40 loading conditions for each model. Violin plots depict kernel density estimates, with internal box plots marking the median and interquartile range. Statistical significance was assessed using a two-sided unpaired Student’s *t*-test, with exact *P* values indicated in the graph (**p < 0.01, ***p < 0.001). MBFFA exhibits an upward-shifted and tightly clustered distribution. **g**, **h** Normalized performance scores of different models on the training (**g**) and test (**h**) sets. The proposed multimodal feature fusion strategy maintains superior performance across diverse metrics.

The losses of all models dropped rapidly within the first 40 epochs and then stabilized without obvious oscillations or overfitting observed (Fig. 3e and Supplementary Figs. 30, 31), suggesting that the mapping in this task is relatively simple and learnable. MBFFA converged fastest and remained fully stable by the end of training, achieving the lowest final loss (0.0249) among all models. Prediction stability shows a similar advantage (Fig. 3f). The Nash–Sutcliffe efficiency (NSE) distribution of MBFFA shifts upward, with a median of about 0.983 and a compact spread with negligible tails, whereas the other models cluster between 0.911 and 0.987 and exhibit greater dispersion. Thus, synergistic fusion improves not only accuracy but also markedly reduces prediction variability across initial microstructures and loading conditions (two-sided *t*-statistic (78 degrees of freedom) = 5.57, *p* < 0.001, Cohen’s *d* = 1.24, 95% confidence interval for the difference of means = [0.0085, 0.0180]).

On the multi-dimensional curve, MBFFA exhibits well-balanced behavior with no apparent weaknesses (Fig. 3g, h, and Supplementary Table 12). In contrast, MCC-Net and ECMMF-Net without channel separation have relative drawbacks: the former yields MAE and uncertainty of 0.229 and 0.452, whereas the latter exhibits a high training loss (0.0514). Base-w/oSM approaches MBFFA on some metrics, but its MAE (0.205) and uncertainty (0.402) remain inferior. Taken together, simple feature concatenation and channel stacking struggle to reliably distinguish redundancy from complementarity across modalities. The decoupling-guided fusion strategy overcomes this limitation and enables more robust mapping under complex multimodal conditions.

### Interpretive analysis

Although the approach yields respectable accuracy in predicting SED, elucidating the underlying physical processes is still far from straightforward. To address this, we probed the governing factors by combining heatmap visualization and Shapley Additive Explanations (SHAP) analysis^29^ with molecular dynamics simulations. This study presents a spatially resolved and systematic analysis of how the dominant degradation mode evolves with strain amplitude under cyclic loading. Building on this, the influence of precipitates across different regimes is further clarified, thereby advancing the mechanistic understanding of SMA degradation.

### Crystal orientation

**Fig. 4:**
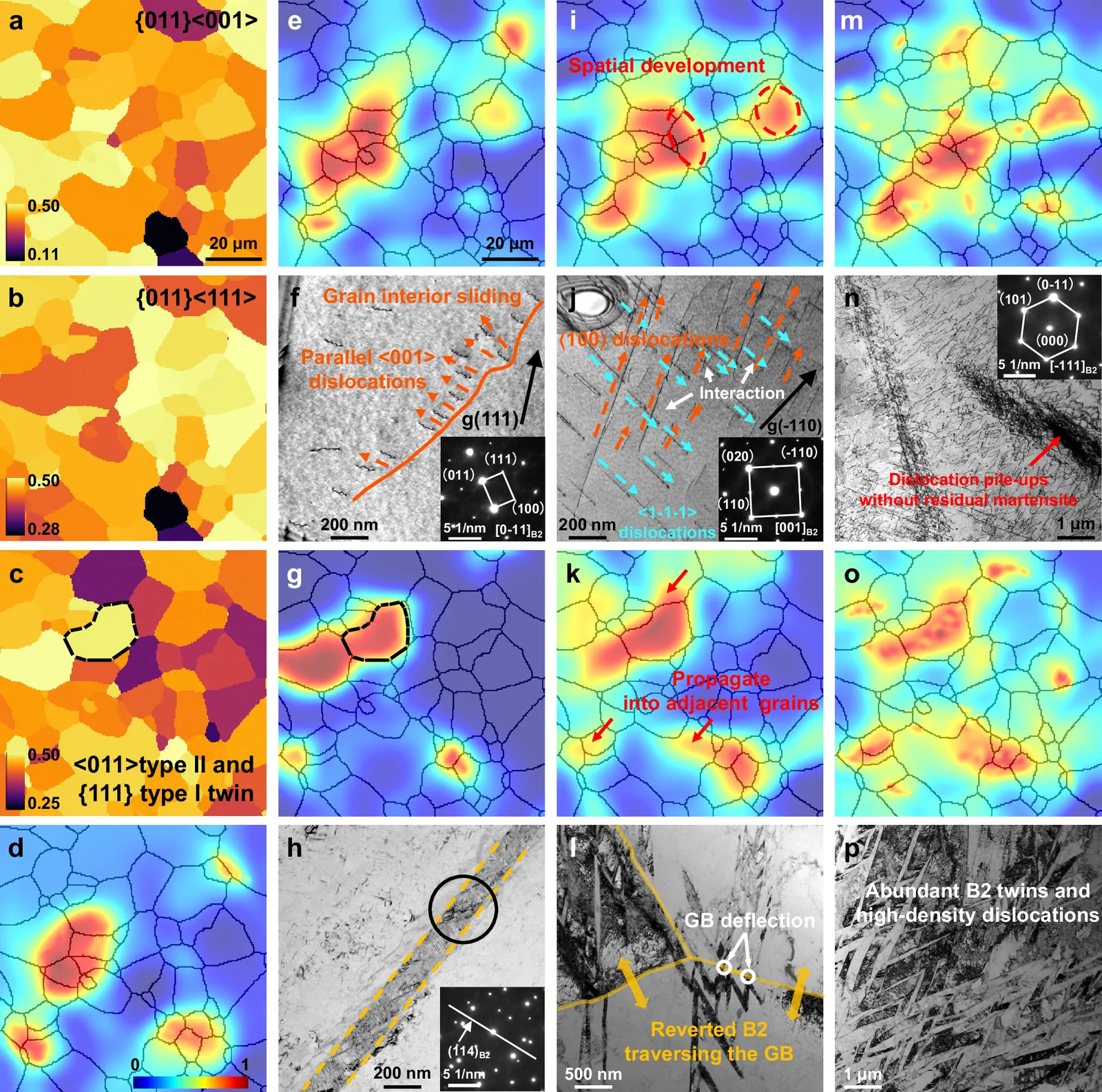
Visualization reveals the orientation evolution of dominant degradation modes under various loading conditions. **a**–**c** Schmid factor maps corresponding to the {011}〈001〉, {011}〈111〉 slip systems, and SIMT. **e**, **i**, **m** Grad-CAM heatmaps generated from the Euler matrix branch for the 2% strain amplitude after 1, 3 and 10 cycles, respectively, where hotspots indicate orientation regions contributing most to degradation prediction. Activation intensities are normalized between 0 and 1 for each map. Corresponding transmission electron microscopy observations are shown in (**f**), (**j**), and (**n**). Similarly, (**g**), (**k**), and (**o**), as well as (**h**), (**l**), and (**p**), present the respective results for the 8% strain amplitude. The transition of dominant degradation modes under specific loading conditions is revealed: at high strain amplitude, hotspots strongly coincide with high Schmid factor regions for SIMT, indicating transformation-driven degradation; while at low strain amplitude, {011}〈001〉 and {011}〈111〉 dislocation slip dominates. Furthermore, **d** is derived from the first cycle at 6.38% strain amplitude, representing a transitional state between the two competing mechanisms.

Grad-CAM was applied to the Euler matrix to generate response heatmaps, in which highlighted regions correspond to local orientations that contribute most to degradation prediction. For crystallographic interpretation, the heatmap is overlaid with the grain boundary map. The current view holds that SED is primarily driven by two irreversible processes: the continuous accumulation of dislocation slip^7^ and the formation of retained structures from SIMT^3,30,31^. To verify the activation orientations associated with hotspots, we constructed three Schmid factor maps (Fig. 4a–c) incorporating local stress-field corrections induced by Ni_4_Ti_3_ precipitates. Two slip systems, {011}〈001〉 and {011}〈111〉, together with SIMT, are considered. The transformation pathways involve 〈011〉 type II and {111} type I twinning^4,32^. The Schmid factor for each pixel was computed from the Euler angles.

During the first cycle at 2% (Fig. 4e), hotspots are concentrated within a few grains and coincide with regions of high Schmid factors for the {011}〈001〉 slip system. This suggests that at low strain amplitudes, orientations favorable for 〈001〉 dislocation slip are the primary source of early degradation. Extinction observations (Fig. 4f) reveal bundles of nearly parallel 〈001〉 dislocation bands within grains, confirming the predominance of a single slip system in the initial stage. By the third cycle (Fig. 4i), hotspot coverage develops markedly, with newly formed regions partially overlapping the high value zones of {011}〈111〉 slip, indicating activation of the 〈111〉 slip system. Within the same field of view, intersections between 〈001〉 and 〈111〉 Burgers vector dislocations are observed (Fig. 4j), demonstrating that both slip systems begin to cooperate in driving degradation. After the tenth cycle (Fig. 4m), hotspots are distributed almost uniformly across the microstructure. No residual martensite or austenitic twins are detected (Fig. 4n), while a large number of dislocations accumulate both within grains and along grain boundaries.

In contrast, at 8% amplitude, the hotspots of the first cycle (Fig. 4g) largely coincide with regions exhibiting a high Schmid factor for transformation, indicating SIMT-dominated deformation. Striped {114} B2 austenitic twins traverse the grain interior (Fig. 4h), consistent with the transformation-plastic coupling theory under high stress^33^: transformations between martensitic variants generate austenitic twins in the parent phase to accommodate recovery strain rather than achieving stability through retained martensite. This study does not discuss reverse transformation pathways but focuses on the orientation responses revealed by residual structures—specifically, those more prone to SIMT and thus governing degradation. By the third cycle (Fig. 4k), hotspots propagate into adjacent grains, accompanied by austenitic twins traversing across grain boundaries (Fig. 4l). This propagation shows no clear preference for grain volume fraction or orientation and instead appears random, with its driving origin discussed later. By the tenth cycle (Fig. 4o), hotspots are almost pervasive throughout the microstructure. The corresponding morphology (Fig. 4p) shows widespread B2 twins coexisting with a high density of dislocations, while no residual martensite is yet observed.

It is worth emphasizing that the heatmaps reveal the orientational cues to which the model is most sensitive under given loading conditions. They reflect the structural response that governs degradation at the current stage, rather than representing an exclusive determination. In systems where multiple mechanisms compete and coexist, this approach is particularly practical for tracking the transition of dominant modes during deformation.

### Assessment of CNN-based visualizations

**Fig. 5:**
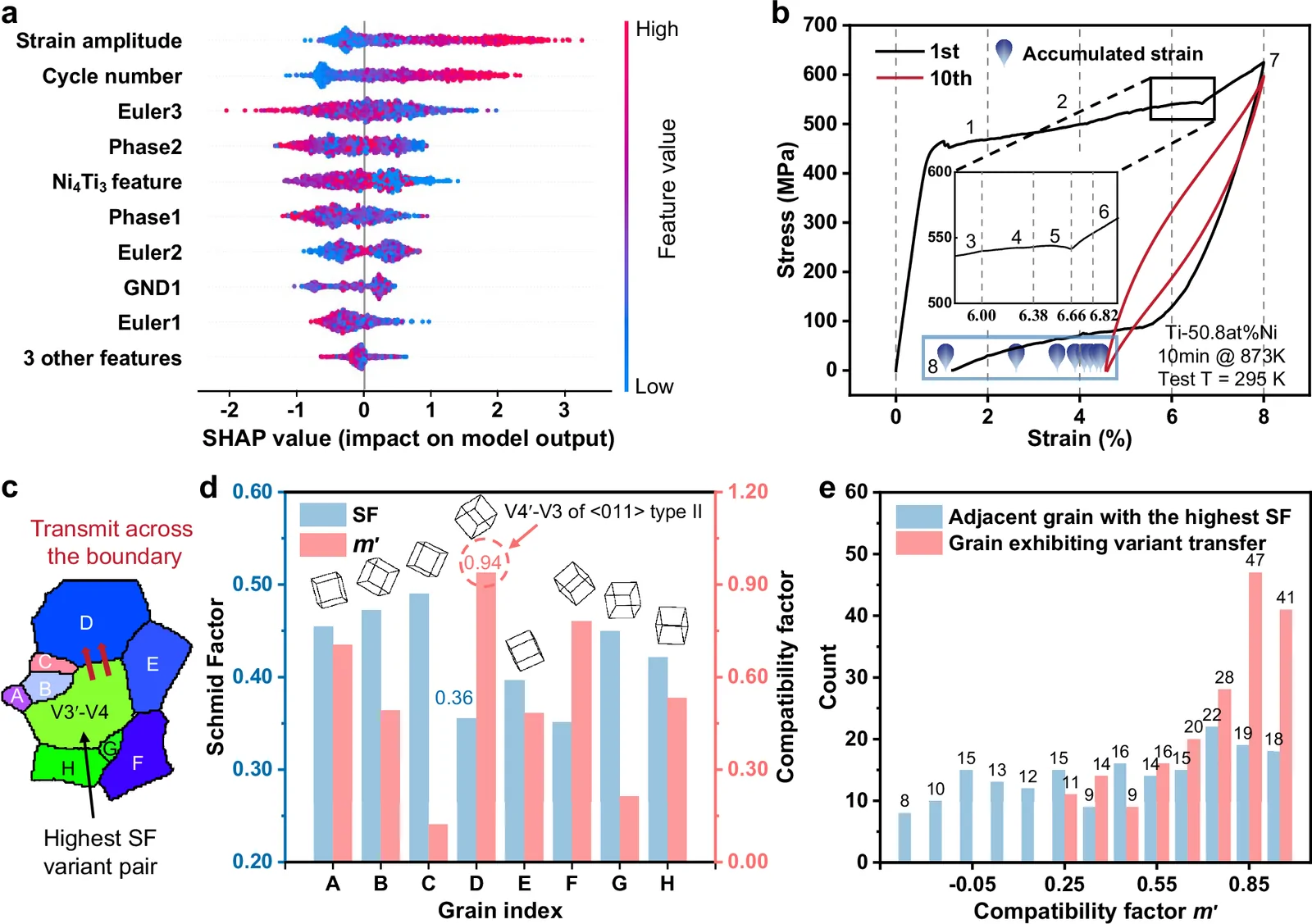
Quantitative statistics and geometric compatibility pathways of superelastic degradation. **a** SHAP summary plot ranking the contributions of loading and microstructural features. Strain amplitude is the dominant factor, followed by cycle number. **b** Representative stress-strain curves for the first and tenth cycles. Critical deformation stages within a single loading-unloading loop are indexed from 1 to 8, with indicators tracking the gradual accumulation of residual strain. **c** Hotspot grain selected from Fig. 4g and its eight adjacent grains A–H for variant transfer analysis. **d** Crystallographic metrics comparison, including Schmid factor and geometric compatibility factor *m*′ for the eight adjacent grains. Grain D exhibits the highest *m*′, with a pathway of V4′–V3 〈011〉 type II variant pair. Such alignment facilitates the propagation of transformation across grains. **e** Statistical validation across 186 sampled grains shows that neighboring grains exhibiting actual variant transfer exhibit consistently higher *m*′ than those with the maximum Schmid factor. These results indicate that the CNN can capture microscale strain gradients from Euler matrices to infer variant transfer directions.

Heatmaps primarily depict spatial distributions and are limited in quantifying the global impact of individual variables^34,35^. Thus, we introduced the SHAP method for attribution decomposition (Fig. 5a). The strain amplitude has the greatest influence, with SHAP values showing a clear upward trend as the amplitude increases. Cycle number ranks second, and its cumulative effect manifests as a gradual intensification of the degradation process. This quantitative characterization provides direct, measurable evidence that serves as a foundation for subsequent analysis.

Guided by this, we set the amplitude to 6.38% to capture the critical moment where slip and transformation contribute comparably (Fig. 4d), at which point transformation remains incomplete (Fig. 5b and Supplementary Fig. 13). This approach also enables a clear distinction of martensitic twin variants. For example, in the grain highlighted by the white dashed box in Fig. 4g, the highest Schmid factor corresponds to the twin pair V3′–V4. This grain, together with eight neighboring grains, was then selected and denoted A–H (excluding the adjacent hotspot grain on the left, Fig. 5c). Variant transmission is observed in grain D. When the transformation path follows V4′–V3 〈011〉 type II variant pair, the geometric compatibility factor *m*′ is highest with the original hotspot grain, rather than with grain B, which exhibits the most significant Schmid factor. Initially introduced to describe twin growth across grain boundaries in Mg alloys^36,37^ or Ti alloys^38^, *m*′ quantifies the alignment between the shear direction of variants and the normal to the habit plane in adjacent grains. A higher *m*′ represents less geometric mismatch and a more favorable condition for intergranular transmission.

Orientation-dependent propagation of the transformation zone appears repeatedly in the visualization results, rather than as an isolated case. Statistical analysis of 186 grains reveals that grains undergoing variant transfer exhibit significantly higher *m*′ with the original hotspot grain than neighboring grains with the highest Schmid factor (Fig. 5e). These findings indicate that the CNN captures implicit correlations within microscale strain gradients derived from the Euler matrix and can reliably infer the direction of variant transfer even without incorporating the *m*′.

### Impact of nanoscale precipitates

**Fig. 6:**
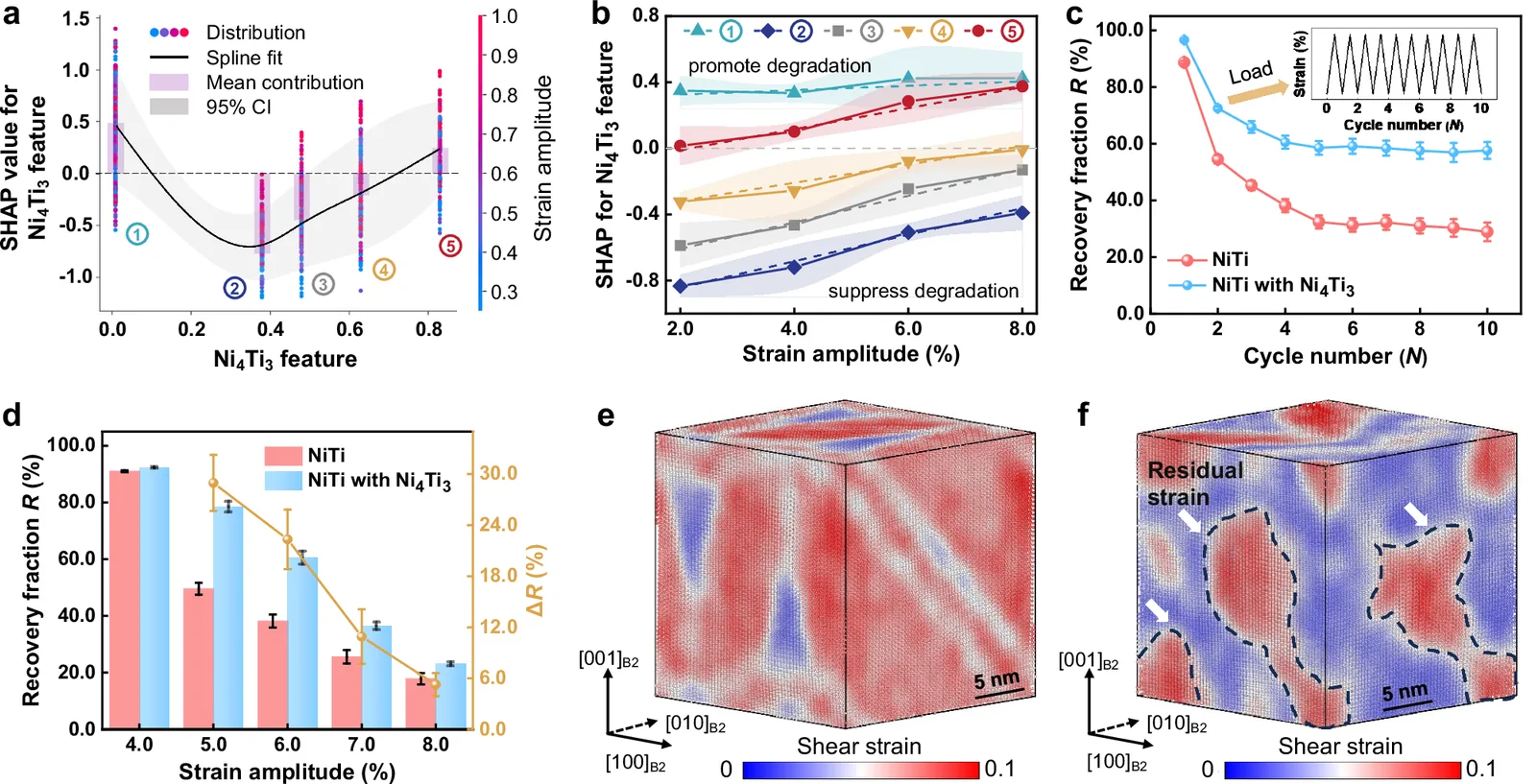
Impact of Ni_4_Ti_3_ precipitation. **a** SHAP dependence plot of the PCA-derived Ni_4_Ti_3_ feature, with individual samples colored by the corresponding strain amplitudes. Circled labels 1–5 denote distinct precipitation states under varying annealing conditions, where negative and positive SHAP values signify the suppression and promotion of SED, respectively. The shaded band represents the 95% confidence interval of the fitted spline smoother. **b** Amplitude-dependent mean contribution of the Ni_4_Ti_3_ feature for precipitation states 1–5. Shaded regions represent the mean ± two standard deviations (SD) derived from *n* = 10 independent replicates, and dotted lines represent linear fits. The suppression effect peaks at low amplitudes and gradually diminishes as the amplitude increases. **c** Evolution of the austenite recovery rate *R* as a function of cycle number, comparing NiTi models in the presence and absence of Ni_4_Ti_3_ precipitates. **d** *R* after the tenth cycle and the recovery differences between the two cases. Error bars denote the SD from three simulation runs. **e**, **f** Atomic shear strain distributions after cyclic loading. The system without precipitates (**e**) exhibits pronounced strain localization and residual deformation regions, whereas the system containing Ni_4_Ti_3_ precipitates (**f**) displays a more uniform deformation pattern and lower residual strain, demonstrating that precipitate-induced internal stress effectively suppresses functional degradation.

The contribution of nanoscale Ni_4_Ti_3_ precipitates is quantified (Fig. 6a). The circled labels 1–5 correspond to the characteristic parameters of Ni_4_Ti_3_ under different annealing conditions. Fine and uniformly distributed precipitates (Label 2) exhibit the lowest and negative SHAP values, indicating the strongest suppression of degradation. As the parameters increase, the SHAP values rise gradually, suggesting a weakening suppression effect. At high parameter values, the contribution turns positive, implying that coarse precipitates (Label 5) begin to promote degradation. When the contributions are projected onto different strain amplitudes (Fig. 6b), the suppression effect of Ni_4_Ti_3_ is most evident at low amplitudes and weakens as the amplitude increases.

To further validate this pattern, we constructed models with and without Ni_4_Ti_3_ precipitates of controlled size (Supplementary Fig. 53) and performed cyclic tensile simulations across multiple amplitudes. The precipitate-free model consistently exhibits the lowest austenite recovery fraction *R* (defined as the B2 volume fraction restored at the end of each unloading), indicating the fastest loss of superelasticity, as exemplified by the 6% amplitude case (Fig. 6c). Introducing Ni_4_Ti_3_ markedly suppresses degradation^8,39^. The effect is reproducible over many cycles and representative across the examined amplitude window. The degradation proceeds in stages, featuring a rapid decline over the first four cycles, followed by a gradual approach to a near-steady state over the subsequent six cycles. While the simulations necessarily employ a relatively high strain rate and the idealized monodisperse precipitate distribution^40^, the obtained pattern is consistent with the interpretive result.

Quantitatively, *R* evaluated after the tenth cycle decreases monotonically with increasing amplitude for both models (Fig. 6d). At 4% strain, plastic accumulation is negligible; at higher amplitudes, *R* declines progressively, yet Ni_4_Ti_3_ retains a discernible mitigating effect that weakens with increasing amplitude. The evolution of the phase topology and that of the residual strain are compared (Supplementary Figs. 54–57). The precipitate-free system develops early, spatially localized damage during the rapid degradation stage, which subsequently intensifies in confined regions (Fig. 6e). By contrast, the precipitate-containing system exhibits more homogeneous transformation kinetics and a reduced tendency toward localization—consistent with favorable precipitate-induced internal stresses and nucleation pathways—thereby substantially suppressing SED (Fig. 6f). Note that this beneficial effect diminishes as precipitates grow or coarsen, likely due to reduced or lost coherency at the matrix–precipitate interface^41^.

### Extensibility validation

**Fig. 7:**
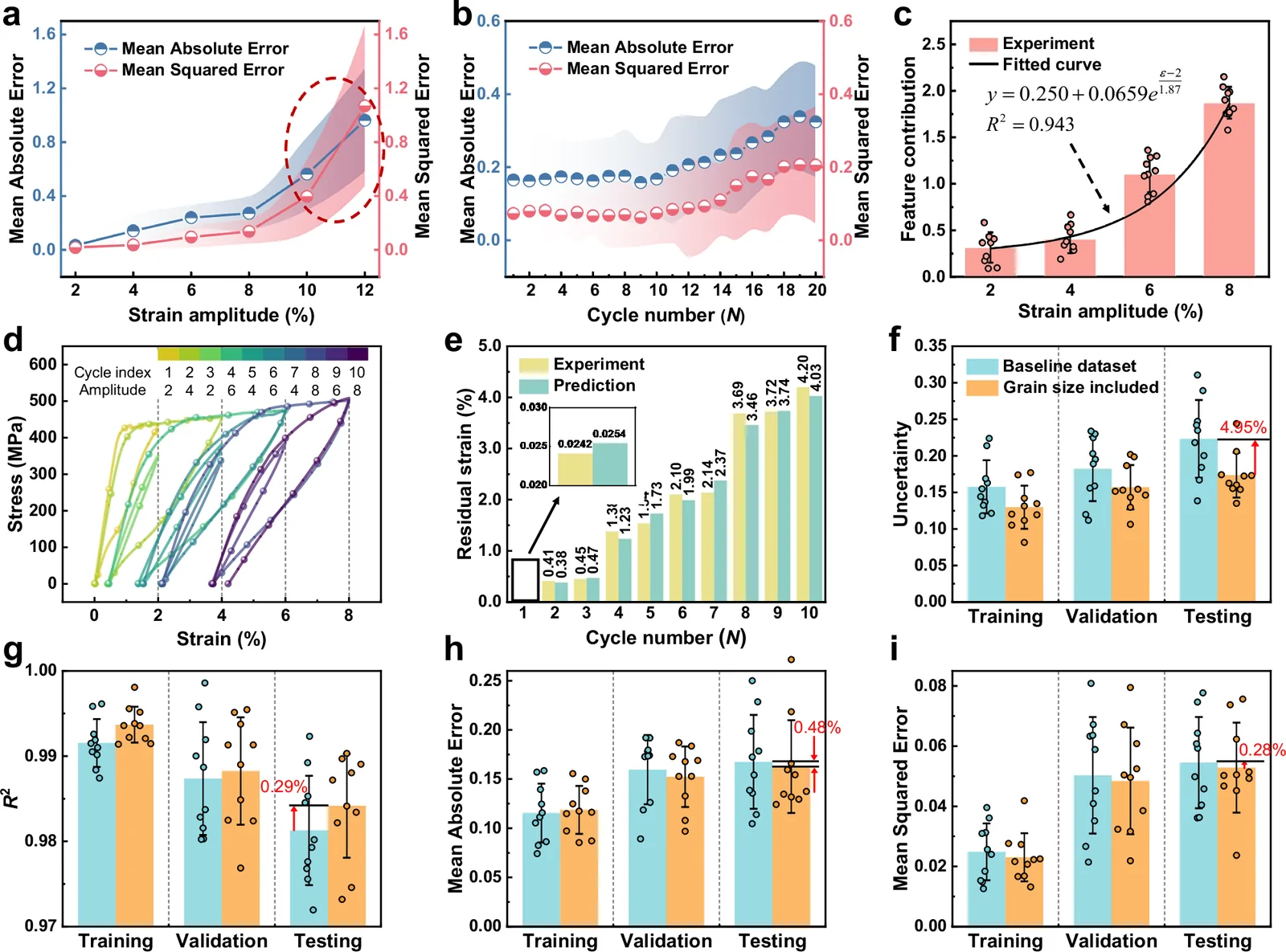
Extensibility and robustness validation of the multimodal framework. **a**, **b** Prediction errors under boundary-transcending conditions. When loading amplitudes exceed the training range (**a**), both MSE and MAE increase sharply. Errors remain stable even when the cycle number increases to 20 (**b**). **c** SHAP analysis attributes this discrepancy to an exponential increase in feature contributions as the strain amplitude rises from 2% to 8%. **d** Cyclic loading–unloading path over 10 cycles, with the prescribed amplitude sequence indicated above the stress–strain curve. **e** Model inference under frozen network parameters, successfully reproducing the cumulative evolution of residual strain when fed with the prescribed amplitude sequence. **f**–**i** Incorporation of grain size into the numerical branch yields nearly unchanged accuracy while reducing uncertainty by approximately 4.95% (**f**). Collectively, the approach demonstrates robust extensibility, and embedding physically meaningful structural variables effectively mitigates performance fluctuations. Shaded regions and error bars denote two and one SD, respectively, both calculated from *n* = 10 independent replicates. Source data are provided as a Source Data file.

The scenarios beyond the training distribution are tested. The overall error increases with amplitude; at 10% and 12%, the MAE and MSE rise approximately 2–3 times relative to the training range, while the residual distributions become more dispersed. SHAP analysis reveals the root cause of this trend: feature contributions exhibit exponential growth as amplitude increases from 2% to 8% (Fig. 7c), indicating progressively stronger influence on decisions. Beyond 8%, deformation behavior exceeds the learned domain, leading to unstable feature responses and rapid deterioration in generalization capability. In contrast, cycle number extrapolation demonstrates greater robustness when the loading is extended to 11–20.

Furthermore, the composite loading process, comprising 10 cycles of variable amplitudes, was investigated. This path more closely approximates real service scenarios^42^, exhibiting pronounced historical dependence in deformation (Fig. 7d). During inference, network parameters remain frozen^43^. Amplitude sequences are fed by cycle, with strain states updated iteratively to enable sequential prediction of amplitude variation. Over 10 cycles, the proposed approach tracks the cumulative evolution of residual strain, keeping errors within acceptable limits (Fig. 7e). This demonstrates the transferability of our framework under complex loading histories.

In addition to loading parameters and precipitate characteristics, grain size is another variable that influences the mechanical response of NiTi alloys. By modulating grain boundary density and dislocation storage capacity, it alters the nucleation sites and martensite evolution pathways, thereby affecting both the stress plateau and residual strain^44,45^. However, conventional methods struggle to directly characterize such a size effect. To address this, grain size was incorporated into the numerical branch. The mean prediction accuracy changes only marginally, but uncertainty shrinks by ≈4.95%. The structural descriptor stabilizes model performance under limited data. While grain size is not the decisive factor, it significantly enhances robustness and underscores the value of embedding physically meaningful parameters into data-driven modeling.

## Discussion

Superelastic degradation under cyclic loading poses a significant challenge to the stability of SMAs, as its evolution is jointly governed by microstructure, external load, and deformation history. Conventional variable-controlled approaches isolate individual factors under simplified assumptions, struggling to capture the full degradation process. This study proposes an interpretable multimodal framework. Compared with previous methods that utilized a single modality or statistical feature, its contribution lies not in creating yet another black-box predictor, but in providing a paradigm for organizing, leveraging, and understanding multi-source information in complex cyclic problems.

The complexity is manifested in the fact that localized deformation in NiTi precludes meaningful in-situ diffraction characterization throughout a continuous loading sequence^3,46–48^. Against this backdrop, the interpretable ML framework demonstrates a quasi-in-situ mechanistic reconstruction by resolving and visualizing degradation processes, thereby offering an alternative path to full-field observations that are otherwise elusive through traditional experimental or theoretical routes. Unlike molecular dynamics simulations, which hinge on precise potentials and idealized initial configurations^49^, this approach spontaneously extracts statistical patterns directly from real microstructures. By corroborating these learned patterns with independent experimental and simulation findings, this study narrows the existing observational gap and lays the foundation for future calibration of multiscale theoretical models^11,50^.

On this basis, the competition between slip and SIMT is disentangled across varying strain amplitudes and cyclic stages, yielding a sharper physical landscape than earlier conceptual impressions. Using the Schmid factor^4,32^ as a criterion, we correlated localized hotspots with crystal orientation and stress state. At high amplitudes, degradation is dominated by grains with elevated Schmid factors for SIMT, typified by the emergence of {114} austenite twins during the B2–B19′ cyclic transition. At low amplitudes, dislocation slip on {011}〈001〉 and {011}〈111〉 systems is preferentially activated, and degradation is driven by the accumulation of irreversible defects. Once this mapping is converted into measurable spatial distributions, we can track the migration of dominant deformation zones across the entire gauge, moving beyond empirical judgments limited to isolated grains or restricted fields of view. Together, these results integrate previously qualitative and fragmented mechanistic insights into a more unified perspective: SED of NiTi emerges as a competition between mechanisms regulated by loading conditions, with the active pathway selected at the microscale by crystallographic geometric compatibility. This process can now be quantified and predicted in a data-driven manner.

The longstanding notion^6,51^ that Ni_4_Ti_3_ precipitates are uniformly beneficial is revisited. Through TEM statistics followed by PCA compression (Supplementary Table 4), the influence of Ni_4_Ti_3_ was found to be conditionally dependent rather than monotonically positive. Under loading regimes that favor SIMT, a moderate dispersion of fine precipitates stabilizes superelasticity by elevating local transformation stresses and strengthening dislocation pinning. Conversely, coarse precipitates coupled with high amplitudes intensify damage and accelerate SED. The pronounced mechanical mismatch at precipitate–matrix interfaces introduces additional stress concentrations^9^, while even slight Ni depletion (~0.1 at.%) during nucleation noticeably alters transformation temperatures and stress plateaus^52^, thereby reshaping the reversible transformation window at room temperature. This underscores that material behavior is not governed by any single rule; treating Ni_4_Ti_3_ as a standalone variable risks obscuring its detrimental effects under specific conditions. Therefore, this study advocates an analysis method that jointly characterizes multiple factors and their coupling within a multimodal ensemble learning framework, ensuring more reliable and objective assessment.

From a design perspective, the above insights can be directly translated into actionable guidelines for processing and service windows. For components requiring high functional stability, such as brackets and actuators, two principles are paramount: (i) employ annealing treatments that promote the nucleation of fine, homogeneously dispersed Ni_4_Ti_3_ precipitates while suppressing overaging-induced coarsening; (ii) define operational strain envelopes within regimes where SIMT is fully activated but dislocation slip remains marginal, allowing reversible twinning to accommodate deformation while delaying defect accumulation. For damping or energy dissipation elements^1^, lower amplitudes are more conducive to the formation of dislocation modulation structures, and can yield higher dissipation capacity under managed degradation. Overall, transforming mechanism maps into design criteria signals a shift in NiTi design from trial-and-error toward refined guidance grounded in verifiable principles.

Notably, the significance of this interpretable framework extends beyond merely elucidating degradation mechanisms; its architecture also embodies a clear physical hierarchy. Crystallographic information is encoded in the image branch through representations such as Euler matrices, while loading conditions and precipitation features are represented as numerical vectors. Cross-modal alignment and merging are performed in the synergy module. This modular design grants the model natural extensibility when confronted with additional variables. For instance, grain size *d* can be introduced to capture Hall–Petch strengthening^53^ and guide the design of nanocrystalline NiTi, while the thickness and grain size ratio *t/d* can be considered to explore hitherto overlooked size effects in NiTi sheets. Although thermo-mechanical coupling is not systematically investigated here, the present approach enables seamless integration of external factors such as temperature and strain rate (Supplementary Figs. 47, 48) to address more demanding scenarios^54^. By establishing a unified coordinate scheme for microstructure, loading, and properties, this work formulates a generalizable protocol for design and analysis rather than a model restricted to a specific dataset.

The framework certainly exhibits predictive boundaries over the engineering-relevant regime. Across the 2%–8% amplitude range, the model accurately reproduces the evolution of multistage response features and demonstrates superior extrapolation capability with respect to cycle number. Consequently, even with limited experimental data and cost, it holds great promise for evaluating fatigue damage^48,55^ and functional degradation^56,57^ over thousands to tens of thousands of loading cycles. However, once the amplitude exceeds the training interval, the exponential amplification of feature contributions will rapidly inflate the error. Extrapolation should therefore be anchored by experimental benchmarks and not viewed as an unconditional substitute for physical testing. Nevertheless, the current study covers the primary operational scope of most NiTi functional devices. For example, stretchable conductors in flexible electronics typically accommodate 2%–4% reversible strain to maintain electrical continuity, whereas medical devices for soft-tissue support or implant repair generally operate at strains below 8%.

Admittedly, the interpretative method identifies and quantifies high-dimensional correlations in multimodal data. When applied to examine qualitative mechanistic hypotheses, it provides spatially resolved, quantitative evidence rather than independently establishing rigorous causal laws, although the findings are strongly corroborated by Schmid factor analysis and TEM observations. Furthermore, as the present work relies on 2D EBSD data, the bulk stress states and intergranular constraints governing transformation pathways and slip-SIMT competition are inevitably simplified^58,59^. This approximation neglects out-of-plane stress components and subsurface grain interactions, potentially constraining the representativeness of Schmid factor analysis and the interpretation of microstructural evolution. In this context, EBSD preprocessing (e.g., denoising intensity and step size) directly influences the precision of hotspot localization and may even obscure subtle orientation gradients. While the identified physical trends are unlikely to be attributable to preprocessing variations and are supported by multiscale experiments and simulations, systematic evaluation of model sensitivity remains essential for further optimization. Future research should integrate 3D EBSD, in situ diffraction, and X-ray tomography to bridge the gap between planar characterization and bulk deformation behavior. Augmented with 3D structural information and enhanced robustness to noise, the framework will occupy a superior vantage point for deconstructing the origin of functional fatigue in SMAs.

Collectively, this study developed a comprehensive, simple, interpretable, and extensible multimodal learning framework to explore the SED of NiTi shape memory alloys. In essence, once multimodal data are organized into representations and leveraged by interpretable ML, they serve as the language for understanding material deformation behavior rather than merely as experimental records. This marks a shift in smart materials research from property characterization toward mechanistic insight, paving the way for more intelligent, cognitive systems in the field.

## Methods

### X-ray diffraction

X-ray diffraction (XRD) testing was performed on a SmartLab SE diffractometer manufactured by Rigaku Corporation of Japan. The test employed Cu Kα radiation (*λ* = 1.5406 Å) at room temperature using a *θ*–2*θ* reflection geometry. The scan range was 10°–90° (2*θ*) with a step interval of 0.02°. The incident beam illuminated the sample surface, and the diffraction signal was collected by a high-resolution detector to obtain a two-dimensional diffraction pattern. The two-dimensional diffraction pattern was converted to a one-dimensional diffraction spectrum using Rigaku SmartLab Studio II software (version 4.5.286.0). Gaussian fitting was used to determine peak areas and positions for phase analysis. The relative peak intensity measurement error was less than 0.1%.

### DSC characterization

The thermally induced transformation behavior of NiTi alloys was characterized in accordance with ASTM F2004-05 using a simultaneous thermal analyzer (STA449F3). Disk-shaped samples (~2 mm in diameter and 0.5 mm in thickness) were mechanically ground to ensure flat surfaces and optimal thermal contact. Testing was conducted under a continuous flow of high-purity nitrogen. To establish a consistent initial state, samples were first cooled from room temperature to −150 °C at 20 °C/min and held for 1 min. Subsequently, the heat flow was recorded during heating to 150 °C and subsequent cooling to −150 °C, both at a constant rate of 10 °C/min. Characteristic transformation temperatures, including the start (*M*_s_, *A*_s_), peak (*M*_p_, *A*_p_), and finish (*M*_f_, *A*_f_) temperatures, were determined using the standard tangent method. Transformation enthalpies (∆*H*) were calculated by integrating the areas of the endothermic and exothermic peaks relative to the baseline. Additionally, thermal hysteresis was quantitatively evaluated as:

1 $$\Delta T=({A}_{s}+{A}_{f})/2-({M}_{s}+{M}_{f})/2$$

### Room temperature cyclic tensile loading and unloading

Tensile specimens (70 × 10 × 0.5 mm^3^) were fabricated from annealed NiTi sheets by electrical discharge wire cutting, with the loading axis aligned with the rolling direction. The gauge section measured 25 mm in length and 6 mm in width. Specimen surfaces were sequentially polished with graded sandpaper and ultrasonically cleaned with acetone solution to remove surface residues. Cyclic loading-unloading tests were performed on an MTS universal testing machine at a constant strain rate of 5 × 10^−4^ s^−1^. Strain was measured in real time using an Epsilon Technology Model 3442 extensometer to ensure accuracy. All tests were conducted at room temperature and repeated three times to verify reproducibility. In each cycle, specimens were loaded to a prescribed strain amplitude and immediately unloaded to zero stress. Residual strain (*ε*_res_), defined as the strain at the intersection of the unloading curve with the strain axis, was selected as the primary degradation metric. Compared with hysteresis-based descriptors such as hysteresis width or dissipated energy, *ε*_res_ provides a more robust measure of the cumulative loss of recoverable deformation capacity. Full-field digital image correlation was employed to monitor SIMT during testing, and the amplitudes were defined from uniaxial tensile tests calibrated with the same DIC setup (Supplementary Fig. 12).

### Molecular dynamics simulation

In this study, the large-scale atomic/molecular massively parallel simulator (LAMMPS, version 28Mar2023) was employed^60^, using the widely adopted 2NN MEAM interatomic potential^49^ with a time step of 1 fs. The model was constructed using ATOMSK 0.13.1 software^61^ to ensure interface coherency. Configurations were visualized and analyzed using OVITO Pro 3.10.0 software^62^, in which the crystal structure and orientation were identified using the Polyhedral Template Matching (PTM) algorithm. The atomic strain was calculated using the von Mises local shear invariant theory via the atomic strain modifier in OVITO. The simulation system was modeled under an NPT (constant number of particles, pressure, and temperature) ensemble with zero external pressure. Periodic boundary conditions were applied to the three-dimensional system to eliminate surface effects. During the cyclic simulation process, energy minimization was first carried out using the conjugate gradient method, followed by a 10 ps relaxation at 300 K and zero external pressure. Finally, cyclic tensile simulations were performed at a strain rate of 10^8^ s^−1^.

### Training the DL model

The final dataset comprises approximately 31,536 labeled sample sequences. To prevent information leakage arising from spatial overlap, data partitioning was performed at the level of the original EBSD scan regions, ensuring that all samples derived from a single region were assigned to the same subset. Specifically, the training set was used for parameter optimization, while the validation set was used for performance monitoring and hyperparameter tuning. Feedback from the validation loss determined the early stopping criteria and model selection. The test set was strictly reserved for the final, unbiased performance evaluation and remained independent of all training and adjustment processes. Following partitioning, offline data augmentation was applied exclusively to the training set using cropped sub-images to enhance generalization. The validation and test sets contained only original, unaugmented data. Prediction scatter plots were generated from grouped tenfold cross-validated predictions over the original labels, whereas final metrics were computed on the independent test set from the fixed 8:1:1 split. Model performance was evaluated using the coefficient of determination (*R*^2^), mean squared error (MSE), and mean absolute error (MAE)^20^.

Experiments were conducted on a 64-bit Windows 10 operating system equipped with dual Intel Xeon Platinum 8336C CPUs, 224 GB of RAM, and an NVIDIA GeForce RTX 4090 GPU with 24 GB of dedicated VRAM. The software environment comprised CUDA 11.1, Python 3.8.8, and PyTorch 1.9.0^63^. Training was performed using the MSE as the loss function and the Adam optimizer. The initial learning rate was set to 1 × 10^−3^ and gradually decayed during training using a cosine annealing strategy. The batch size was set to 32, with a maximum of 150 training iterations. After each iteration, the loss was computed on the validation set, and the model weights with the lowest validation error were saved. Training was automatically terminated when the validation loss did not decrease for 10 consecutive iterations, thereby preventing overfitting.

Additionally, four comparative prediction models were constructed in this study: MCC-Net, ECMMF-Net, Base-w/oSM, and MBNI-Net. These models share the same computational environment as the main MBFFA model, and their parameter configurations are summarized in Supplementary Table 9. All implementations are based on the PyTorch framework and trained on the self-constructed dataset to ensure optimal performance.

### Calculation and correction of the Schmid factor

The Schmid factor for each orientation is calculated from raw Euler angle data obtained by EBSD. The Euler angles of each pixel are converted into a crystal orientation matrix, with the principal stress axes aligned with the applied load direction. For each potential deformation system, the Schmid factor *m* is computed as follows:

2 $$m=\cos \lambda \cos \phi$$

Here, *λ* and *ϕ* represent the angles between the loading direction and the slip direction, and between the loading direction and the slip plane normal, respectively. In the slip system calculations, two primary slip systems commonly found in B2 NiTi—{011}〈001〉 and {011}〈111〉—were considered. For the calculation of the transformation Schmid factor, 48 sets of martensitic correspondence variant pairs (CVP) associated with the stress-induced B2 → B19′ transformation were considered^64–66^, encompassing both {111} Type I and <011> Type II twin variants. The shear directions and normal vectors to the habit planes for each variant were determined based on lattice parameters and transformed into the experimental coordinate system for computation. The maximum values among all slip systems and variants were selected to construct the Schmid factor distribution diagram.

In samples containing nanoscale Ni_4_Ti_3_ precipitates, lattice mismatch between the precipitates and the matrix generates a localized elastic stress field, thereby altering the critical conditions for different deformation mechanisms^9,39^. In slip systems, the mismatch strain induced by precipitates generates an additional shear stress in the matrix opposite to the applied load, thereby forming a back stress. Local stress correction is calculated using the Eshelby method^67^, expressed as:

3 $${\tau }_{{{\rm{i}}}}(x)={\alpha }_{{ij}}^{{\prime} }{C}_{{ijkl}}{D}_{{klmn}}\left(x\right){\varepsilon }_{{mn}}^{p}$$

Where ${\alpha }_{{ij}}^{{\prime} }$ is the orientation tensor expressed in the principal coordinate system of the precipitate, *C*_*ijkl*_ is the elastic constant tensor, ${D}_{{klmn}}\left(x\right)$ is a fourth-order interaction tensor constructed from the Eshelby tensor that relates the precipitate eigenstrain to the induced elastic field in the matrix at any position *x*, and ${\varepsilon }_{{mn}}^{p}$ is the lattice mismatch strain between the precipitate phase and the matrix. The modified effective decomposed shear stress is ${\tau }_{{{\rm{eff}}}}={\tau }_{{{\rm{e}}}}+{\tau }_{{{\rm{i}}}}$. This section describes the modulating effect of reverse internal stresses introduced by precipitates on the conditions for slip activation, namely, increasing the critical stress required for dislocation slip and suppressing its local occurrence. Conversely, in SIMT systems, the mismatch stress induced by precipitates increases the local driving force, thereby reducing the critical stress required for transformation. In the calculations, a local stress component was superimposed on the results for each martensite variant to correct the transformation Schmid factor. When the precipitate size exceeded approximately 300 nm and lost its semi-coherent relationship, the influence of the local stress field became negligible. The calculation process was performed on each EBSD pixel using a custom-developed program.

### TEM characterization

The target sample was prepared into thin sections approximately 50 μm thick via slicing, thinning, and mechanical polishing, and then punched into 3-mm-diameter disks for transmission electron microscopy (TEM) analysis. Perforation was achieved using a dual-jet electrolytic polishing method at −35 °C with a nitrate-methanol solution. Routine microscopic characterization was performed on an FEI Talos F200X TEM equipped with an energy dispersive spectrometer (EDS) at an acceleration voltage of 200 kV. Atomic-scale high-angle annular dark-field (HAADF) images were acquired in STEM mode at 300 kV on an FEI Themis Z microscope equipped with a probe aberration corrector and Super-X EDS detector. Strain field analysis (GPA)^68^ was performed using the FRWR plugin in Digital Micrograph.

### EBSD experiment

After undergoing stepwise mechanical grinding and polishing to a mirror finish, the samples were placed in a VP1 vibratory polisher. CSPS1 silica polishing slurry was applied for at least 4 h to achieve a high-quality surface free of scratches and oxide films. After polishing, the samples were ultrasonically cleaned in acetone and air-dried. Electron backscatter diffraction (EBSD) testing was performed on a FEI Verios 5 UC scanning electron microscope equipped with an Oxford Symmetry detector. Test parameters included a voltage of 20 kV, a step size of 0.2 μm, a working distance of approximately 15 mm, and a sample tilt angle of 70°. Patterns were indexed using the standard Hough-transform-based routine implemented in the AZtec software (version 5.0). Three distinct regions were independently scanned for each annealing condition, with raw image resolution of 1024 × 720 pixels.

Post-processing was performed using Oxford Instruments AZtecCrystal software (version 2.1). EBSD data were subjected to the removal of isolated single pixels (i.e., wild spikes) and a six nearest neighbor zero-solution correction. The mean angular deviation (MAD) was calculated for each indexed point; the average MAD across all EBSD maps was less than 0.62°. No data points were excluded based on MAD or other quality metrics. The overall indexing rates for EBSD characterizations under different annealing conditions are summarized in Supplementary Table 2. The original images were subsequently cropped into six sub-images of 336 × 336 pixels, a size that balances grain-morphology integrity with local structural detail. Data augmentation was applied to these cropped images using offline operations such as rotation and mirroring (Supplementary Fig. 29).

### Implementation of interpretive technologies

To achieve a comprehensive interpretable analysis, UMAP^69^, Grad-CAM, and SHAP^70^ methods were employed. Following model training, latent feature vectors were extracted from 3360 original training samples (excluding augmented data) at the corresponding network layer. Dimensionality reduction was performed using the umap-learn v0.5.7 package to project the features into a 2D space for visualization. The manifold learning parameters were set to n_neighbors = 15 and min_dist = 0.1, with identical configurations used across all comparative visualizations. Scatter points were colored by true residual strain or other key physical variables, enabling intuitive interpretation of the latent space structure and its relation to deformation behavior. Grad-CAM was applied to identify spatial regions contributing to the predictions. During backpropagation, gradients were retained only for the target branch, while those of other branches were frozen. To achieve specific visualization, parameters of the numerical branch were fixed, and only the microstructure inputs corresponding to prescribed cycle numbers and strain amplitudes were varied. Forward propagation then generated conditional activation maps, which were converted into corresponding heatmaps. Finally, SHAP analysis was conducted on the composite feature vector at the input of the synergy module. This vector was constructed by concatenating numerical features with image-modality representations, with labels appended to distinguish the origins of features across different branches.

## Supplementary information

Supplementary Information

Transparent Peer Review file

## Source data

Source Data

## Data Availability

The Source Data file contains the numerical data underlying the main figures and selected Supplementary Figs. Raw X-ray diffraction, in situ XRD, and DSC data are available on Figshare^71^. Additional raw datasets, including raw EBSD data, raw TEM data, cyclic tensile loading–unloading data, digital image correlation data, and molecular dynamics simulation output files, are available from the corresponding authors upon request. Source data are provided with this paper.

## Code availability

The code implementing the multimodal MBFFA framework, including model training and evaluation in this study, is available on GitHub at (https://github.com/NiTiShapeMemoryAlloy/Multimodal-MBFFA). A permanent version is released on Zenodo^72^.
