# Supplementary material for: An interpretable learning framework for exploring superelastic degradation of NiTi shape memory alloys using multimodal data
Source: Nat Commun. 2026 Aug 31;17:10360. doi: 10.1038/s41467-026-77250-4 (PMC13623974; doi:10.1038/s41467-026-77250-4)
Supplement: Supplementary file 1 [file 41467_2026_77250_MOESM1_ESM.pdf]

Supplementary information

An interpretable learning framework for exploring superelastic degradation of NiTi shape memory alloys using multimodal data

Yuejie Hu[1], Haiyang Wang[1], Ming Chen[2,3], Gang Chen[1,4], Chuanjie Wang[1,4,*], Bin Guo[4], M.W. Fu[5,*]

[1] School of Materials Science and Engineering, Harbin Institute of Technology at Weihai, Weihai 264209, China

[2] State Key Laboratory of Metal Matrix Composites, Shanghai Jiao Tong University, Shanghai 200240, China

[3] School of Materials Science and Engineering, Shanghai Jiao Tong University, Shanghai 200240, China

[4] Key Laboratory of Micro-Systems and Micro-Structures Manufacturing (Harbin Institute of Technology), Ministry of Education, Harbin 150080, China

[5] Department of Mechanical Engineering, The Hong Kong Polytechnic University, Hung Hom, Kowloon, Hong Kong, China

[*]Corresponding authors. E-mail: cjwang@hitwh.edu.cn; mmmwfu@polyu.edu.hk

Inventory of Supporting Information

Supplementary Figure 1: Cyclic tensile loading–unloading engineering stress–strain curves of NiTi alloys under different annealing conditions

Supplementary Figure 2: Cyclic tensile loading–unloading engineering stress–strain curves at a strain rate of 10^{-3} s^{-1}

Supplementary Figure 3: Cyclic tensile loading–unloading engineering stress–strain curves at a strain rate of 10^{-2} s^{-1}

Supplementary Figure 4: Cyclic tensile loading–unloading engineering stress–strain curves at a strain rate of 3×10^{-2} s^{-1}

Supplementary Figure 5: Cyclic tensile loading–unloading engineering stress–strain curves at 40 °C

Supplementary Figure 6: Residual strain accumulation under different annealing conditions

Supplementary Figure 7: Residual strain accumulation at a strain rate of 10^{-3} s^{-1}

Supplementary Figure 8: Residual strain accumulation at a strain rate of 10^{-2} s^{-1}

Supplementary Figure 9: Residual strain accumulation at a strain rate of 3×10^{-2} s^{-1}

Supplementary Figure 10: Dissipated energy density under different annealing conditions

Supplementary Figure 11: Forward transformation start stress

Supplementary Figure 12: Digital image correlation setup for full-field strain measurement

Supplementary Figure 13: First-cycle deformation analysis by digital image correlation

Supplementary Figure 14: Cyclic deformation analysis by digital image correlation

Supplementary Figure 15: X-ray diffraction patterns of NiTi alloys

Supplementary Figure 16: Differential scanning calorimetry characterization of phase transformation behavior

Supplementary Figure 17: EBSD characterization of the specimen annealed at 600 °C for 30 min

Supplementary Figure 18: Mean KAM values and representative KAM maps

Supplementary Figure 19: Orientation relationship between Ni_4Ti_3 precipitates and the B2 austenite matrix

Supplementary Figure 20: Quantitative statistics of Ni_4Ti_3 precipitates under different annealing conditions

Supplementary Figure 21: Tensile testing at 200 °C for determining the critical stress for slip

Supplementary Figure 22: Phase evolution during tensile loading characterized by in situ X-ray diffraction

Supplementary Figure 23: TEM characterization of NiTi after cyclic loading

Supplementary Figure 24: EBSD characterization after 5 cycles at 8% strain amplitude
Supplementary Figure 25: EBSD characterization after 10 cycles at 8% strain amplitude
Supplementary Figure 26: Dataset construction and partitioning workflow
Supplementary Figure 27: Design concept and structure of the feature synergy module
Supplementary Figure 28: Single-branch network architecture
Supplementary Figure 29: Data augmentation effect on the GND map
Supplementary Figure 30: Evolution of MSE loss during model training
Supplementary Figure 31: Learning curve and overfitting assessment
Supplementary Figure 32: Association between multimodal representation evolution and prediction performance
Supplementary Figure 33: Ablation study on the number of feature representations
Supplementary Figure 34: Sensitivity analysis of data-splitting ratios and network blocks
Supplementary Figure 35: Illustration of input modalities and model components
Supplementary Figure 36: Influence of network complexity on model performance
Supplementary Figure 37: Model generalization and overfitting assessment based on 10-fold cross-validation
Supplementary Figure 38: UMAP visualization of latent spaces before and after feature synergy
Supplementary Figure 39: UMAP manifold evolution and multi-physical mapping across feature dimensions
Supplementary Figure 40: Cosine similarity distributions for features in latent space
Supplementary Figure 41: Global interpretability based on SHAP analysis
Supplementary Figure 42: Local interpretability across six annealing conditions
Supplementary Figure 43: Feature interaction matrices across different degradation levels
Supplementary Figure 44: Comparison of Grad-CAM visualizations
Supplementary Figure 45: Comparative Grad-CAM visualizations across multimodal inputs
Supplementary Figure 46: Euler matrix response heatmaps for deformation analysis
Supplementary Figure 47: Predictive performance of residual strain across varying strain rates
Supplementary Figure 48: Predictive performance under different temperature conditions
Supplementary Figure 49: Predictive performance of dissipated energy
Supplementary Figure 50: Predictive performance of forward transformation start stress
Supplementary Figure 51: Ablation study with additional mechanical descriptors
Supplementary Figure 52: Microstructural characterization and degradation prediction for the specimen annealed at 800 °C for 1 h

Supplementary Figure 53: Atomistic models used in molecular dynamics simulations
Supplementary Figure 54: Atomistic configurations of NiTi after each loading cycle
Supplementary Figure 55: Atomistic configurations of NiTi with Ni_4Ti_3 precipitates
Supplementary Figure 56: Atomic strain maps of NiTi after each loading cycle
Supplementary Figure 57: Atomic strain maps of NiTi with Ni_4Ti_3 precipitates
Supplementary Table 1: Transformation stress of NiTi specimens
Supplementary Table 2: Mean angular deviation and indexing rates of EBSD scans
Supplementary Table 3: Transformation temperatures of NiTi alloys
Supplementary Table 4: PCA loading matrix and explained variance for the Ni_4Ti_3 precipitate descriptor
Supplementary Table 5: Theoretical solutions of the habit plane system for <011> type II twins
Supplementary Table 6: Theoretical solutions of the habit plane system for {11-1} type I twins
Supplementary Table 7: Summary of studies on superelastic degradation mechanisms
Supplementary Table 8: Hyper-parameters and their descriptions
Supplementary Table 9: Initialization parameters of different machine-learning models
Supplementary Table 10: Ablation study of model components in MBFFA
Supplementary Table 11: Comparative prediction performance of GND and KAM features under different fusion strategies
Supplementary Table 12: Statistical comparison of model performance
Supplementary Method 1: Construction of the Ni_4Ti_3 precipitate descriptor by principal component analysis
Supplementary Method 2: Calculation and correction of the Schmid factor
Supplementary Method 3: Dataset construction and multimodal learning framework description
Supplementary Method 4: Data augmentation strategy
Supplementary Method 5: Molecular dynamics cyclic loading and atomic strain calculation
Supplementary Note 1: Electron backscatter diffraction characterization
Supplementary Note 2: Deep learning model structure
Supplementary Discussion 1: Multimodal representation and performance
Supplementary Discussion 2: Input modality design, ablation variants and feature synergy analysis
Supplementary References: References cited in the Supplementary Information

Supplementary Figure 1

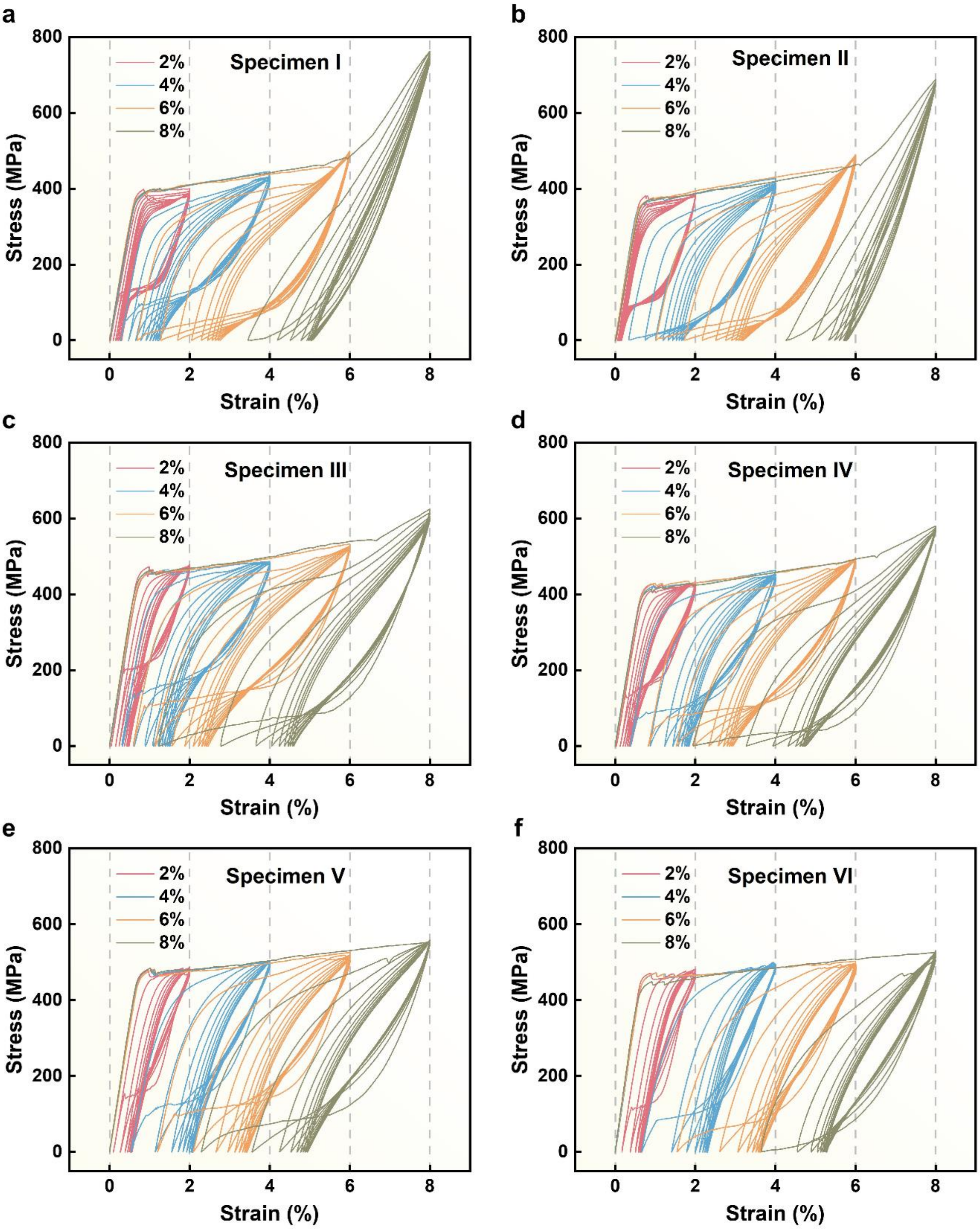

Supplementary Fig. 1 | Cyclic tensile loading–unloading engineering stress–strain curves of NiTi alloys under different annealing conditions. (**a**–**f**) correspond to specimens I–VI, respectively, showing the cyclic test results under strain amplitudes of 2%, 4%, 6%, and 8%.

Supplementary Figure 2

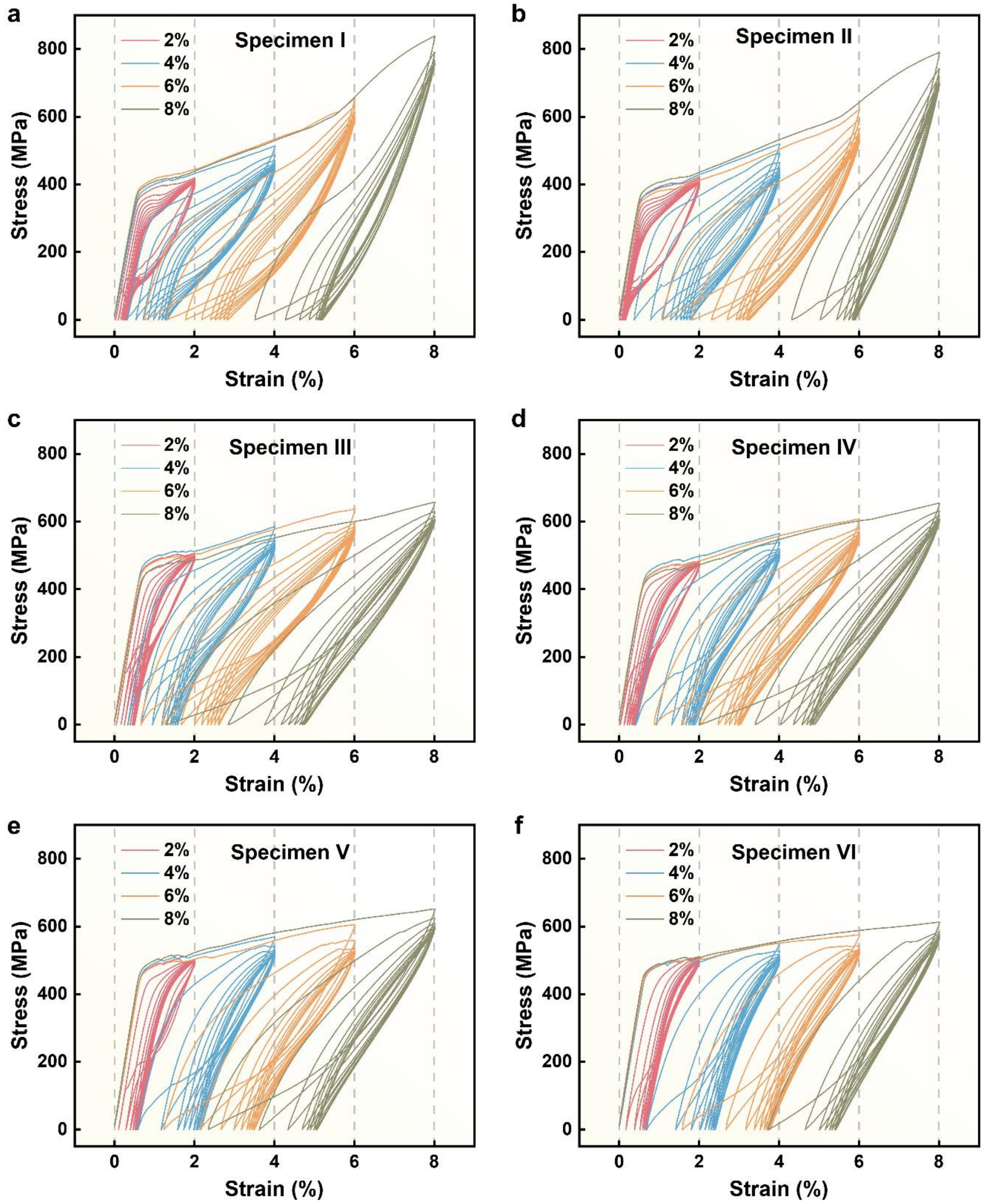

Supplementary Fig. 2 | Cyclic tensile loading–unloading engineering stress–strain curves of NiTi alloys at a strain rate of 10^{-3} s^{-1}. (**a**–**f**) correspond to specimens I–VI, respectively, showing the cyclic test results under strain amplitudes of 2%, 4%, 6%, and 8%.

Supplementary Figure 3

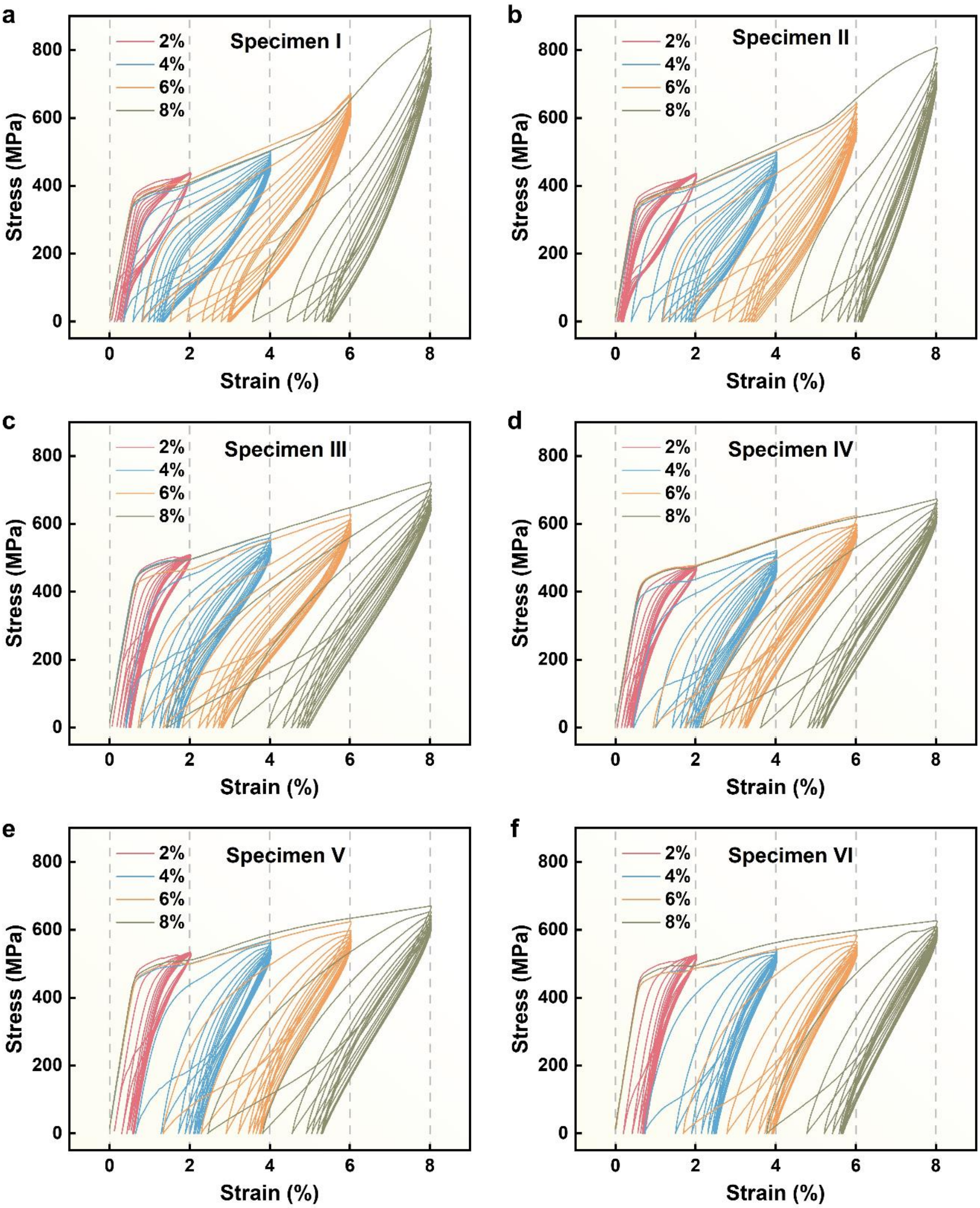

Supplementary Fig. 3 | Cyclic tensile loading–unloading engineering stress–strain curves of NiTi alloys at a strain rate of 10^{-2} s^{-1}. (**a**–**f**) correspond to specimens I–VI, respectively, showing the cyclic test results under strain amplitudes of 2%, 4%, 6%, and 8%.

Supplementary Figure 4

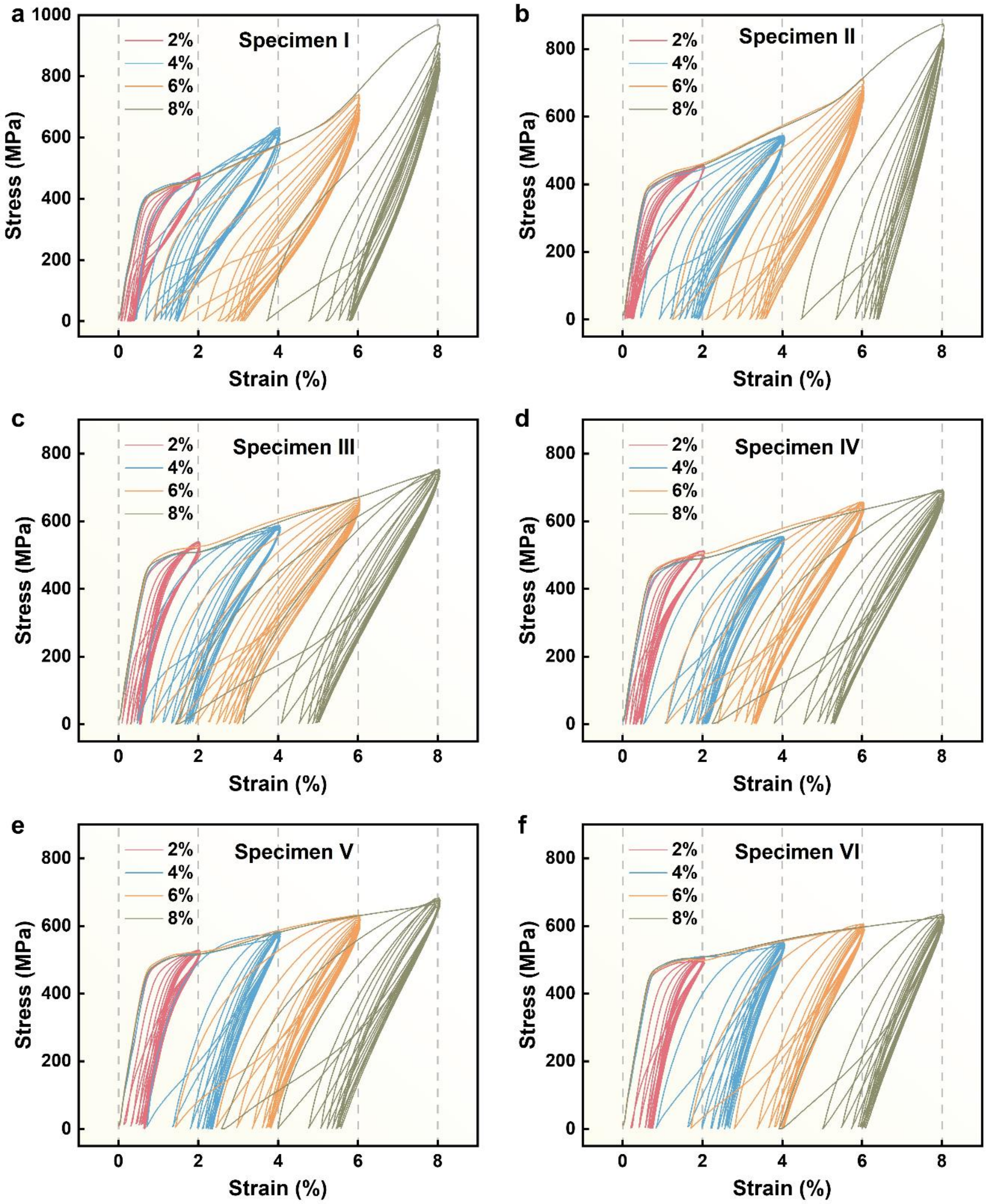

Supplementary Fig. 4 | Cyclic tensile loading–unloading engineering stress–strain curves of NiTi alloys at a strain rate of 3 × 10^{-2} s^{-1}. (**a**–**f**) correspond to specimens I–VI, respectively, showing the cyclic test results under strain amplitudes of 2%, 4%, 6%, and 8%. Due to equipment overshoot at this high strain rate, the raw data were denoised to ensure accurate identification of cyclic characteristics.

Supplementary Figure 5

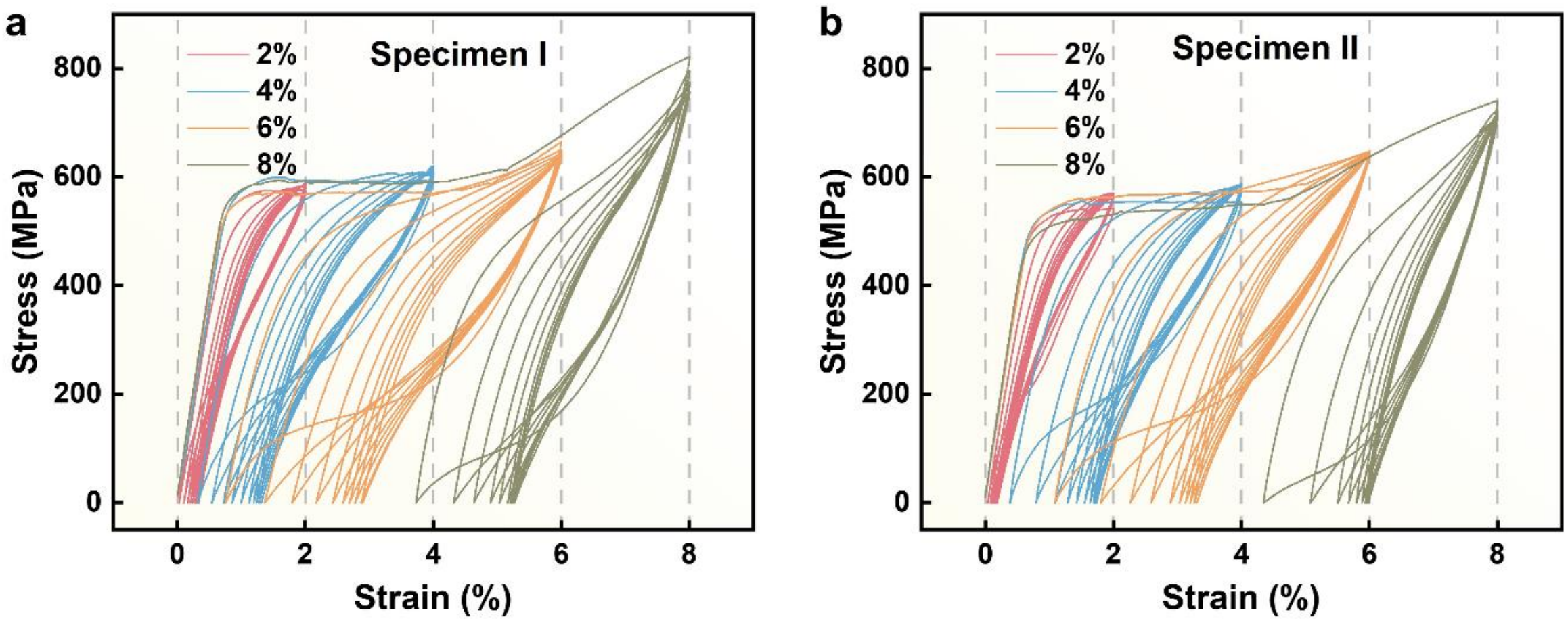

Supplementary Fig. 5 | Cyclic tensile loading–unloading engineering stress–strain curves of NiTi alloys at 40 °C under a strain rate of 5 × 10^{-4} s^{-1}. (**a, b**) correspond to specimens I and II, respectively, showing the cyclic test results under strain amplitudes of 2%, 4%, 6%, and 8%.

Supplementary Figure 6

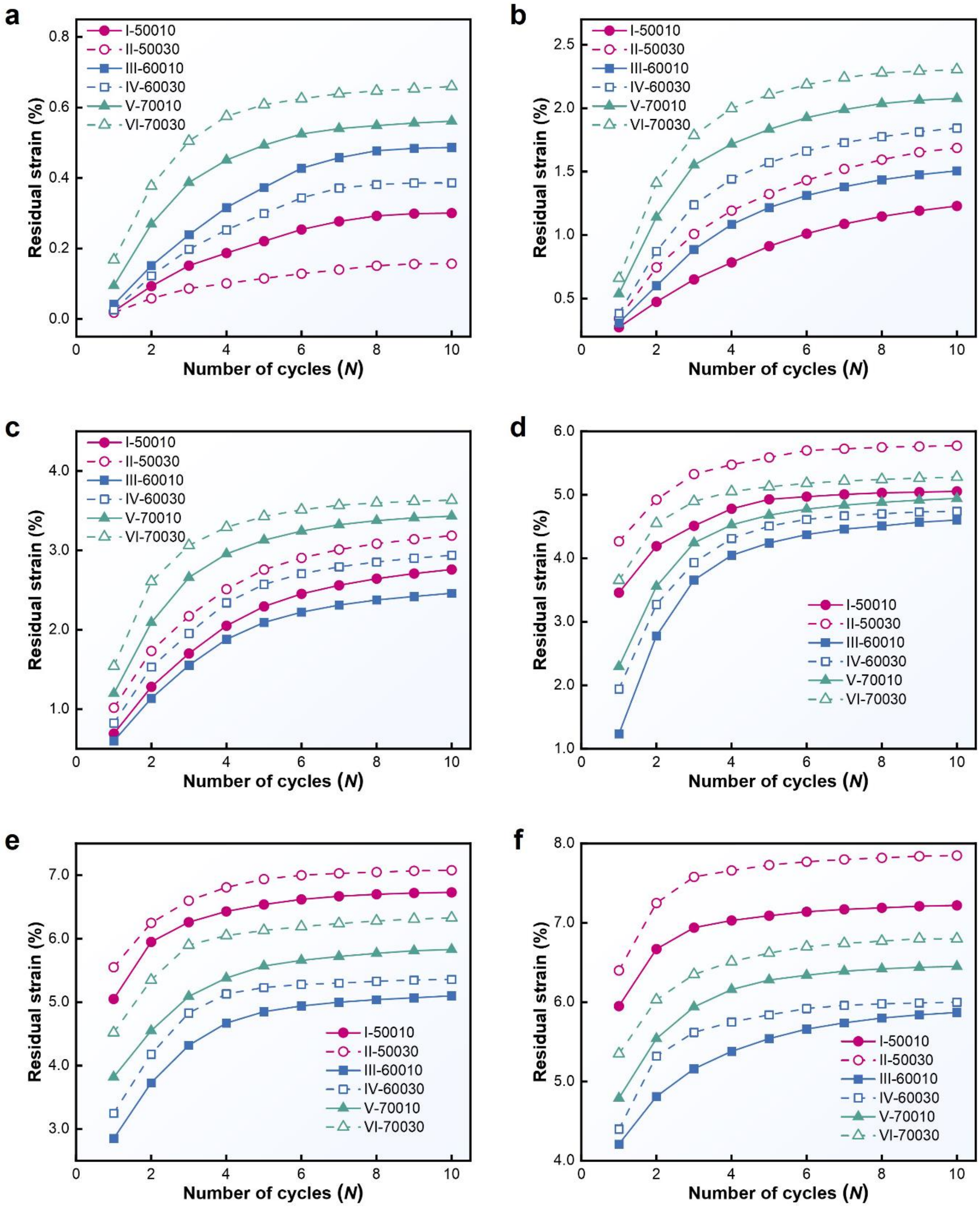

Supplementary Fig. 6 | Residual strain accumulation of NiTi alloys under various annealing conditions. Evolution of residual strain with increasing cycle number at strain amplitudes of (**a**) 2%, (**b**) 4%, (**c**) 6%, (**d**) 8%, (**e**) 10%, and (**f**) 12%. The first four amplitudes were employed for model training and validation. The latter two were used to evaluate extensibility, where the material had fully entered the martensitic plastic deformation regime.

Supplementary Figure 7

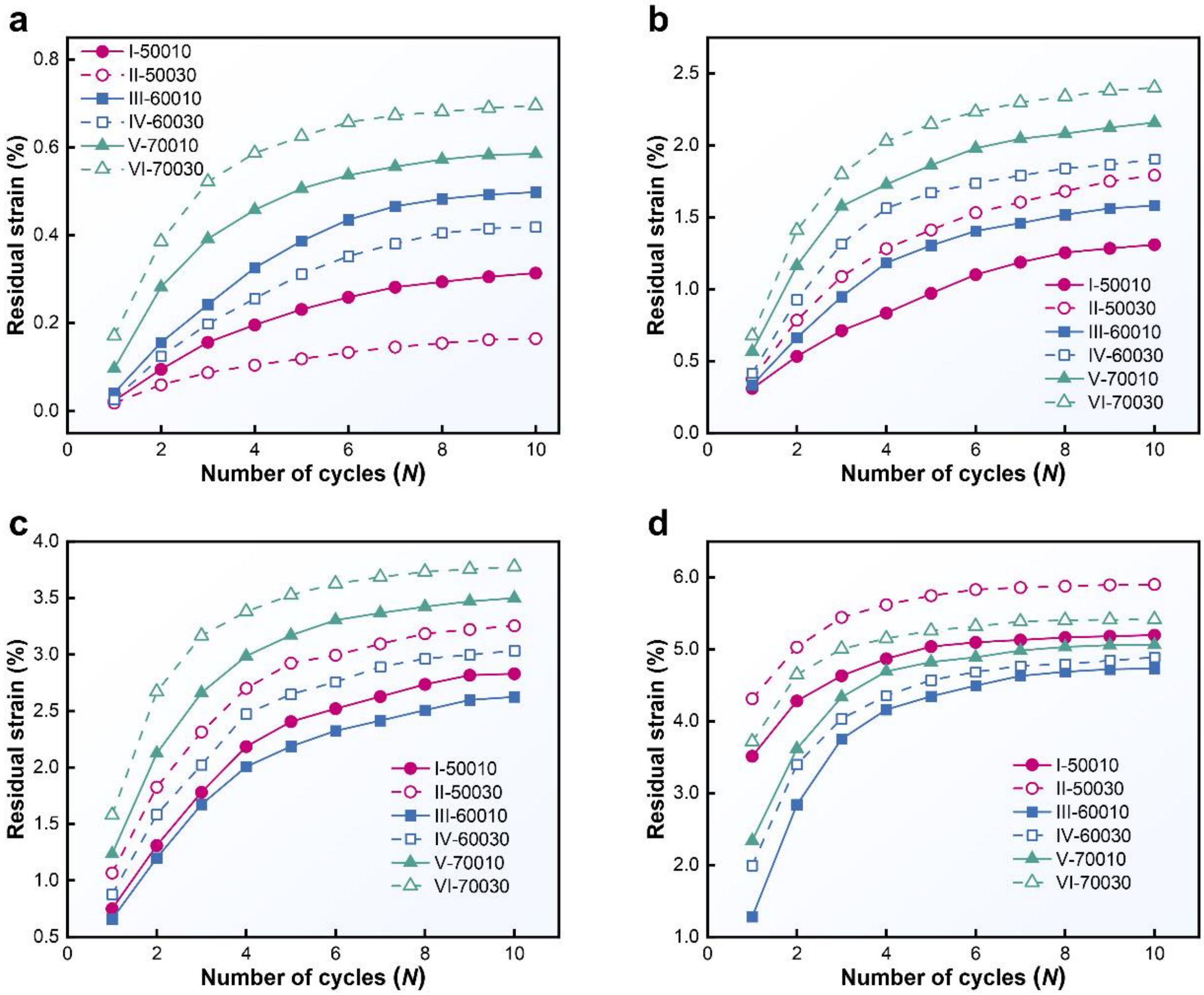

Supplementary Fig. 7 | Residual strain accumulation of NiTi alloys under various annealing conditions at a strain rate of 10^{-3} s^{-1}. Evolution of residual strain with increasing cycle number at strain amplitudes of (**a**) 2%, (**b**) 4%, (**c**) 6%, (**d**) 8%.

Supplementary Figure 8

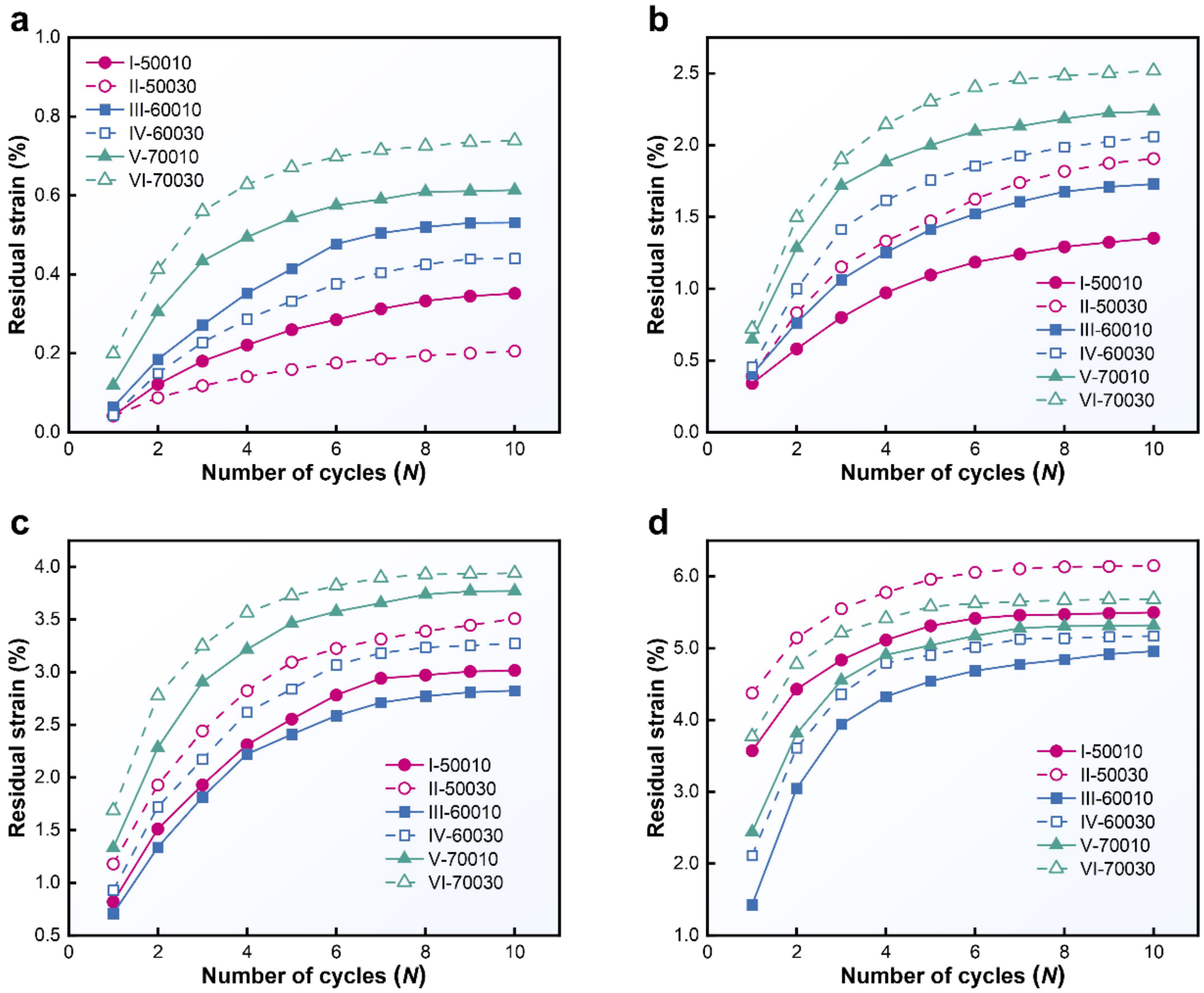

Supplementary Fig. 8 | Residual strain accumulation of NiTi alloys under various annealing conditions at a strain rate of 10^{-2} s^{-1}. Evolution of residual strain with increasing cycle number at strain amplitudes of (**a**) 2%, (**b**) 4%, (**c**) 6%, (**d**) 8%.

Supplementary Figure 9

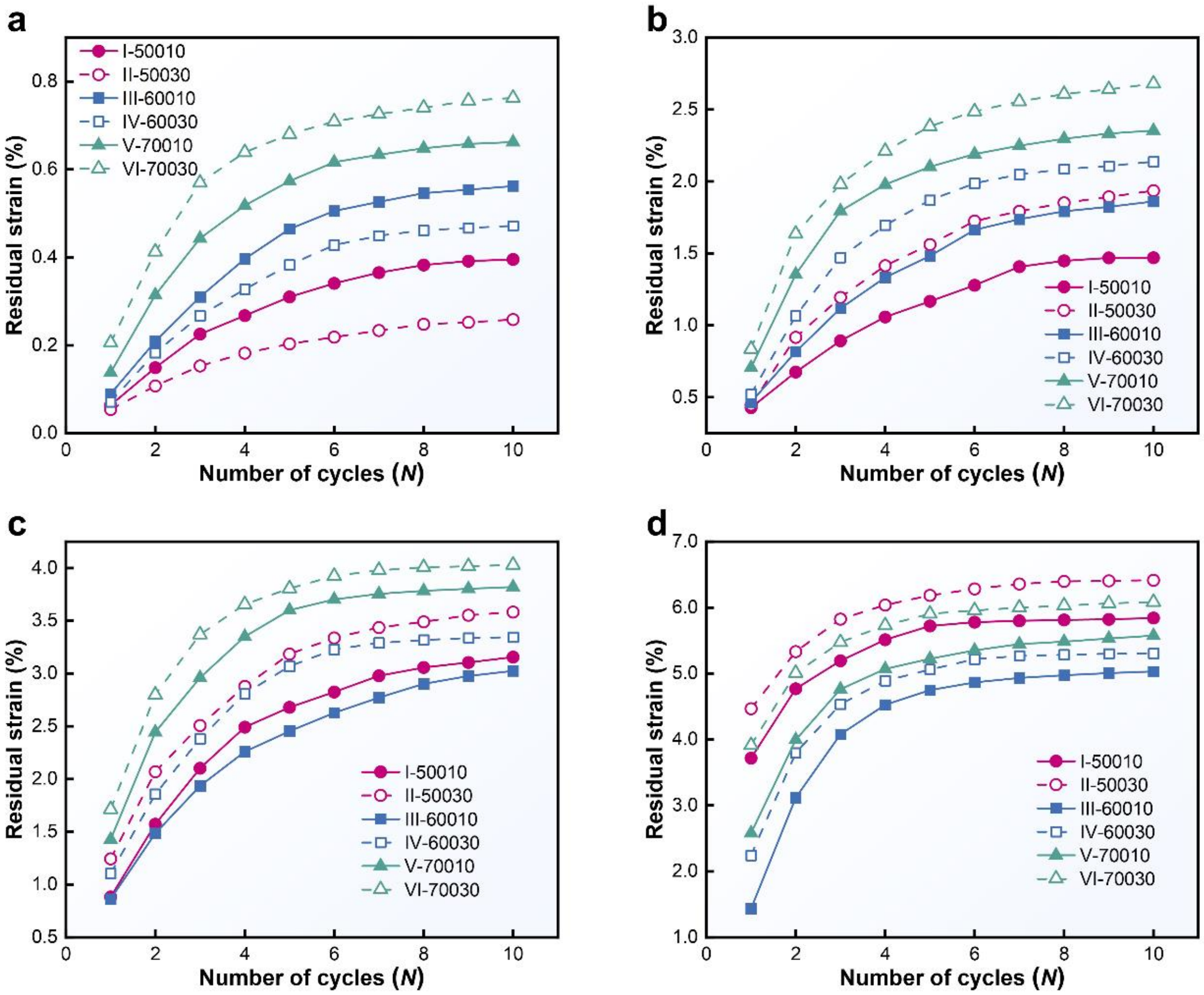

Supplementary Fig. 9 | Residual strain accumulation of NiTi alloys under various annealing conditions at a strain rate of 3 × 10^{-2} s^{-1}. Evolution of residual strain with increasing cycle number at strain amplitudes of (**a**) 2%, (**b**) 4%, (**c**) 6%, (**d**) 8%.

Supplementary Figure 10

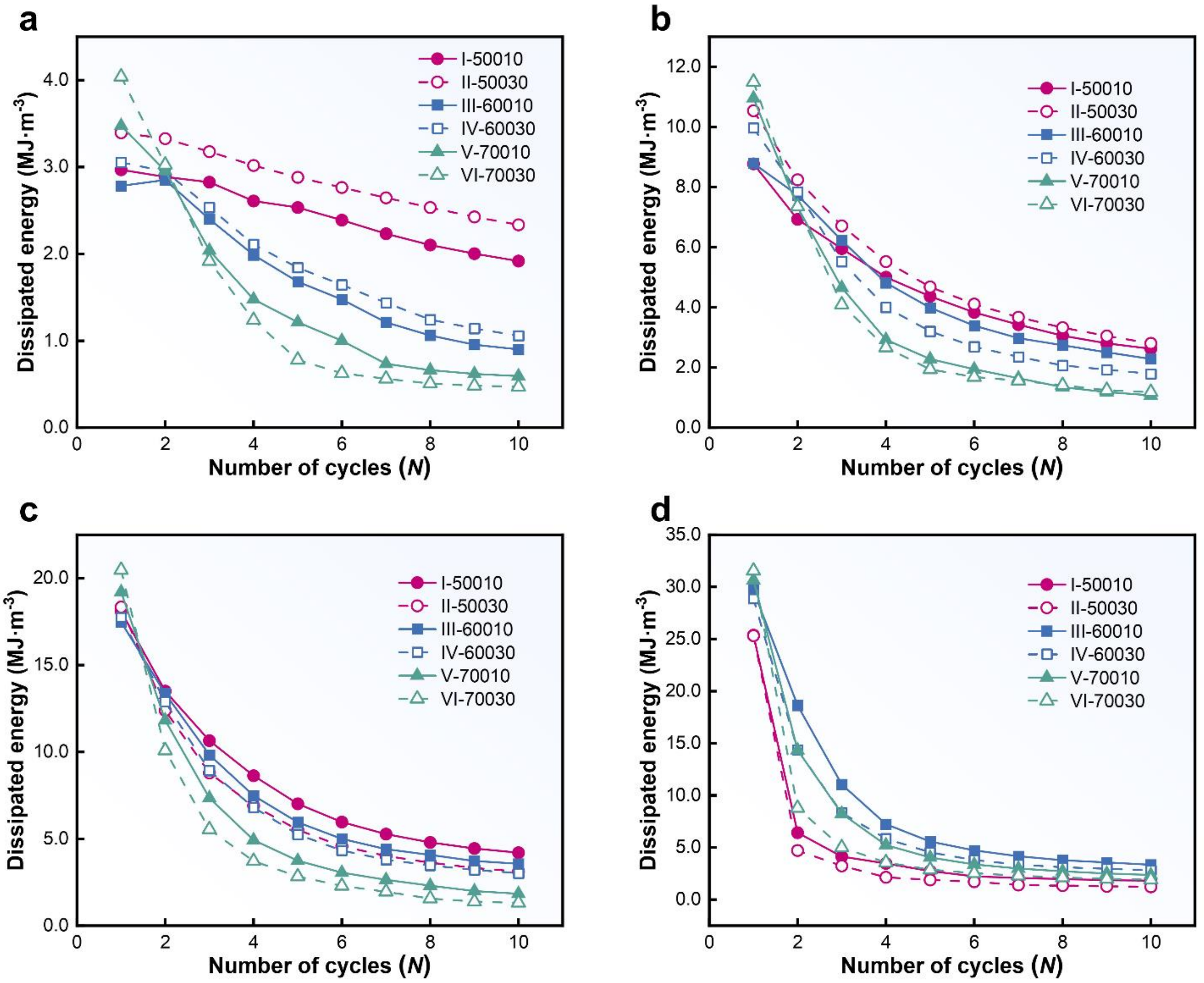

Supplementary Fig. 10 | Dissipated energy density of NiTi alloys under various annealing conditions. Evolution of dissipated energy density with increasing cycle number at strain amplitudes of (**a**) 2%, (**b**) 4%, (**c**) 6%, (**d**) 8%. These measurements were conducted under room-temperature quasi-static conditions.

Supplementary Figure 11

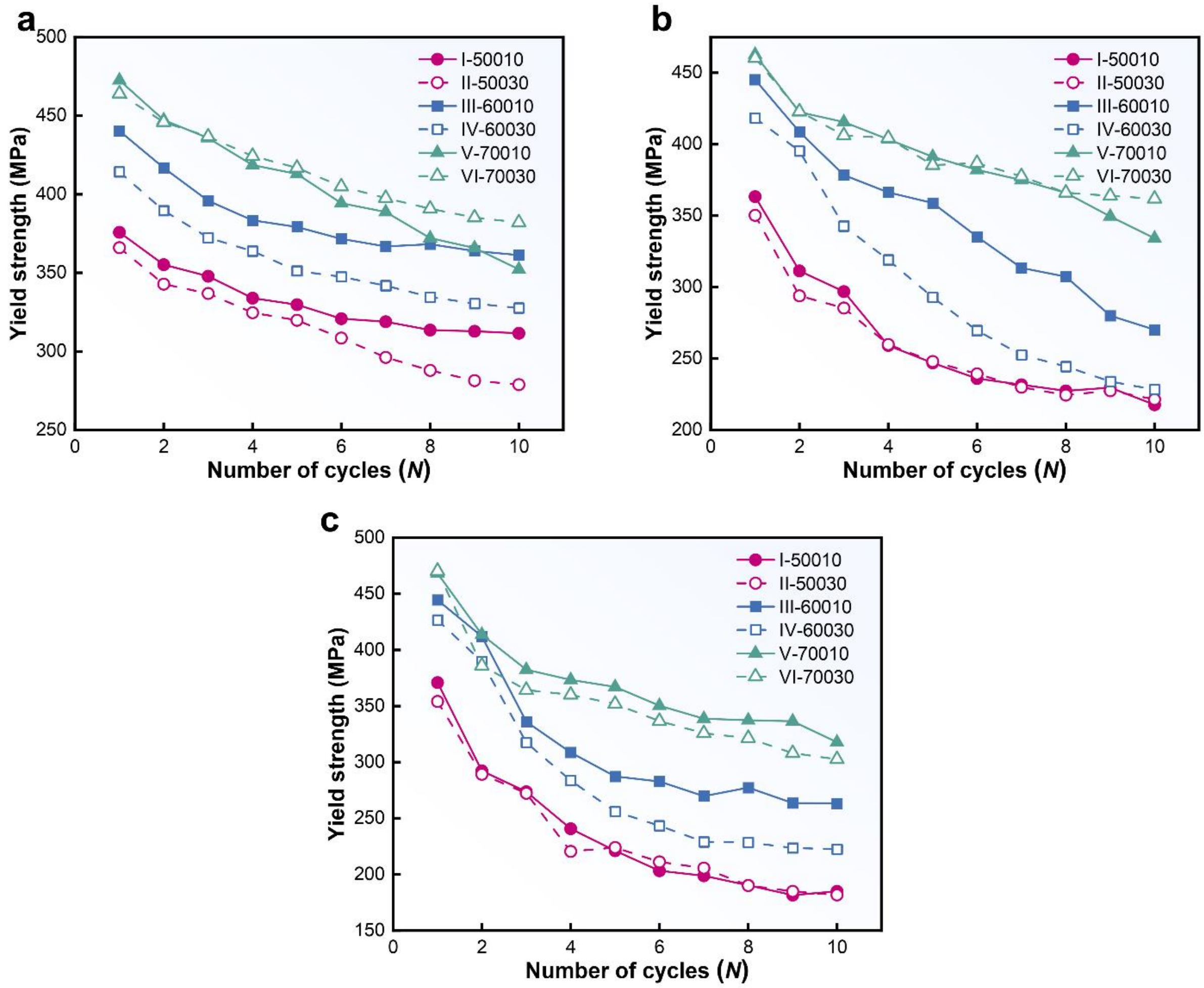

Supplementary Fig. 11 | Forward transformation start stress of NiTi alloys under various annealing conditions. Evolution of transformation stress with increasing cycle number at strain amplitudes of (**a**) 2%, (**b**) 4%, (**c**) 6%. These measurements were conducted under room-temperature quasi-static conditions.

Supplementary Table 1

Supplementary Table 1 | Transformation stress of NiTi specimens (I–VI) during the first loading cycle at a strain amplitude of 8%

Specimen	I	II	III	IV	V	VI
Transformation stress (MPa)	374.36	360.76	457.68	415.48	491.24	453.41

Supplementary Table 2

Supplementary Table 2 | The mean angular deviation (MAD) and indexing rates of EBSD scans under different annealing conditions. All EBSD scans were acquired with a step size of 0.2 μm. Values are reported as mean and standard deviation from three independently scanned regions for each annealing condition

Condition	MAD (°)	SD (°)	Indexing (%)	SD (%)
Specimen I	0.62	0.18	82.2	2.8
Specimen II	0.58	0.15	84.7	2.1
Specimen III	0.49	0.17	89.3	2.4
Specimen IV	0.45	0.13	91.6	1.9
Specimen V	0.38	0.11	94.4	1.6
Specimen VI	0.35	0.12	96.1	1.5

Supplementary Figure 12

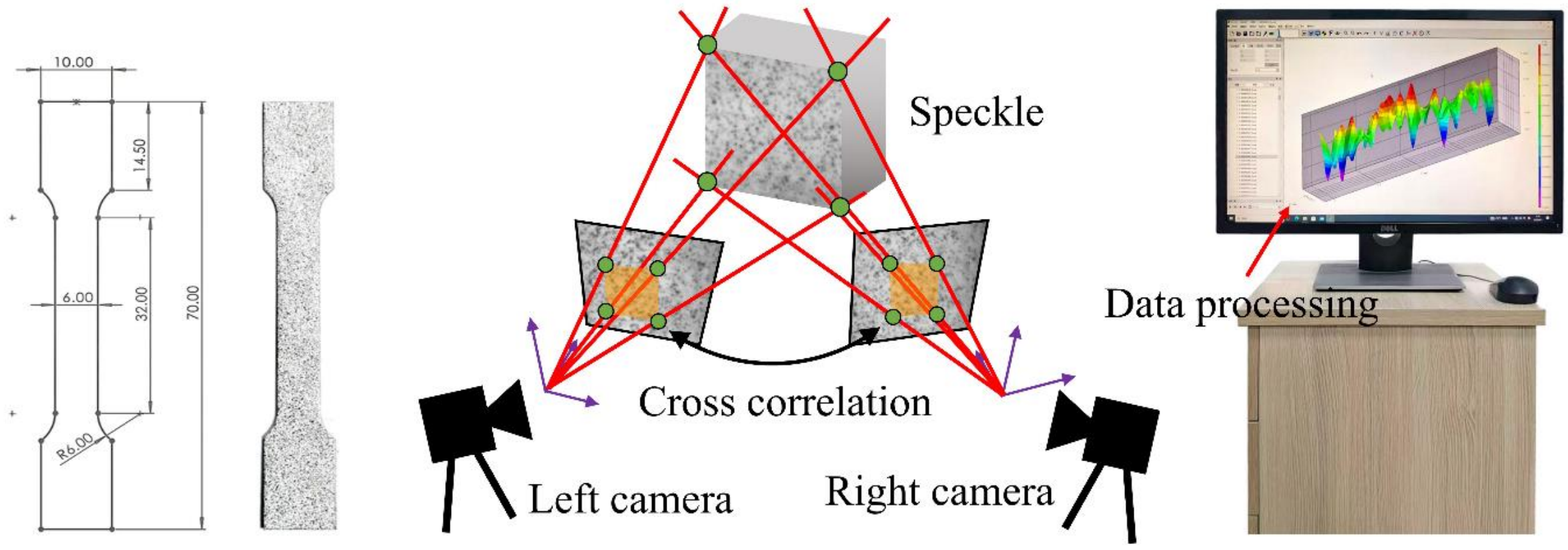

Supplementary Fig. 12 | Schematic of the digital image correlation (DIC) setup used to measure full-field strain during cyclic tensile loading. Dog-bone specimens with a gauge section of 25 mm × 6 mm were sprayed with a random speckle pattern and recorded by a stereo DIC system comprising two 5-megapixel CCD industrial cameras equipped with 35-mm lenses. Image pairs were continuously acquired and processed to obtain the three-dimensional displacement and strain fields. The target strain amplitudes for the subsequent cyclic tensile loading–unloading tests were first calibrated from monotonic uniaxial tensile experiments using the same DIC system.

Supplementary Figure 13

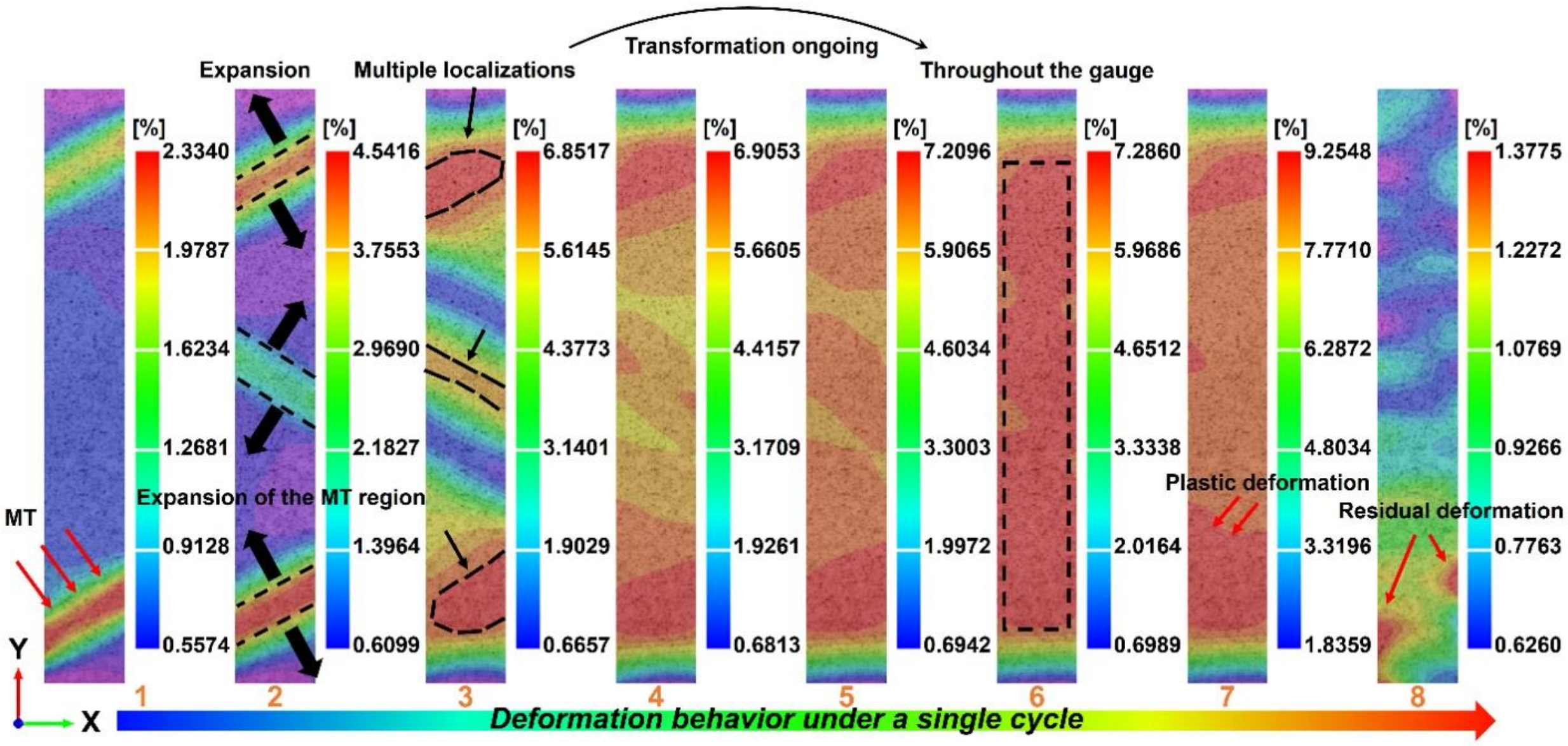

Supplementary Fig. 13 | First cycle deformation analysis. DIC maps 1–8 mark key deformation stages and correspond to the symbols on the stress-strain curve in Fig. 5b. At 2% strain, the specimen has entered the stress plateau and the initial martensitic transformation is activated. The transformation evolves from local nucleation to multiple localizations and eventually to a fully transformed gauge section. Notably, the end of the plateau at 6.66% strain does not coincide with the completion of transformation: by 6.82% the gauge section is fully transformed, and at 8% strain martensitic plastic deformation emerges.

Supplementary Figure 14

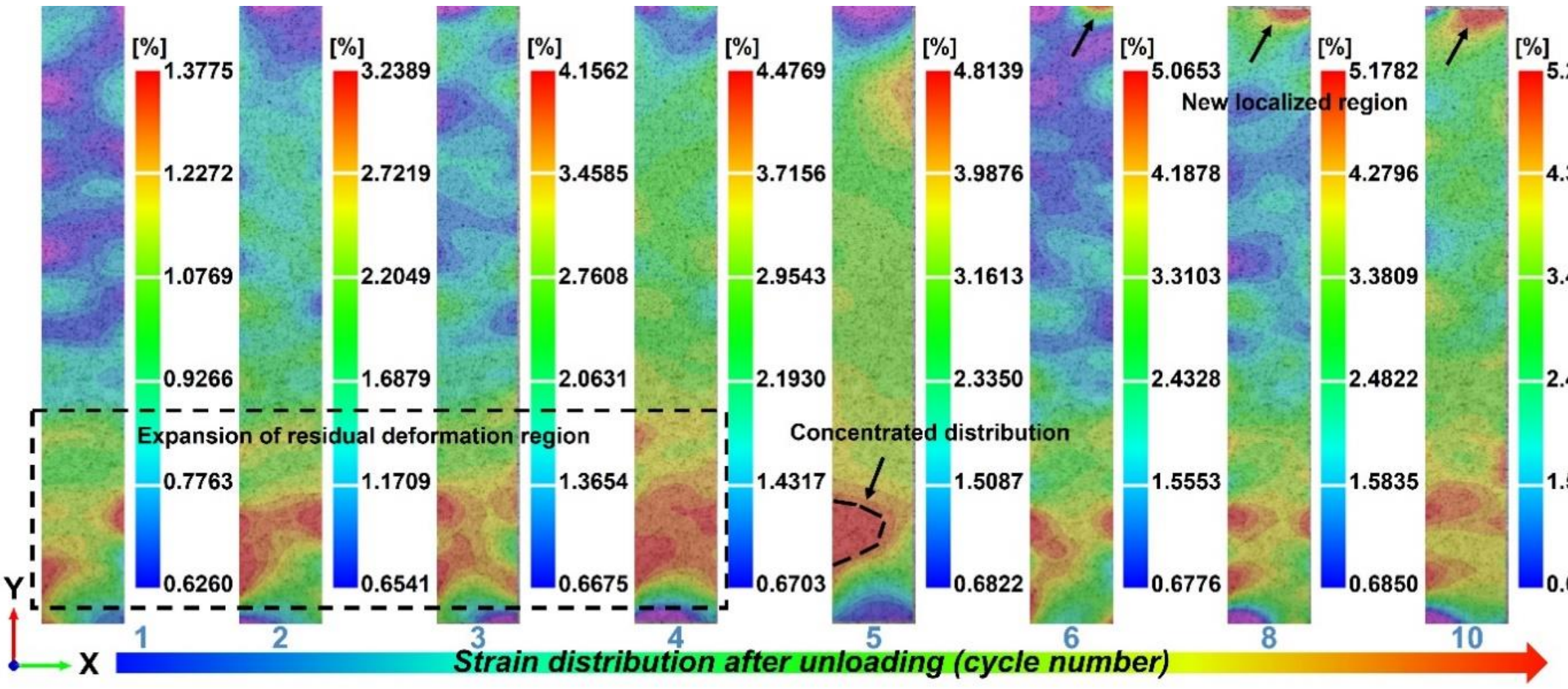

Supplementary Fig. 14 | Cyclic deformation analysis. DIC maps for successive cycles show the local deformation of the specimen at the end of each loading-unloading loop, corresponding to the blue markers on the stress-strain curve in Fig. 5b and visualizing the progressive accumulation of residual strain. As shown in the figure, the residual deformation region continuously expands during the first five loading cycles, which corresponds to the observed orientation-dependent variant transfer phenomenon reported in the main text.

Supplementary Figure 15

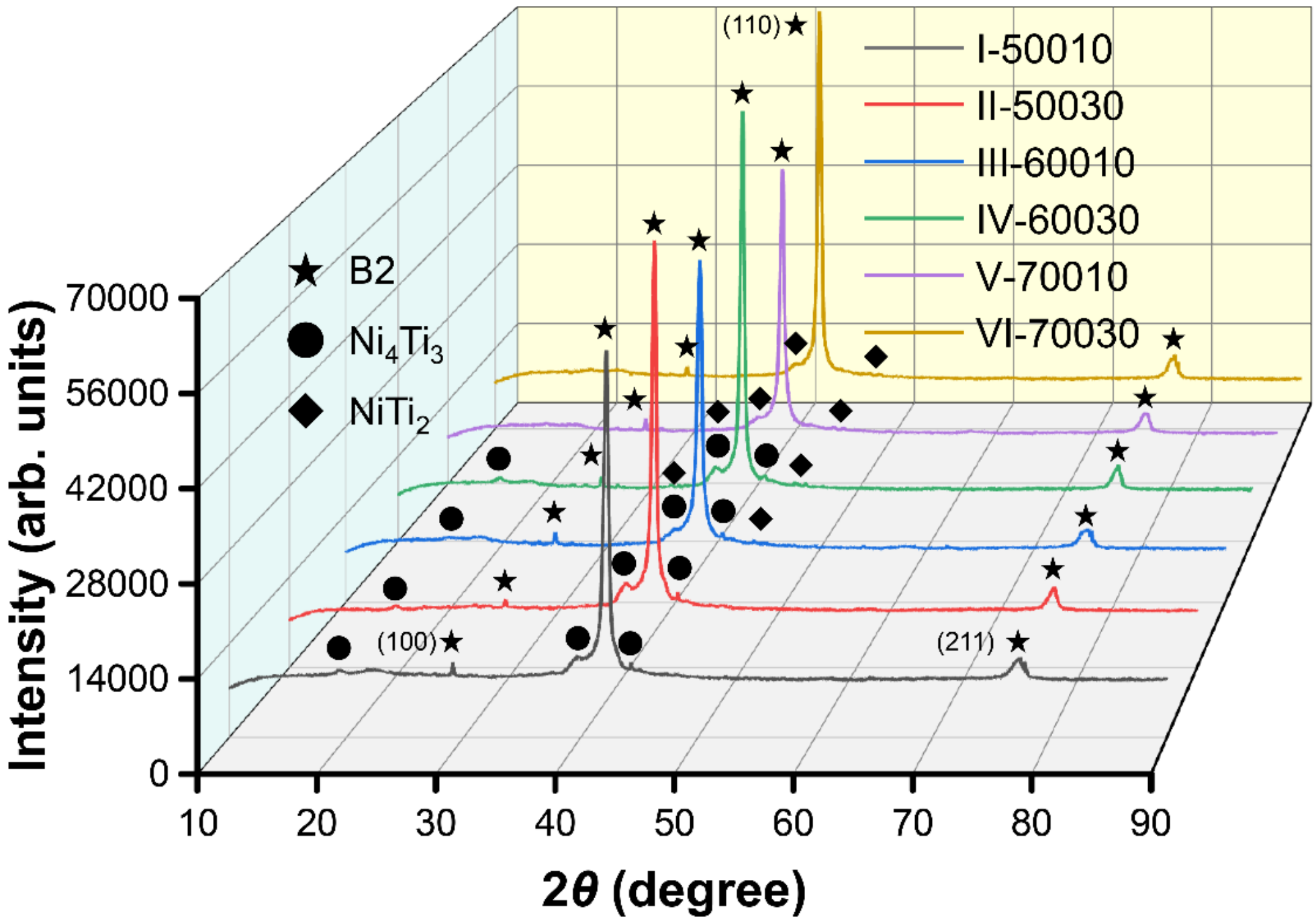

Supplementary Fig. 15 | XRD patterns of NiTi alloys annealed under various short-term annealing conditions. Specimens I–VI correspond to distinct annealing regimes: I–50010 (500 °C, 10 min), II–50030 (500 °C, 30 min), III–60010 (600 °C, 10 min), IV–60030 (600 °C, 30 min), V–70010 (700 °C, 10 min), and VI–70030 (700 °C, 30 min). These six conditions were selected after a preliminary screening of a broader range of annealing parameters, considering their overall mechanical and functional responses (e.g., tensile strength and cyclic stability). They represent distinct microstructural states involving variations in precipitate characteristics, while all specimens retain a B2 austenitic matrix at room temperature. With increasing annealing temperature, fine Ni_4Ti_3 precipitates dominate at 500 °C; a multiphase microstructure containing both Ni_4Ti_3 and $NiTi_2$ is observed at 600 °C, whereas $NiTi_2$ becomes the prevailing phase at 700 °C. Prolonging the holding time mainly promotes the coarsening and redistribution of precipitates without altering their types.

Supplementary Figure 16

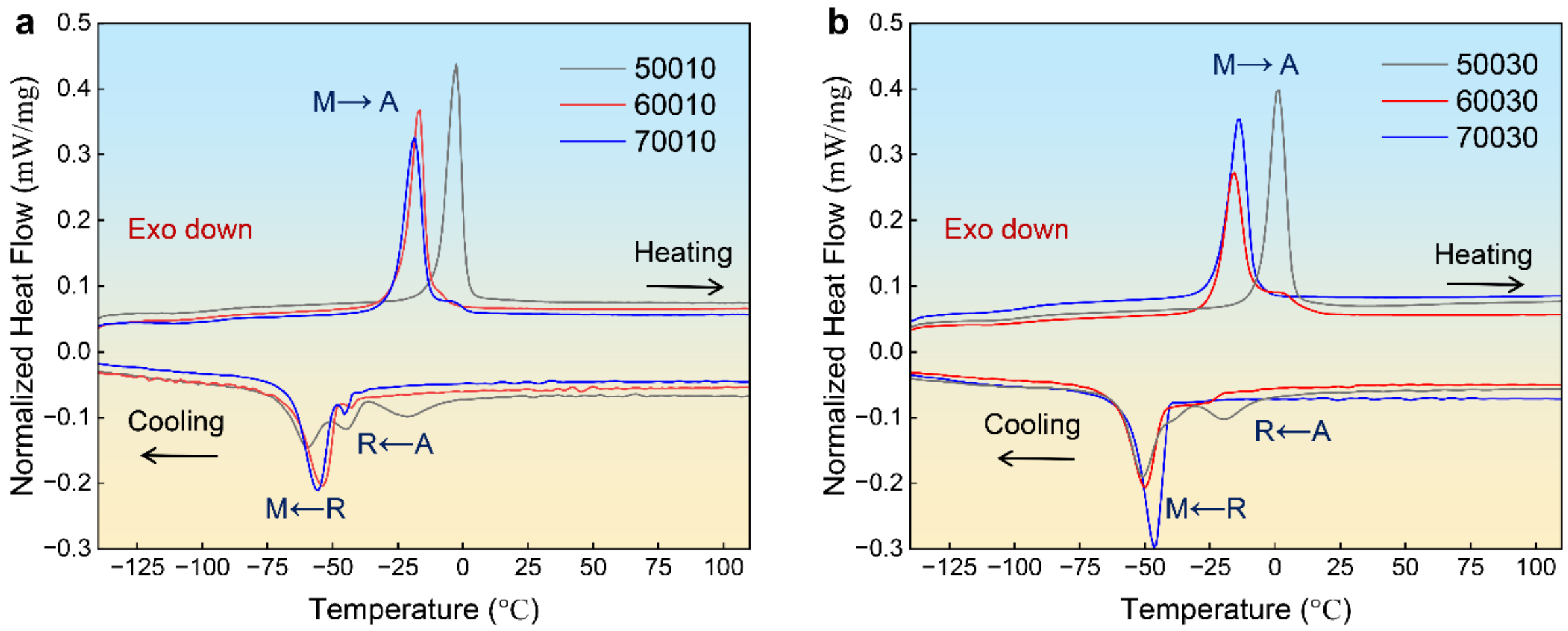

Supplementary Fig. 16 | DSC characterization of phase transformation behavior. a and **b** show the DSC curves for samples I, III, V and II, IV, VI, respectively. All samples remain in B2 austenite at room temperature, with the austenite finish temperatures (A_f) ranging from -13.77 °C to 5.43 °C. During heating, no distinct or well-separated double endothermic peaks are observed, indicating that the reverse transformation predominantly follows a single-step pathway. During cooling, samples annealed at 500 °C exhibit clearly separated dual exothermic peaks, characteristic of the B2→R→B19′ two-step transformation. This behavior is attributed to local internal stress fields introduced by Ni_4Ti_3 precipitates and their modulation of the local Ni concentration in the matrix[1]. With annealing temperatures increasing to 600 °C and 700 °C, the double-peak feature gradually weakens and tends to merge, indicating a transition toward a single-step B2→B19′ transformation. This evolution is associated with precipitate coarsening and redistribution, leading to reduced matrix-precipitate coherency, together with residual stress relaxation during high-temperature annealing, which collectively diminish the driving force for R phase formation.

Supplementary Table 3

Supplementary Table 3 | Transformation temperatures of NiTi alloys under different annealing conditions. A_s and A_f denote the start and finish temperatures of the austenite transformation during heating, while M_s and M_f represent those of the martensitic transformation during cooling

Condition	A_s (℃)	A_f (℃)	M_s (℃)	M_f (℃)
Specimen I	-9.460	1.030	-38.55	-69.74
Specimen II	-4.470	5.430	-45.15	-58.43
Specimen III	-23.57	-13.00	-48.56	-65.91
Specimen IV	-22.67	-10.61	-44.07	-59.28
Specimen V	-26.12	-13.77	-43.51	-65.00
Specimen VI	-21.80	-9.710	-42.36	-54.33

Supplementary Note 1

Electron backscatter diffraction: The EBSD results from Specimen IV are presented as a representative example. The microstructure exhibits a multiphase configuration comprising the B2 matrix (yellow) together with uniformly dispersed Ni_4Ti_3 (red) and $NiTi_2$ (blue) precipitates. Due to cold-rolling deformation prior to annealing, residual internal stresses were not fully relieved, resulting in a pronounced {101} texture. This microstructural state, characterized by a certain density of residual dislocations and refined grain structures, is instrumental in strengthening the matrix against plastic slip during stress-induced martensitic transformation, thereby enhancing superelastic stability[2,3]. Nevertheless, the overall structure remains predominantly uniform, with equiaxed grains averaging approximately 10.3 μm. In the phase image, $NiTi_2$ precipitates are generally identifiable, while Ni_4Ti_3 can only be effectively resolved by EBSD when sufficiently large (>200 nm). EBSD struggles to fully analyze smaller Ni_4Ti_3 precipitates. This is why we incorporated TEM results into the numerical feature to achieve a more comprehensive microstructural characterization.

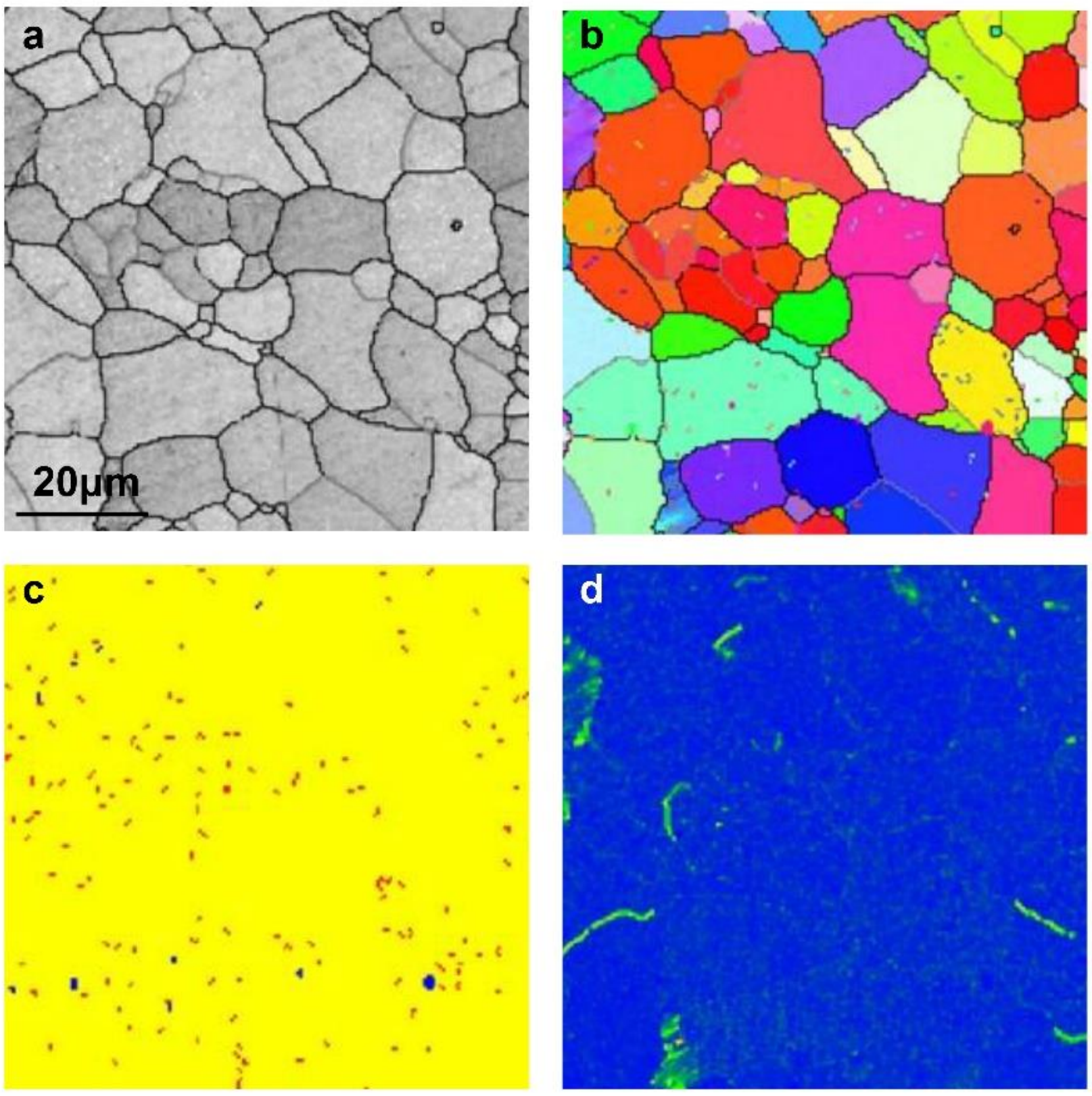

Supplementary Fig. 17 | EBSD characterization of specimen IV annealed at 600 °C for 30 min. a Band contrast image. **b** Inverse pole figure (IPF) map showing an equiaxed grain structure. **c** Phase map indicating that the matrix is primarily B2 austenite. **d** GND map revealing the spatial distribution of geometrically necessary dislocation density and local lattice curvature.

Supplementary Figure 18

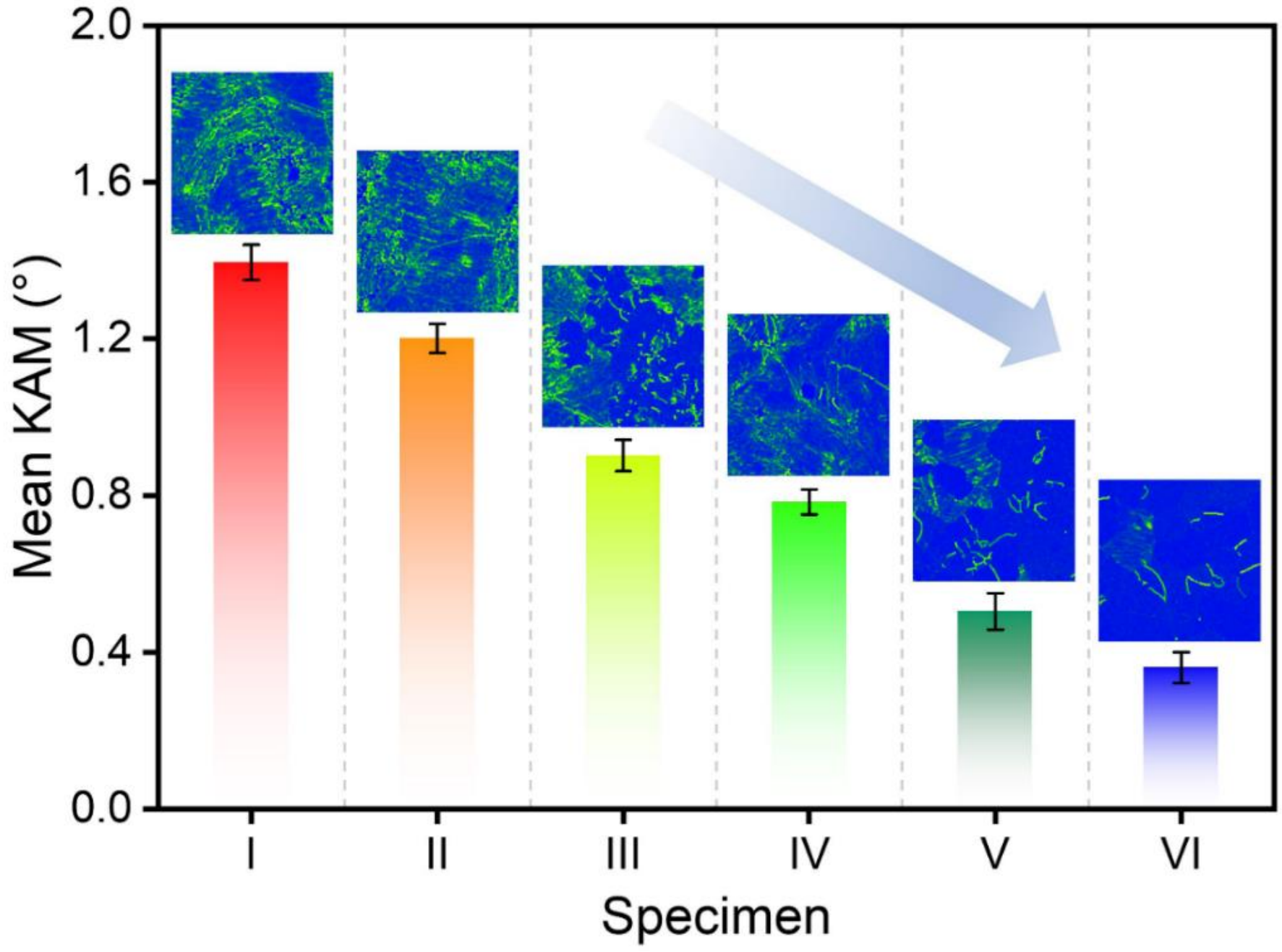

Supplementary Fig. 18 | Mean KAM values and representative KAM maps for specimens I–VI under different annealing conditions. Bars and error bars denote mean ± SD from $n = 3$ EBSD scan regions for each specimen. From specimens I to VI, the mean KAM decreases progressively from 1.40° to 0.36°, indicating a gradual reduction in local lattice distortion. This trend is generally associated with a decrease in geometrically necessary dislocation density during annealing, accompanied by the reduction of high-KAM regions.

Supplementary Figure 19

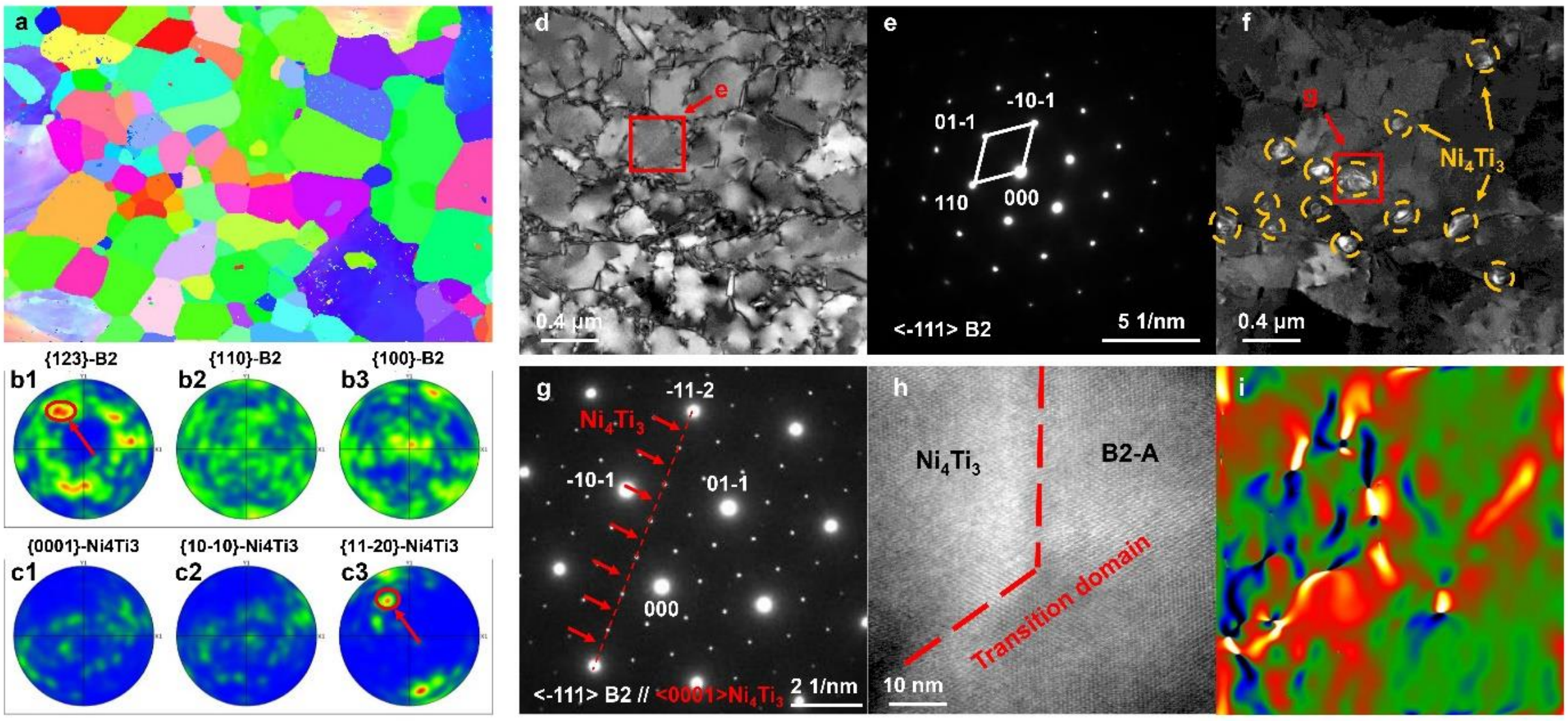

Supplementary Fig. 19 | Orientation relationship between Ni_4Ti_3 precipitates and the B2 austenite matrix. a Orientation distribution map. **b_1–b_3** {123}, {110}, and {100} pole figures of the B2 austenite matrix. **c_1–c_3** {0001}, {10-10}, and {11-20} pole figures of the Ni_4Ti_3 precipitates. **d** Bright-field TEM image of the overall region, and (**e**) the selected area electron diffraction (SAED) pattern taken from the red boxed area in (**d**). **f** Corresponding dark-field image, with (**g**) and (**h**) showing the SAED pattern and high-resolution TEM (HRTEM) image from the red boxed region in (**f**), respectively. **i** Geometric phase analysis[4] (GPA) strain map corresponding to (**h**). No common crystallographic plane was identified between Ni_4Ti_3 and the B2 matrix from the pole figures. Nevertheless, the pole figure and SAED analyses reveal a specific orientation relationship of $\{123\}\langle 111\rangle_{B2}$ // $\{11\text{-}20\}\langle 0001\rangle_{Ni_4Ti_3}$. The dark-field image demonstrates that precipitates are uniformly distributed as nanoscale particles. HRTEM and GPA strain mapping indicate that the formation of Ni_4Ti_3 precipitates induces strain inhomogeneity in the NiTi matrix, with significant local tensile strain in regions of continuous distribution.

Supplementary Figure 20

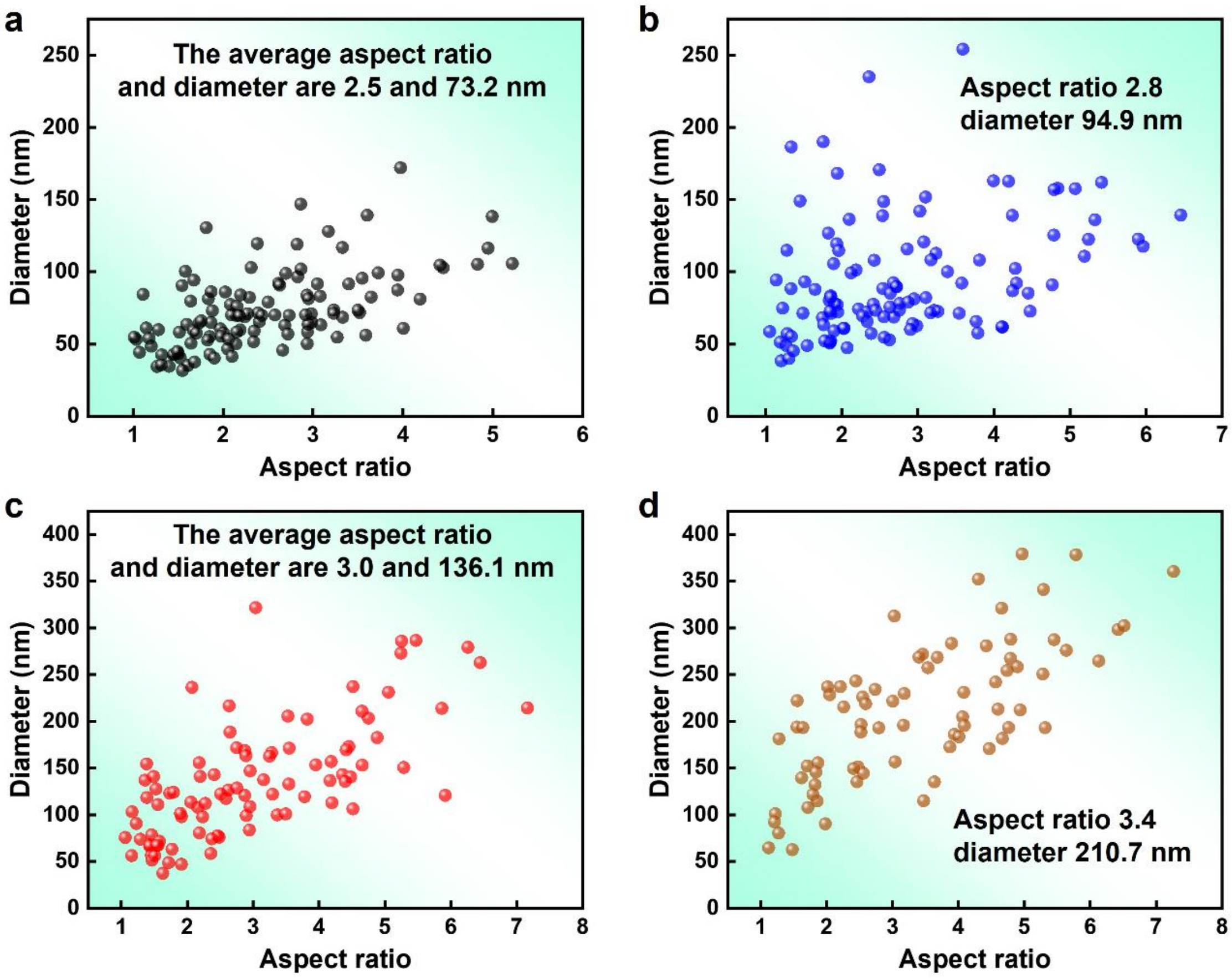

Supplementary Fig. 20 | Quantitative statistics of Ni_4Ti_3 precipitates under different annealing conditions. a–d Aspect ratio–diameter distributions of Ni_4Ti_3 precipitates in Specimens I–IV, corresponding to 500 °C × 10 min, 500 °C × 30 min, 600 °C × 10 min and 600 °C × 30 min, respectively. Each symbol denotes a single precipitate, and the insets show the mean aspect ratio and equivalent diameter. From Specimens I to IV, the data clouds shift toward larger diameters and higher aspect ratios, and the distributions gradually broaden, indicating progressive coarsening and slight elongation of Ni_4Ti_3. The increase in average diameter and aspect ratio is much more pronounced when the annealing temperature rises from 500 °C to 600 °C than when the holding time is extended from 10 min to 30 min, indicating that temperature has a stronger influence on the precipitation behavior than time.

Supplementary Method 1

Construction of the Ni_4Ti_3 precipitate descriptor by principal component analysis: To encode the precipitation state as a single numerical input while avoiding redundancy among correlated TEM statistics, we reduced the Ni_4Ti_3 measurements to a one-dimensional descriptor using principal component analysis (PCA)[5,6]. TEM observations were first summarized into statistical features describing diameter, aspect ratio, and projected area, followed by PCA performed on the correlation matrix of the dataset. The explained variance ratio of component *k* was calculated as $\eta_k = \lambda_k / \sum_j \lambda_j$, where λ_k is the eigenvalue associated with principal component *k*. The first principal component (PC1) captured the dominant covariance of precipitate evolution and explained 83.45% of the total variance, whereas PC2 and PC3 accounted for 15.23% and 1.32%, respectively. Consequently, the one-component representation retained 83.45% of the original variance while discarding 16.55%. The corresponding loading matrix is provided in Supplementary Table 4.

The precipitate descriptor used in the numerical branch was defined as the PC1 score. Given the arbitrary sign of PCA eigenvectors, PC1 was normalized using a sigmoid function such that a larger value corresponds to a coarser precipitation state. For the conditions in which no precipitates were observed, a small constant value of 0.01 was assigned to avoid zero-valued inputs that may induce numerical instability during network training. This dimensionality reduction preserves the essential microstructural information while preventing overweighting of mutually correlated precipitate features in the numerical branch. Consistently, the unreduced precipitate statistics lead to inferior predictive performance (Supplementary Table 10).

Supplementary Table 4 | PCA loading matrix and explained variance for the Ni_4Ti_3 precipitate descriptor

Principal component	Diameter loading	Aspect ratio loading	Projected area loading	Explained variance ratio
PC1	0.5893	0.5212	0.6173	0.8345
PC2	-0.5008	0.8352	-0.2271	0.1523
PC3	-0.6340	-0.1753	0.7532	0.0132

Supplementary Method 2

Calculation and correction of the Schmid factor: For multiphase NiTi alloys, traditional Schmid theory struggles to accurately predict critical transformation stresses. The primary limitation arises from the localized stress field introduced by precipitates, which modifies the effective resolved shear stress distribution within grains. As a result, the actual slip and transformation behaviors depart from idealized crystallographic expectations. Previous studies indicate that the coherency mismatch stress of Ni_4Ti_3 precipitates can elevate the critical stress for dislocation slip while simultaneously lowering the onset stress required for martensitic transformation[1,7,8]. With Ni_4Ti_3 precipitates distributed nearly uniformly and the analysis centered on preferentially oriented grains that govern superelastic degradation, the use of this correction is well-justified and broadly applicable. In principle, the Ni_4Ti_3 precipitates larger than ~300 nm, once they lose coherency with the matrix, should contribute only weakly to the internal stress field[9], similar to $NiTi_2$ particles. However, TEM observations (Supplementary Fig. 20) show that such oversized Ni_4Ti_3 particles are extremely scarce, so the overall adjustment is still formulated by treating the Ni_4Ti_3 population as effectively coherent. The specific derivation is as follows. We first assume that dislocation slip or SIMT is triggered when the total resolved shear stress attains a critical threshold τ_{c}, such that:

$$\tau_{\text{rss-total}} = m \cdot \sigma_{\text{app}} + \tau_{\text{rss-i}} \geq \tau_{c} \tag{1}$$

Where $\tau_{\text{rss-total}}$ denotes the total critical resolved shear stress. *m* represents the traditional Schmid factor, σ_{app} corresponds to the nominal stress under applied load, and $\tau_{\text{rss-i}}$ signifies the local resolved shear stress introduced by the coherent stress field of the Ni_4Ti_3 precipitates. To analyze the activation behavior in different regions, the above relationship can be equivalently expressed as a modified Schmid factor:

$$m_{\text{eff}} \cdot \sigma_{\text{app}} = m \cdot \sigma_{\text{app}} + \tau_{\text{rss-i}} \tag{2}$$

$$m_{\text{eff}} = m + \frac{\tau_{\text{rss-i}}}{\sigma_{\text{app}}} \tag{3}$$

Here, m_{eff} denotes the corrected Schmid factor, which integrates the combined effects of the external load and the local coherency stress field. For different deformation mechanisms, the corrected form can be expressed as follows:

$$\begin{cases} m_{\text{eff,slip}} = m_{\text{slip}} - \dfrac{\tau_{\text{rss-i,slip}}}{\sigma_{\text{app,slip}}} \\ m_{\text{eff,trans}} = m_{\text{trans}} - \dfrac{\tau_{\text{rss-i,trans}}}{\sigma_{\text{app,trans}}} \end{cases} \tag{4}$$

Among these, m_{slip} and m_{trans} represent the conventional Schmid factors for slip and transformation, respectively. $\tau_{\text{rss-i,slip}}$ and $\tau_{\text{rss-i,trans}}$ denote the local shear stress corrections for the precipitates, with the former corresponding to the slip-inhibition effect and the latter to the transformation-promotion effect. $\sigma_{\text{app,slip}}$ and $\sigma_{\text{app,trans}}$ represent the applied stresses, respectively. For transformation, $\sigma_{\text{app,trans}}$ is taken as the stress corresponding to the first tensile transformation plateau. To quantify the local stress fields generated by precipitates, the Eshelby approach is employed:

$$\tau_{\text{rss-i,trans}} = \alpha'_{ij} C_{ijkl} D_{klmn}(x) \varepsilon^{\text{p}}_{mn} \tag{5}$$

Here, α'_{ij} is the orientation tensor transformed into the principal coordinate system of the precipitate from α_{ij} :

$$\alpha_{ij} = \frac{1}{2}(m_i n_j + n_i m_j) \tag{6}$$

m and *n* denote the shear direction and the habit plane normal associated with each martensitic twin variant. Each martensite twin variant pair comprises two martensite single-crystal variants V*i* and V*j* connected by a twin relationship, maintaining a specific volume fraction ratio *f*:(1-*f*). Although the interface between the austenite and each individual martensite variant (V*i* or V*j*) is not an invariant plane, the interface between the austenite and the martensite variant pair (V*i*–V*j*) is an invariant habit plane. In this study, we consider the only two types of martensitic twins with invariant habit planes: the <011> type II twin and the {11-1} type I twin, yielding a total of 48 variant pairs[10,11]. For the first type, the crystallographic parameters are **m** = [0.41145, −0.76331, 0.49807] and **n** = [−0.88888, −0.21524, 0.40443]; for the second type, **m** = [−0.82044, 0.02068, 0.57136] and **n** = [0.27789, −0.85894, 0.43012]. The parameters for the other 46 variants can be obtained through cyclic permutation of the indices. Supplementary Table 5 and Table 6 summarize the theoretical solutions for all habit planes of these two twin types.

It should be clarified that Ni_4Ti_3 precipitates follow a specific orientation relationship with the B2 matrix, as verified by the pole figure and SAED analyses (Supplementary Fig. 19). Accordingly, although multiple symmetry-related precipitate variants may exist, the precipitate principal coordinate system in the specimen frame is determined from the local B2 orientation together with this orientation relationship[12]. In the present implementation, the precipitate-induced internal stress correction is evaluated at each EBSD pixel using the corresponding matrix orientation and is then applied to each martensitic variant to obtain the corrected Schmid

factor. Since the explicit population of Ni_4Ti_3 variants is not resolved, the variant diversity is represented in terms of an effective contribution to the resolved shear stress.

Additionally, C_{ijkl} represents the elastic constant tensor for the precipitate and the surrounding matrix (assumed to be consistent). The tensor $D_{klmn}(x)$ is constructed from the Eshelby tensor for the stress fields exterior to the inhomogeneity. This tensor is a function of the position vector outside the precipitate, x, the geometry of the precipitate, and the elastic constants of the precipitate[13]. For an isotropic elastic medium, it is given by:

$$\begin{aligned} 8\pi(1-v)D_{klmn}(x) = \psi_{,mnkl}(x) - 2v\delta_{mn}\phi_{,kl}(x) - \\ (1-v)[\phi_{,kj}(x)\delta_{ln} + \phi_{,lm}(x)\delta_{kn} + \phi_{,kn}(x)\delta_{lm} + \phi_{,ln}(x)\delta_{km}] \end{aligned} \tag{7}$$

with the scalar potentials:

$$\psi(x) = \int_\Omega |x - x'| \mathrm{d}x', \ \phi(x) = \int_\Omega \frac{1}{|x - x'|} \mathrm{d}x' \tag{8}$$

Where Ω denotes the precipitate domain, and x' is the integration variable within Ω. v is the Poisson's ratio of the B2 matrix. δ_{mn} is the Kronecker delta, defined as $\delta_{mn} = 1$ when $m = n$ and $\delta_{mn} = 0$ otherwise. The comma notation denotes partial differentiation with respect to the components of the field point x. For example, $\psi_{,mnkl}(x) = \dfrac{\partial^4 \psi}{\partial x_m \partial x_n \partial x_k \partial x_l}$.

$\varepsilon^{\mathrm{p}}_{mn}$ represents the intrinsic lattice-misfit strain of the Ni_4Ti_3 precipitate, corresponding to the stress-free transformation strain arising from the crystallographic lattice correspondence between the B2 matrix and the rhombohedral Ni_4Ti_3 phase[9]. In the principal coordinate system of the precipitate (with the 3-axis aligned with its symmetry axis), the misfit strain tensor is approximated as a uniform diagonal form:

$$\varepsilon^{\mathrm{p}}_{mn} = \begin{bmatrix} \varepsilon^{\mathrm{p}}_{11} & 0 & 0 \\ 0 & \varepsilon^{\mathrm{p}}_{22} & 0 \\ 0 & 0 & \varepsilon^{\mathrm{p}}_{33} \end{bmatrix} = \begin{bmatrix} -4.17\times10^{-3} & 0 & 0 \\ 0 & -4.17\times10^{-3} & 0 \\ 0 & 0 & -2.57\times10^{-2} \end{bmatrix} \tag{9}$$

For slip-related correction, Eshelby's calculation method remains consistent, employing only the crystallographic parameters of the slip system in the orientation projection section. Specifically, in Equation (6), α_{ij} no longer represents the orientation tensor of the transformation variant, but instead denotes the Schmid orientation tensor $\alpha_{ij} = s_i n_j$, defined by the slip direction s_i and slip plane normal n_j. The most representative slip systems in B2

NiTi, {011}<001> and {011}<111>, are selected. Moreover, the $\sigma_{\mathrm{app,slip}}$ is the conventional yield stress[14], extracted from tensile tests conducted at 200 °C (Supplementary Fig. 21), a temperature at which no stress-induced transformation occurs and aging effects are still negligible.

Supplementary Table 5

Supplementary Table 5 | Theoretical solutions of the habit plane system for <011> type II twin. The volume fraction ratio of the twinning *f*:(1-*f*)=0.72898:0.27102

Variant pairs	Invariant habit plane	Invariant shear direction
1-2	(−0.88888 −0.21524 0.40443)	[0.41145 −0.76331 0.49807]
1-2′	(−0.88888 −0.40443 0.21524)	[0.41145 −0.49807 0.76331]
1′-2	(0.88888 −0.40443 0.21524)	[−0.41145 −0.49807 0.76331]
1′-2′	(0.88888 −0.21524 0.40443)	[−0.41145 −0.76331 0.49807]
2-1	(−0.88888 −0.21524 −0.40443)	[0.41145 −0.76331 −0.49807]
2-1′	(−0.88888 −0.40443 −0.21524)	[0.41145 −0.49807 −0.76331]
2′-1	(0.88888 −0.40443 −0.21524)	[−0.41145 −0.49807 −0.76331]
2′-1′	(0.88888 −0.21524 −0.40443)	[−0.41145 −0.76331 −0.49807]
3-4	(0.40443 −0.88888 −0.21524)	[0.49807 0.41145 −0.76331]
3-4′	(0.21524 −0.88888 −0.40443)	[0.76331 0.41145 −0.49807]
3′-4	(0.21524 0.88888 −0.40443)	[0.76331 −0.41145 −0.49807]
3′-4′	(0.40443 0.88888 −0.21524)	[0.49807 −0.41145 −0.76331]
4-3	(−0.40443 −0.88888 −0.21524)	[−0.49807 0.41145 −0.76331]
4-3′	(−0.21524 −0.88888 −0.40443)	[−0.76331 0.41145 −0.49807]
4′-3	(−0.21524 0.88888 −0.40443)	[−0.76331 −0.41145 −0.49807]
4′-3′	(−0.40443 0.88888 −0.21524)	[−0.49807 −0.41145 −0.76331]
5-6	(−0.21524 0.40443 −0.88888)	[−0.76331 0.49807 0.41145]
5-6′	(−0.40443 0.21524 −0.88888)	[−0.49807 0.76331 0.41145]
5′-6	(−0.40443 0.21524 0.88888)	[−0.49807 0.76331 −0.41145]
5′-6′	(−0.21524 0.40443 0.88888)	[−0.76331 0.49807 −0.41145]
6-5	(−0.21524 −0.40443 −0.88888)	[−0.76331 −0.49807 0.41145]
6-5′	(−0.40443 −0.21524 −0.88888)	[−0.49807 −0.76331 0.41145]
6′-5	(−0.40443 −0.21524 0.88888)	[−0.49807 −0.76331 −0.41145]
6′-5′	(−0.21524 −0.40443 0.88888)	[−0.76331 −0.49807 −0.41145]

Supplementary Table 6

Supplementary Table 6 | Theoretical solutions of the habit plane system for {11-1} type I twin. The volume fraction ratio of the twinning f:(1-f)=0.67994:0.32006

Variant pairs	Invariant habit plane	Invariant shear direction
1–4	(0.27789 −0.85894 0.43012)	[−0.82044 0.02068 0.57136]
1–5′	(0.27789 −0.43012 0.85894)	[−0.82044 −0.57136 −0.02068]
1′–3	(−0.27789 −0.85894 0.43012)	[0.82044 0.02068 0.57136]
1′–6	(−0.27789 −0.43012 0.85894)	[0.82044 −0.57136 −0.02068]
2–3′	(0.27789 −0.85894 −0.43012)	[−0.82044 0.02068 −0.57136]
2–5	(0.27789 −0.43012 −0.85894)	[−0.82044 −0.57136 0.02068]
2′–4′	(−0.27789 −0.85894 −0.43012)	[0.82044 0.02068 −0.57136]
2′–6′	(−0.27789 −0.43012 −0.85894)	[0.82044 −0.57136 0.02068]
3–1′	(0.85894 0.27789 −0.43012)	[−0.02068 −0.82044 −0.57136]
3–6	(0.43012 0.27789 −0.85894)	[0.57136 −0.82044 0.02068]
3′–2	(0.85894 −0.27789 −0.43012)	[−0.02068 0.82044 −0.57136]
3′–5	(0.43012 −0.27789 −0.85894)	[0.57136 0.82044 0.02068]
4–1	(−0.85894 0.27789 −0.43012)	[0.02068 −0.82044 −0.57136]
4–5′	(−0.43012 0.27789 −0.85894)	[−0.57136 −0.82044 0.02068]
4′–2′	(−0.85894 −0.27789 −0.43012)	[0.02068 0.82044 −0.57136]
4′–6′	(−0.43012 −0.27789 −0.85894)	[−0.57136 0.82044 0.02068]
5–2	(−0.85894 0.43012 0.27789)	[0.02068 0.57136 −0.82044]
5–3′	(−0.43012 0.85894 0.27789)	[−0.57136 −0.02068 −0.82044]
5′–1	(−0.85894 0.43012 −0.27789)	[0.02068 0.57136 0.82044]
5′–4	(−0.43012 0.85894 −0.27789)	[−0.57136 −0.02068 0.82044]
6–1′	(−0.85894 −0.43012 0.27789)	[0.02068 −0.57136 −0.82044]
6–3	(−0.43012 −0.85894 0.27789)	[−0.57136 0.02068 −0.82044]
6′–2′	(−0.85894 −0.43012 −0.27789)	[0.02068 −0.57136 0.82044]
6′–4′	(−0.43012 −0.85894 −0.27789)	[−0.57136 0.02068 0.82044]

Supplementary Figure 21

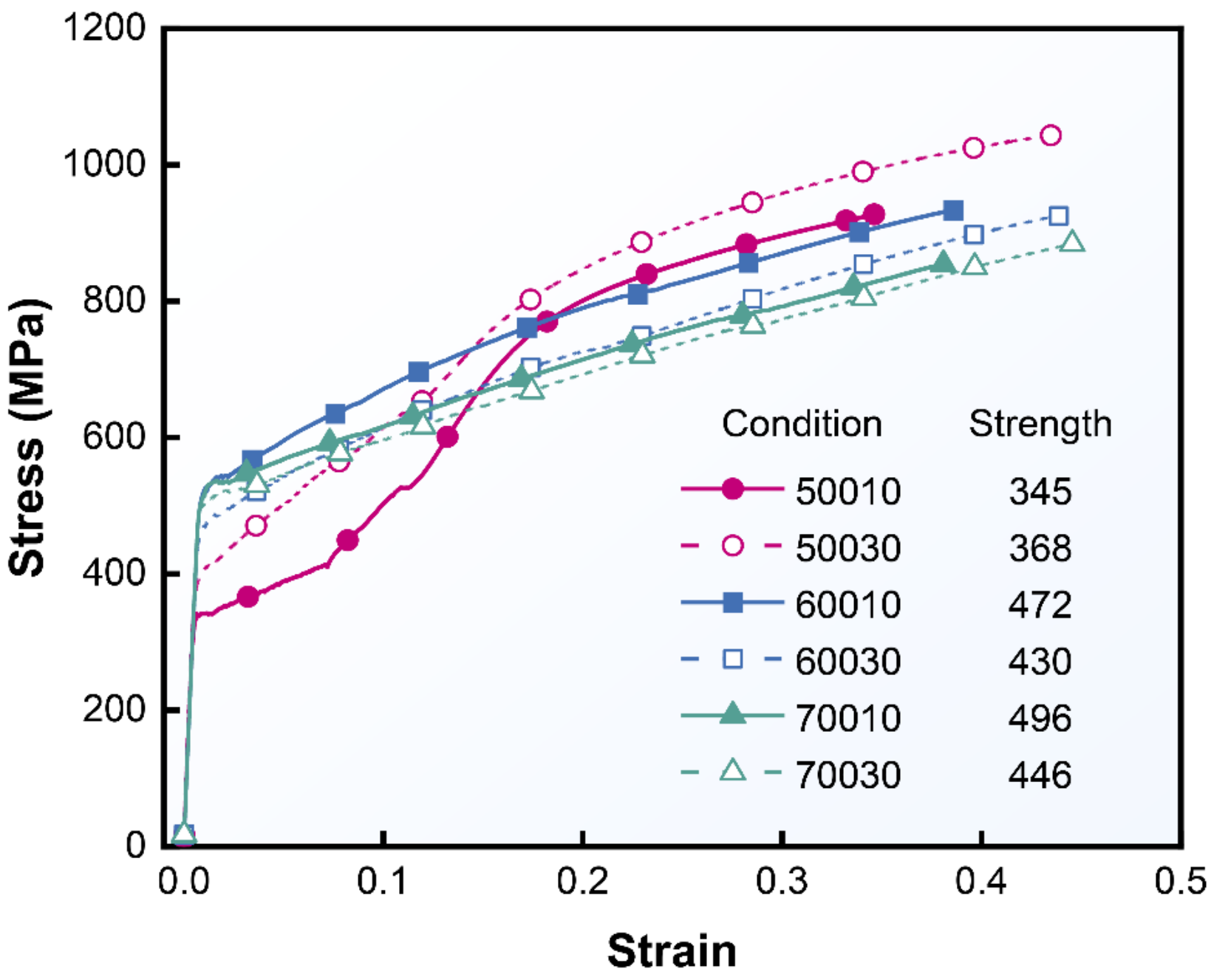

Supplementary Fig. 21 | Tensile testing at 200 °C is used to determine the critical stress $\sigma_{\text{app,slip}}$ for slip (conventional yield strength). At this temperature, no stress-induced transformation occurs and aging effects are still negligible.

Supplementary Table 7

Supplementary Table 7 | Research on the SED mechanism of NiTi alloy. Competition defines the analytical depth of the interplay between dislocation slip and martensitic transformation. Spatially resolved indicates whether the study reaches the spatially resolved level of microstructure or deformation

Reference	Method	Competition	Spatially resolved
Prog. Mater. Sci., 2005, 50, 511-678	Review	Conceptual	No
Acta Mater., 2000, 48, 3311–3326	Experiment	Model	No
Prog. Mater. Sci., 2017, 85, 1-42	Review	Conceptual	No
Prog. Mater. Sci., 2018, 98, 249-298	Review	Qualitative	No
Mater. Des., 2017, 134, 111-120	DIC	Qualitative	No
Int. J. Plast., 2015, 72, 60-90	Constitutive model	Model	No
Acta Mater., 2015, 94, 257-270	Synchrotron XRD	Qualitative	No
Acta Mater., 2017, 126, 389-400	TEM	Qualitative	Local
Acta Mater., 2017, 132, 444-454	Cyclic experiments	Qualitative	Local
Acta Mater., 2018, 144, 874-883	XRD+SEM-DIC	Qualitative	Local
Int. J. Plast., 2018, 105, 99-127	Constitutive model	Model	No
Int. J. Plast., 2018, 111, 53-71	Experiment	Qualitative	Local
Acta Mater., 2019, 167, 149-158	Synchrotron XRD	Qualitative	Local
Scr. Mater., 2019, 162, 230-234	Cyclic test + TEM	Qualitative	No
Nat. Commun., 2025, 16, 56775	Microstructure	Qualitative	No
Mater. Des., 2020, 191, 108622	High-resolution DIC	Qualitative	Local
Mater. Sci. Eng. A, 2020, 782, 139271	TEM	Qualitative	Local
Int. J. Plast., 2023, 161, 103487	Cyclic experiments	Model	No
Int. J. Solids Struct., 2023, 280, 112384	Cyclic experiments	Model	No
Int. J. Plast., 2025, 194, 104483	Cyclic experiments	Conceptual	Local

Supplementary Figure 22

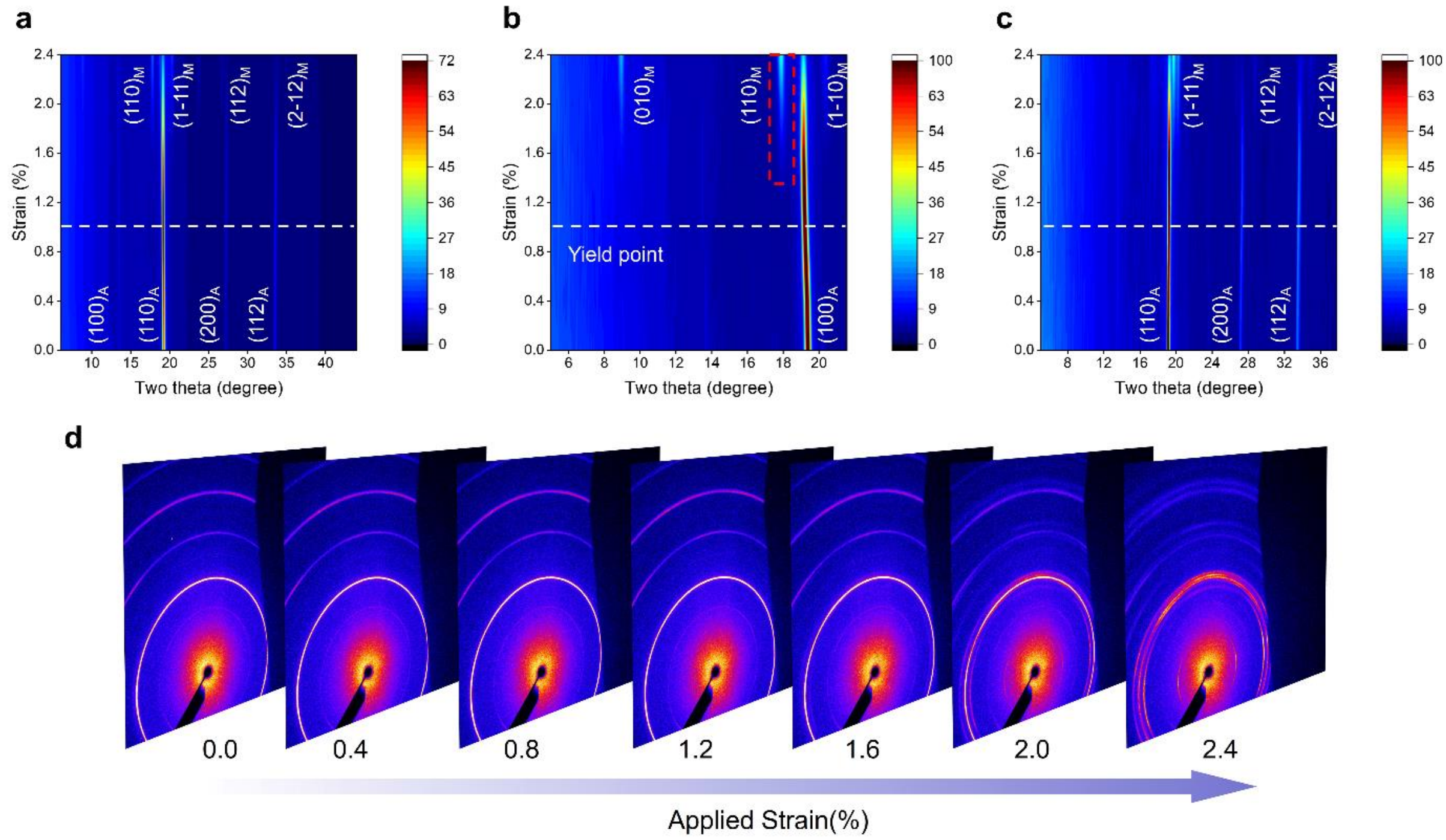

Supplementary Fig. 22 | Phase evolution during tensile loading characterized by in-situ XRD for the specimen annealed at 500 °C for 30 min. Measurements were performed using a NANOPIX-WE system with a Mo rotating anode X-ray source (λ = 0.7093 Å). **a–c** Evolution of diffraction intensity with strain along the FS, LD, and TD directions; B2 austenite (A) and B19′ martensite (M) are indicated, and dashed lines mark the yield point from the stress–strain curves. **d** 2D diffraction rings at different applied strains (0–2.4%). With increasing strain, the B19′ (1-11) and (2-12) reflections intensify within the 18.5° to 21.5° 2θ range, accompanied by attenuation of the B2 (110) peak. Notably, no discernible splitting of the B2(110) reflection into R-phase peaks (e.g., (300) or (211)) is observed, nor are any stable R-phase signatures detected[15,16]. Although subtle shoulder features emerge at high strains along the LD direction, their transient nature and negligible intensity preclude their identification as a B2→R→B19′ pathway. These results demonstrate that, despite the presence of Ni_4Ti_3 precipitates, the transformation is dominated by a direct B2→B19′ pathway within the investigated thermomechanical window.

Supplementary Figure 23

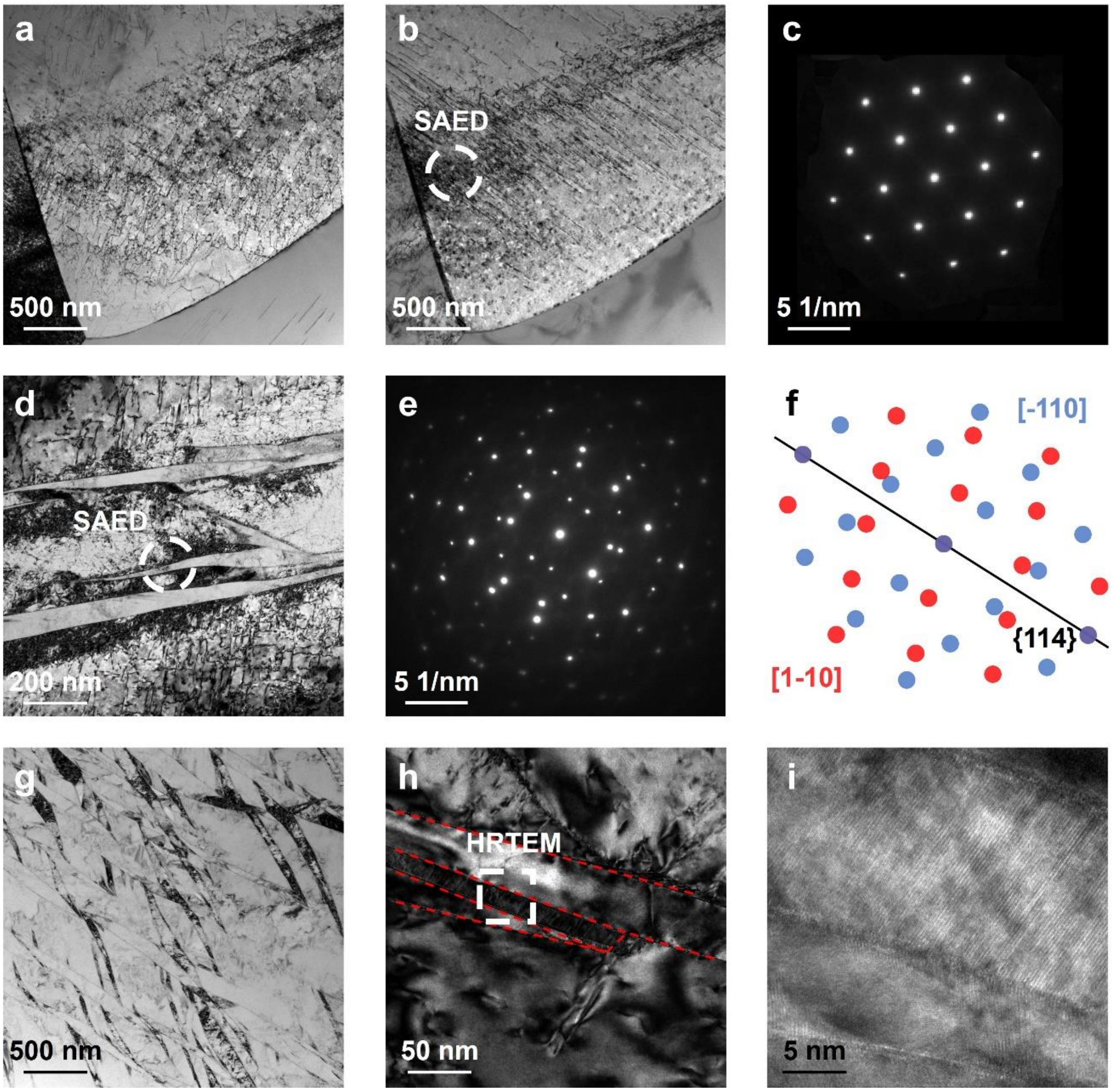

Supplementary Fig. 23 | TEM characterization of NiTi after cyclic loading at different strain amplitudes. **a**, **b** The bright-field images of the specimen cycled at 4% strain amplitude under different diffraction conditions, revealing a high density of dislocations but no discernible austenite twins or retained martensite plates. **c** SAED pattern from the region marked in (**b**). **d**–**f** TEM results for the specimen cycled at 6% strain amplitude, showing the appearance of {114} B2 austenite twins. **g** BF image of the specimen cycled at 8% strain, displaying extensive austenite twins. **h** Local observation of a representative twin band, and (**i**) corresponding HRTEM image from the boxed region in (**h**), where the twin boundaries are clearly resolved. Overall, superelastic degradation of the NiTi alloy under cyclic loading is primarily governed by dislocation accumulation and austenite twinning, with no retained martensite detected over the entire strain amplitude range.

Supplementary Figure 24

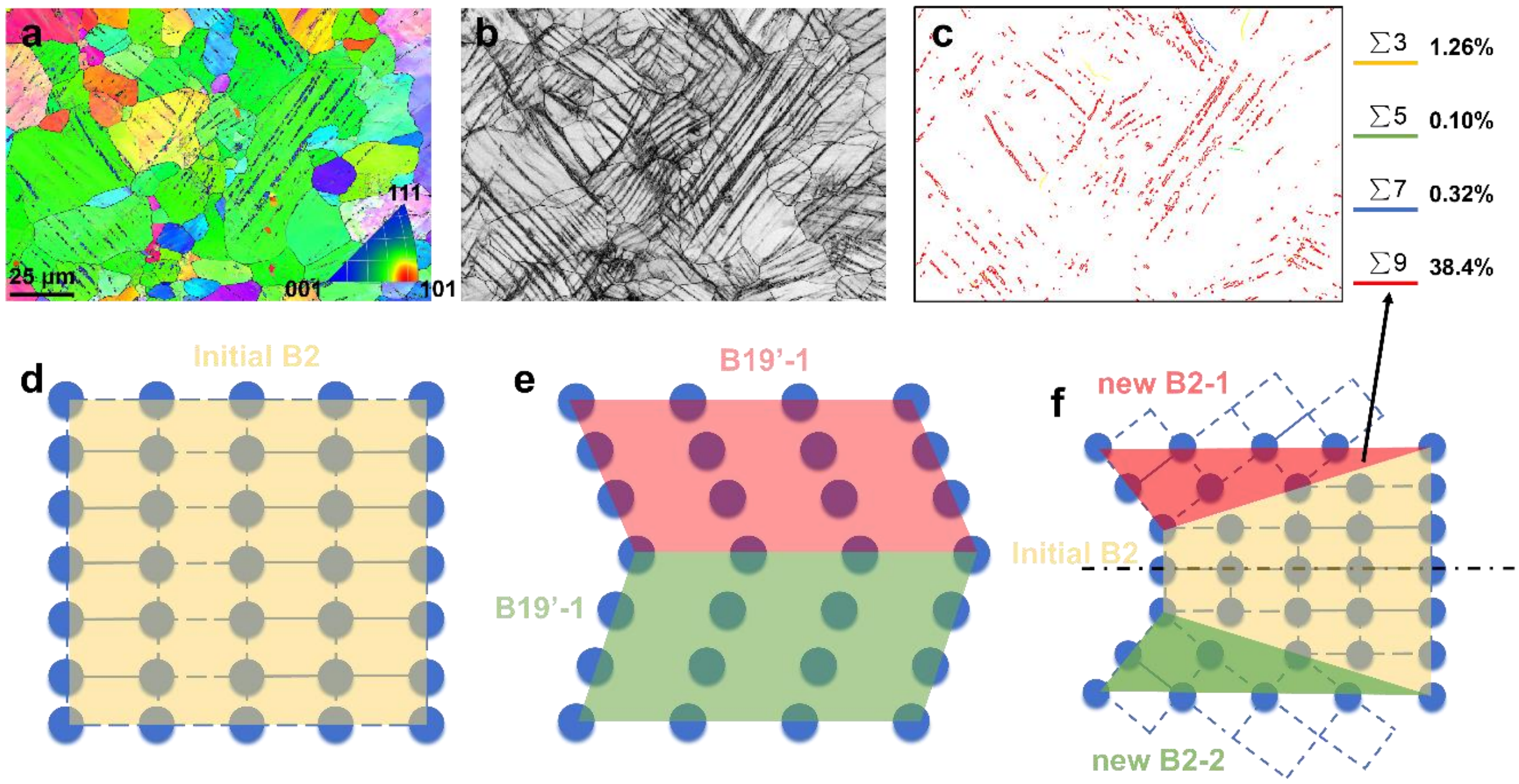

Supplementary Fig. 24 | EBSD characterization of the NiTi alloy annealed at 700 °C for 30 min after 5 cycles at 8% strain amplitude. a IPF map showing the development of a pronounced {101} texture. **b** Band contrast image, where dense deformation bands aligned along specific orientations traverse the grains after high-strain cycling. **c** Coincidence site lattice (CSL) boundary map revealing the extensive formation of Σ9 boundaries, reaching 38.4%, indicative of substantial grain boundary reconfiguration during deformation. We provide a schematic illustration of the formation mechanism of Σ9 special boundaries in B2 austenite during cyclic phase transformation. **d** The initial B2 phase (schematized using the {011} plane) exhibits a regular lattice. **e** Cyclic loading induces the formation of a composite B19′ martensite twin structure. **f** Subsequent reverse transformation produces two newly formed B2 regions with a relative lattice rotation that satisfies the Σ9 relation, thereby generating high densities of Σ9 boundaries at the microscale.

Supplementary Figure 25

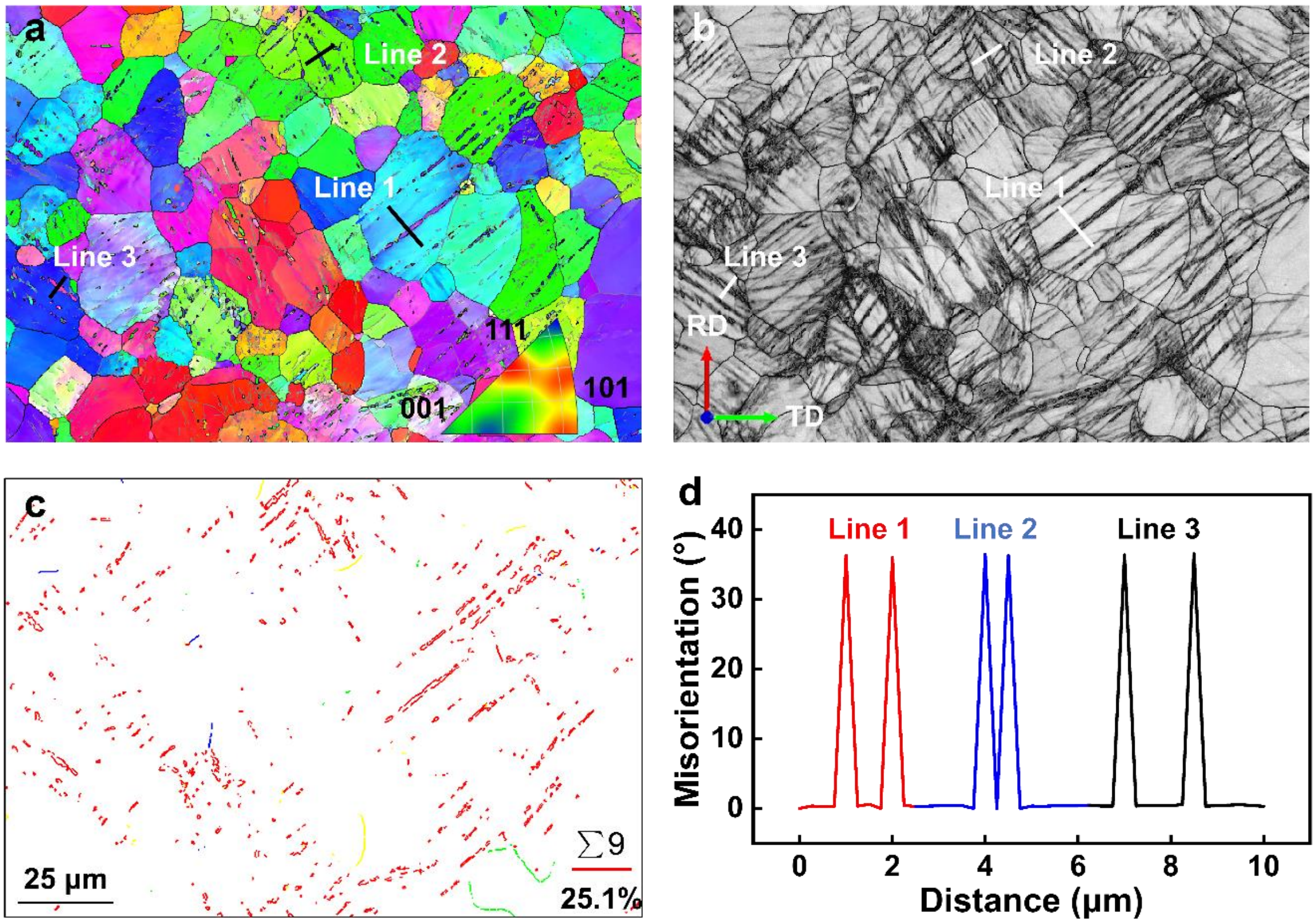

Supplementary Fig. 25 | EBSD characterization of the NiTi alloy annealed at 700 °C for 30 min after 10 cycles at 8% strain amplitude. a IPF orientation map, with Lines 1–3 indicating the paths used for subsequent misorientation profiles. **b** Corresponding BC image on the RD–TD section, showing numerous deformation twin bands traversing the grains along specific orientations after high-strain cyclic loading. **c** CSL boundary map, revealing a high density of Σ9 special deformation boundaries with an area fraction of 25.1%. **d** Intragrain misorientation profiles extracted along Lines 1–3 in (**a**, **b**) show the mismatch angle of approximately 36° when passing through the grain boundary, confirming the existence of deformation twins.

Supplementary Method 3

Dataset description: During dataset construction, EBSD maps obtained under various annealing conditions were first segmented into cropped sub-images, which were then paired with the corresponding Ni_4Ti_3 descriptor, cycle number, and strain amplitude to form the base samples. The dataset was divided into training, validation, and test sets in an 8:1:1 ratio. In practice, all sub-images cropped from the same raw EBSD map, together with their associated samples, were kept within the same subset during partitioning. Offline data augmentation (e.g., rotation and mirroring) was subsequently applied to the sub-images in the training set, resulting in a total of 31,536 labeled sequences. Details of the augmentation strategies are provided in Supplementary Fig. 29.

Framework description: In this study, pronounced modality gaps and semantic misalignment exist between microstructural features and loading conditions. Handling cross-modal entanglement and semantic inconsistency remains a central challenge for effective multimodal feature fusion. For clarity, we take GND maps and Euler angle matrices (EAM) as a representative pair of modalities. Most existing approaches[17] simply concatenate these features and then perform feature extraction, which can be expressed as:

$$P = h_{\text{pred}}(R \,|\, F_{GND}(B_{GND}), F_{EAM}(B_{EAM})\,) \tag{10}$$

Here, $F_{GND}(B_{GND})$ and $F_{EAM}(B_{EAM})$ represent GND and EAM features, respectively. [] denotes the concatenation operation. R denotes the extracted feature representation, which is subsequently fed into the prediction head h_{pred} to obtain the prediction result P. Although concatenating multimodal features followed by unified feature extraction simplifies the modeling process, this strategy overlooks latent inconsistencies between modalities, leaving the intrinsic conflicts in complex tasks unresolved. On the other hand, Wu et al.[18] explicitly account for such cross-modal inconsistencies by decomposing the feature encoding process into multiple coordinated subtasks, which can be formulated as:

$$P = h_{\text{pred}}([R_{GND} \,|F_{GND}(B_{GND}), R_{EAM} \,|F_{EAM}(B_{EAM})]) \tag{11}$$

Among them, R_{GND} and R_{EAM} represent the GND and EAM feature representations, respectively. This method only segregates feature extraction through two independent networks, while failing to explore and leverage their mutual cooperation. Distinct from the above methods, the construction of our model accommodates the disentanglement and modulation of signals, which is inspired by cognitive neuroscience[19]. From the perspective of feature learning, it can be expressed as:

$$
\begin{cases}
f_{GND} = \varepsilon_{GND}(R_{GND} \left| F_{GND}(B_{GND}), R_{EAM} \right| F_{EAM}(B_{EAM})) \\
f_{EAM} = \varepsilon_{EAM}(R_{EAM} \left| F_{EAM}(B_{EAM}), R_{GND} \right| F_{GND}(B_{GND})) \\
P = h_{\text{pred}}(\phi(f_{GND}, f_{EAM}))
\end{cases}
\tag{12}
$$

Here, ε and ϕ denote the feature alignment and merging operations, respectively. Specifically, we first employ two backbone networks to extract GND and EAM features independently, as illustrated in Fig. 1 of the main text. Since features learned by different backbones may exhibit semantic misalignment, we develop a feature synergy module to align and merge these disentangled representations. Note that the design inspiration for this module is derived from visual perception systems that integrate multipartite information, although the underlying mechanism remains unknown (Supplementary Fig. 27a). Therefore, the method proposed in this paper emphasizes drawing lessons from its conceptual basis, while the technical details are not strictly aligned.

Feature synergy module: We designed a feature synergy module to mitigate the contextual misalignment between modalities. This module comprises feature alignment and merging, which are detailed in this section. As illustrated in Supplementary Fig. 27b and Eq. (13), the alignment step primarily involves estimating an offset and applying it to the corresponding feature representations:

$$
\begin{cases}
\Delta_i = f_0^i([R_{GND} \left| F_{GND}(B_{GND}), R_{EAM} \right| F_{EAM}(B_{EAM})]),\ i \in \{GND, EAM\} \\
\hat{F}_i = f_1^i(R_i \left| F_i(B_i), \Delta_i \right)\ i \in \{GND, EAM\}
\end{cases}
\tag{13}
$$

where f_0 and f_1 represent functions that learn coordinate offsets of the features and align the features with offsets, respectively. In this work, they are implemented using 3×3 deformable convolution, activation function and standard convolutions with the same kernel size. In the feature merging stage, we emphasize the significant feature maps that contain a large amount of spatial information, while suppressing redundant information to achieve precise allocation. The process is shown in Equation (14):

$$
\begin{cases}
u = \text{GAP}([\hat{R}_{GND}, \hat{R}_{EAM}]) \\
R_i^* = \hat{R}_i + \sigma(\text{W}_i \otimes u) \odot \hat{R}_i, i \in \{GND, EAM\} \\
R = R_{GND}^* \oplus R_{EAM}^*
\end{cases}
\tag{14}
$$

where u is obtained by global average pooling after concatenation of $\hat{R}_{GND}$ and $\hat{R}_{EAM}$. σ represents the Sigmoid function, and W_i refers to the 1×1 convolution kernel. Besides, we add residual connections to prevent any response of a particular channel from being overly amplified or suppressed. Ultimately, the obtained feature R is used for prediction.

Supplementary Figure 26

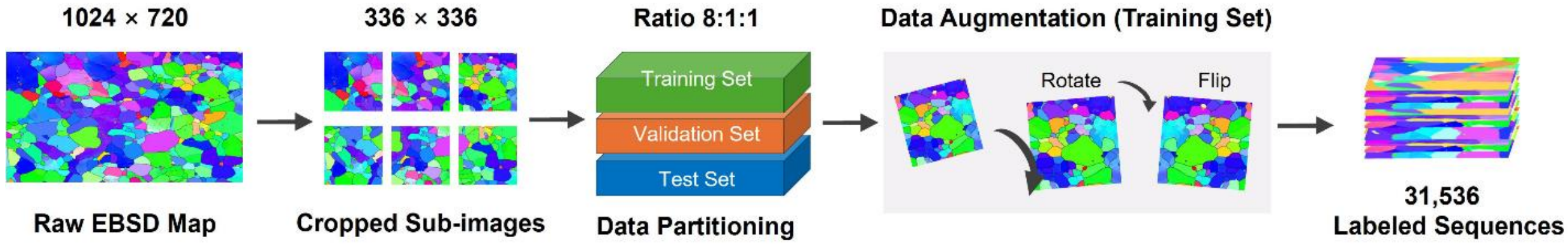

Supplementary Fig. 26 | Schematic illustration of the dataset construction and partitioning workflow. Raw EBSD maps are segmented into cropped sub-images, followed by data partitioning (training/validation/test = 8:1:1) to avoid spatial leakage. Data augmentation is applied only to the training set, resulting in 31,536 labeled sequences.

Supplementary Figure 27

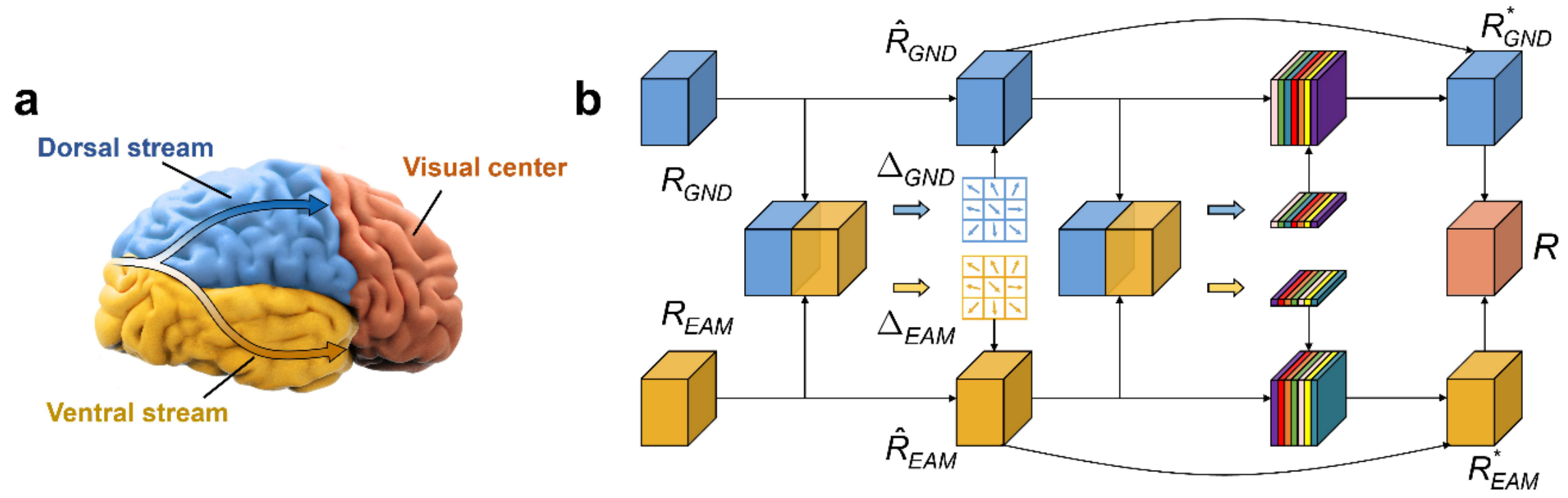

Supplementary Fig. 27 | Design concept and structure of the feature synergy module. a The design draws inspiration from the human visual system, where two streams separately process recognition and localization, enabling complementary information processing. **b** Illustration of the feature synergy module consisting of both feature alignment (*i.e.*, ε_{GND} and ε_{EAM}) and feature merging operations (*i.e.*, ϕ). Refer to the text description for more details.

Supplementary Note 2

Deep learning model structure: Each branch uses the same feature extraction backbone. The input tensor of size $H \times W \times 3$ is first passed sequentially through five stacked ConvBlocks. Each ConvBlock consists of a 2D convolution, batch normalization, ReLU, dropout, and a pooling layer; downsampling pooling is enabled only in selected blocks to moderately compress spatial resolution and expand the receptive field while controlling network depth, and batch normalization together with dropout helps suppress overfitting. A DilConvBlock is then introduced to reinforce multi-scale context modeling: this module applies four parallel 3×3 dilated convolutions with dilation rates of 1, 2, 3, and 4 to the same feature map, concatenates their outputs along the channel dimension (dim = 1). The output of the DilConvBlock is compressed into a 1D vector by global adaptive average pooling and then mapped to three scalar features via several fully connected layers. Applying this pipeline to the Euler, GND, and phase branches yields three sets of matrix features, which are combined with numerical features and fed into the feature synergy module of the main network.

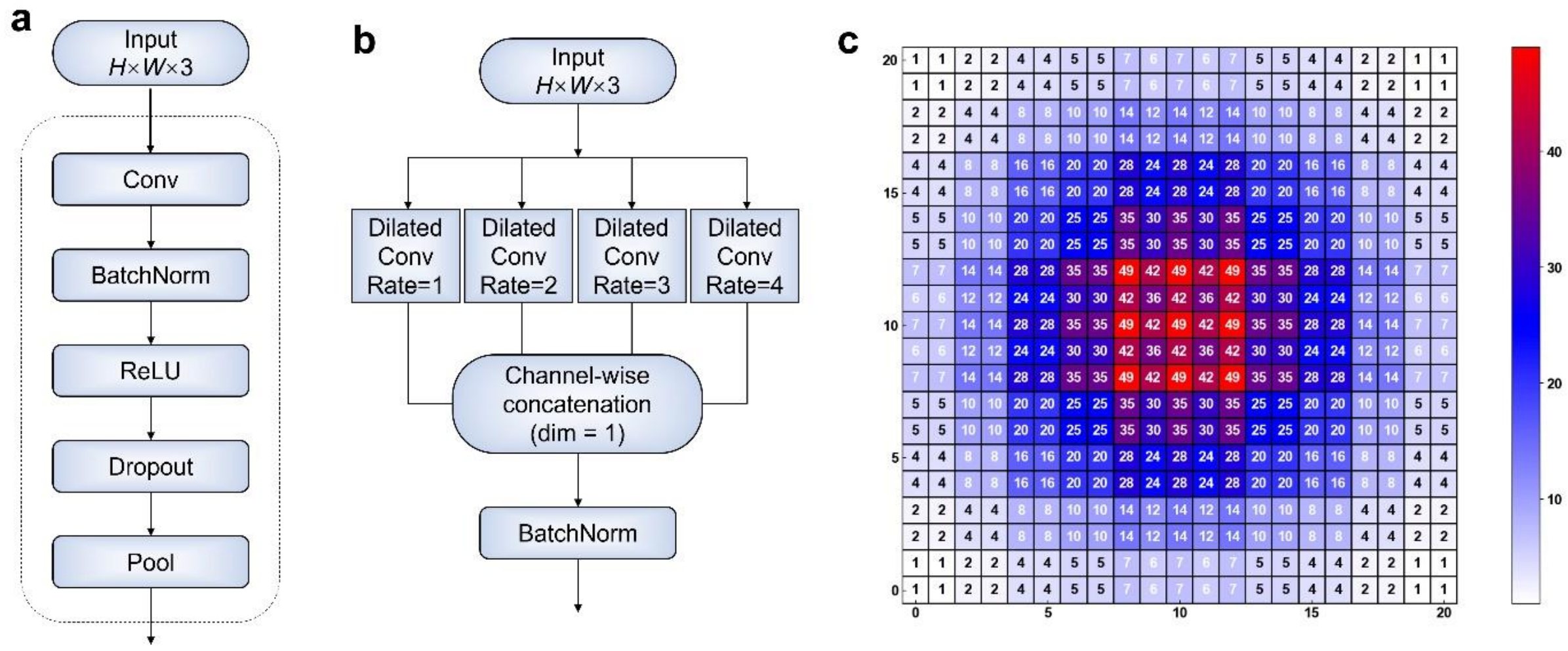

Supplementary Fig. 28 | Schematic of the single-branch network architecture. a Structure of the ConvBlock, consisting of convolution, batch normalization, ReLU, dropout, and pooling layers in sequence. **b** Structure of the DilConvBlock, comprising four parallel 3×3 dilated convolutions with dilation rates of 1, 2, 3, and 4; their outputs are concatenated along the channel dimension (dim = 1) and followed by batch normalization. **c** Illustration of the sampling pattern and effective receptive field of the dilated convolutions, showing the number of times each spatial location is used.

Supplementary Table 8

Supplementary Table 8 | Hyper-parameters and their descriptions

Hyper-parameter	Description
Data configuration	
num_images=3	Number of input images; matrix treated as images
num_parameters=3	Number of input numerical parameters
num_outputs=1	Number of predicted outputs
image_size=336	Input image size
Model configuration	
backbone	Custom CNN backbone; optional pretrained ResNet-18/34/50
num_blocks=5	Number of convolution blocks
feature_dim=512	Feature dimension after convolution
num_feature=3	Number of features extracted from a single input image
dropout=0.5	Dropout rate for regularization
ReLU	Activation function
Training configuration	
batch size = 32	Number of samples used in each iteration
epochs = 150	Number of model iterations
Loss = MSE	Loss function
Adam	Adaptive moment estimation optimizer
LR = 0.001	Initial learning rate
WD=0.0001	Weight decay
Cosine	Learning rate scheduler
early_stopping=10	Early stopping patience

Supplementary Table 9

Supplementary Table 9 | Initialization parameters of different ML models. For each comparative network (MCC-Net, ECMMF-Net, Base-w/oSM, MBNI-Net), we conducted a small grid search over the Adam hyperparameters on the validation set, while keeping all other optimizer settings at their default values. The final configuration for each model is summarized

Model	Batch size	Optimizer	Loss function	Initial LR	Weight decay
MCC-Net	32	Adam	MSE	1.0×10^{-3}	1.0×10^{-4}
ECMMF-Net	32	Adam	MSE	5.0×10^{-4}	2.0×10^{-4}
Base-w/oSM	32	Adam	MSE	1.5×10^{-3}	5.0×10^{-5}
MBNI-Net	32	Adam	MSE	5.0×10^{-4}	2.0×10^{-4}
Ours	32	Adam	MSE	1.0×10^{-3}	1.0×10^{-4}

Supplementary Method 4

Data augmentation: Given the limited dataset size in this study, we adopted offline data augmentation to expand the effective sample size. Images were preprocessed using the Imgaug Python library, and the augmented samples were then fed into the network for training. Seven augmentation strategies were used, including geometric transforms (horizontal flipping, small-angle random rotation of approximately ±10°–15°, and shear affine transformation) and photometric perturbations (Gaussian blur with Gaussian noise, random brightness adjustment, and contrast/gamma perturbation), as well as Mosaic augmentation, in which four images were randomly cropped and stitched into composite scenes.

Horizontal flipping, small-angle rotation, and shear primarily enhance the robustness to variations in orientation and pose, whereas blur+noise, brightness, and contrast/Gamma perturbations enrich the distribution of imaging conditions and appearance. Mosaic augmentation further increases the diversity of spatial compositions and structural patterns[20]. Together, these seven strategies provide a balanced and semantically consistent expansion of the small-scale dataset, as illustrated in Supplementary Fig. 29.

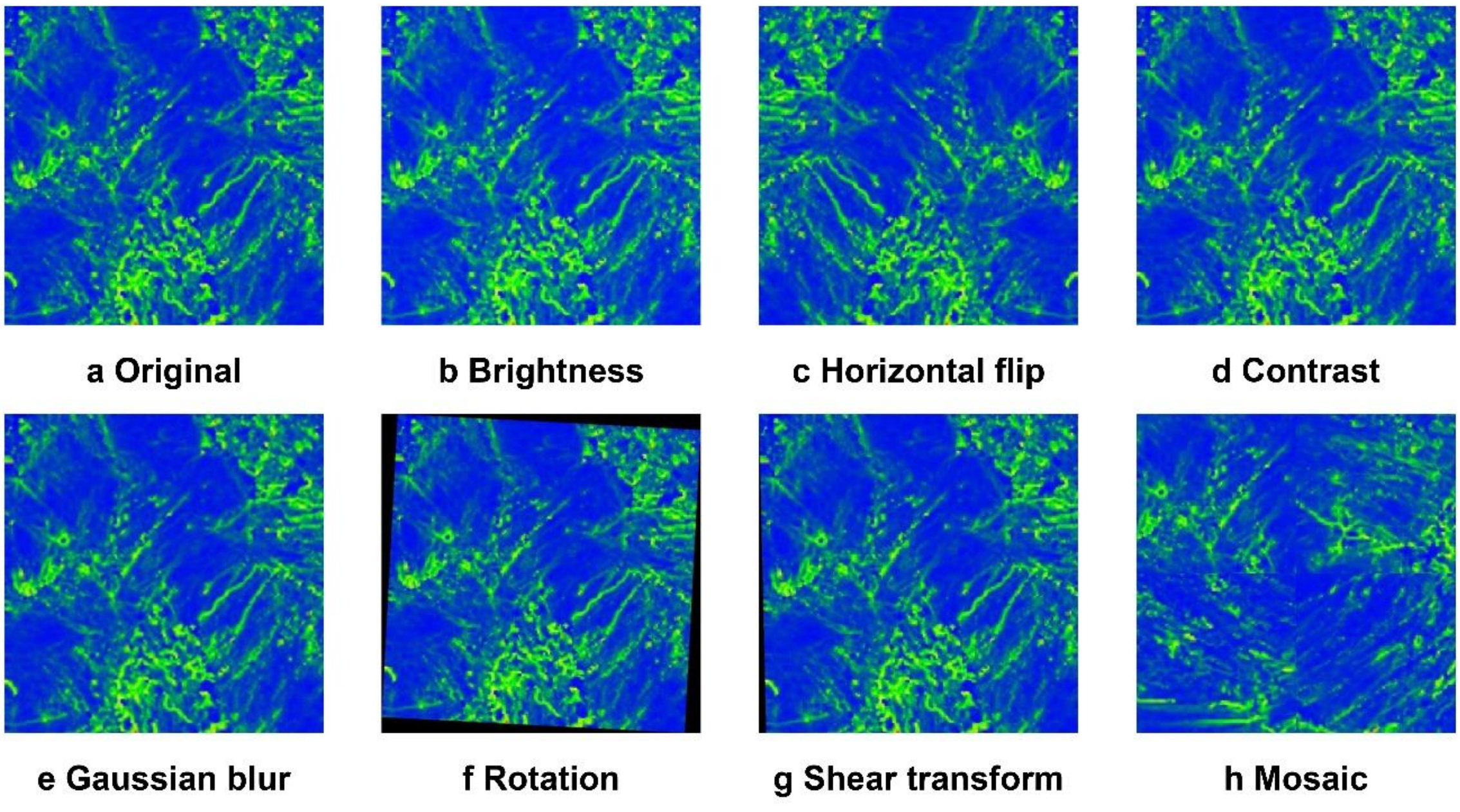

Supplementary Fig. 29 | Schematic illustration of the data augmentation effect on the GND map.

Supplementary Figure 30

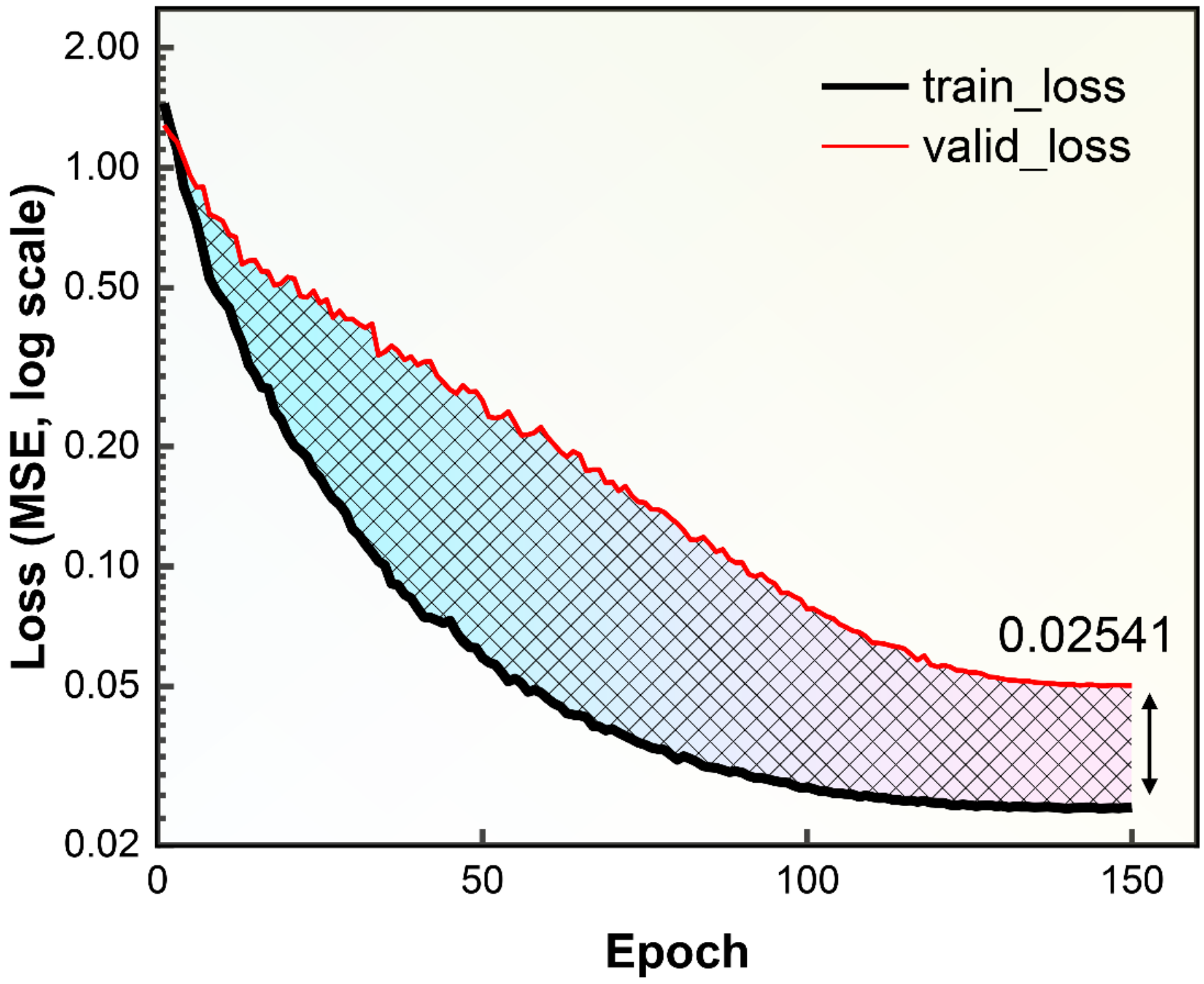

Supplementary Fig. 30 | Evolution of MSE loss during learning. Training and validation losses decrease rapidly and stabilize, followed by minor fluctuations without further reduction. The validation loss remains consistently higher than the training loss, likely due to the high-dimensional nature and relatively small scale of the dataset[21]. At the conclusion of the process, the model had fully converged with a validation loss of 0.0503.

Supplementary Figure 31

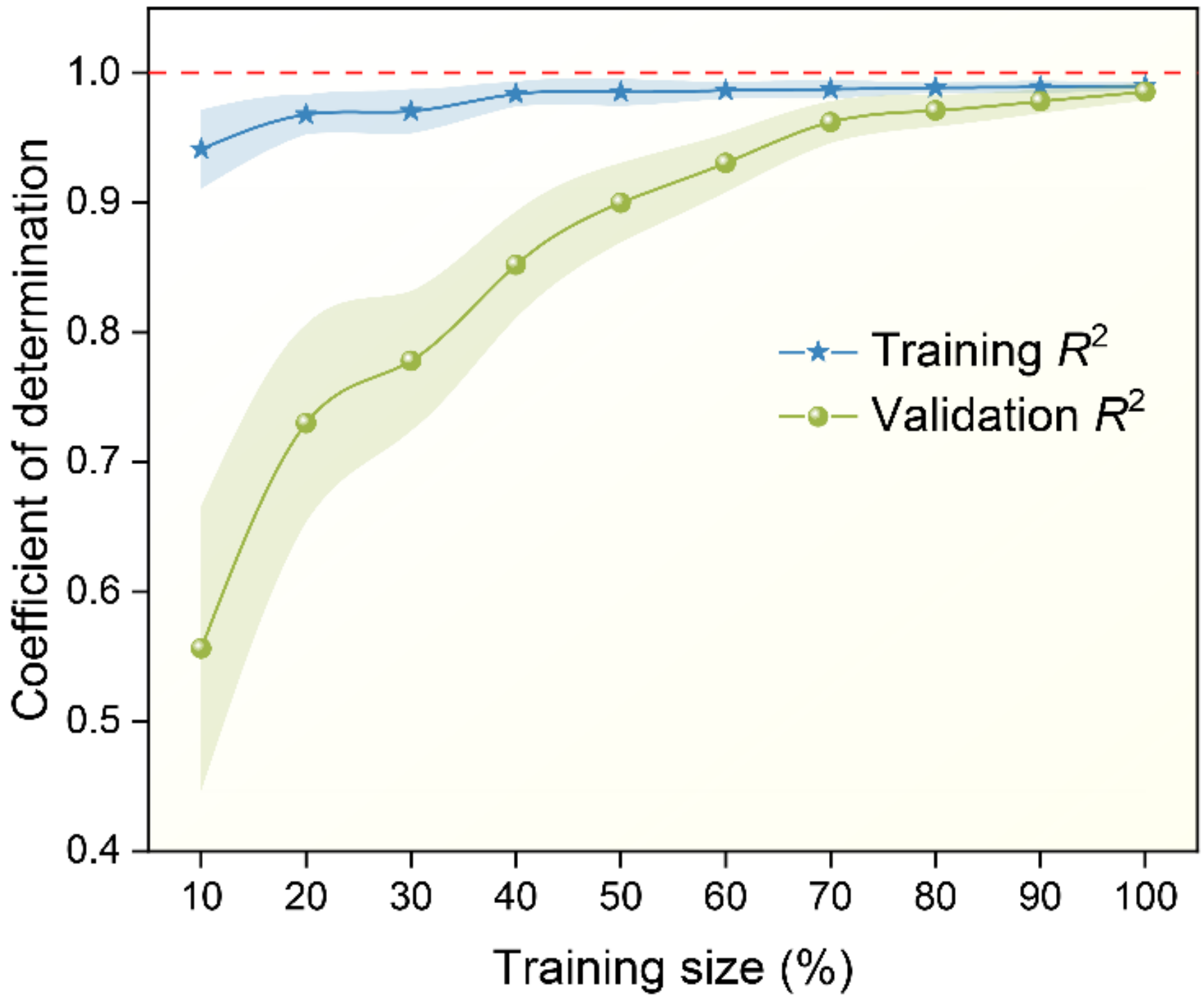

Supplementary Fig. 31 | Learning curve and overfitting assessment. Evolution of R^2 for training and validation sets across varying training proportions (10%–100%). Shaded regions represent the standard deviation from $n = 10$ random partitions. The narrowing gap and subsequent convergence beyond 70% training data signify that the model effectively learns the underlying multimodal mapping. The close alignment of the two curves at the plateau suggests minimal overfitting and strong generalization performance under the existing data scale.

Supplementary Discussion 1

Multimodal representation and performance: To quantitatively assess multimodal integration during training, two representational metrics were introduced. The inter-modality distance is defined as the mean pairwise cosine distance (1−cosine similarity) among the three image modalities. In addition, following the Representational Dissimilarity Matrix (RDM) theory[22], a latent representation distance matrix $D_{ij}^{\text{latent}} = 1 - \cos(r_i, r_j)$ and a corresponding physical output dissimilarity matrix $D_{ij}^{\text{out}} = \left| y_i - y_j \right|$ were constructed for all training samples. The latent–output RDM correlation was then determined by calculating the Spearman correlation coefficient between the upper triangular elements of these two matrices. This metric quantifies the consistency between inter-sample distances in the latent space and the corresponding differences in residual strain:

$$\rho_{\text{RDM}} = \text{SpearmanCorr}(\text{upper}(D^{\text{latent}}), \text{upper}(D^{\text{out}})) \tag{15}$$

Clear associations are observed among the representational and performance metrics: as inter-modality distance diminishes, the latent–output RDM correlation increases (R = −0.94), accompanied by a concurrent rise in test R^2. Furthermore, the latent–output RDM correlation is significantly positively correlated with predictive accuracy (R = 0.95). These findings demonstrate that latent space optimization during training manifests as both the convergence of multimodal features and enhanced consistency between inter-sample distances and true residual strain gradients. In other words, when latent representations better reflect relative differences in residual strain, model prediction accuracy improves accordingly.

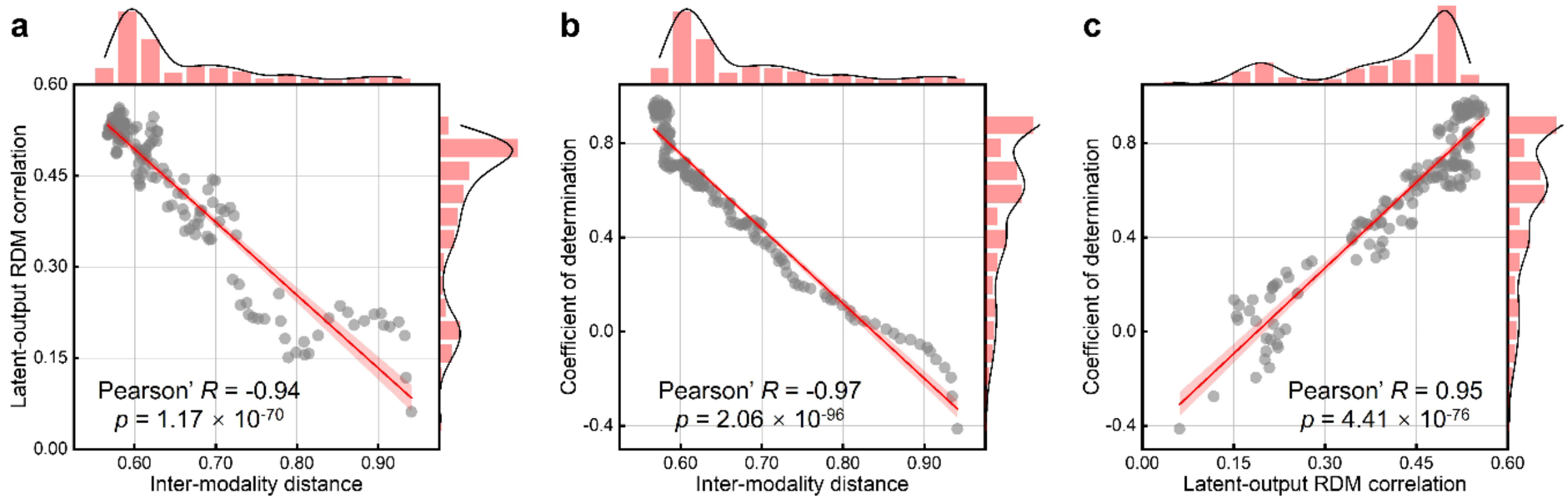

Supplementary Fig. 32 | Quantitative association between multimodal representation evolution and prediction performance. Each point represents a training stage. Metrics are computed using training samples and evaluated on an independent test set. As training progresses, inter-modality distance decreases steadily, accompanied by concurrent increases in latent–output RDM correlation and test R^2. These trends indicate that reduced inter-modal separation leads to a latent space that more faithfully captures residual strain differences, thereby enhancing predictive performance.

Supplementary Figure 33

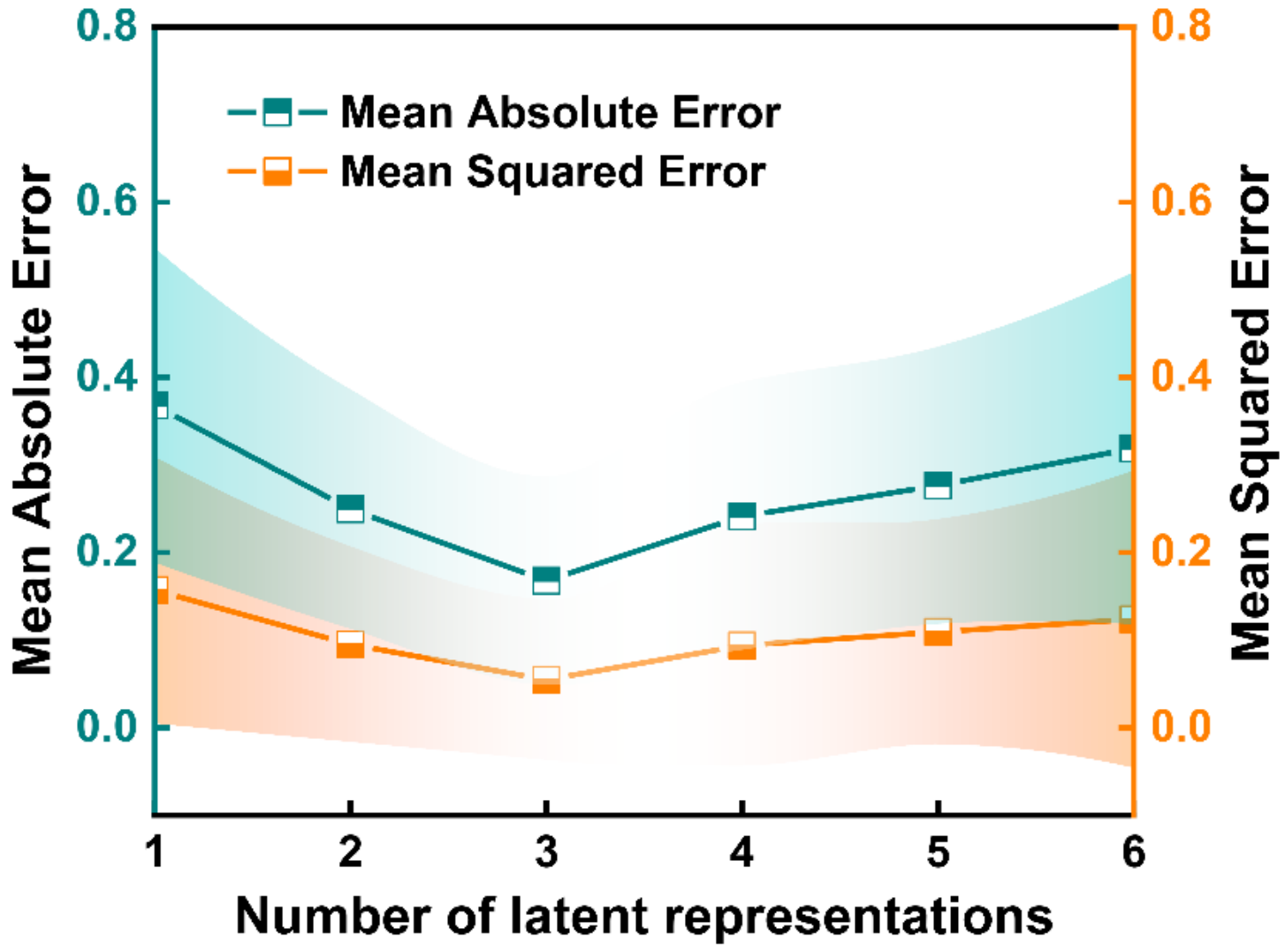

Supplementary Fig. 33 | Ablation study on the number of convolutional feature representations. This figure depicts the evolution of prediction errors under varying counts of latent feature representations (n = 1–6). Optimal performance across both metrics is attained when the image-modality branch extracts three feature representations. Further increases in feature dimensionality result in higher errors, likely due to modality imbalance. Given that the numerical modality comprises only scalars, an overly complex image representation may dominate the fusion stage, leading to scale mismatch and reduced synergy[23]. Consequently, a moderate number of representations strikes an optimal balance between informational expressiveness and redundancy control.

Supplementary Figure 34

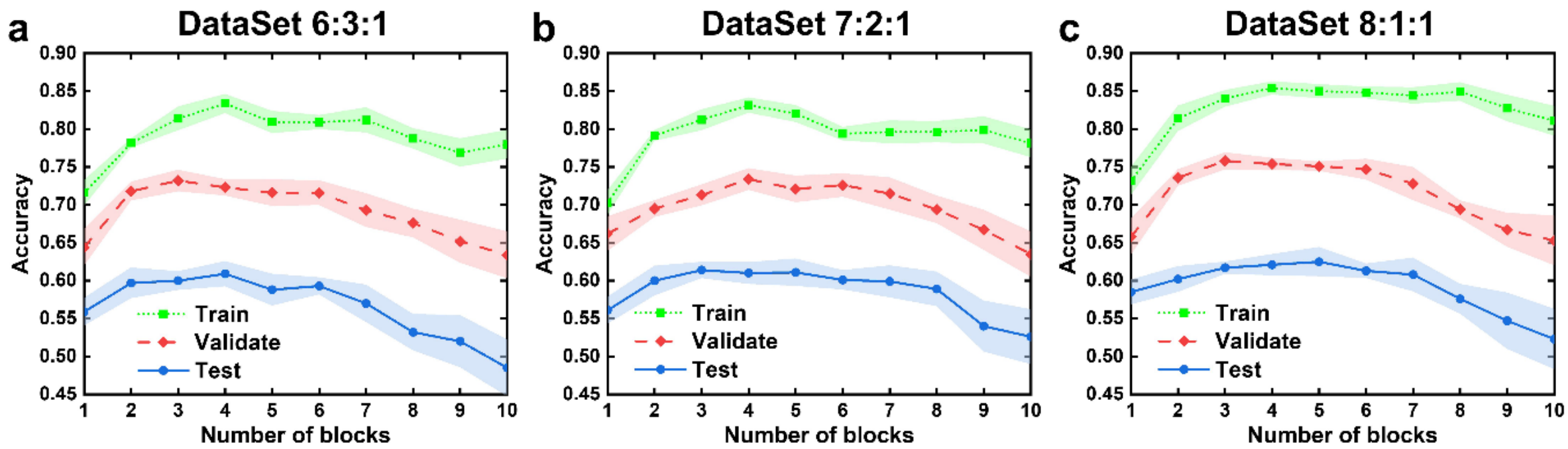

Supplementary Fig. 34 | Sensitivity analysis of data-splitting ratios and network block numbers. Accuracy trajectories for training, validation, and test sets under three partitions: (**a**) 6:3:1, (**b**) 7:2:1, and (**c**) 8:1:1. Accuracy is computed following our previous study[24]. While the test set was kept constant, increasing the training proportion yielded consistent performance gains, peaking at the 8:1:1 ratio. The influence of network depth showed a parabolic trend, peaking at 3–5 blocks. Further increasing depth provides no additional benefit; instead, it introduces feature redundancy and representation saturation, leading to reduced stability and a decline in predictive accuracy.

Supplementary Discussion 2

Why Euler orientation matrix instead of alternative encodings? We encode the three Euler angles into three channels to construct the Euler matrix that represents the actual crystal orientation, rather than directly using the more readily available IPF map. The IPF map essentially compresses the orientation of a selected reference direction in the crystal frame into RGB colors, where a single color may correspond to multiple crystallographic orientations (Supplementary Fig. 35a). In contrast, the Euler matrix fully describes all three degrees of freedom. Interestingly, this approach unexpectedly introduces a new modality, yet it can still be processed in the same manner as an image matrix. It should be noted that Euler angle parameterization may introduce redundancy and discontinuities. To address this, we constructed a control model based on quaternion representation, which has been widely adopted as a mathematically robust and non-singular alternative to Euler angles for crystallographic orientation representation and EBSD learning tasks[25,26]. The results indicate that, compared to the Euler angle representation, it did not yield significant performance improvements. This may be attributed to the fact that, in the current task, model learning is primarily dominated by orientation variations and their spatial distributions, thereby reducing dependence on specific parameterization forms. Therefore, in this study, the Euler matrix is sufficient to characterize the relevant information. If future work extends to modeling local orientation, continuous orientation encodings such as quaternions may offer advantages.

re-IPF: In this ablation variant, we replace the Euler matrix with the IPF map as the orientation input.

re-Quat: In this ablation variant, we replace the Euler matrix with the quaternion matrix (encoded into four channels) as the orientation input.

Why is PCA used to reduce the dimensionality of Ni_4Ti_3 features? We extract multiple descriptors of Ni_4Ti_3 from TEM images, including diameter, aspect ratio, and area, and then compress them into a single principal component via PCA. Similar strategies have been applied in other material systems. For instance, researchers have used PCA to reduce the dimensionality of γ'-phase morphological descriptors, improving the robustness of creep strain prediction in Ni-based superalloys[27]. In the present work, if all precipitate features are directly concatenated into the numerical input branch, the performance degrades significantly (Supplementary Table 10). This likely occurs because the inflated dimensionality of the numerical branch interferes with weight allocation among other dominant factors.

w/o-PCA: We omit the PCA step and directly concatenate all individual Ni_4Ti_3 precipitate features into the numerical input branch.

Importance of GND and phase maps. For NiTi alloys, regions with elevated GND density indicate a high density of geometrically necessary dislocations associated with lattice curvature and crystallographic incompatibility, which are more susceptible to damage evolution and functional degradation. Here, GND is used as a scalar field to characterize the spatial heterogeneity of dislocation density, while detailed mechanistic discussion is beyond the scope of this study. $NiTi_2$ appears as incoherent, brittle second-phase particles along grain boundaries or within grains, acting as stress concentrators, promoting dislocation slip and microcrack initiation, and thereby degrading superelastic stability[28,29]; our observations align with this view (Supplementary Fig. 35b). Ablation experiments show that removing either the GND or phase map input leads to a marked drop in accuracy.

w/o-GND: we remove the GND channel and retain only the Euler matrices and phase maps as matrix inputs.

w/o-Phase: we remove the phase map channel and retain only the Euler matrices and GND maps as matrix inputs.

re-KAM: in this ablation variant, the GND maps are replaced with KAM maps.

Other available microstructural information. BC maps primarily capture the contrast and sharpness of backscattered electron patterns (Supplementary Fig. 35d), thereby allowing visualization of grain morphology, grain boundaries, and surface conditions. We tested BC as a fourth image channel, but the gain in prediction accuracy was negligible, so it was not retained in the main framework. A plausible explanation is that degradation-related information is already encoded in the existing inputs, whereas BC contributes little independent signal and is highly sensitive to sample preparation and imaging conditions, thus introducing task-irrelevant noise.

ad/BC: we additionally feed the BC map as a fourth image channel, with the remaining network architecture and training configuration unchanged.

Feature synergy module. The extracted matrix features and numerical features are projected into a shared embedding space to mitigate cross-modal context misalignment, thereby improving the effectiveness and stability of multimodal fusion.

re-FSM: in this ablation variant, the feature synergy module is replaced with a multi-head self-attention module[30].

Supplementary Figure 35

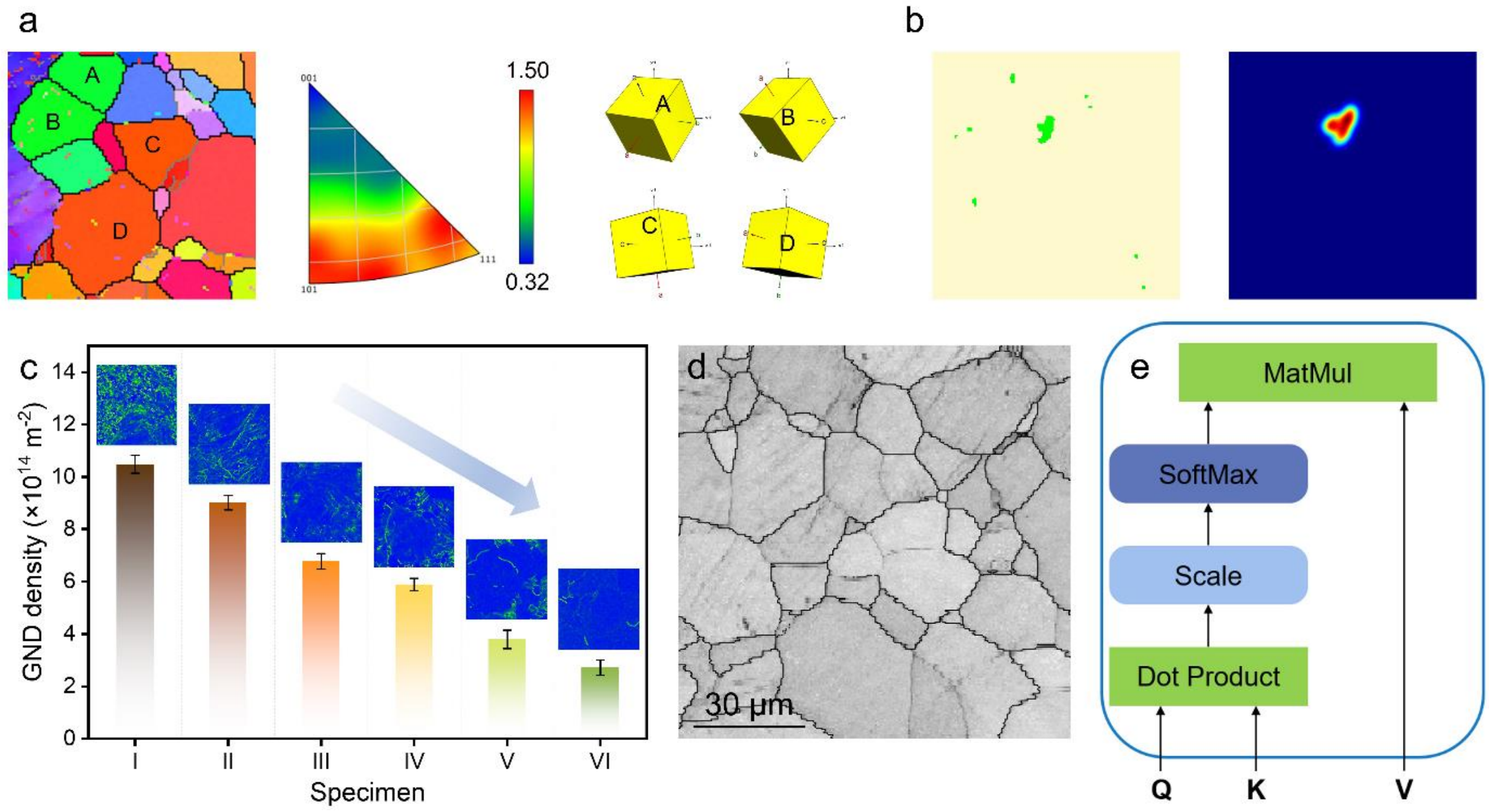

Supplementary Fig. 35 | Illustration of input modalities and model components. a IPF map and crystallographic orientation schematics of four selected grains A–D, illustrating how grains A/B or C/D differ markedly in orientation yet still exhibit similar colours, reflecting the information simplification in the IPF representation. **b** Phase map, where green denotes $NiTi_2$ precipitates and the light background denotes the matrix, together with the corresponding response heatmap derived from this map. **c** Mean GND density for specimens I–VI subjected to various heat treatments, with representative GND maps for each specimen shown above. **d** BC image highlighting the grain morphology and grain boundaries. **e** Schematic of the self-attention mechanism. The **Q** matrix for each element is multiplied with the **K** matrix of the other elements, and the result is then scaled and fed into the Softmax function for normalization. Finally, the **V** matrix is multiplied by the Softmax output to determine the relative importance of each element.

Supplementary Table 10

Supplementary Table 10 | Ablation study of model components in MBFFA

Method	MSE (10^{-1})	MAE	R^2 (10^{-2})
w/o-PCA	0.642	0.314	85.19
w/o-GND	0.634	0.307	86.26
w/o-Phase	0.579	0.202	89.89
re-FSM	0.571	0.198	93.52
re-IPF	0.567	0.188	95.37
ad/BC	0.558	0.179	97.05
re-KAM	0.552	0.172	98.02
re-Quat	0.544	0.167	98.15
Base	0.545	0.168	98.13

Supplementary Table 11

Supplementary Table 11 | Comparative prediction performance of GND and KAM features under varied fusion strategies. KAM+GND v1 and v2 denote configurations with three and two feature representations per channel, respectively. GND alone achieves a slight improvement over KAM. However, incorporating GND into a fused representation degrades performance. Reducing the number of feature representations (v2) does not mitigate this effect, indicating that the degradation is not primarily caused by increased feature dimensionality, but is more likely attributed to feature redundancy and physical inconsistency

Configuration	MSE (10^{-1})			MAE		
	Train	Val	Test	Train	Val	Test
KAM+GND v2	0.365	0.708	0.826	0.137	0.189	0.210
KAM+GND v1	0.298	0.589	0.695	0.126	0.172	0.191
KAM	0.235	0.496	0.552	0.115	0.160	0.172
GND	0.249	0.503	0.545	0.116	0.160	0.168

Supplementary Figure 36

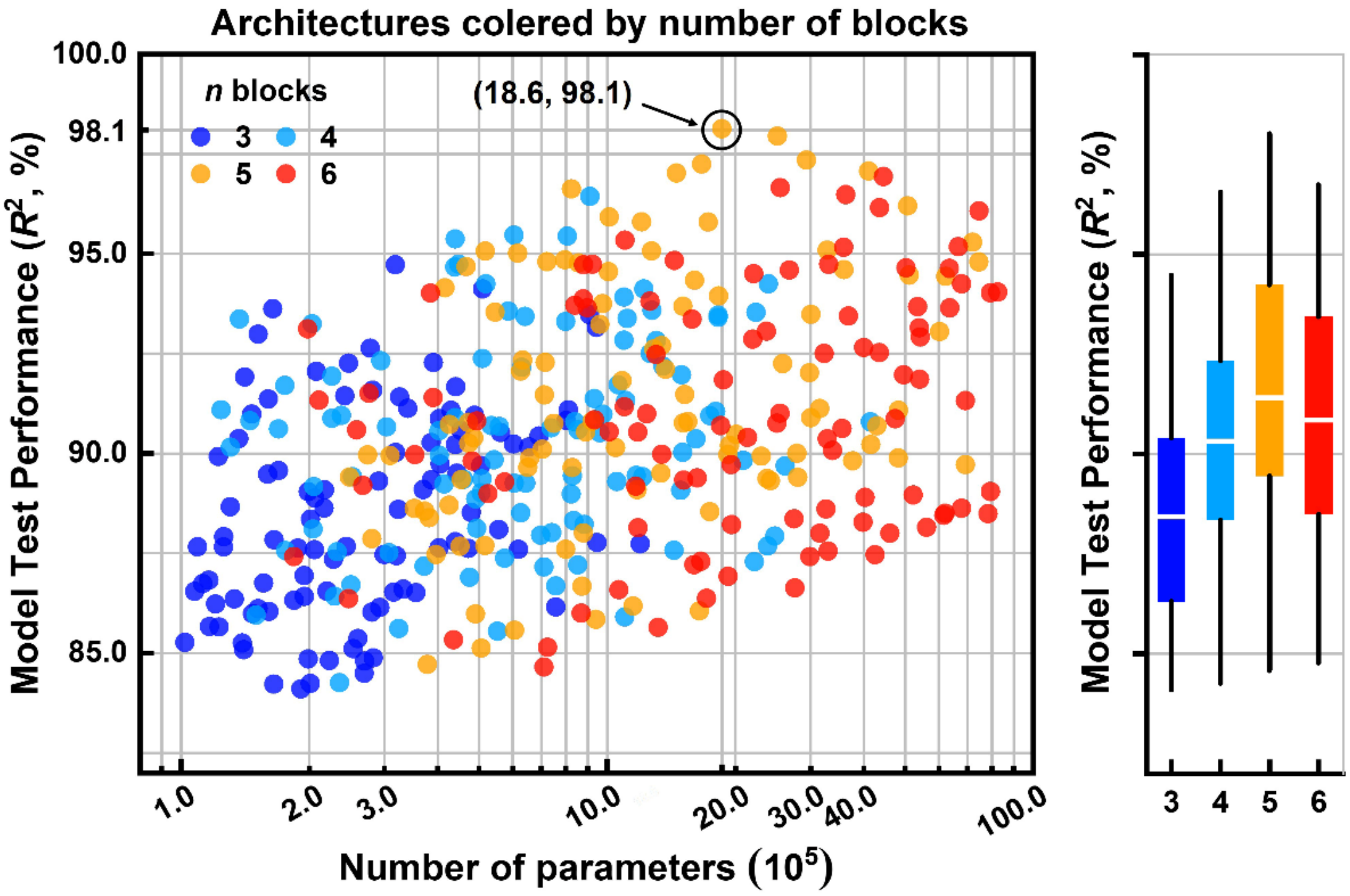

Supplementary Fig. 36 | Influence of network complexity on model performance. Each point represents a candidate architecture, plotted against the number of trainable parameters and colored by the number of ConvBlocks; boxplots summarize the distributions of test R^2 across network depths. The parameter–performance relationship exhibits a convex trend, with rapid initial improvement followed by a gradual slowdown. An optimal balance is achieved at five ConvBlocks, yielding a peak R^2 of 98.1% with 1.86×10^6 parameters. Further increases in depth provide no consistent gains and instead lead to performance degradation, indicative of overfitting. This configuration was therefore adopted as the baseline for subsequent analyses.

Supplementary Figure 37

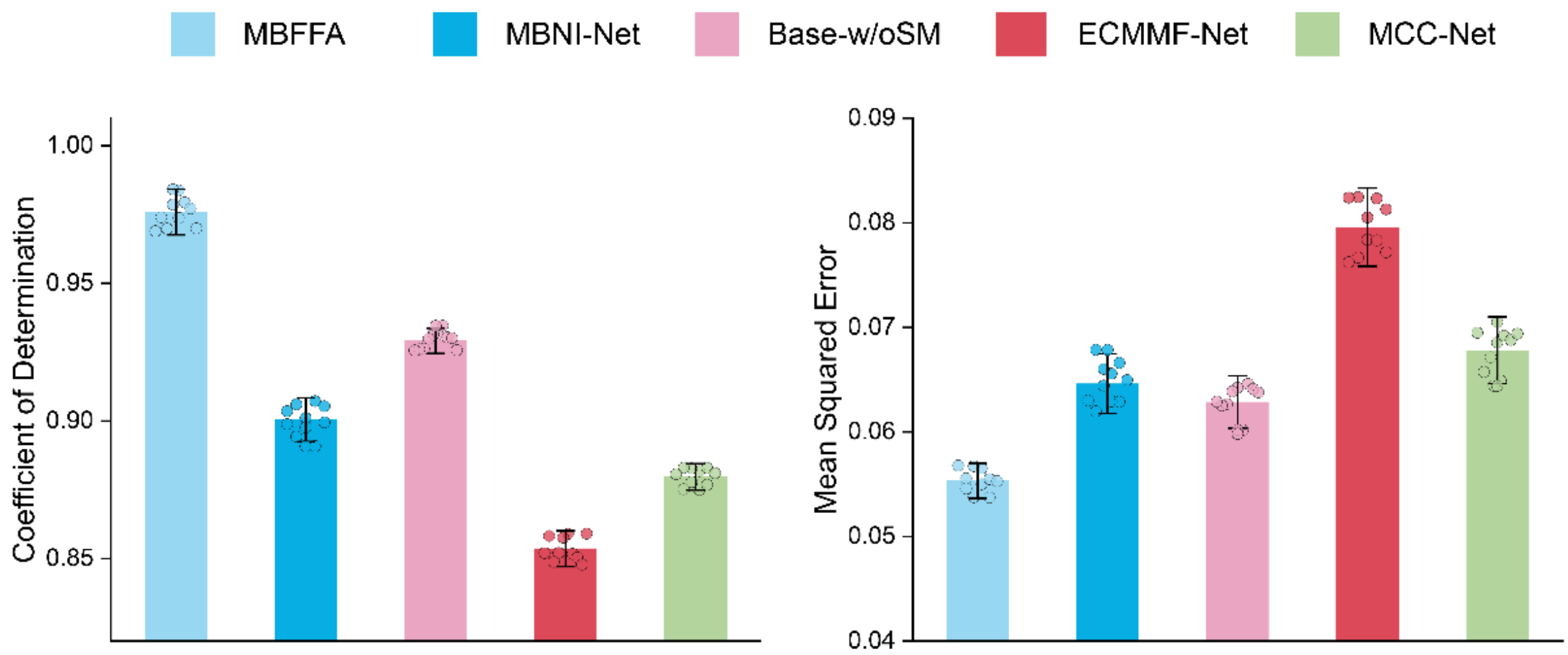

Supplementary Fig. 37 | Model generalization and overfitting assessment based on 10-fold cross-validation. The left and right panels show the coefficient of determination (R^2) and mean squared error (MSE), respectively. Under the fixed 8:1:1 split, 10-fold cross-validation was conducted to evaluate performance stability across data partitions. Error bars denote standard deviation across folds. Samples from the same raw region were grouped to prevent spatial information leakage, and each fold included all sample types in the test set. MBFFA consistently outperformed all benchmarks, achieving the highest R^2 and lowest MSE with the narrowest variance across folds. In contrast, Base-w/oSM and MBNI-Net showed inferior performance, while ECMMF-Net and MCC-Net exhibited both lower accuracy and higher instability. The consistent model ranking across all folds indicates that the results are independent of specific data splits. These findings suggest that MBFFA possesses robust generalization and remains free of significant overfitting despite high-dimensional inputs.

Supplementary Table 12

Supplementary Table 12 | Statistical comparison of model performance. Metrics include MSE (×10^{-2}) and MAE (×10^{-1}) across six specimens at strain amplitudes of 2 %, 4 %, 6 %, and 8 %. Best and second-best results are bolded and underlined, respectively. Our model achieves superior overall accuracy with an average rank of 1.563. Ablation of the feature synergy module (Base-w/oSM) leads to increases in MSE and MAE of 6.45 × 10^{-3} and 3.78 × 10^{-2}, representing performance degradations of 11.8 % and 22.6 %, respectively. This underscores the critical role of the synergy module in enhancing predictive reliability

Models		MBFFA		Base-w/oSM		MBNI-Net		MCC-Net		ECMMF-Net	
Metric		MSE	MAE	MSE	MAE	MSE	MAE	MSE	MAE	MSE	MAE
Specimen I	2	**0.133**	**0.264**	0.143	0.291	0.162	0.336	0.188	0.387	0.195	0.442
	4	2.745	**1.330**	**2.622**	1.446	3.486	1.850	3.187	1.738	4.450	2.109
	6	7.377	2.238	**7.350**	**2.100**	9.477	2.953	10.831	3.291	12.201	3.493
	8	**10.365**	**2.558**	13.235	3.638	11.749	3.063	14.827	3.851	14.636	3.826
Specimen II	2	**0.130**	**0.256**	0.148	0.324	0.177	0.359	0.160	0.338	0.246	0.496
	4	2.830	1.367	3.215	1.728	**2.756**	**1.294**	3.194	1.704	4.966	2.228
	6	8.119	2.314	**7.926**	**2.188**	8.863	2.894	9.860	3.068	13.679	3.699
	8	**10.695**	**2.643**	13.046	3.477	11.235	2.950	14.138	3.576	14.048	3.748
Specimen III	2	0.137	0.278	0.166	0.338	0.135	**0.248**	**0.125**	0.354	0.221	0.468
	4	2.929	1.417	3.233	1.754	2.962	1.554	**2.718**	**1.303**	5.302	2.303
	6	**7.949**	**2.410**	9.856	3.139	9.902	3.147	8.598	2.434	12.973	3.602
	8	**11.108**	2.742	13.590	3.686	11.353	**2.624**	15.265	3.907	14.920	3.863
Specimen IV	2	0.144	0.281	**0.127**	**0.276**	0.150	0.319	0.152	0.334	0.277	0.526
	4	3.021	1.459	3.331	1.798	3.138	1.682	**2.829**	**1.364**	7.327	2.593
	6	**8.198**	2.480	9.196	**2.408**	9.112	2.971	8.904	2.720	11.187	3.345
	8	11.445	2.824	12.852	3.585	**11.195**	**2.662**	13.141	3.625	14.697	3.834
Specimen V	2	0.154	**0.297**	**0.138**	0.318	0.144	0.324	0.208	0.456	0.268	0.518
	4	3.153	**1.526**	**2.957**	1.629	3.015	1.607	3.336	1.802	7.405	2.668
	6	**8.603**	**2.600**	8.872	2.805	10.154	2.919	10.703	3.272	11.510	3.393
	8	11.973	**2.850**	**11.091**	3.096	15.621	3.952	14.176	3.588	14.310	3.783
Specimen VI	2	0.118	0.241	0.171	0.372	0.122	0.251	**0.116**	**0.227**	0.237	0.487
	4	**2.611**	**1.263**	3.187	1.785	3.636	1.898	4.240	2.059	4.699	2.168
	6	**6.965**	**2.130**	8.972	2.871	9.726	2.661	10.339	3.148	11.818	3.438
	8	**9.828**	**2.437**	10.785	2.948	15.509	3.763	14.310	3.783	12.707	3.565
Avg Rank		**1.562**		2.458		2.833		3.375		4.771	

Supplementary Figure 38

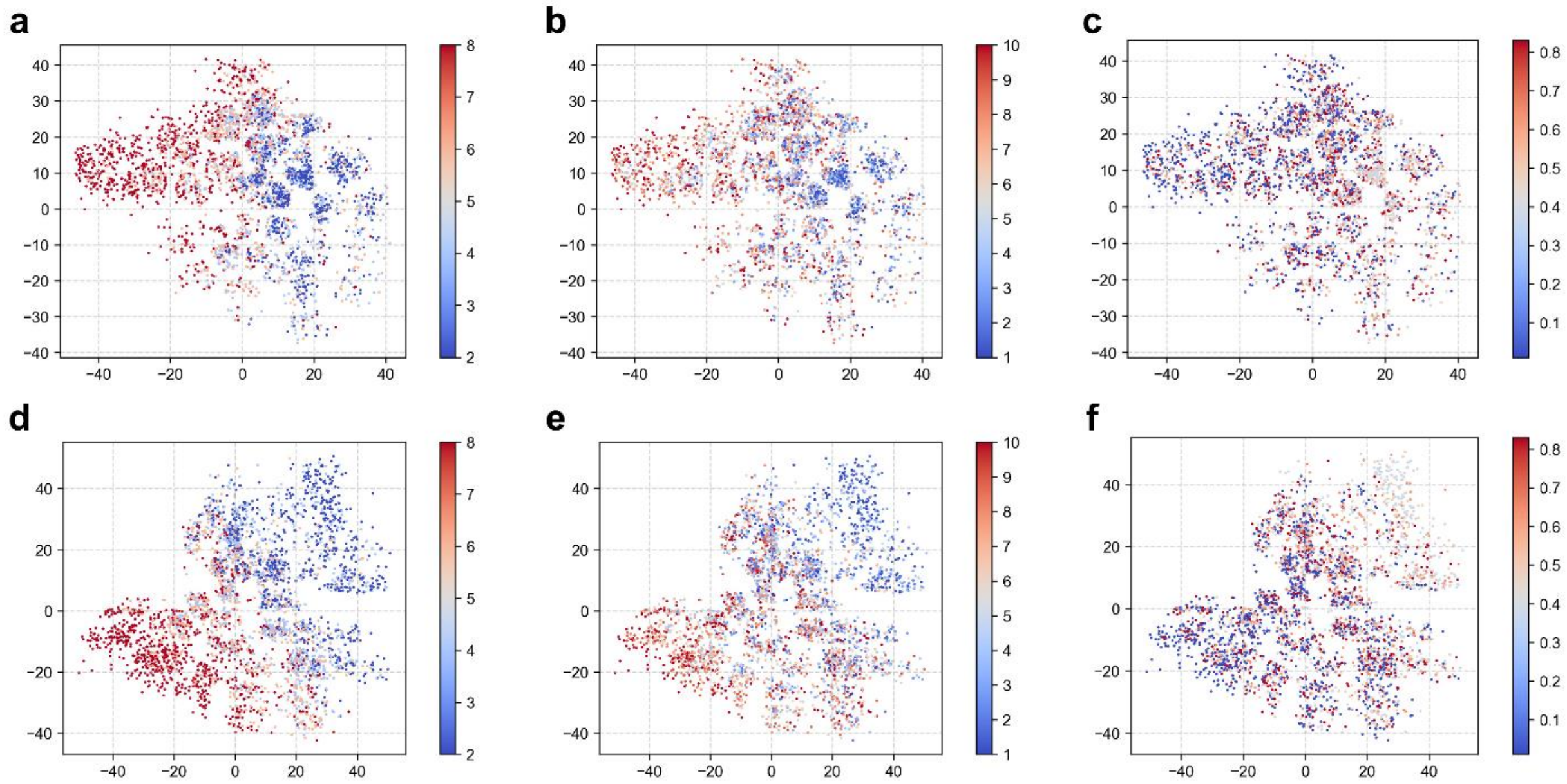

Supplementary Fig. 38 | UMAP visualization of latent spaces before and after feature synergy. Panels **a–c** and **d–f** illustrate the latent representations prior to and following feature fusion, respectively. Clusters are colored by strain amplitude (**a, d**), cycle number (**b, e**), and the Ni_4Ti_3 descriptor (**c, f**). Strain amplitude exhibits the most continuous and well-defined spatial gradient, closely aligning with the residual strain distribution and underscoring its primary role in latent space organization. While cycle number displays a discernible trend, its spatial separation is less pronounced. Notably, samples with intermediate Ni_4Ti_3 characteristic values form distinct local regions, whereas high and low values show increased clustering or spatial proximity. This distribution is consistent with the experimental observation that both coarsened precipitates and precipitate-free conditions accelerate superelastic degradation. After fusion, the latent space maintains a stable yet more compact topology, indicating that the synergy module effectively enhances the integrated representation of multimodal inputs.

Supplementary Figure 39

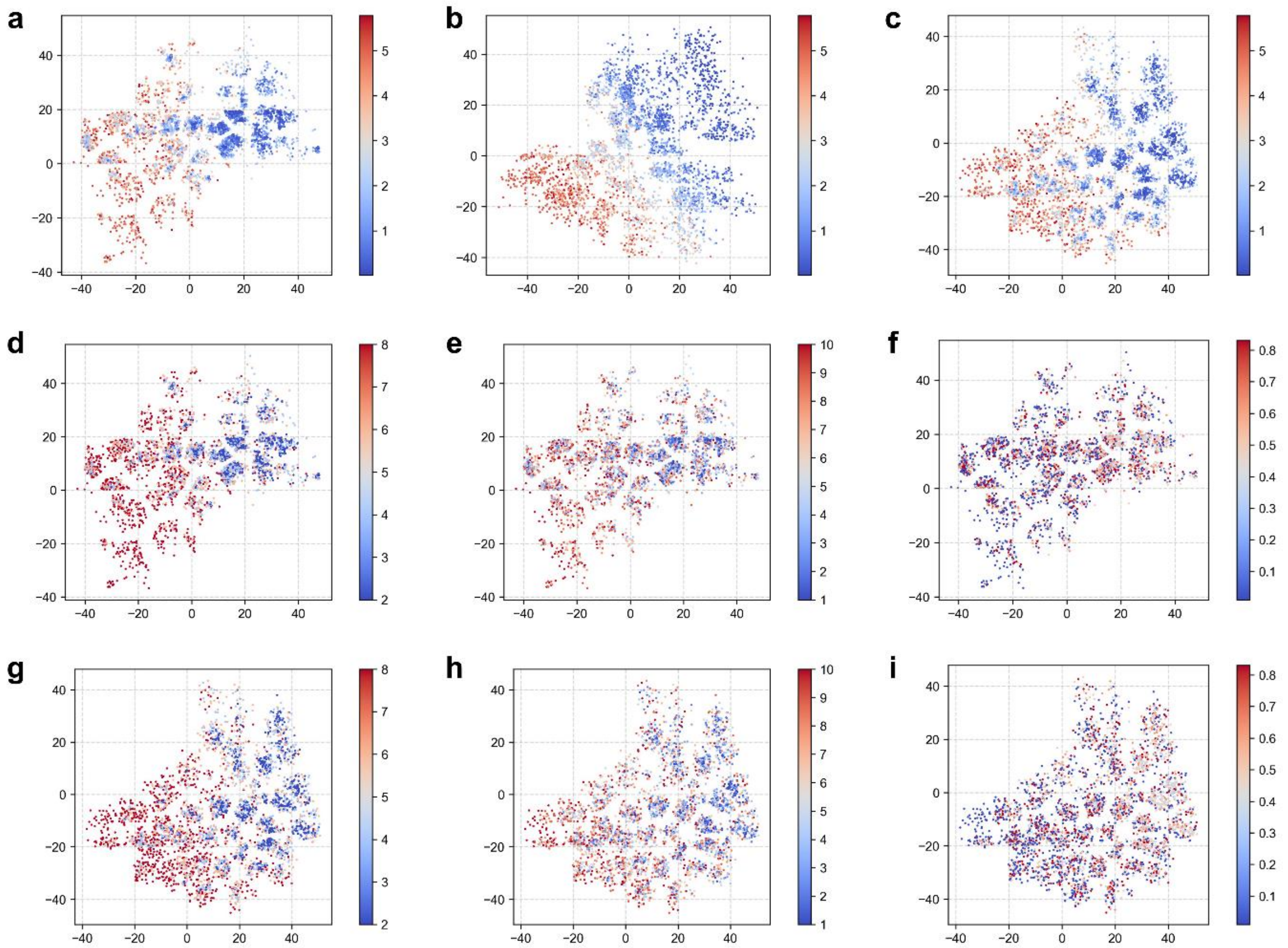

Supplementary Fig. 39 | UMAP manifold evolution and multi-physical mapping across feature dimensions. This figure evaluates how the number of latent feature representations (n = 2, 3, 4) extracted by the convolutional branch influences latent space organization. **a–c** show embeddings colored by residual strain for n = 2, 3, and 4, respectively. **d–f** and **g–i** illustrate latent spaces for n = 2 and n = 4 mapped against strain amplitude, cycle number, and Ni_4Ti_3 descriptor. As shown in **a–c**, n = 3 yields the most continuous residual strain gradient, characterized by smooth transitions and minimal overlap. Conversely, n = 2 yields a compressed distribution with significant mixing in transition zones. Combined with **d–f**, it is evident that while strain amplitude forms a dominant partition, information regarding cycle number and Ni_4Ti_3 remains insufficiently resolved, limiting representational capacity. At n = 4, the embedding becomes fragmented and disjointed, with similar residual strain values dispersed across isolated clusters. Although the global structure governed by strain amplitude is preserved (as seen in **g–i**), local variations associated with cycle number and Ni_4Ti_3 are amplified, suggesting that redundant features degrade representational stability.

Supplementary Figure 40

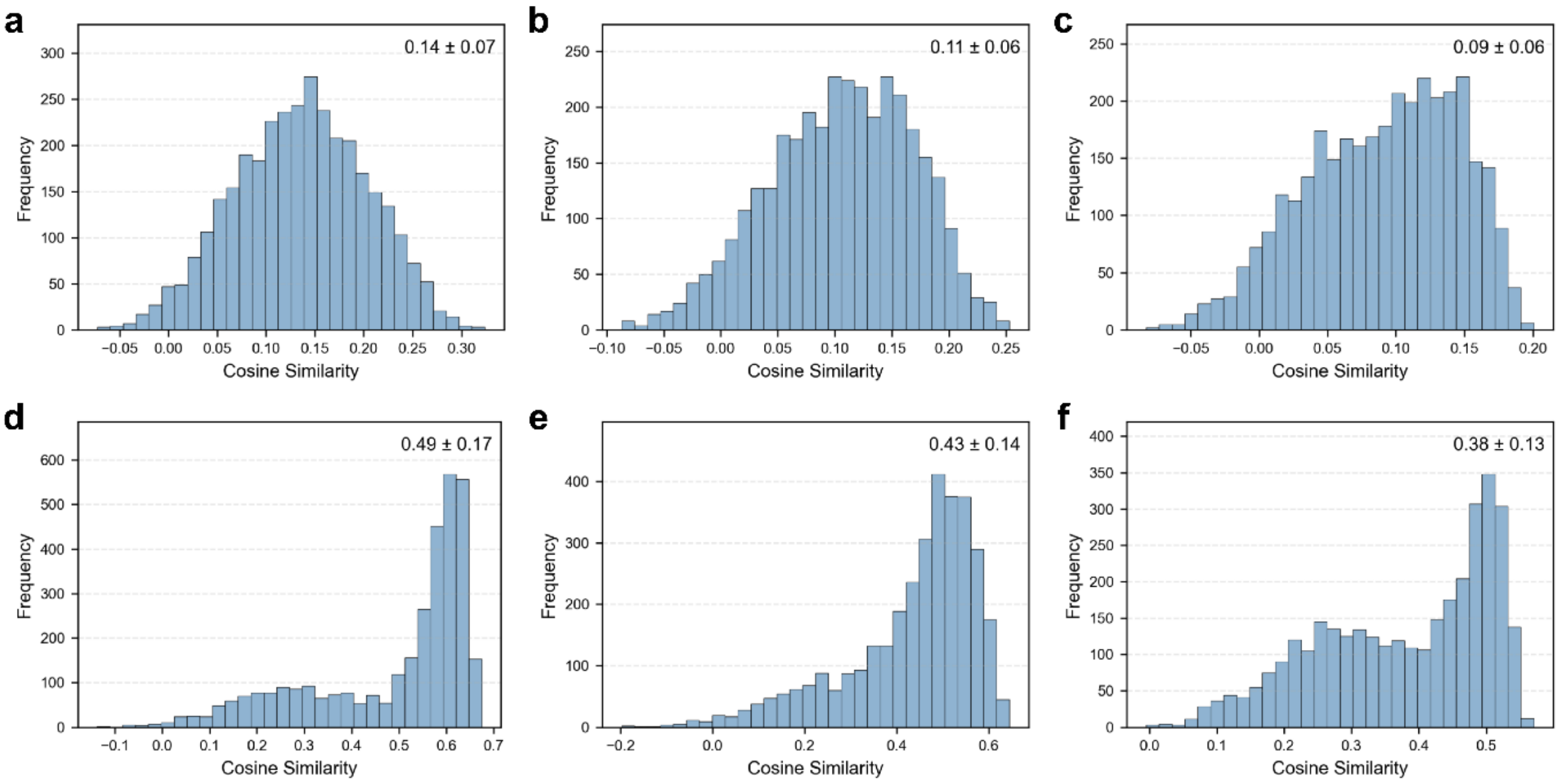

Supplementary Fig. 40 | Evolution of cosine similarity distributions for multimodal features in the latent space. Pairwise cosine similarities[31] were calculated from training samples for convolutional features of three image modalities: Euler, Phase, and GND. **a–c** show the distributions for Euler–GND, Euler–phase, and GND–phase pairs before the synergy module; **d–f** present the corresponding results after fusion. Before fusion, mean similarities range between 0.09 and 0.14, suggesting strong independence between modalities; the marginally higher Euler–GND similarity points to an intrinsic correlation between crystallographic orientation and localized lattice distortion. Post-fusion, the distributions shift significantly toward higher intervals (0.38–0.49), indicating markedly enhanced cross-modal alignment and successful extraction of shared information. Notably, the distributions maintain a finite width, suggesting that modalities are not compressed into homogeneous spaces. Instead, the synergy module establishes robust inter-modal coupling while preserving the distinct characteristics of each modality, effectively mitigating representation collapse.

Supplementary Figure 41

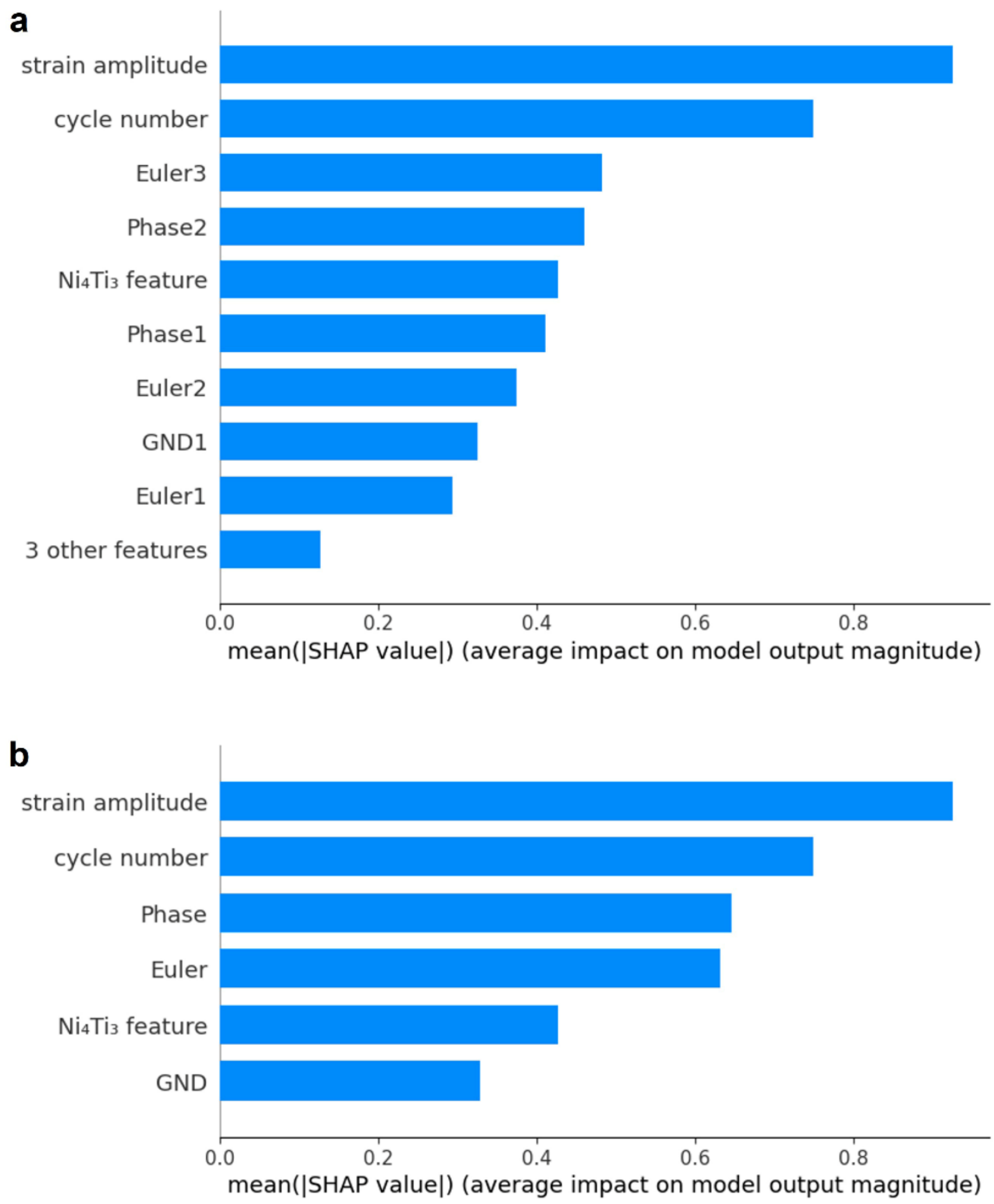

Supplementary Fig. 41 | Global interpretability of residual strain prediction based on SHAP analysis. a Mean absolute SHAP values of individual latent features, ranked by their contributions to the model output. **b** Grouped mean SHAP values aggregated at the modality level. At both levels, strain amplitude and cycle number dominate the prediction, highlighting the primary role of loading conditions in superelastic degradation. Among microstructural descriptors, orientation-related features and phase information contribute moderately. Specifically, the Ni_4Ti_3 feature captures essential precipitate effects, whereas the GND density exhibits a localized yet discernible impact associated with lattice distortion.

Supplementary Figure 42

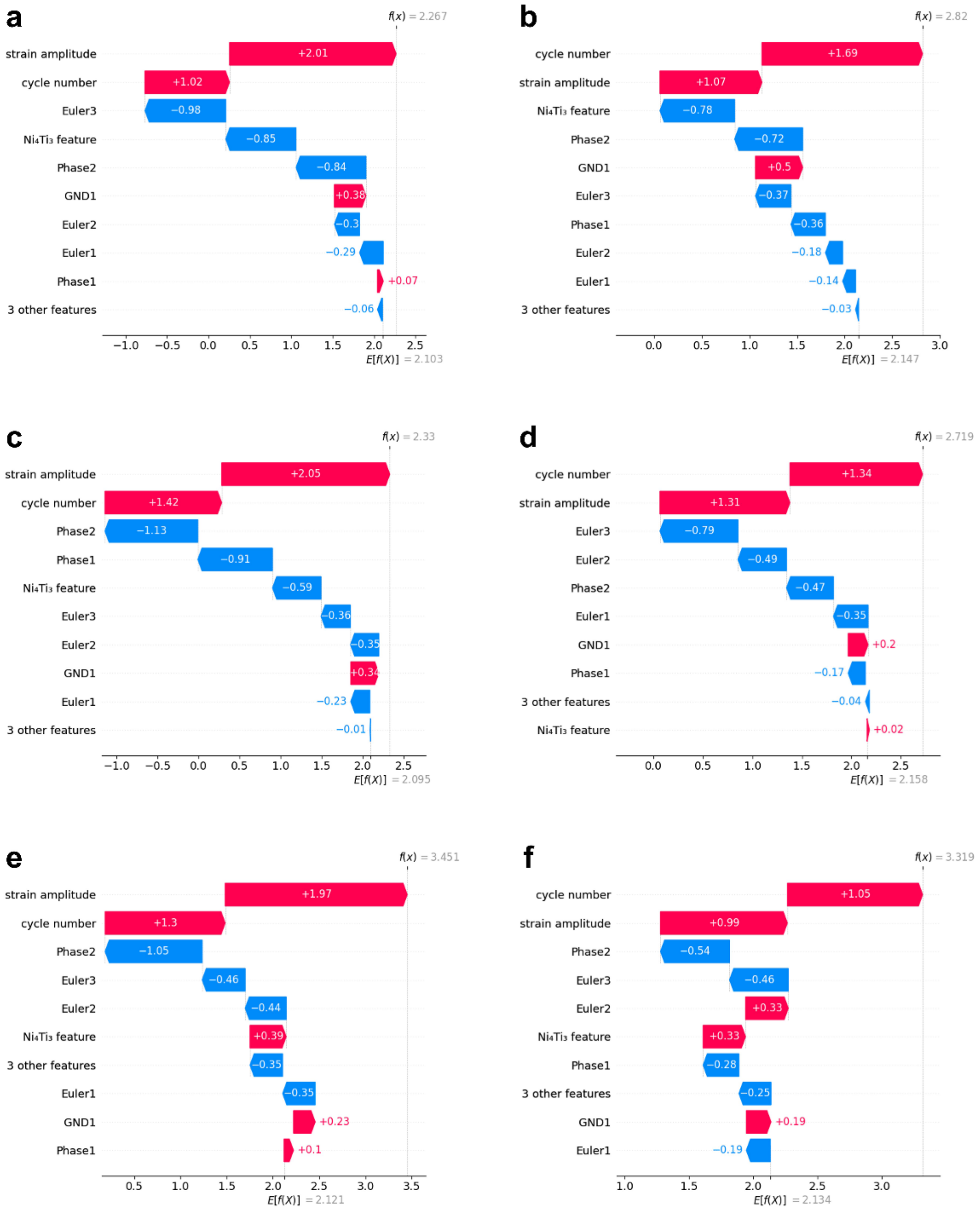

Supplementary Fig. 42 | Local interpretability of residual strain predictions across six annealing conditions. Results are shown for specimens I–VI at 6% strain after 10 cycles. **a–f** SHAP waterfall plots detailing feature attributions for each specimen. Overall, strain amplitude and cycle number act as the dominant positive contributors across all specimens. The Ni_4Ti_3 feature generally provides a strong negative contribution, confirming that fine, dispersed precipitates mitigate degradation. In contrast, the contribution of the GND1 feature is more pronounced in lightly annealed specimens and decreases at higher annealing intensities. This trend suggests that GND1 effectively captures localized strain concentrations that gradually relax during annealing.

Supplementary Figure 43

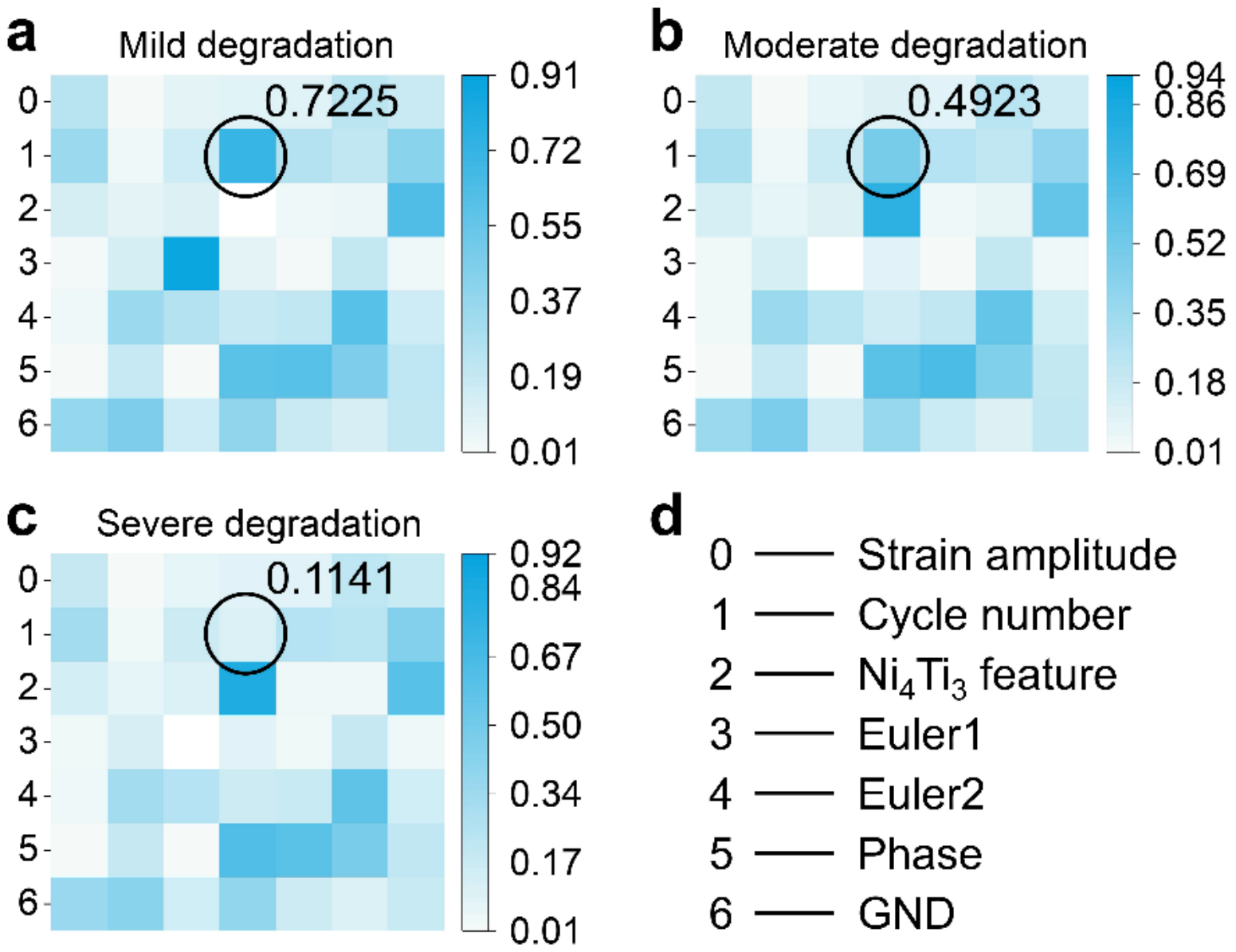

Supplementary Fig. 43 | Feature interaction matrices across different levels of superelastic degradation. a–c Interaction matrices for mild, moderate, and severe degradation, with colors indicating normalized intensity. The coupling between cycle number and orientation features (highlighted by black circles) exhibits a systematic reduction, with magnitudes decreasing from 0.7225 to 0.4943 and finally to 0.1141. This decline indicates that the impact of initial crystallographic orientation is gradually superseded by cyclically accumulated damage and irreversible strain. As degradation intensifies, the dependency of the response on orientation diminishes, weakening its interaction with the cycle number. **d** Reference table mapping matrix indices to specific features.

Supplementary Figure 44

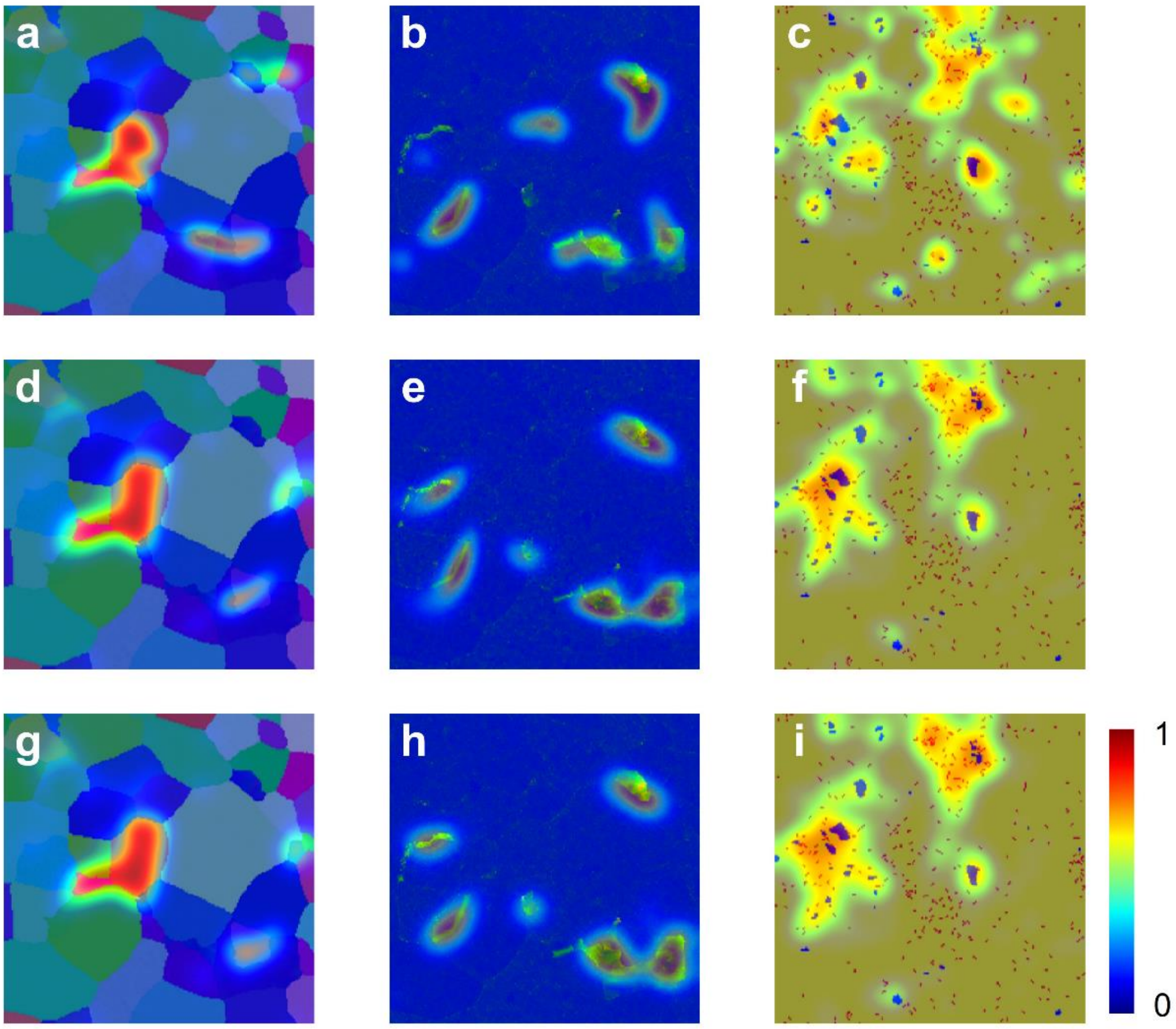

Supplementary Fig. 44 | Comparison of Grad-CAM visualizations under varied feature extraction strategies. a–c Heatmaps generated using the channel stacking strategy for orientation (IPF), GND, and phase maps. **d–f** Results using the previous method (KAM-based), corresponding to Euler, KAM, and phase maps, respectively; **g–i** Results from the proposed method. Comparison shows that channel stacking produces a dispersed response with poor feature correspondence. The KAM-based approach reflects orientation gradients but suffers from spatial misalignment. Conversely, the proposed GND-based method achieves well-defined boundaries and stable localization, capturing dislocation-induced lattice curvature and plastic incompatibility more effectively. Crucially, when individual modalities are activated in isolation, the transition from KAM to GND also modulates the response distributions of the phase and orientation channels, revealing a degree of coupling among modalities.

Supplementary Figure 45

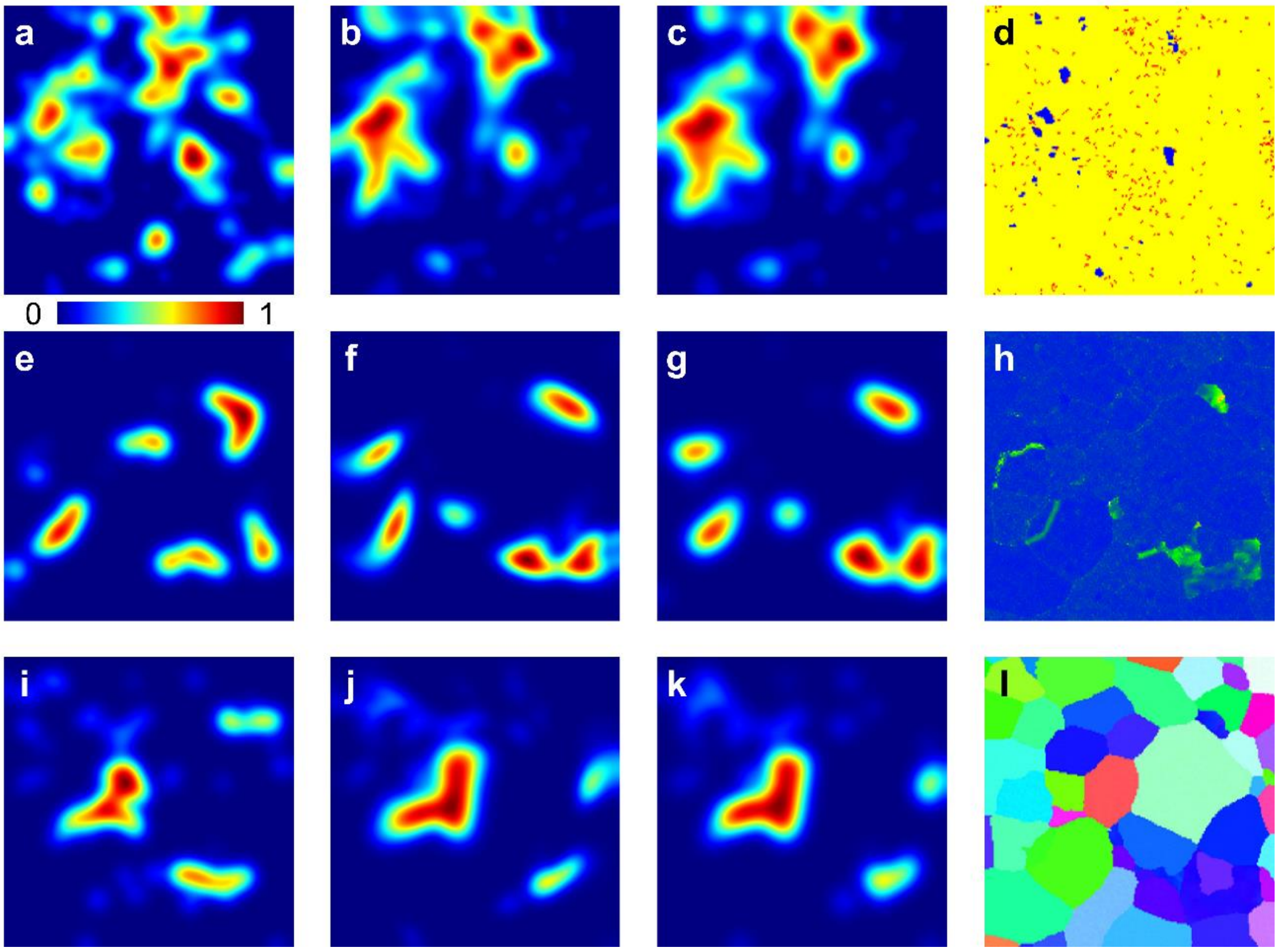

Supplementary Fig. 45 | Comparative Grad-CAM visualizations across multimodal inputs. a–d, **e–h**, and **i–l** illustrate spatial responses for phase, GND, and Euler modalities, respectively, under (**a, e, i**) channel stacking, (**b, f, j**) KAM-based, and (**c, g, k**) GND-based strategies. **d, h, l** provide the corresponding reference maps. Comparisons across modalities consistently show that the channel-stacking strategy yields relatively diffuse responses, whereas the KAM-based approach improves localization but still exhibits spatial deviation.

Supplementary Figure 46

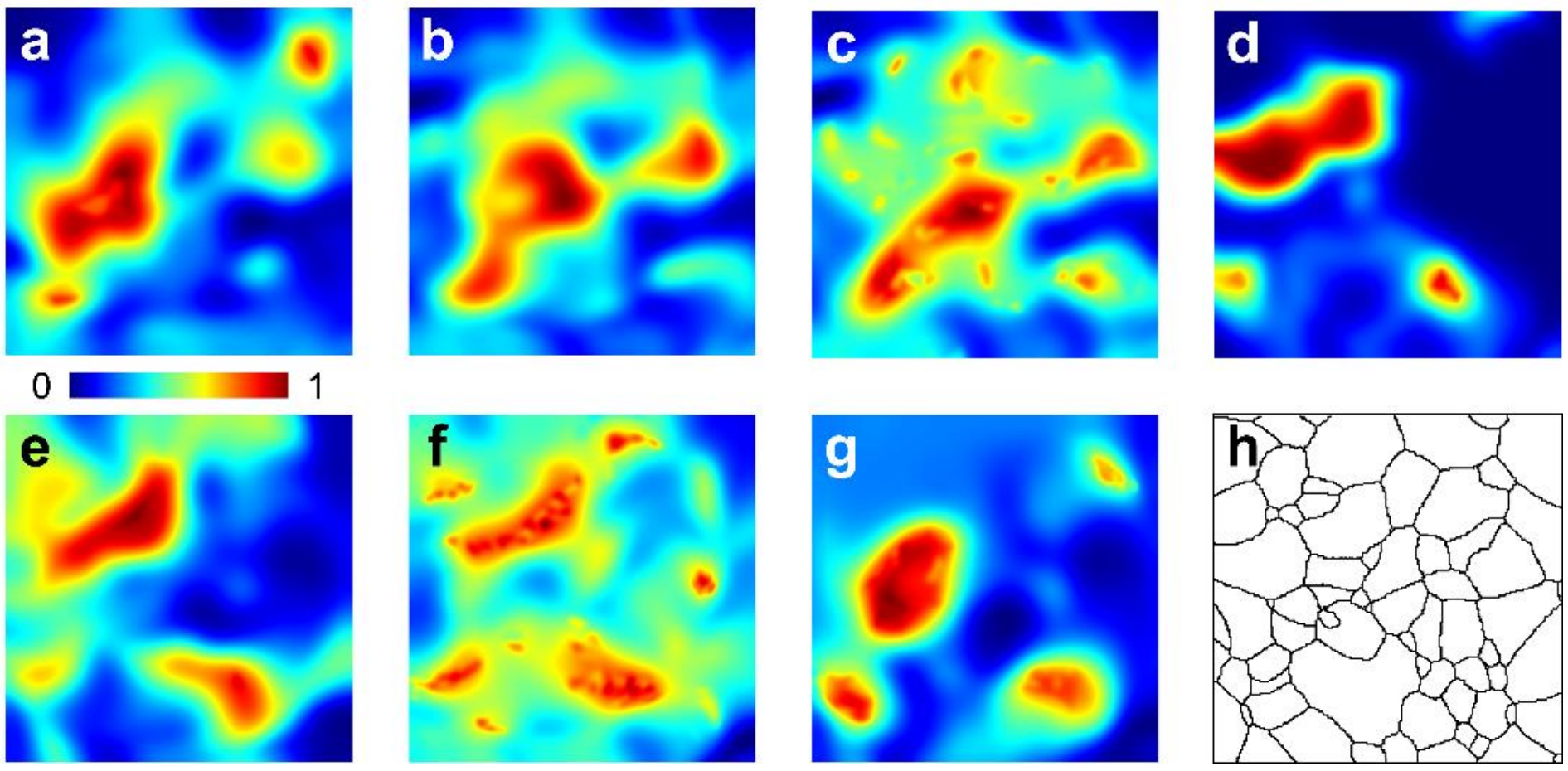

Supplementary Fig. 46 | Euler matrix response heatmaps. a–g Response heatmaps obtained by feature visualization of the Euler orientation matrices in the same region, corresponding to different strain amplitudes and cycle numbers: (**a**) 2%–1 cycle; (**b**) 2%–3 cycles; (**c**) 2%–10 cycles; (**d**) 8%–1 cycle; (**e**) 8%–3 cycles; (**f**) 8%–10 cycles; (**g**) 6.38%–1 cycle. **h** Grain boundary map of the same region. In **Fig. 4** of the main text, the heatmaps in (**a–g**) are spatially superimposed with the grain boundary contours in (**h**), and the high-response regions are further compared with the Schmid factor distribution to identify the grain orientations and associated deformation mechanisms that dominate superelastic degradation.

Supplementary Figure 47

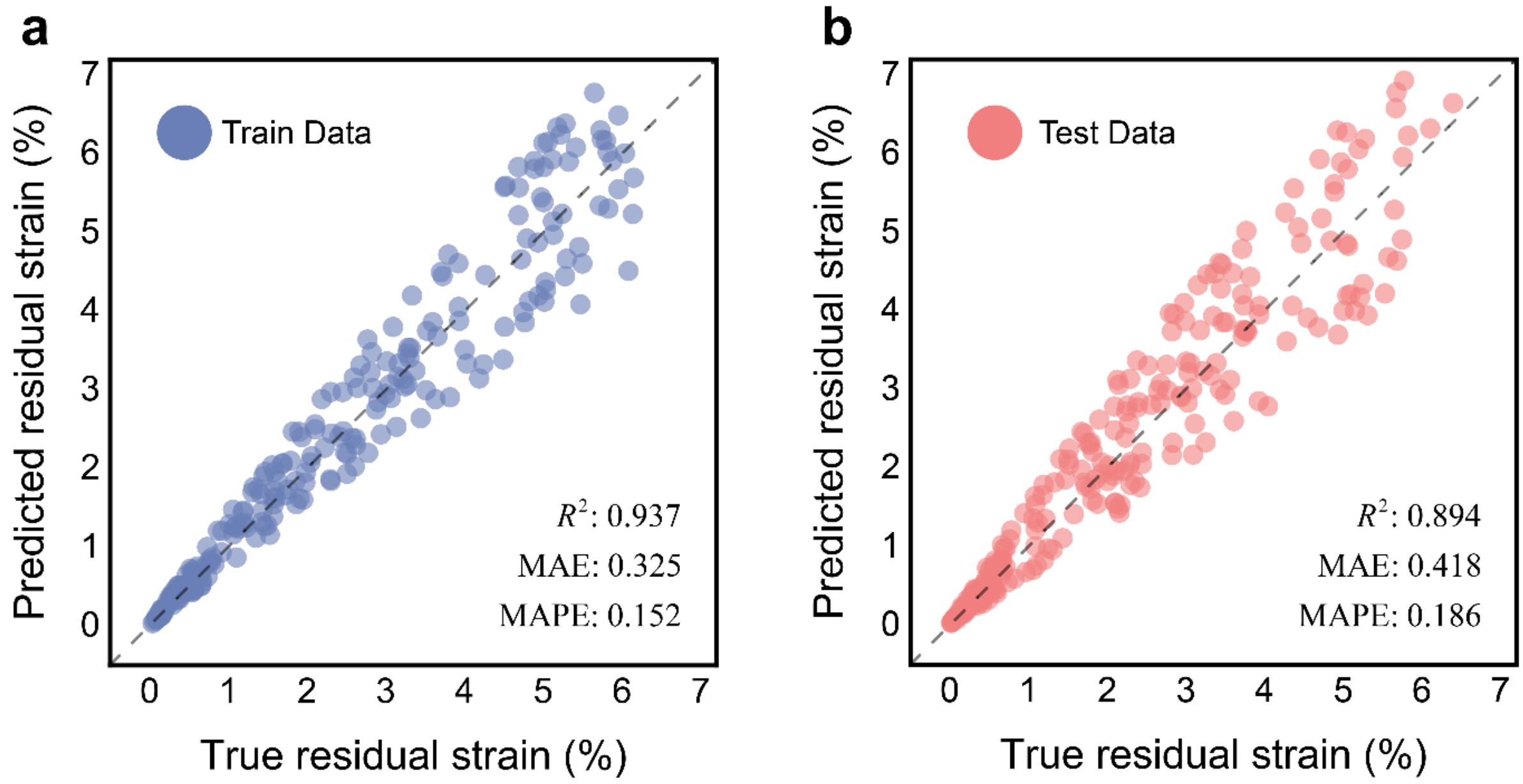

Supplementary Fig. 47 | Predictive performance of residual strain across varying strain rates. a, **b** Comparison between predicted and experimental residual strain for the (**a**) training and (**b**) test datasets at room temperature. Predicted residual strain is plotted against experimental values for cyclic tensile loading–unloading tests performed at four strain rates (5×10^{-4} s^{-1}, 1×10^{-3} s^{-1}, 1×10^{-2} s^{-1}, and 3×10^{-2} s^{-1}). Points indicate averaged training predictions and held-out test predictions from grouped tenfold cross-validation over the original labels. The dashed line denotes ideal agreement ($y = x$). Model performance is quantified by R^2, MAE, and MAPE, yielding 0.937, 0.325, and 0.152 for the training set, and 0.894, 0.418, and 0.186 for the test set, respectively.

Supplementary Figure 48

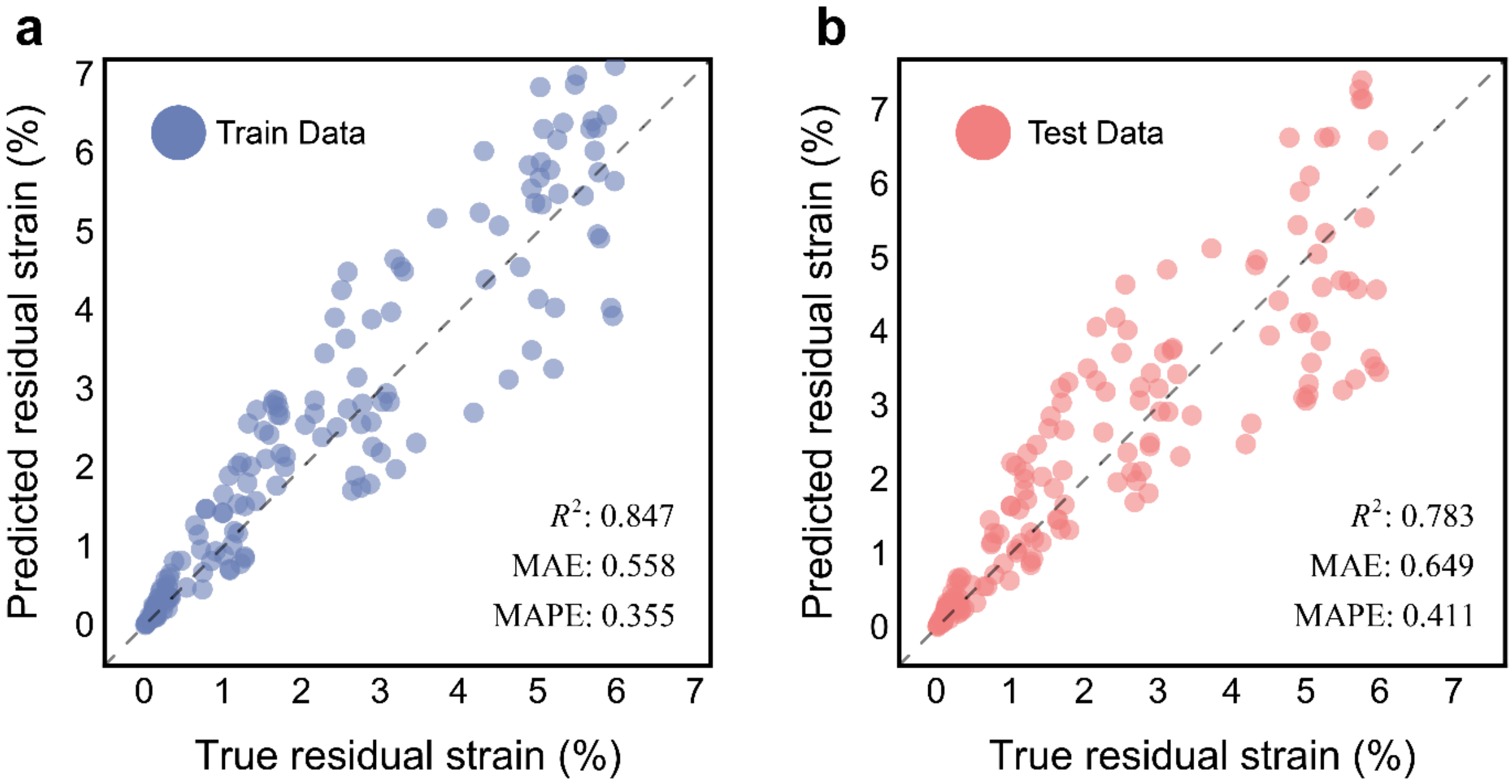

Supplementary Fig. 48 | Predictive performance of residual strain under different temperature conditions. a, **b** Comparison between predicted and experimental residual strain for the (**a**) training and (**b**) test datasets. The results are obtained from cyclic tensile loading–unloading tests conducted at room temperature and 40 °C under a quasi-static strain rate (5×10^{-4} s^{-1}). Points indicate averaged training predictions and held-out test predictions from grouped tenfold cross-validation over the original labels. The dashed line denotes ideal agreement ($y = x$). Model performance is quantified by R^2, MAE, and MAPE, yielding 0.847, 0.558, and 0.355 for the training set, and 0.783, 0.649, and 0.411 for the test set, respectively.

Supplementary Figure 49

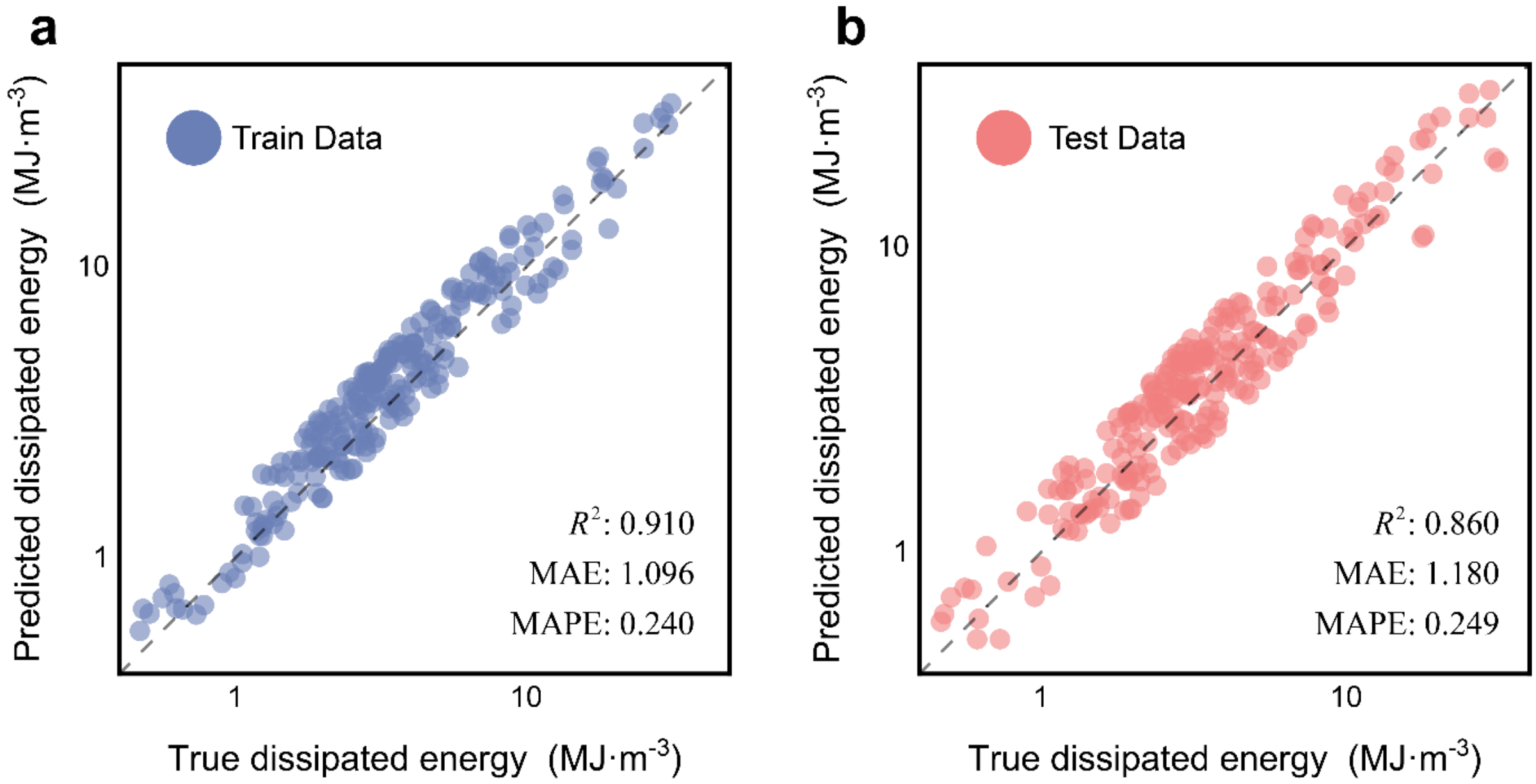

Supplementary Fig. 49 | Predictive performance of dissipated energy. a, **b** Comparison between predicted and experimental dissipated energy for the (**a**) training and (**b**) test datasets. Both axes are presented on a $\log_{10}$ scale. Points indicate averaged training predictions and held-out test predictions from grouped tenfold cross-validation over the original labels. The dashed line denotes ideal agreement ($y = x$). Model performance is quantified by R^2, MAE, and MAPE, yielding 0.910, 1.096, and 0.240 for the training set, and 0.860, 1.180, and 0.249 for the test set, respectively.

Supplementary Figure 50

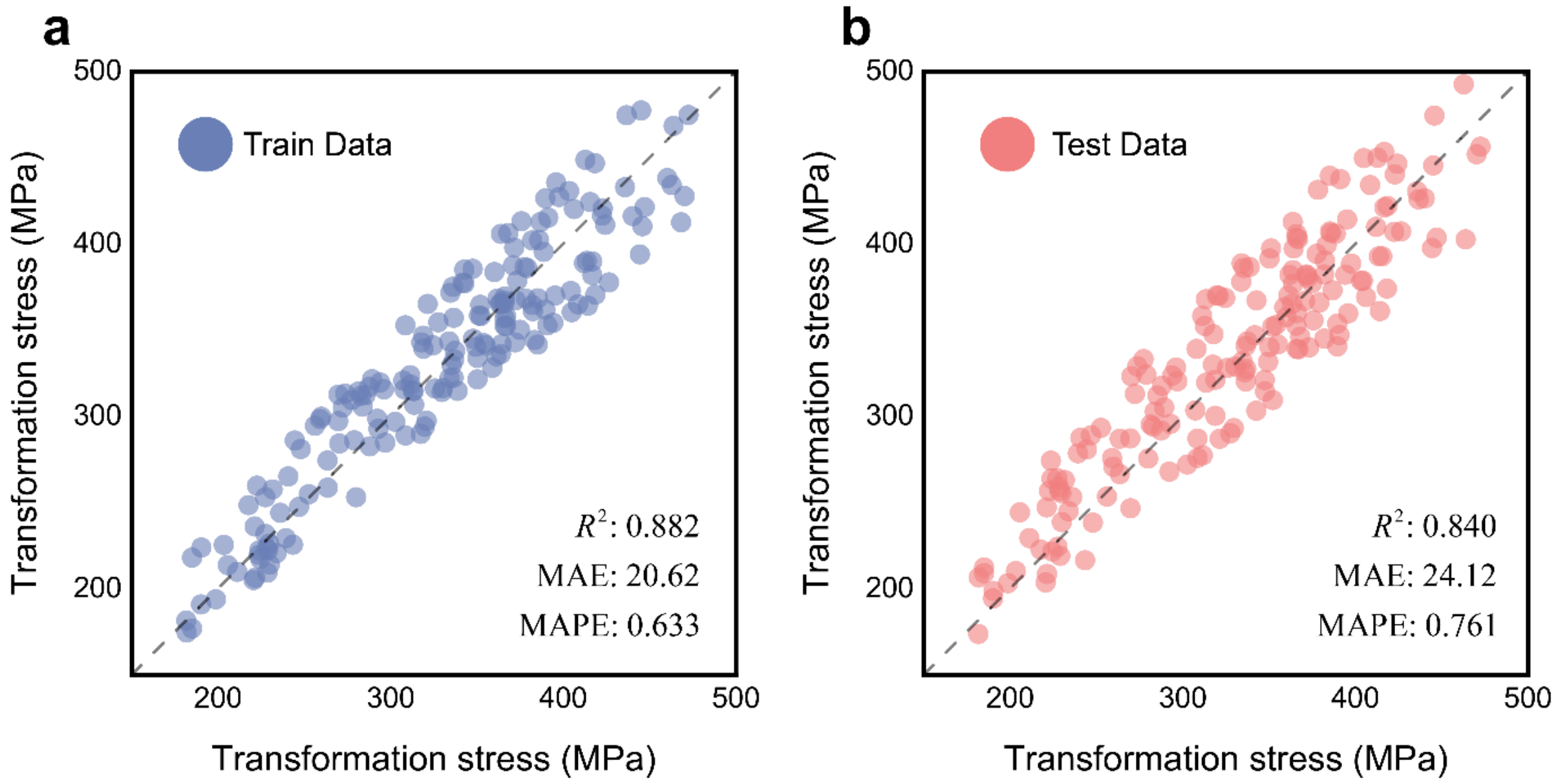

Supplementary Fig. 50 | Predictive performance of forward transformation start stress. **a**, **b** Comparison between predicted and experimental transformation stress for the (**a**) training and (**b**) test datasets. Points indicate averaged training predictions and held-out test predictions from grouped tenfold cross-validation over the original labels. The dashed line denotes ideal agreement ($y = x$). Model performance is quantified by R^2, MAE, and MAPE, yielding 0.882, 20.62, and 0.633 for the training set, and 0.840, 24.12, and 0.761 for the test set, respectively.

Supplementary Figure 51

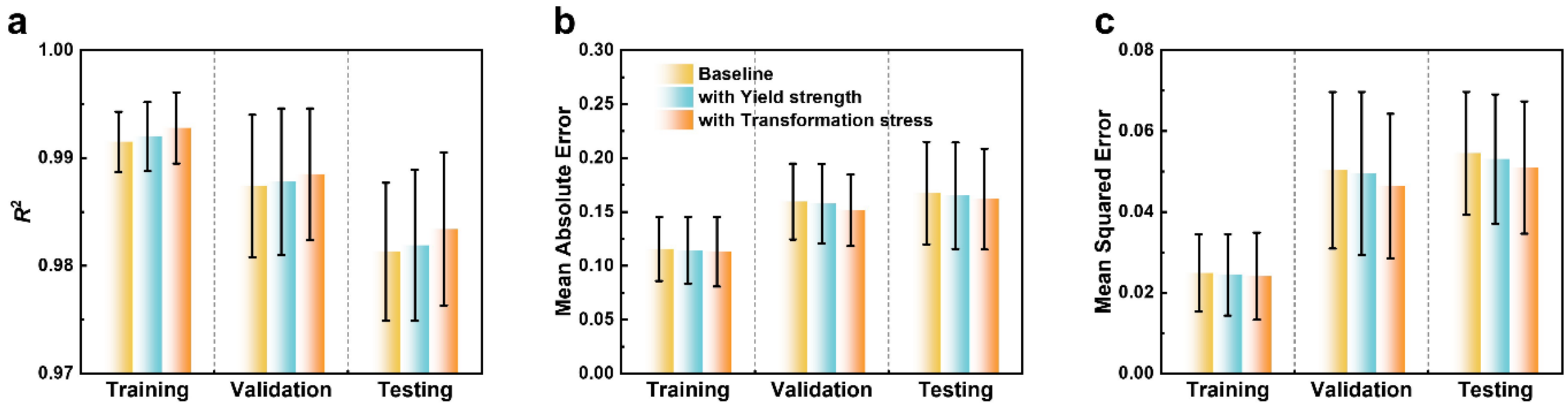

Supplementary Fig. 51 | Ablation study with additional mechanical descriptors. a-c Predictive performance metrics (R^2, MAE, and MSE) for models integrated with either conventional yield stress or forward transformation start stress. Bars and error bars represent the mean ± SD from n = 10 independent repeated experiments. Results are obtained by incorporating either the conventional yield stress (determined at 200 °C from the critical stress for austenite slip) or the transformation start stress as additional scalar inputs. The transformation stress is extracted from cyclic tensile loading–unloading curves, while the conventional yield stress is defined from the first loading cycle. Both descriptors facilitate incremental improvements in performance, with the transformation start stress providing a slightly greater benefit.

Supplementary Figure 52

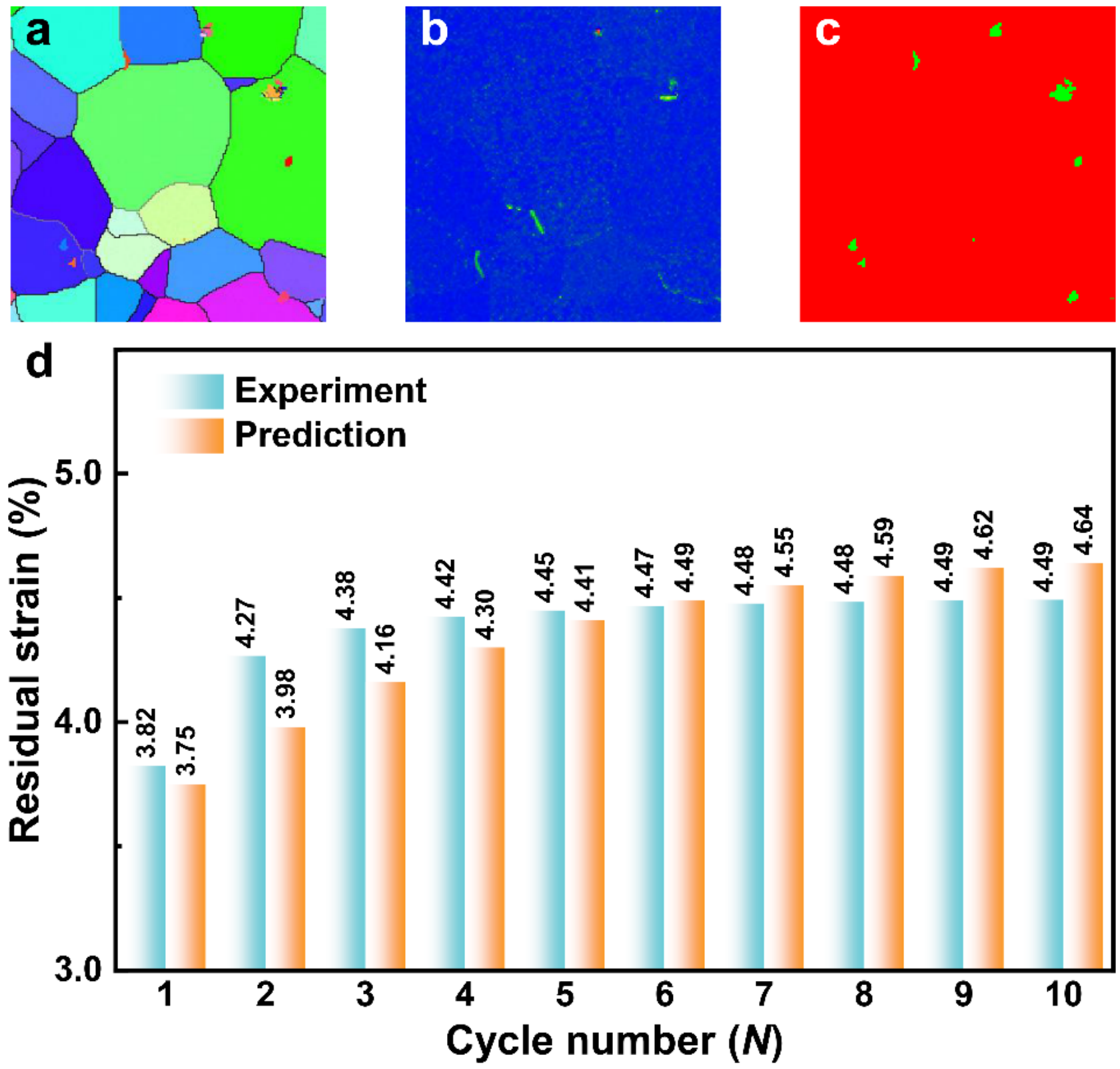

Supplementary Fig. 52 | Microstructural characterization and degradation prediction for the specimen annealed at 800 °C for 1 h. a Inverse pole figure (IPF) map exhibiting a fully recrystallized microstructure with a dominant <101> texture. **b** GND map illustrating a generally low dislocation density. **c** Phase map showing a B2 austenite matrix containing $NiTi_2$ precipitates. **d** Comparison between experimental and predicted residual strain under 6% strain amplitude over 10 cycles. Notably, this 800 °C / 1 h condition was excluded from the training set, demonstrating the robustness of the framework in capturing superelastic degradation trends of an unseen, fully annealed state.

Supplementary Method 5

Cyclic loading: After energy minimization and thermal equilibration, cyclic tensile loading-unloading was applied to characterize the structural evolution of the models under repeated deformation. Specifically, the system was gradually stretched along the loading direction from the initial state to a prescribed maximum engineering strain, then unloaded back to the initial state, thereby completing one cycle. This procedure was repeated for multiple cycles to compare the evolution of atomic configuration and the accumulation of local deformation at different cycling stages. The engineering strain was defined as $\varepsilon = (L - L_0)/L_0$, where L_0 and L are the initial and instantaneous box lengths along the loading direction, respectively. Atomic configurations were saved at the end of each cycle for subsequent structural analysis.

Atomic strain calculation: The atomic strain analysis was performed using the *Atomic Strain* method implemented in OVITO Pro 3.10.0 software[32]. Taking the initial configuration as the reference state, this method determines the local deformation gradient tensor **F** for each atom by comparing the relative positions of neighboring atoms between the reference and deformed configurations, such that the local affine deformation provides the best fit. The corresponding Green–Lagrange strain tensor is then calculated as $\mathbf{E}=1/2(\mathbf{F}^{\mathbf{T}}\mathbf{F}-\mathbf{I})$. Based on this approach, the local atomic strain distribution can be obtained and visualized, thereby characterizing the formation, propagation, and accumulation of high-strain regions during cyclic deformation. The resulting atomic strain maps were used to compare the strain localization behavior between the precipitate-free NiTi model and the NiTi model containing Ni_4Ti_3 precipitates.

Supplementary Figure 53

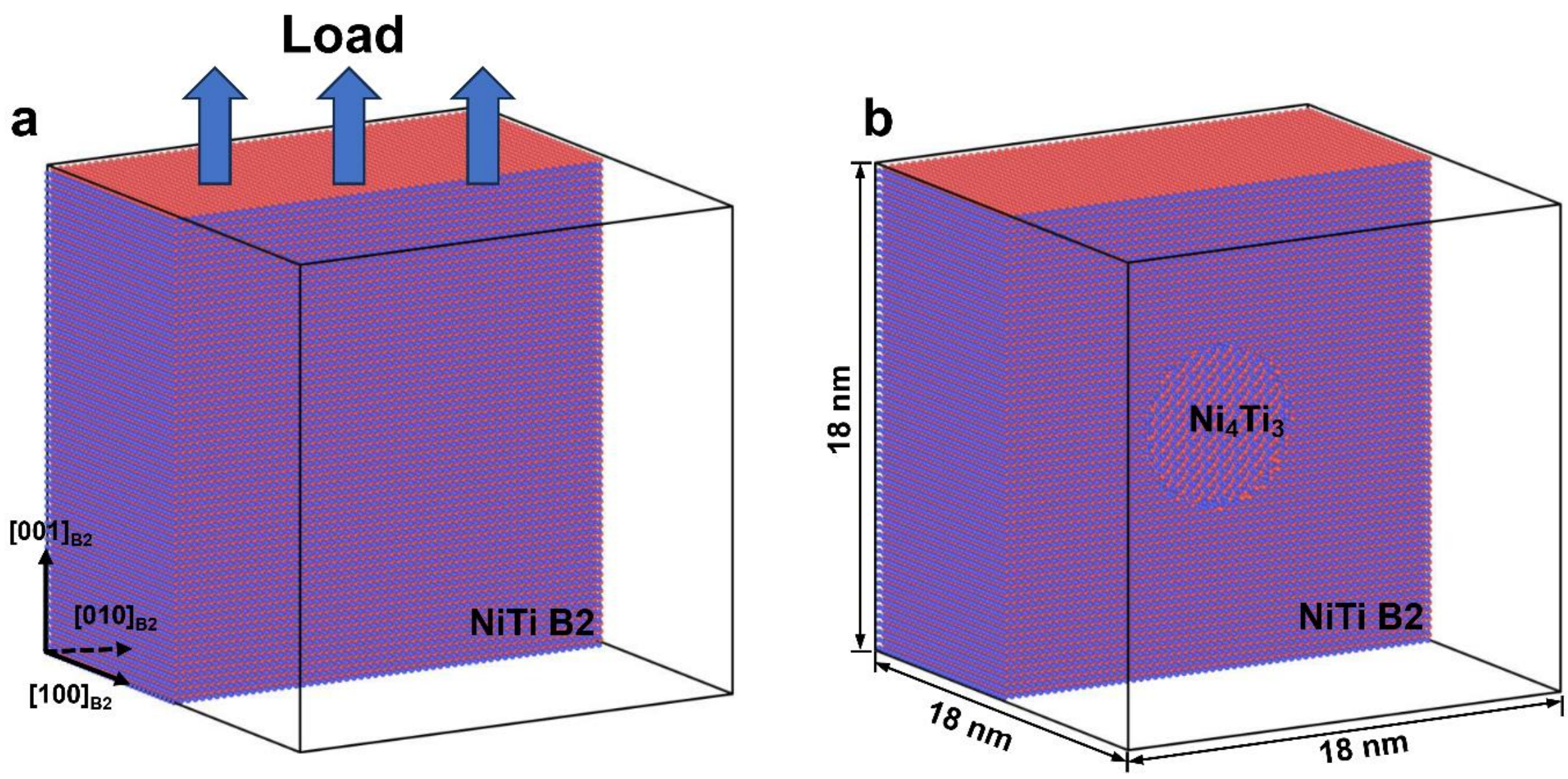

Supplementary Fig. 53 | Atomistic models used in this study (sliced at 1/2 along the x-axis). a NiTi without Ni_4Ti_3 precipitates. **b** NiTi with Ni_4Ti_3 precipitates.

Supplementary Figure 54

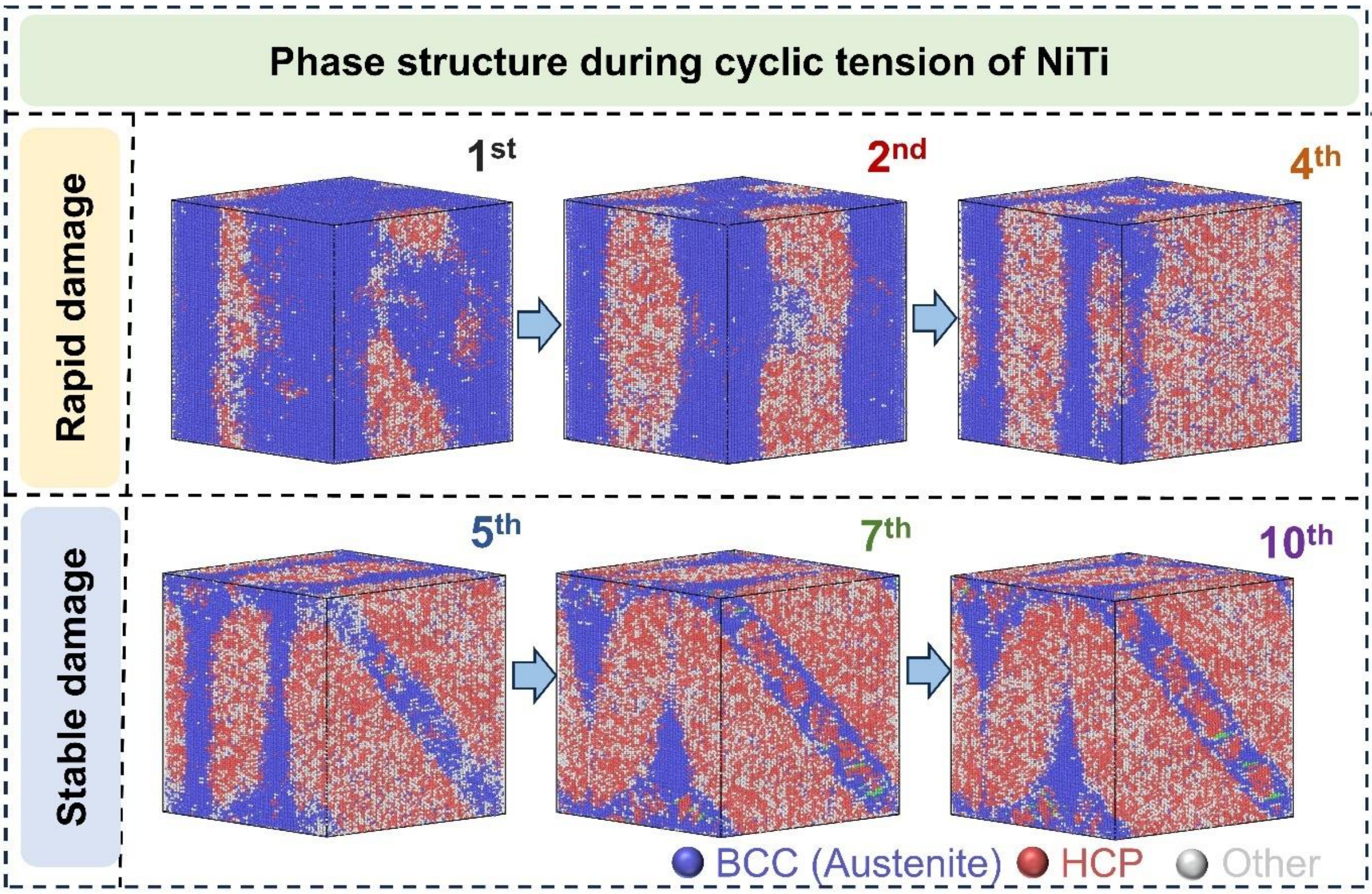

Supplementary Fig. 54 | Atomistic configurations of NiTi after each cycle in the cyclic simulation.

Supplementary Figure 55

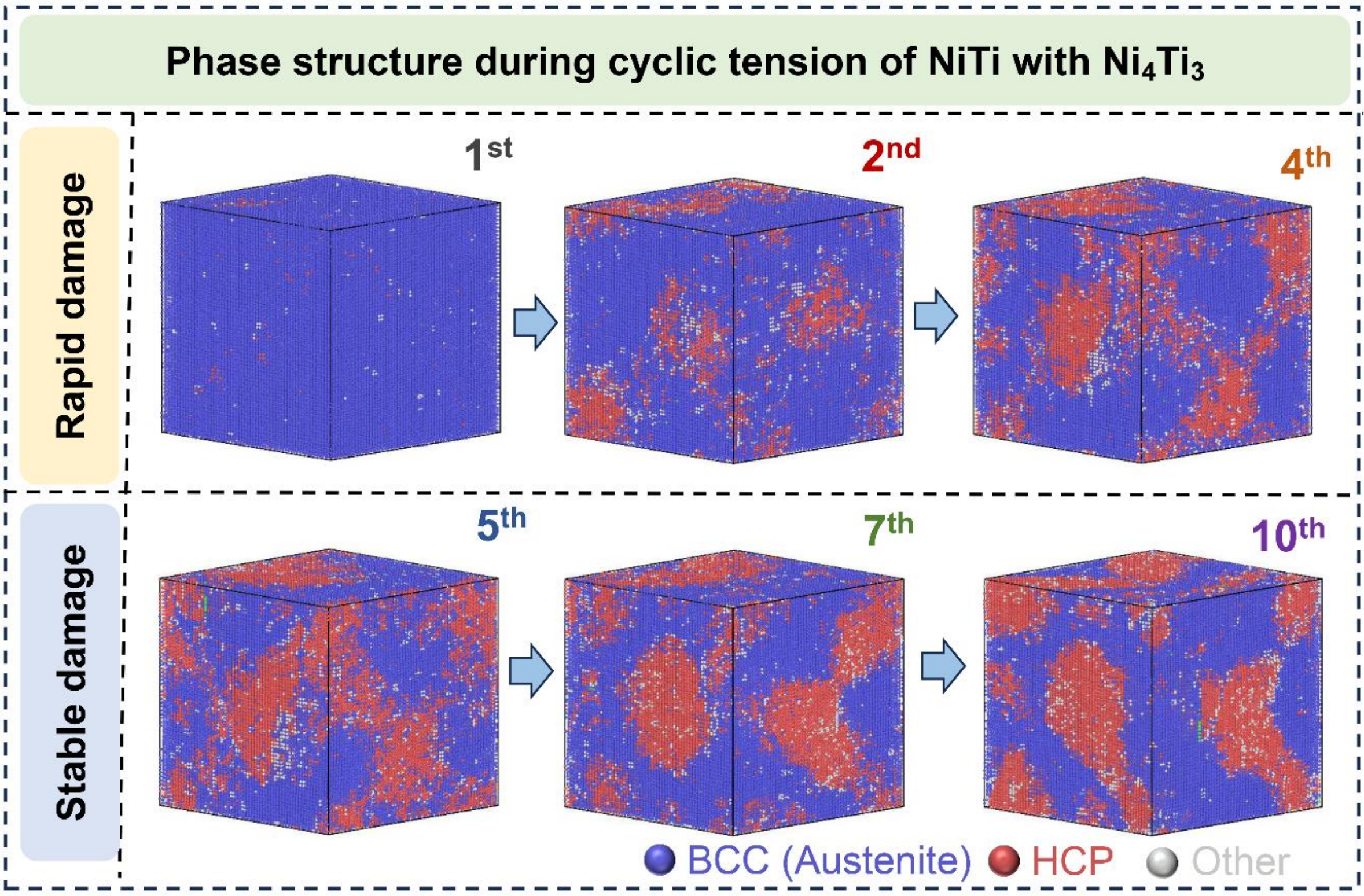

Supplementary Fig. 55 | Atomistic configurations of NiTi with Ni_4Ti_3 precipitates after each cycle in the cyclic simulation.

Supplementary Figure 56

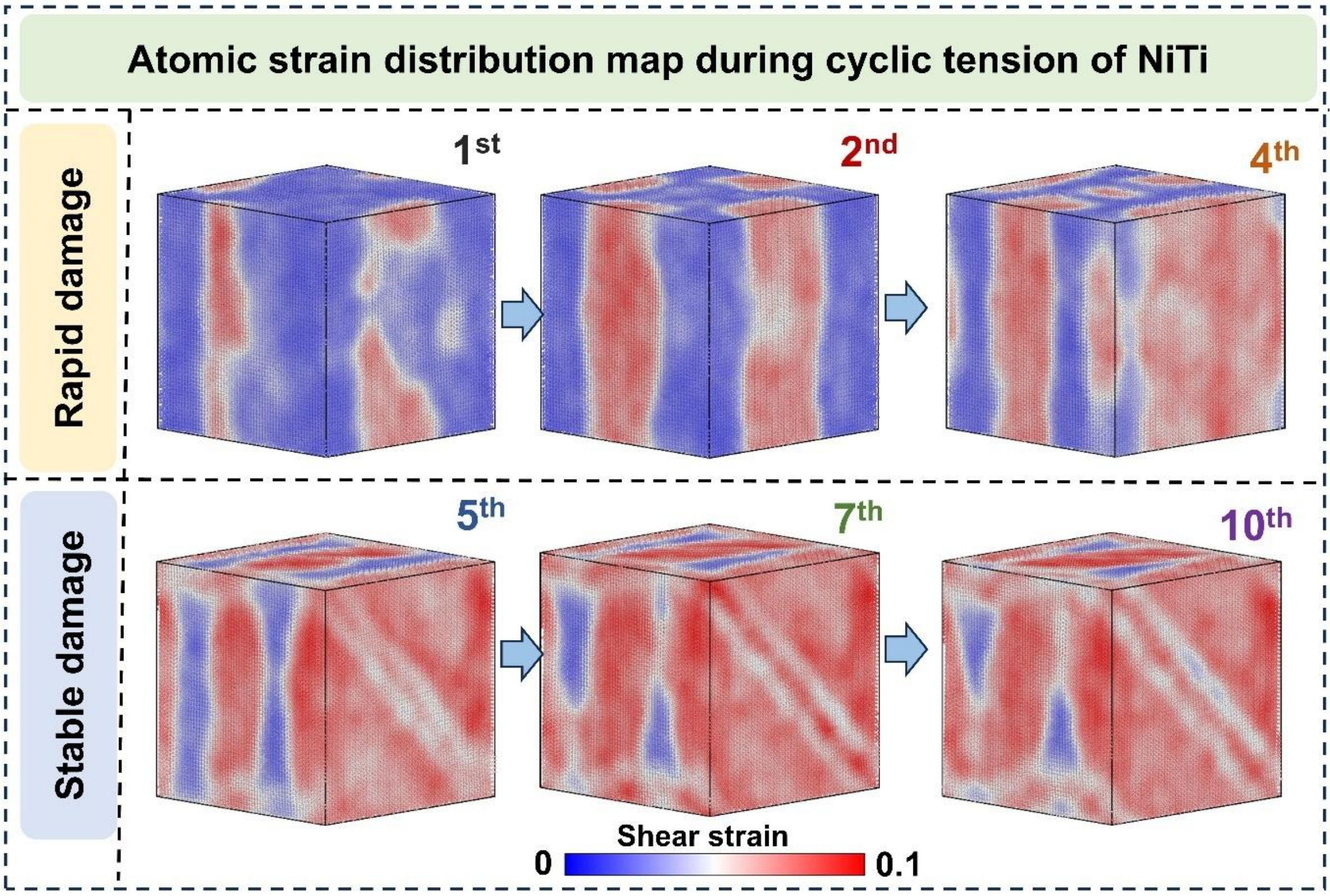

Supplementary Fig. 56 | Atomic strain maps of NiTi after each cycle in the cyclic simulation.

Supplementary Figure 57

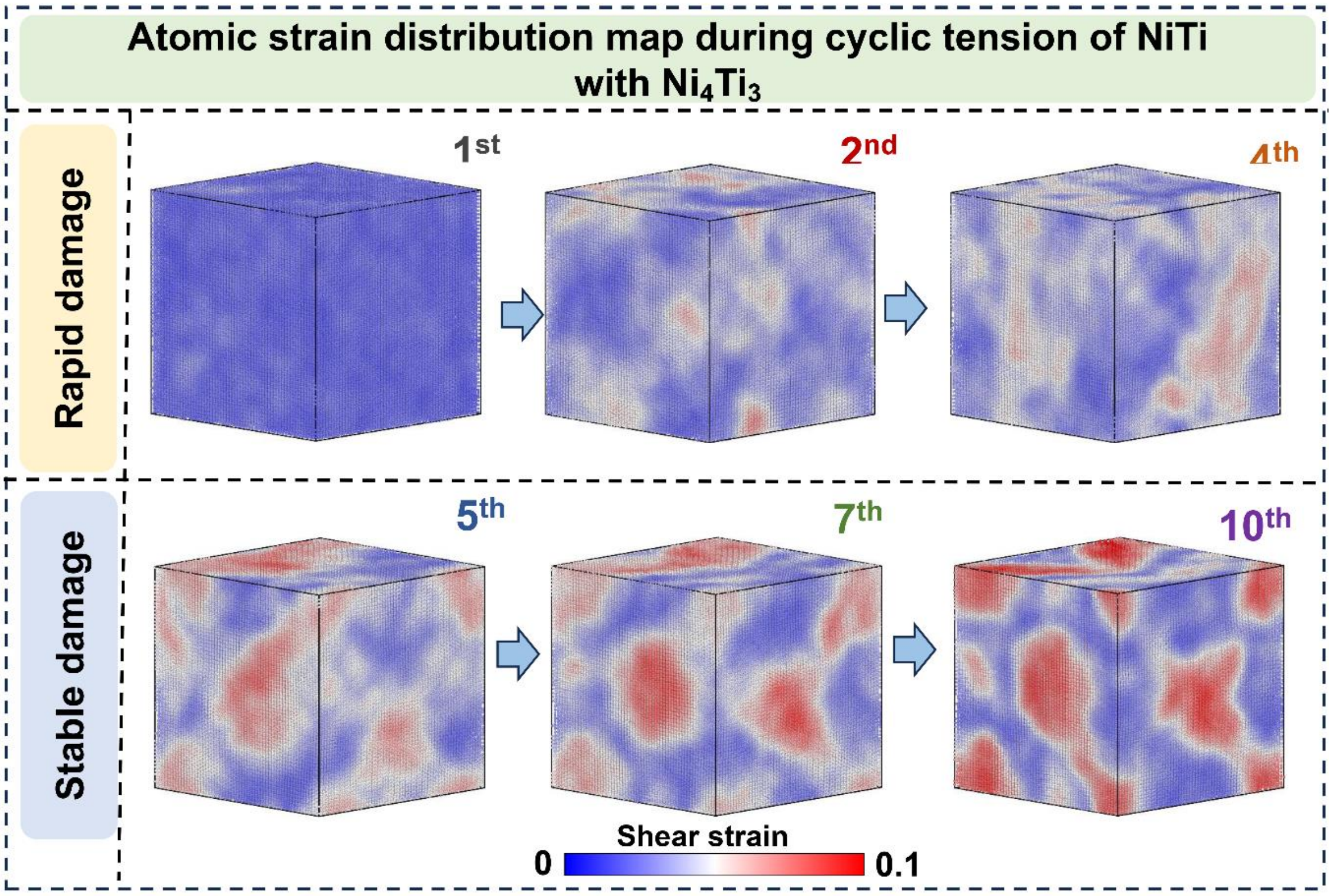

Supplementary Fig. 57 | Atomic strain maps of NiTi with Ni_4Ti_3 precipitates after each cycle in the cyclic simulation.

Supplementary References

1. Zhou, N. et al. Effect of Ni_4Ti_3 precipitation on martensitic transformation in Ti–Ni. *Acta Mater*. **58**, 6685–6694 (2010).
2. Delville, R., Malard, B., Pilch, J., Sittner, P. & Schryvers, D. Transmission electron microscopy investigation of dislocation slip during superelastic cycling of Ni–Ti wires. *Int. J. Plast.* **27**, 282–297 (2011).
3. Li, P., Liu, Y., Shi, X. & Liu, P. Micromechanism of superelastic instability of nanocrystalline NiTi shape memory alloys. *J. Mater. Res. Technol.* **37**, 5360–5368 (2025).
4. Hytch, M., Snoeck, E. & Kilaas, R. Quantitative measurement of displacement and strain fields from HREM micrographs. *Ultramicroscopy* **74**, 131–146 (1998).
5. Gorgannejad, S., Reisi Gahrooei, M., Paynabar, K. & Neu, R. W. Quantitative prediction of the aged state of Ni-base superalloys using PCA and tensor regression. *Acta Mater.* **165**, 259–269 (2019).
6. Niezgoda, S. R., Yabansu, Y. C. & Kalidindi, S. R. Understanding and visualizing microstructure and microstructure variance as a stochastic process. *Acta Mater.* **59**, 6387–6400 (2011).
7. Wang, X. et al. Improved functional stability of a coarse-grained Ti-50.8 at.% Ni shape memory alloy achieved by precipitation on dislocation networks. *Scr. Mater.* **163**, 57–61 (2019).
8. Xu, B. et al. Effect of Ni_4Ti_3 precipitates on the functional properties of NiTi shape memory alloys: A phase field study. *Int. J. Plast.* **177**, 103993 (2024).
9. Gall, K., Sehitoglu, H., Chumlyakov, Y. & Kireeva, I. Tension-compression asymmetry of the stress-strain response in aged single crystal and polycrystalline NiTi. *Acta Mater.* **47**, 1203–1217 (1999).
10. Jin, Y. & Weng, G. A direct method for the crystallography of martensitic transformation and its application to TiNi and AuCd. *Acta Mater.* **50**, 2967–2987 (2002).
11. Liu, Y. The superelastic anisotropy in a NiTi shape memory alloy thin sheet. *Acta Mater.* **95**, 411–427 (2015).
12. Li, Z. et al. Atomic scale modeling of the coherent strain field surrounding Ni_4Ti_3 precipitate and its effects on thermally-induced martensitic transformation in a NiTi alloy. *Acta Mater.* **211**, 116883 (2021).
13. Tirry, W. & Schryvers, D. Quantitative determination of strain fields around Ni_4Ti_3 precipitates in NiTi. *Acta Mater.* **53**, 1041–1049 (2005).

14. Hornbogen, E., Mertinger, V. & Wurzel, D. Microstructure and tensile properties of two binary NiTi-alloys. *Scr. Mater.* **44**, 171–178 (2001).
15. Chen, Y. et al. Lattice distortion and atomic shuffle coupling of the R phase under grain size confinement in a $Ni_{48}Ti_{50}Fe_2$ alloy. *Acta Mater.* **292**, 121055 (2025).
16. Chien, C. et al. Characteristics of the strain glass transition in as-quenched and 250 °C early-aged $Ti_{48.7}Ni_{51.3}$ shape memory alloy. *Acta Mater.* **120**, 159–167 (2016).
17. Ren, D., Wang, C., Wei, X., Lai, Q. & Xu, W. Building a quantitative composition-microstructure-property relationship of dual-phase steels via multimodal data mining. *Acta Mater.* **252**, 118954 (2023).
18. Wu, Y. et al. A versatile multimodal learning framework bridging multiscale knowledge for material design. *npj Comput. Mater.* **11**, 276 (2025).
19. Goodale, M., Milner, A., Jakobson, L. & Carey, D. A neurological dissociation between perceiving objects and grasping them. *Nature* **349**, 154–156 (1991).
20. Xu, M., Yoon, S., Fuentes, A. & Park, D. A comprehensive survey of image augmentation techniques for deep learning. *Pattern Recognit.* **137**, 12 (2023).
21. Schmidt, J., Marques, M. R. G., Botti, S. & Marques, M. A. L. Recent advances and applications of machine learning in solid-state materials science. *npj Comput. Mater.* **5**, 83 (2019).
22. Mehrer, J., Spoerer, C. J., Kriegeskorte, N. & Kietzmann, T. C. Individual differences among deep neural network models. *Nat. Commun.* **11**, 5725 (2020).
23. Na Narong, T., Zachko, Z. N., Torrisi, S. B. & Billinge, S. J. L. Interpretable multimodal machine learning analysis of X-ray absorption near-edge spectra and pair distribution functions. *npj Comput. Mater.* **11**, 98 (2025).
24. Hu, Y. et al. Investigation of full-field strain evolution behavior of Cu/Ni clad foils by interpretable machine learning. *Int. J. Plast.* **184**, 104181 (2025).
25. Jangid, D. K. et al. Adaptable physics-based super-resolution for electron backscatter diffraction maps. *npj Comput. Mater.* **8**, 255 (2022).
26. Mason, J. K. & Schuh, C. A. Hyperspherical harmonics for the representation of crystallographic texture. *Acta Mater.* **56**, 6141–6155 (2008).
27. Song, J. et al. Phase-field-informed machine learning on creep behavior of Ni-based single-crystal superalloys. *J. Mater. Inform.* **5**, 19 (2025).
28. Li, S., Hassanin, H., Attallah, M., Adkins, N. & Essa, K. The development of TiNi-based negative Poisson's ratio structure using selective laser melting. *Acta Mater.* **105**, 75–83 (2016).

29. Otsuka, K. & Ren, X. Physical metallurgy of Ti-Ni-based shape memory alloys. *Prog. Mater. Sci.* **50**, 511–678 (2005).
30. Han, K. et al. A Survey on Vision Transformer. *IEEE Trans. Pattern Anal. Mach. Intell.* **45**, 87–110 (2023).
31. Leitherer, A., Ziletti, A. & Ghiringhelli, L. M. Robust recognition and exploratory analysis of crystal structures via Bayesian deep learning. *Nat. Commun.* **12**, 6234 (2021).
32. Stukowski, A. Visualization and analysis of atomistic simulation data with OVITO-the Open Visualization Tool. *Modell. Simul. Mater. Sci. Eng.* **18**, 7 (2010).
