# Supplementary material for: An interpretable learning framework for exploring superelastic degradation of NiTi shape memory alloys using multimodal data
Source: Nat Commun. 2026 Aug 31;17:10360. doi: 10.1038/s41467-026-77250-4 (PMC13623974; doi:10.1038/s41467-026-77250-4)
Supplement: Supplementary file 2 [file 41467_2026_77250_MOESM2_ESM.pdf]

nature portfolio

Peer Review File

An interpretable learning framework for exploring superelastic degradation of NiTi shape memory alloys using multimodal data

Corresponding Author: Professor Chuanjie Wang

Parts of this Peer Review File have been redacted as indicated to remove third-party material.

This file contains all reviewer reports in order by version, followed by all author rebuttals in order by version.

Version 0:

Reviewer comments:

Reviewer #1

(Remarks to the Author)
This manuscript presents a solid and timely contribution at the intersection of superelasticity, microstructural characterization, and interpretable machine learning. Rather than treating machine learning as a purely predictive black box, the authors use multimodal data fusion and interpretability tools to extract physically meaningful insight into superelastic degradation in NiTi alloys. The work goes beyond reporting performance metrics and may offer a coherent mechanistic narrative linking crystallography, deformation modes, and cyclic degradation.

The combination of Grad-CAM, SHAP analysis, and targeted molecular dynamics simulations is used in a complementary and physically grounded manner. In particular, the spatial correspondence between model attention, Schmid factor maps, and observed deformation features supports the claim that the network captures physically relevant deformation behavior rather than spurious correlations.

The proposed multi-branch feature fusion architecture is reasonably justified through ablation studies and comparisons with alternative fusion strategies. While the architecture itself is not the main novelty, the authors show that simple channel stacking can obscure physically meaningful features, and that explicit feature decoupling improves both accuracy and stability. The overall validation strategy is solid, although the very high reported R2 values may prompt some readers to scrutinize data splitting procedures and sample independence more closely.

I nonetheless have concerns regarding the broader impact of the approach and the generality of the results. The study focuses on a single alloy system and a single loading condition, resulting in a relatively narrow dataset. While the authors discuss extensibility and extrapolation behavior, it remains unclear how broadly the proposed framework would transfer to other shape memory alloys (different microstructure states for instance) or different loading paths. Moreover, although the framework relies heavily on latent representations to fuse multimodal information, none of the learned latent spaces are explicitly analyzed or visualized. This makes it difficult to assess how different modalities contribute to the prediction and how physical information is encoded internally. In parallel, while the framework is technically sound, it is not entirely clear how much fundamentally new materials insight is gained beyond what is already established in the NiTi literature. Several identified mechanisms, such as the competition between slip and transformation, are already known qualitatively.

While the reported training behavior suggests stable convergence, the training and validation loss curves are presented on a linear scale, which limits the ability to rigorously evaluate convergence, particularly during later training stages. Displaying the loss evolution on a logarithmic scale would provide a clearer assessment. Given the high predictive performance, a more explicit assessment of potential overfitting, for example through additional cross-validation or learning-curve analysis, would further strengthen confidence in the model.

The choice of Euler angles as a crystallographic representation also deserves further discussion. Euler space is redundant, which can introduce artificial discontinuities and ambiguity when used as a learning space. Previous studies have shown that quaternion-based representations are often more robust for machine learning tasks involving orientation data. The

authors should comment on the potential impact of Euler-angle redundancy on the learned latent representations and whether alternative encodings could improve robustness or interpretability.

Relatedly, although the authors emphasize multimodal fusion, the interaction between modalities within the latent space is not clearly examined. The framework appears to merge modalities effectively, but the structure and organization of the resulting latent representation are not investigated, which limits interpretability.

Given the emphasis on deformation mechanisms and local plasticity, the inclusion of geometrically necessary dislocation (GND) maps could be considered. GND density provides a more direct physical measure of lattice curvature and plastic incompatibility than KAM alone and could further strengthen the physical grounding of the framework.

The current analysis relies entirely on two-dimensional EBSD data, whereas superelastic degradation and transformation pathways are inherently three-dimensional phenomena. A brief discussion of how three-dimensional microstructural effects might influence the conclusions would be valuable.

The use of PCA to reduce precipitate statistics to a single descriptor is reasonable, but the manuscript does not report how much variance is retained or lost through this reduction.

The choice of residual strain as a single scalar output is experimentally convenient and justified, but it necessarily compresses the complexity of superelastic degradation into one metric. Other degradation descriptors, such as hysteresis width, transformation stress evolution, or energy dissipation per cycle, are not considered. A short justification of this choice, or a discussion of possible multi-output extensions, would strengthen the work.

Finally, while the interpretability tools link input features to the predicted output, the manuscript does not clearly explain how changes in the latent space correspond to changes in the physical output. It therefore remains unclear how variations in encoded microstructure or other modalities translate quantitatively into predictive residual strain evolution. Explicitly connecting latent feature trends to output sensitivity would further support the claim that the model provides mechanistic insight rather than only accurate predictions. Because the output variable is directly related to well-established degradation mechanisms, there is also a risk that the ML interpretation primarily reflects known correlations rather than uncovering new causal structure. A discussion acknowledging this limitation would help position the contribution more clearly as quantitative and spatially resolved rather than purely confirmatory.

Some specimens appear to be partially recrystallized, and the rationale behind this microstructural state is not entirely clear.

In addition to microstructural maps, it may be beneficial to incorporate macroscopic mechanical descriptors, such as yield strength, as additional input modalities. These parameters are known to influence cyclic response and degradation behavior and may not be fully captured by microstructural maps alone, potentially improving predictive robustness.

Because the model relies heavily on EBSD-derived maps, a brief discussion of sensitivity to EBSD preprocessing steps (e.g., denoising, step size) would strengthen confidence in the robustness of the conclusions.

(Remarks on code availability)

Reviewer #2

(Remarks to the Author)
This study addresses the critical issue of superelastic degradation (SED) in NiTi alloys by constructing an interpretable multimodal learning framework that integrates innovation, rigor, and practicality. The research methodology combines multimodal data, interpretable machine learning, experiments, and simulations, successfully revealing the competitive mechanism between dislocation slip and stress-induced martensitic transformation (SIMT) as well as the dual role of precipitates. The conclusions are reliable and hold significant engineering value. This work reveals the mapping relationship between the featured microstructures at the microscopic scale and superelastic degradation behavior at the macroscopic scale, and is important for practical applications. Thus, I recommend this paper to be published after the following comments are addressed by the authors.
Q1: In page 16, is there a labeling error for Fig. 5f?
Q2: Five metrics are used in Fig. 2j to evaluate the effectiveness of input combinations. As the authors mentioned, comparing the Euler input with Euler+Phase dual inputs, the Euler is superior in some metrics while the Euler+Phase input performs better in others. Then how is the conclusion that 'Euler+Phase < Euler' drawn?
Q3: In fact, superelastic degradation not only depends on the magnitude of the external load but also significantly relies on the loading rate (Int. J. Plasticity, 2015, 72, 60-90). This is an important issue in engineering applications. Is it possible to further incorporate this factor into the framework established in this paper?
Q4: As is well known, when Ni_4Ti_3 precipitates are present in NiTi shape memory alloys, they typically induce a two-step phase transformation involving B2 to R and then R to B19'. Was this phenomenon also observed in the experiments conducted in this study?
Q5. There are some issues with the formulation of Equation (4). The meaning of Alpha on the right side of the equation should be clarified. Additionally, D_{klmn} is not an Eshelby tensor itself, but rather a quantity constructed from the Eshelby

tensor. Additionally, since the orientations of different Ni_4Ti_3 precipitates are not uniform, this can lead to the induction of various internal stress fields with different orientations. How does this paper account for this aspect? It is also recommended to include the explicit expressions of D_{klmn} and the lattice mismatch strain in the supplementary materials.
Q6. In recent years, several multi-scale models have also been developed, which to some extent reveal the relationship between the characteristic microstructure of NiTi shape memory alloys and the macroscopic superelastic degradation (J. Mech. Phys. Solids, 2015, 82, 97-136; Int. J. Solids Struct., 2023, 280, 112384). I suggest that the authors provide a brief review of theses works.

(Remarks on code availability)

Version 1:

Reviewer comments:

Reviewer #1

(Remarks to the Author)
The authors did an excellent job leveraging the reviewer comments and scientific discussion to significantly improve the manuscript. Overall, the revised version is substantially stronger, both technically and scientifically, compared to the original submission. In particular, I appreciate the considerable effort dedicated to strengthening the interpretability analysis, latent-space characterization, generalization assessment, and clarification of the data partitioning strategy. The additions made in response to the review comments clearly improve the rigor, transparency, and overall impact of the work.

The revised manuscript now provides a much more convincing demonstration that the framework captures physically meaningful structure–deformation relationships rather than relying on spurious correlations or potential information leakage. The clarification regarding the partitioning of the dataset at the original EBSD scan-region level is especially important given the very high predictive performance reported. The additional cross-validation, learning-curve analysis, and convergence assessments also help strengthen confidence in the robustness and stability of the model. I also believe the newly added latent-space visualizations, grouped SHAP analyses, and multimodal representation analysis substantially improve the interpretability aspect of the study and were important additions to the manuscript as suggested by reviewer 1.

The quantitative analysis of the transition between slip-dominated and transformation-dominated regimes, together with the discussion of the loading-dependent role of Ni4Ti3 precipitates, adds significantly more mechanistic depth than in the original version. The efforts to connect the learned representations to geometric compatibility, local deformation evolution, and variant-transfer behavior provide a much clearer mechanistic narrative and improve the overall scientific value of the work.

That said, I would still encourage the authors to remain somewhat cautious in the framing of the conceptual novelty and the broader generality of the approach. The work is clearly strong, technically sophisticated, and impactful, but several of the identified mechanisms remain broadly consistent with existing qualitative understanding in the NiTi literature. In my opinion, the manuscript is strongest when positioned as an interpretable multimodal framework capable of quantitatively resolving and visualizing degradation processes that were previously difficult to characterize spatially and systematically, rather than as the discovery of fundamentally new deformation physics. Similarly, while the discussion regarding extensibility has been significantly improved, the experimental validation remains largely confined to the NiTi system and related loading conditions, and I would recommend maintaining a relatively measured tone regarding broader transferability.

Overall, I believe the authors addressed the major concerns raised in the original review in a serious, thoughtful, and technically rigorous manner, and the manuscript has been significantly strengthened as a result.

(Remarks on code availability)

Reviewer #2

(Remarks to the Author)
The authors have addressed all my comments in the revised manuscript. Thus, I recommend this paper to be published in its current form.

(Remarks on code availability)

Open Access This Peer Review File is licensed under a Creative Commons Attribution 4.0 International License, which permits use, sharing, adaptation, distribution and reproduction in any medium or format, as long as you give appropriate credit to the original author(s) and the source, provide a link to the Creative Commons license, and indicate if changes were made.
In cases where reviewers are anonymous, credit should be given to 'Anonymous Referee' and the source.
The images or other third party material in this Peer Review File are included in the article's Creative Commons license, unless indicated otherwise in a credit line to the material. If material is not included in the article's Creative Commons license and your intended use is not permitted by statutory regulation or exceeds the permitted use, you will need to obtain permission directly from the copyright holder.
To view a copy of this license, visit https://creativecommons.org/licenses/by/4.0/

Detailed Response to Reviewers' Comments

We thank the reviewers for their constructive comments. We have carefully considered all comments and made substantial revisions to the manuscript. In particular, the interpretability analysis has been significantly strengthened to better elucidate the organization of the latent space and its physical implications. The generalization of the framework has also been further validated under diverse conditions. In addition, the technical details have been refined to improve overall quality and readability. We again thank you for your time in reviewing our manuscript and making it a much more robust and impactful study. The modifications based on the comments, along with detailed answers to those comments, are as follows.

Note that:

(1) The reply to Reviewer 1's comments is listed on Pages 1-72;
(2) The reply to Reviewer 2's comments is listed on Pages 73-100;

The colours of the text correspond to responses to Reviewer 1 - **Colour 1**, Reviewer 2 – **Colour 2**, with all revisions highlighted accordingly.

Reviewer 1: This manuscript presents a solid and timely contribution at the intersection of superelasticity, microstructural characterization, and interpretable machine learning. Rather than treating machine learning as a purely predictive black box, the authors use multimodal data fusion and interpretability tools to extract physically meaningful insight into superelastic degradation in NiTi alloys. The work goes beyond reporting performance metrics and may offer a coherent mechanistic narrative linking crystallography, deformation modes, and cyclic degradation.

The combination of Grad-CAM, SHAP analysis, and targeted molecular dynamics simulations is used in a complementary and physically grounded manner. In particular, the spatial correspondence between model attention, Schmid factor maps, and observed deformation features supports the claim that the network captures physically relevant deformation behavior rather than spurious correlations.

1.0 Response: We greatly appreciate and admire your work in reviewing the manuscript. Your constructive comments were very helpful and provided new insights. We strongly agree with most of the comments and have made extensive revisions. And we have shared our views on a few points. We have substantially strengthened the analysis of the latent space to better elucidate how multimodal information is internally organized and linked to the predicted degradation behavior. In addition, the generalization of the framework has been further validated through extended studies across diverse conditions. Relevant and critical information has also been provided and explained. Thus, we have carefully considered and addressed the concerns you raised, and we hope that our responses and the revisions to the manuscript will be found appropriate and will meet your expectations.

1.1 The proposed multi-branch feature fusion architecture is reasonably justified through ablation studies and comparisons with alternative fusion strategies. While the architecture itself is not the main novelty, the authors show that simple channel stacking can obscure physically meaningful features, and that explicit feature decoupling improves both accuracy and stability. The overall validation strategy is solid, although the very high reported R2 values may prompt some readers to scrutinize data splitting procedures and sample independence more closely.

1.1 Response: We sincerely appreciate this helpful comment. To avoid potential information leakage arising from spatial overlap and to clarify the data construction and partitioning procedures, we have carefully reviewed the relevant processes and provided additional clarifications in both the revised manuscript and the Supplementary information.

Specifically, in this study, EBSD images obtained under different annealing conditions were first segmented into six cropped sub-images (from 1024 × 720 pixels to 336 × 336 pixels). Each sub-image was then paired with the corresponding Ni_4Ti_3 descriptor, cycle number, and strain amplitude to construct labeled samples. Data partitioning was performed at the level of the original EBSD scan regions (field of

view), such that all sub-images derived from the same original image and their associated samples were assigned to the same subset. This strategy effectively prevents information leakage due to spatial overlap between different subsets. The final dataset was divided into training, validation, and test sets in an 8:1:1 ratio. Data augmentation was applied exclusively to the training set, while the validation and test sets were retained in their original forms.

On this basis, we have supplemented the revised manuscript with a detailed description of the data partitioning and usage strategy to clarify the role of each data subset and to ensure information leakage-free processing **(Page 29, Lines 4–14)**: The final dataset comprises approximately 31,536 labeled sample sequences. To prevent information leakage arising from spatial overlap, data partitioning was performed at the level of the original EBSD scan regions, ensuring that all samples derived from a single region were assigned to the same subset. Specifically, the training set was used for parameter optimization, while the validation set was used for performance monitoring and hyperparameter tuning. Feedback from the validation loss determined the early stopping criteria and model selection. The test set was strictly reserved for the final, unbiased performance evaluation and remained independent of all training and adjustment processes. Following partitioning, offline data augmentation was applied exclusively to the training set using cropped sub-images to enhance generalization. The validation and test sets contained only original, unaugmented data.

Meanwhile, we have provided more detailed descriptions of the data construction and partitioning workflow in the **Supplementary information (Machine learning framework construction section)**, including sub-image generation, label pairing, and data augmentation strategies: Dataset description: During dataset construction, EBSD maps obtained under various annealing conditions were first segmented into cropped sub-images, which were then paired with the corresponding Ni_4Ti_3 descriptor, cycle number, and strain amplitude to form the base samples. The dataset was divided into training, validation, and test sets in an 8:1:1 ratio. In practice, all sub-images cropped

from the same raw EBSD map, together with their associated samples, were kept within the same subset during partitioning. Offline data augmentation (e.g., rotation and mirroring) was subsequently applied to the sub-images in the training set, resulting in a total of 31,536 labeled sequences. Details of the augmentation strategies are provided in Supplementary Fig. 30.

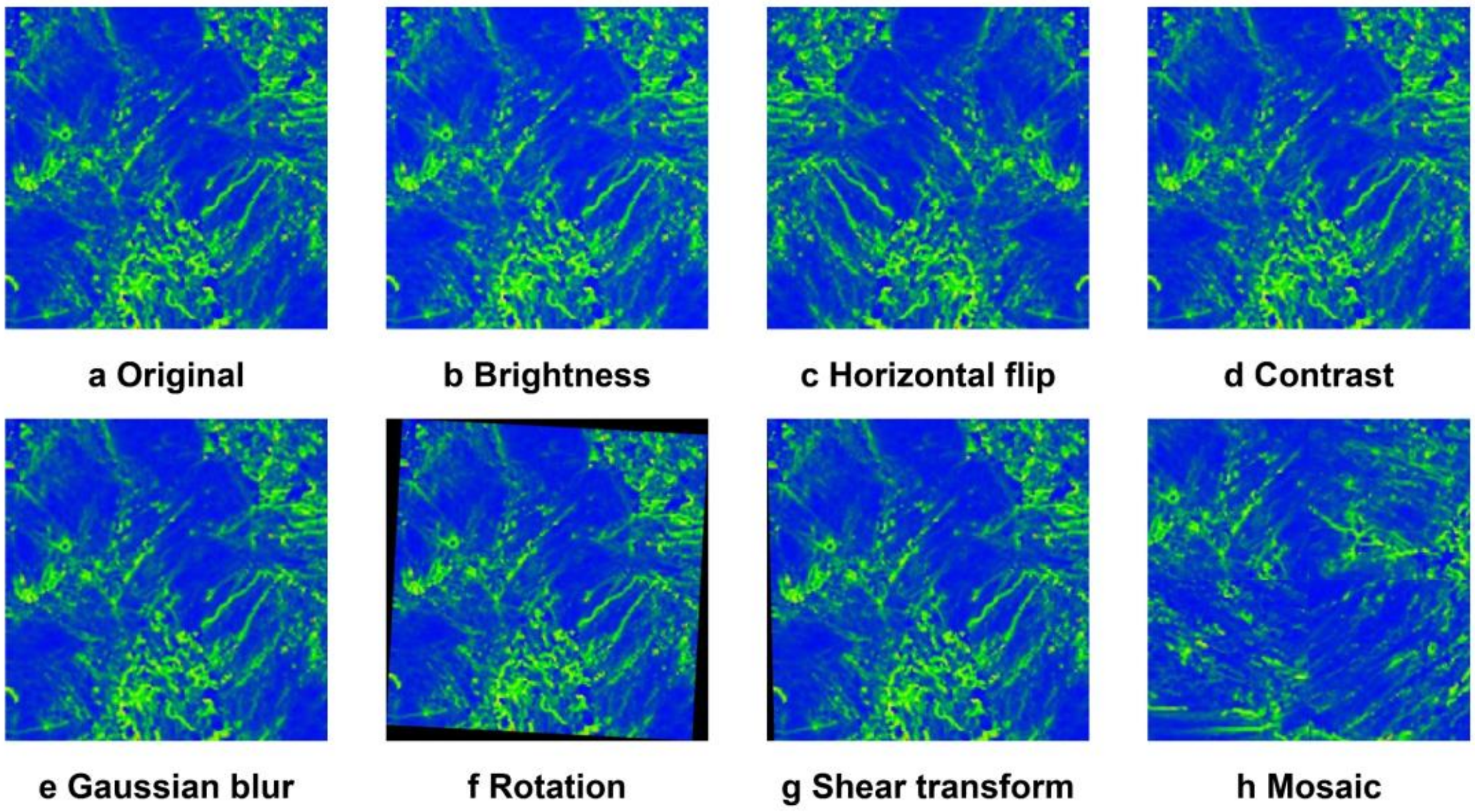

Supplementary Fig. 30 | Schematic illustration of the data augmentation effect on the GND map.

Furthermore, to make the overall workflow more intuitive and clear, we have added a schematic diagram of the data processing workflow in the **Supplementary information (Supplementary Fig. 27)**, which systematically illustrates the entire process from the original EBSD images to the final dataset construction and partitioning.

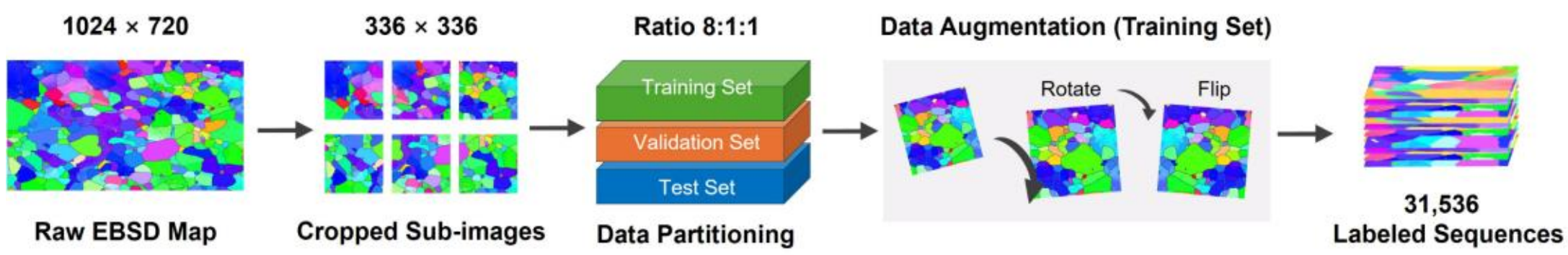

Supplementary Fig. 27 | Schematic illustration of the dataset construction and partitioning workflow. Raw EBSD maps are segmented into cropped sub-images, followed by data partitioning (training/validation/test = 8:1:1) to avoid spatial leakage. Data augmentation is applied only to the training set, resulting in 31,536 labeled sequences.

I nonetheless have concerns regarding the broader impact of the approach and the generality of the results. The study focuses on a single alloy system and a single loading condition, resulting in a relatively narrow dataset. While the authors discuss extensibility and extrapolation behavior, it remains unclear how broadly the proposed framework would transfer to other shape memory alloys (different microstructure states for instance) or different loading paths. Moreover, although the framework relies heavily on latent representations to fuse multimodal information, none of the learned latent spaces are explicitly analyzed or visualized. This makes it difficult to assess how different modalities contribute to the prediction and how physical information is encoded internally. In parallel, while the framework is technically sound, it is not entirely clear how much fundamentally new materials insight is gained beyond what is already established in the NiTi literature. Several identified mechanisms, such as the competition between slip and transformation, are already known qualitatively.

1.2 I nonetheless have concerns regarding the broader impact of the approach and the generality of the results. The study focuses on a single alloy system and a single loading condition, resulting in a relatively narrow dataset. While the authors discuss extensibility and extrapolation behavior, it remains unclear how broadly the proposed framework would transfer to other shape memory alloys (different microstructure states for instance) or different loading paths.

1.2 **Response:** We sincerely appreciate this important comment and have carefully considered it. High predictive accuracy does not imply that the model can be directly extrapolated across different material systems without limitation; its scope of applicability and inherent boundaries require careful clarification. In response, we discuss this issue from three aspects: (i) the generalization performance within the NiTi system, (ii) the extensibility of the framework across different microstructural states, and (iii) its adaptability under diverse loading conditions.

First, regarding the generalization performance of the results: The interpretability

analyses have been validated in this study. From a physical perspective, the revealed superelastic degradation (SED) mechanism, namely the interaction between stress-induced transformation and lattice defects, is a common characteristic of shape memory alloy (SMA) systems. Specifically, the regions to which the model is most responsive, such as phase boundaries, orientation discontinuities, and areas with significant lattice mismatch, are not unique to NiTi. These regions have also been recognized as critical sites for functional fatigue and residual strain accumulation in other SMA systems, including Cu-based and Fe-based alloys[1,2]. Consequently, the model captures intrinsic structural evolution logic rather than mere dataset-specific correlations, suggesting a certain degree of universality beyond the NiTi alloys.

Admittedly, owing to differences in crystal structure and stacking fault energy, the relative contributions of specific features may shift across alloy systems (for example, Cu-based alloys are more sensitive to grain boundary constraints[3]). Consequently, rather than directly extrapolating its quantitative results to other materials, this study emphasizes the methodological value of the proposed multimodal interpretability framework. **Its primary objective is not to establish empirical correlations confined to a single alloy, but to provide an adaptive modeling paradigm tailored to diverse microstructural characteristics.** With appropriate data input, the framework can be extended to characterize the relationships between microstructure, loading conditions, and mechanical response in other material systems, thereby offering a transferable analytical approach for a broader range of SMAs.

Second, regarding extensibility across different microstructural states: The scope of analysis in this study is based on the pre-screening of 32 distinct microstructural states. In the preliminary exploration, specimens that no longer exhibited superelasticity were selected for validation. For instance, for specimens annealed at 800 °C for 1 h, the model reasonably identified the absence of superelastic response **(Supplementary Fig. 53)**. This result can be interpreted in terms of dislocation density. Compared with other typical conditions, high-temperature annealing at 800 °C promotes sufficient recovery and recrystallization in the matrix, thereby significantly reducing the

geometrically necessary dislocation (GND) density and consequently lowering the matrix yield strength. As a result, plastic slip is more likely to occur during loading rather than reversible phase transformation. This example demonstrates that the model is not restricted to specific microstructural types, but **possesses certain adaptability to different microstructural conditions within the same alloy system**, capturing the fundamental correspondence between microstructural state and functional performance.

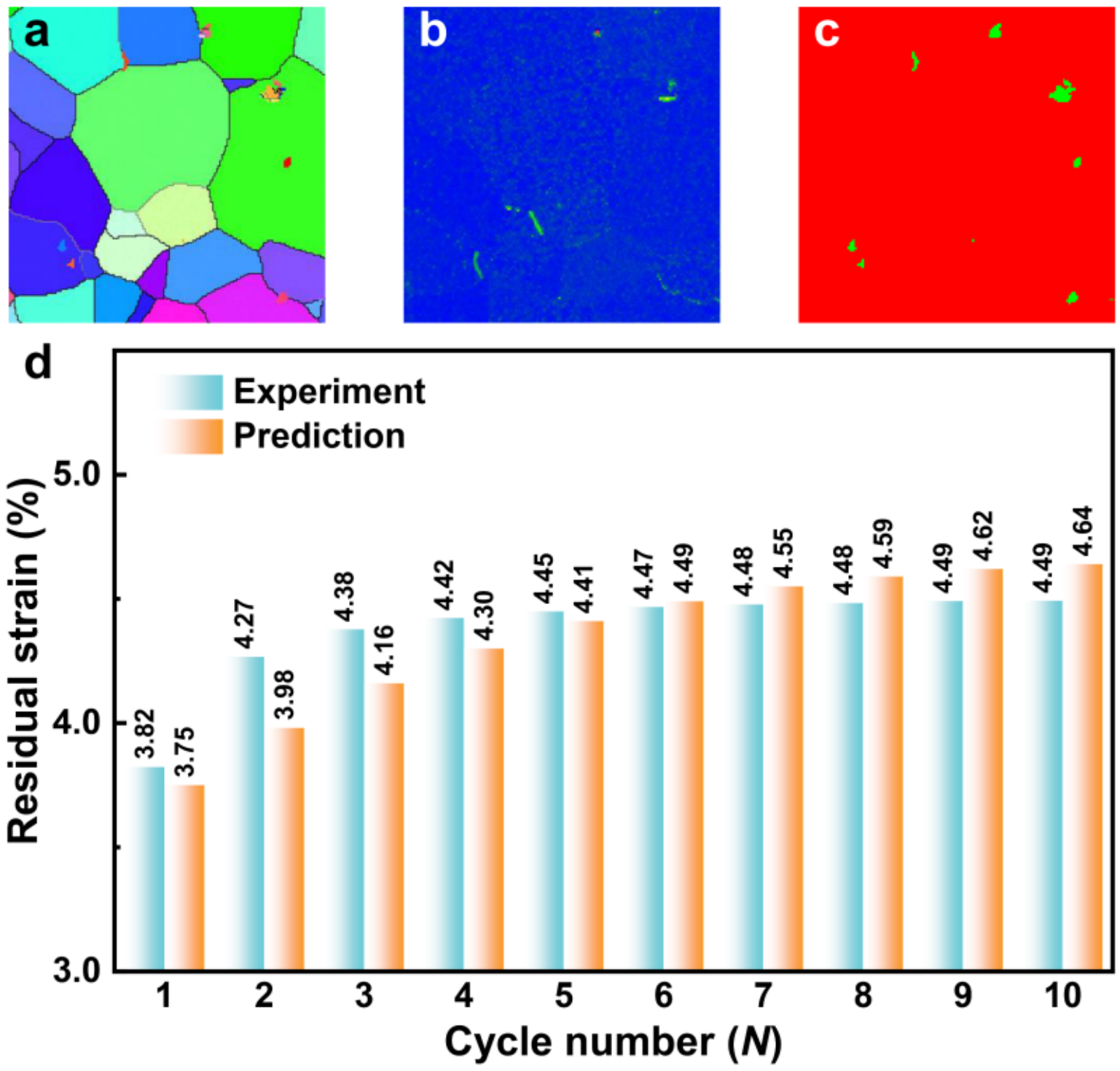

Supplementary Fig. 53 | Microstructural characterization and degradation prediction for the specimen annealed at 800 °C for 1 h. a Inverse pole figure (IPF) map exhibiting a fully recrystallized microstructure with a dominant <101> texture. **b** GND map illustrating a generally low dislocation density. **c** Phase map showing a B2 austenite matrix containing $NiTi_2$ precipitates. **d** Comparison between experimental and predicted residual strain under 6% strain amplitude over 10 cycles. Notably, this 800 °C / 1 h condition was excluded from the training set, demonstrating the robustness of the framework in capturing superelastic degradation trends of an unseen, fully annealed state.

Regarding extensibility across different alloy systems, **the multimodal modeling approach itself is not restricted to any specific alloy family**. This is supported by numerous predictive studies in metallic materials. For example, in dual-phase steels, researchers have combined compositional information with multi-source EBSD images as model inputs to predict mechanical properties across varying Mn contents and processing routes[4]. In Al–Si alloys, Zhu et al. developed a multimodal fusion framework that integrates composition and microstructure to predict tensile strength and noted that the method is applicable to other alloys as well. Additionally, for heat-treated stainless steels, Wang et al.[5] incorporated microstructural images, composition, and processing parameters into a Transformer-based multimodal framework to predict hardness and wear behavior. Although these studies focus on different materials, they demonstrate that multimodal modeling can be effectively applied across different alloy systems.

On this basis, the distinction of the present work lies primarily in its research focus and the organization of information. Existing representative studies have largely concentrated on predicting static mechanical properties or alloy screening, whereas this work addresses cyclic SED, a problem that is more dependent on local microstructural fields and loading history. In terms of information organization, previous approaches typically integrate heterogeneous inputs at the initial stage without explicitly distinguishing their physical semantics. In contrast, this framework processes microstructural information and scalar parameters separately: the image branch extracts microstructural features, while the numerical branch introduces loading conditions and other variables, with fusion performed at a later stage. This strategy helps preserve the physical meaning of each variable and enhances the transparency of microstructural representation. When extended to other SMAs, the framework offers the advantage of explicitly defined interfaces for incorporating additional variables, eliminating the need to redesign input formats as the material system changes. This distinguishes it from approaches that require reorganization of multimodal information at the input stage, thereby improving its practical extensibility.

Furthermore, this structure also facilitates interpretability analysis. Under given loading conditions (e.g., cycle number or amplitude), targeted visualization of microstructural evolution can be achieved, enabling a clearer understanding of the relationship between loading and response.

Third, adaptability under diverse loading conditions: Previous studies have shown that SED is sensitive to temperature, strain rate, and loading path and sequence. We clarify here that temperature and strain rate can be introduced as scalar external variables, whereas the loading path is intrinsically history-dependent and should be represented through history-aware variables, such as internal state descriptors (e.g., martensite fraction or reorientation state variables) or sequence encodings of the loading history[6,7]. In this sense, the presented framework provides an explicit interface not only for introducing additional scalar variables but also for incorporating history-aware path representations. However, due to the limitations of the currently available experiments and testing cycles, systematic comparative experiments under distinct proportional/non-proportional or multiaxial loading paths were not carried out in this study.

To further examine this aspect, we conducted additional validation experiments by introducing external loading variables. Specifically, three strain-rate conditions (1×10^{-3}, 1×10^{-2}, and 3×10^{-2} s^{-1}) were incorporated, together with temperature-dependent tests as supplementary comparisons **(Supplementary Figs. 2-5 and 7-9 are presented below)**. The model maintains predictive performance across four strain rates, and the residual strain prediction achieved R^2 = 89.42% on the test set. Under room temperature and 40 °C, the test R^2 was 78.29% **(Supplementary Figs. 48, 49)**. These results support the extensibility to richer loading-condition inputs, while validation under different loading paths will be the subject of future work.

In summary, rather than claiming unconditional generalizability across all SMAs, this study validates that our model achieves stable performance within the NiTi system across diverse microstructural states. By maintaining independent physical representations and integrating them in a structured manner, the framework provides

clear pathways for expansion to new microstructural conditions or loading variables. The primary contribution of this work is the proposal of a multimodal modeling paradigm tailored to functional degradation. It emphasizes the preservation of physical interpretability, offering a methodological foundation for future research on broader material systems and under increasingly complex service environments.

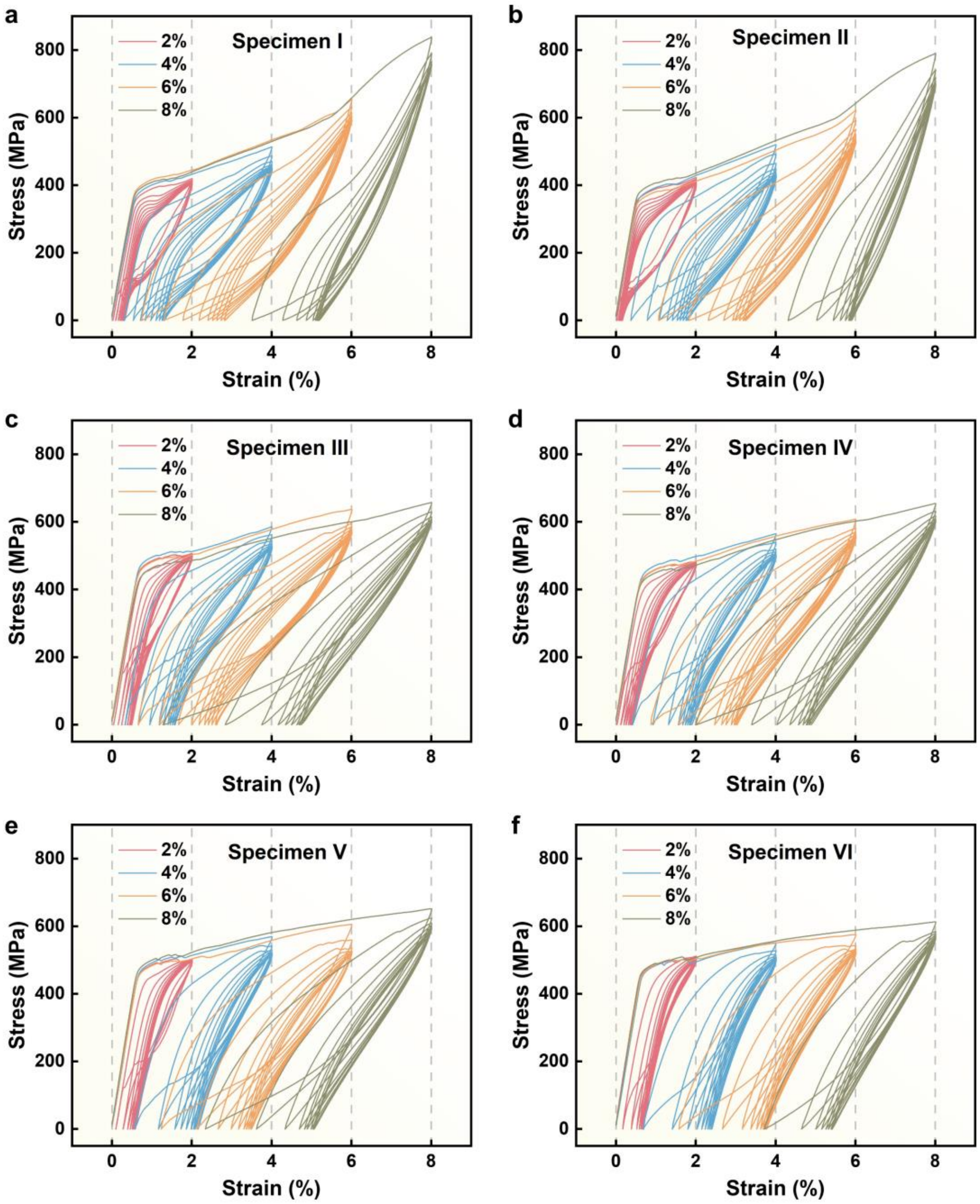

Supplementary Fig. 2 | Cyclic tensile loading–unloading engineering stress–strain curves of NiTi alloys at a strain rate of 10^{-3} s^{-1}. (**a**–**f**) correspond to specimens I–VI, respectively, showing the cyclic test results under strain amplitudes of 2%, 4%, 6%, and 8%.

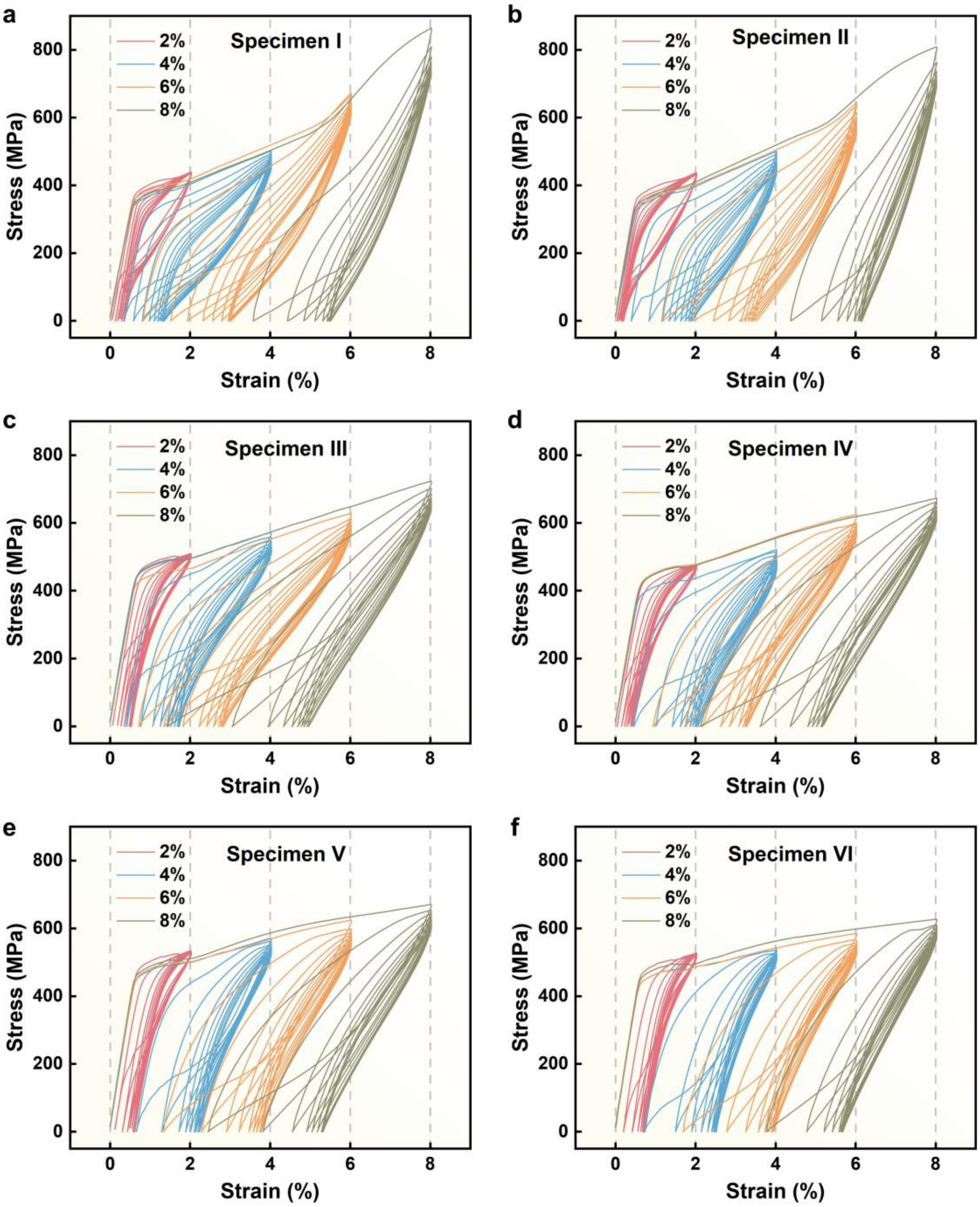

Supplementary Fig. 3 | Cyclic tensile loading–unloading engineering stress–strain curves of NiTi alloys at a strain rate of 10^{-2} s^{-1}. (**a**–**f**) correspond to specimens I–VI, respectively, showing the cyclic test results under strain amplitudes of 2%, 4%, 6%, and 8%.

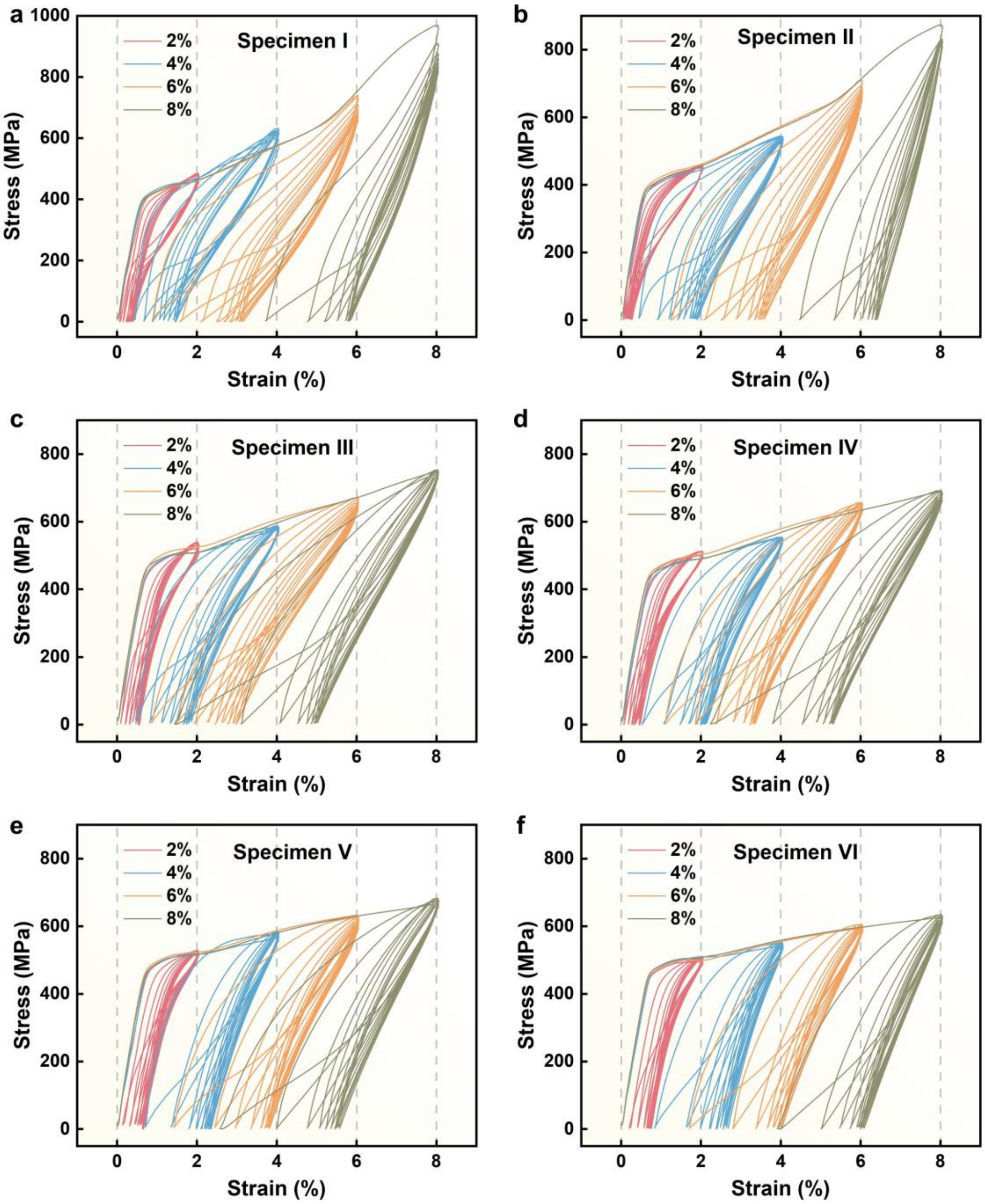

Supplementary Fig. 4 | Cyclic tensile loading–unloading engineering stress–strain curves of NiTi alloys at a strain rate of 3 × 10^{-2} s^{-1}. (**a**–**f**) correspond to specimens I–VI, respectively, showing the cyclic test results under strain amplitudes of 2%, 4%, 6%, and 8%. Due to equipment overshoot at this high strain rate, the raw data were denoised to ensure accurate identification of cyclic characteristics.

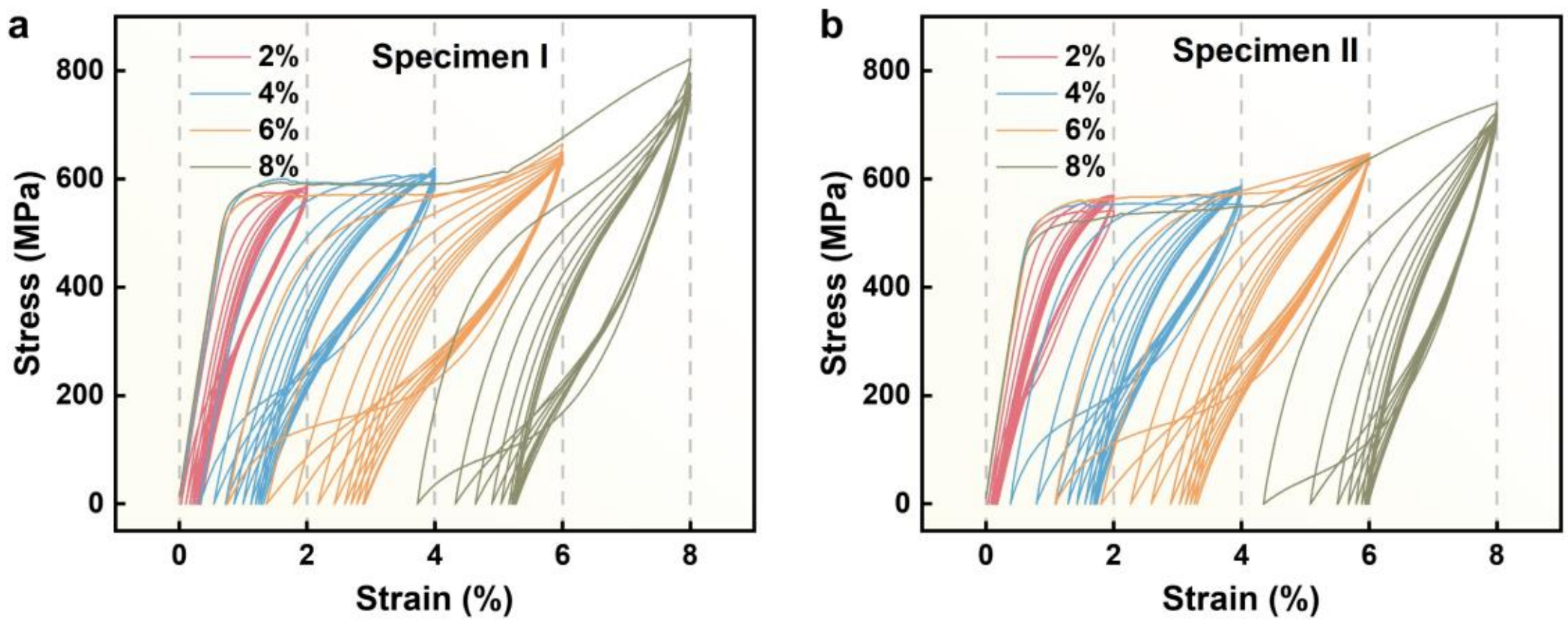

Supplementary Fig. 5 | Cyclic tensile loading–unloading engineering stress–strain curves of NiTi alloys at 40 °C under a strain rate of 5×10^{-4} s^{-1}. (**a**, **b**) correspond to specimens I and II, respectively, showing the cyclic test results under strain amplitudes of 2%, 4%, 6%, and 8%.

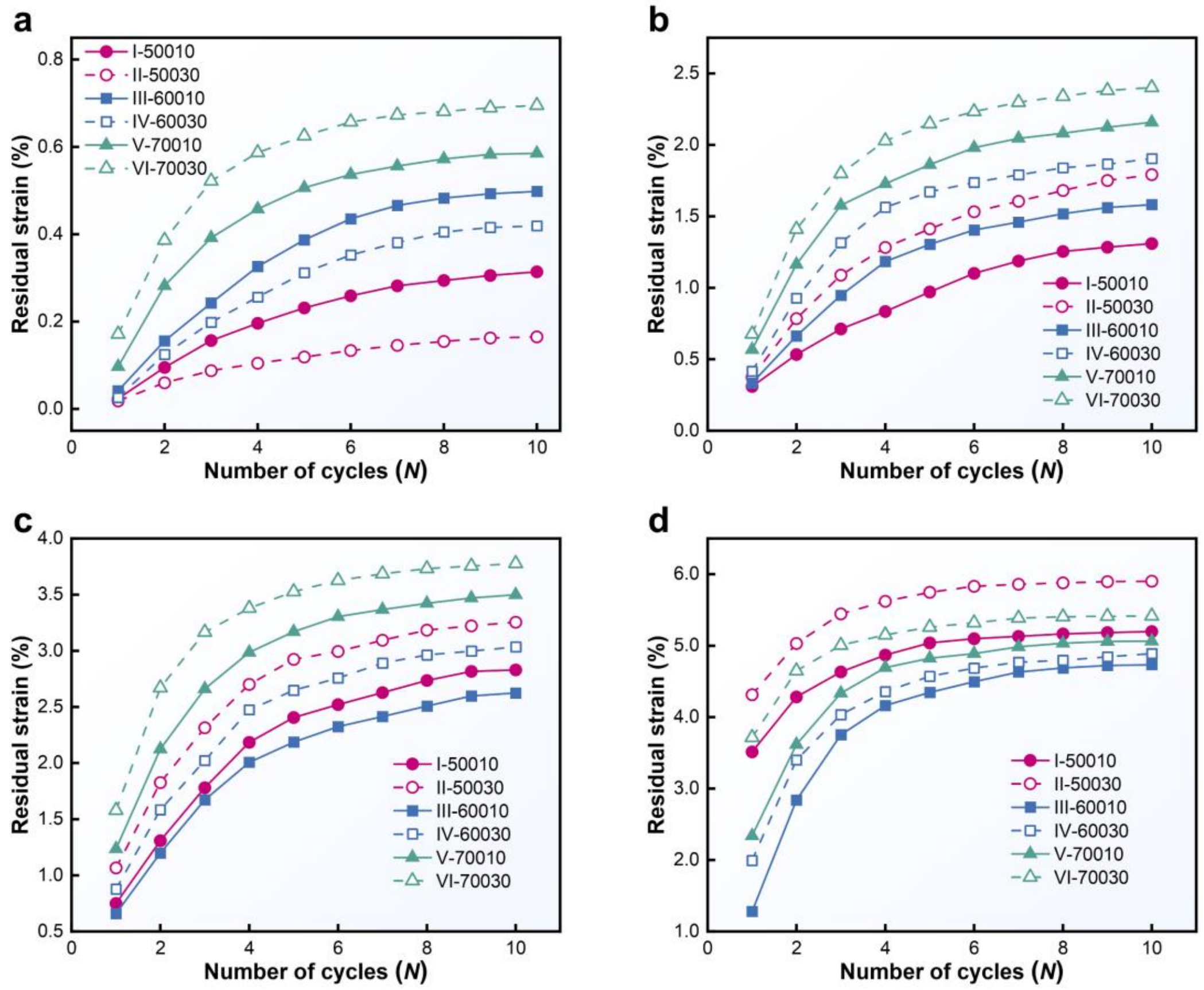

Supplementary Fig. 7 | Residual strain accumulation of NiTi alloys under various annealing conditions at a strain rate of 10^{-3} s^{-1}. Evolution of residual strain with increasing cycle number at strain amplitudes of (**a**) 2%, (**b**) 4%, (**c**) 6%, (**d**) 8%.

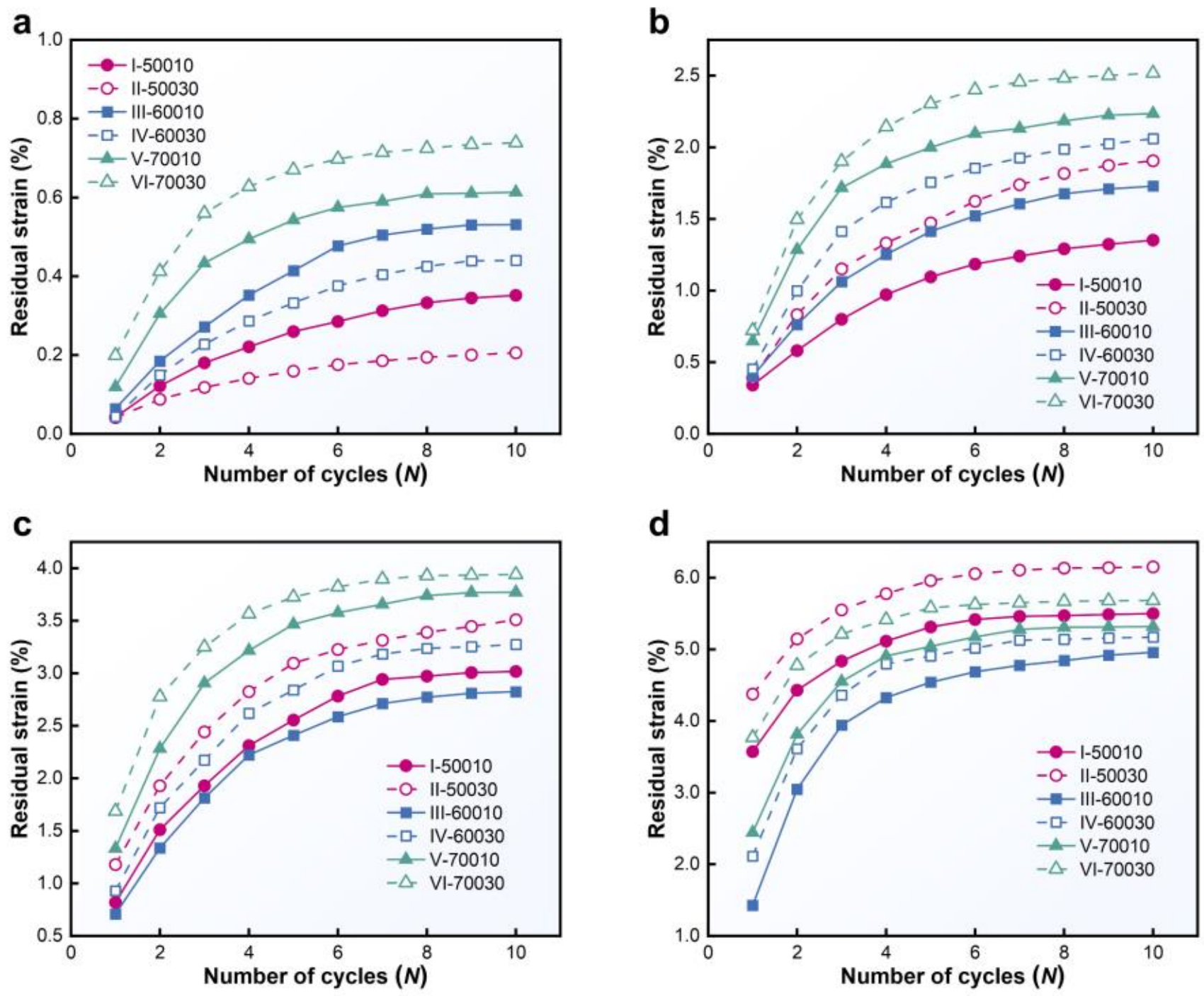

Supplementary Fig. 8 | Residual strain accumulation of NiTi alloys under various annealing conditions at a strain rate of 10^{-2} s^{-1}. Evolution of residual strain with increasing cycle number at strain amplitudes of (**a**) 2%, (**b**) 4%, (**c**) 6%, (**d**) 8%.

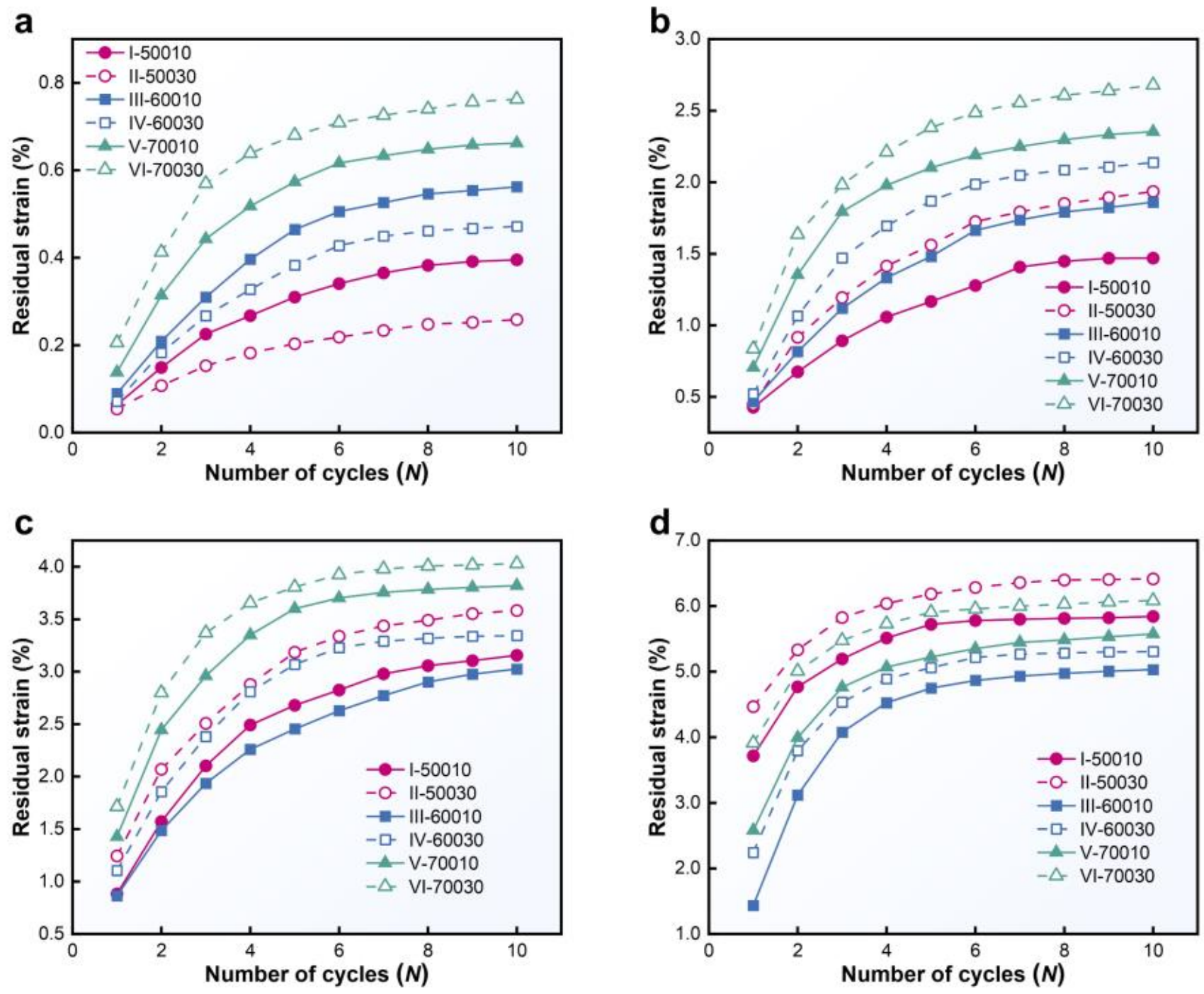

Supplementary Fig. 9 | Residual strain accumulation of NiTi alloys under various annealing conditions at a strain rate of 3×10^{-2} s^{-1}. Evolution of residual strain with increasing cycle number at strain amplitudes of (**a**) 2%, (**b**) 4%, (**c**) 6%, (**d**) 8%.

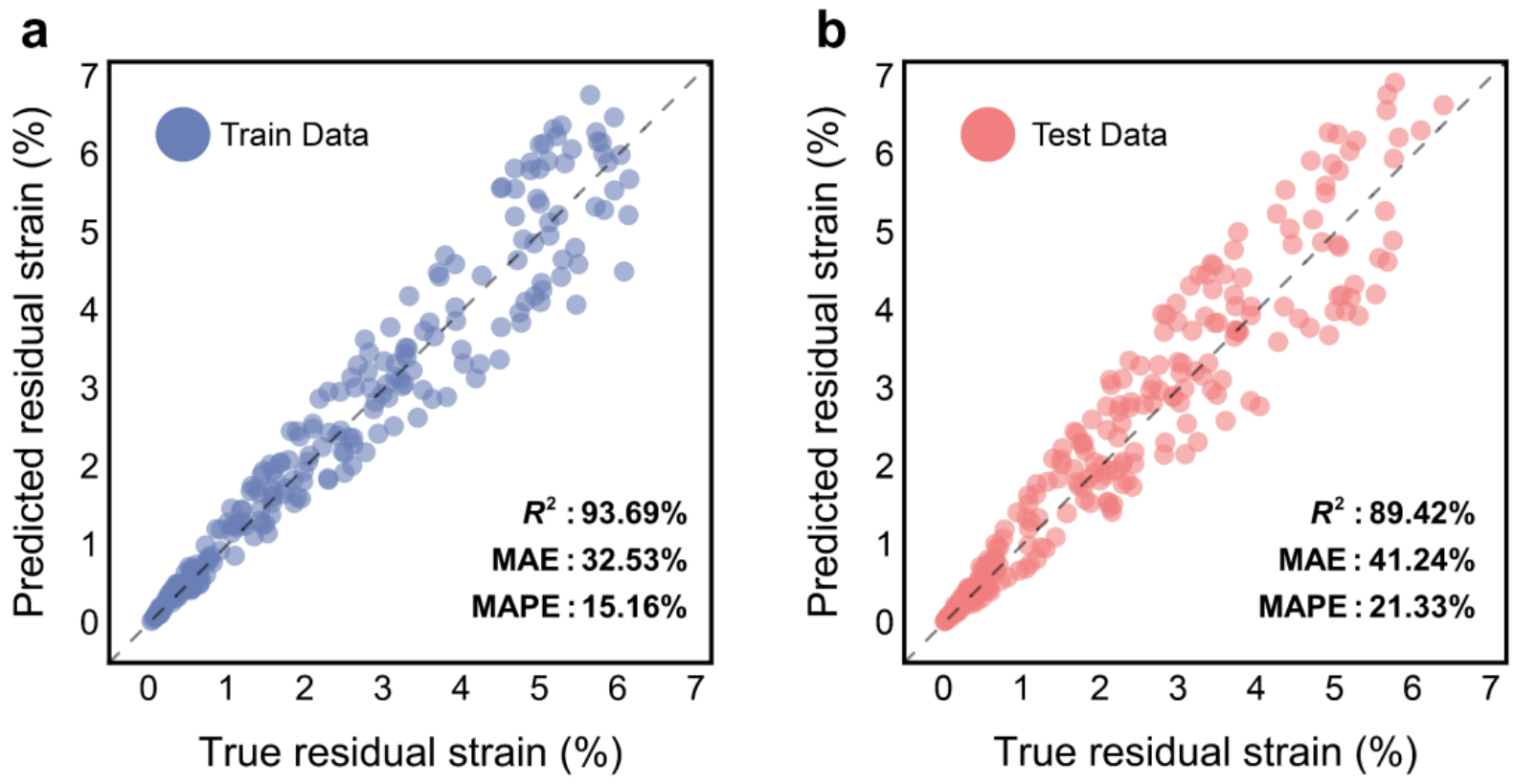

Supplementary Fig. 48 | Predictive performance of residual strain across varying strain rates. a, **b** Comparison between predicted and experimental residual strain for the (**a**) training and (**b**) test datasets at room temperature. Predicted residual strain is plotted against experimental values for cyclic tensile loading–unloading tests performed at four strain rates (5 × 10^{-4} s^{-1}, 1 × 10^{-3} s^{-1}, 1 × 10^{-2} s^{-1}, and 3 × 10^{-2} s^{-1}). The dashed line denotes ideal agreement ($y = x$). Model performance is quantified by R^2, MAE, and MAPE, yielding 93.69%, 32.53%, and 15.16% for the training set, and 89.42%, 41.24%, and 21.33% for the test set, respectively.

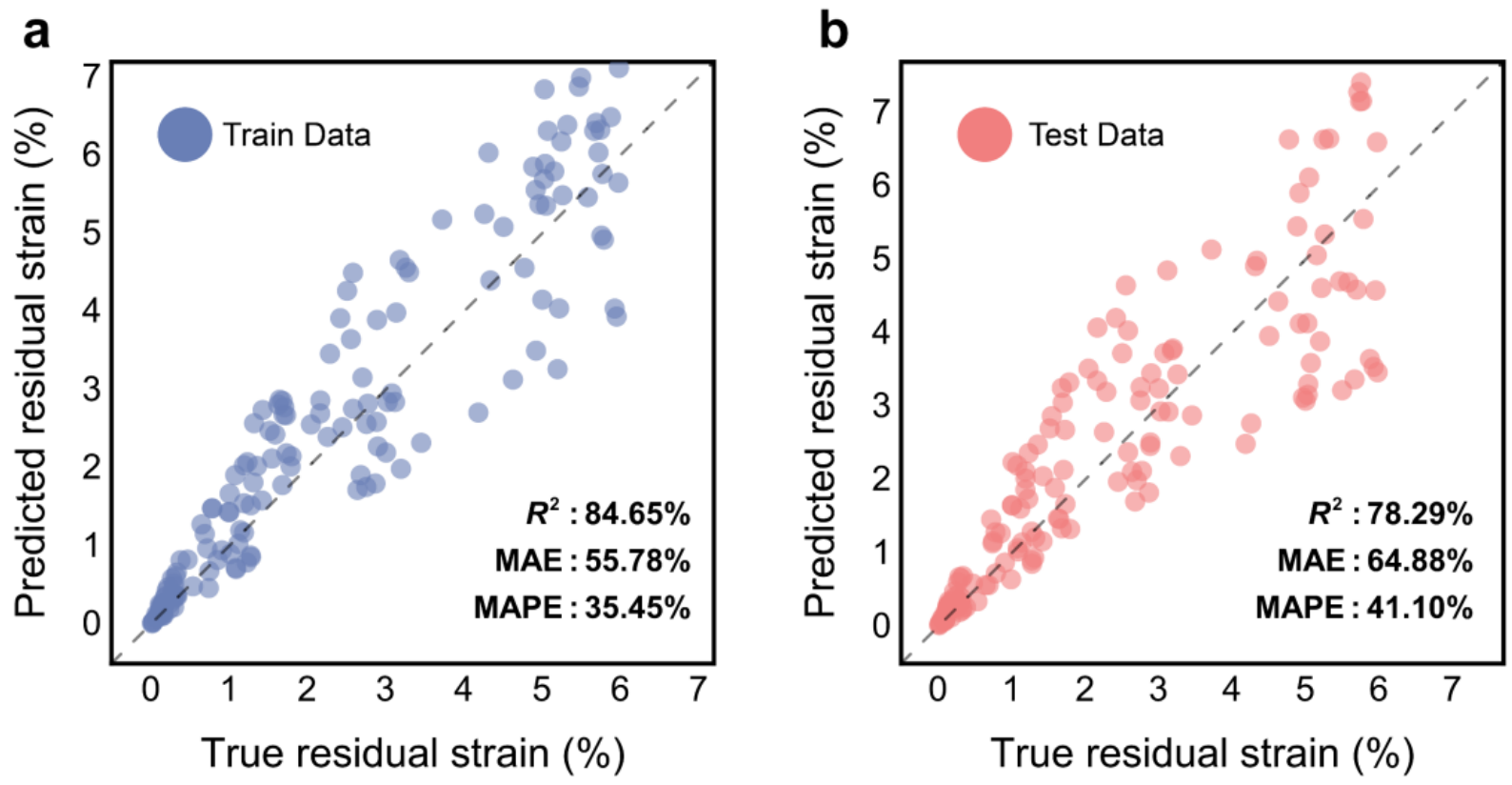

Supplementary Fig. 49 | Predictive performance of residual strain under different temperature conditions. a, **b** Comparison between predicted and experimental residual strain for the (**a**) training and (**b**) test datasets. The results are obtained from cyclic tensile loading–unloading tests conducted at room temperature and 40 °C under a quasi-static strain rate (5 × 10^{-4} s^{-1}). The dashed line denotes ideal agreement ($y = x$). Model performance is quantified by R^2, MAE, and MAPE, yielding 84.65%, 55.78%, and 35.45% for the training set, and 78.29%, 64.88%, and 41.10% for the test set, respectively.

1.3 Moreover, although the framework relies heavily on latent representations to fuse multimodal information, none of the learned latent spaces are explicitly analyzed or visualized. This makes it difficult to assess how different modalities contribute to the prediction and how physical information is encoded internally.

1.3 **Response:** We sincerely appreciate your insightful and helpful comment. The original manuscript primarily demonstrated model interpretability through spatial responses at the input level (Grad-CAM) and importance attribution (SHAP), while lacking direct analysis and visualization of the latent representations after multimodal fusion. This indeed made it difficult to assess how different modalities contribute to the predictions and how physical information is encoded internally. To address this, we have supplemented the revised manuscript with analyses from two perspectives: the contribution of different modalities to the prediction and the encoding of physical information within the latent space.

It should be clarified that the SHAP analysis in this study is not performed on the raw input layer. Instead, SHAP is applied to the concatenated feature vector prior to the feature synergy module, which comprises feature representations extracted from the three convolutional branches together with numerical inputs. Accordingly, SHAP reflects the contribution of each modality after branch-level feature extraction. This has been clarified in the **Implementation of interpretive technologies section (Page 33, Lines 8–11)**: Finally, SHAP analysis was conducted on the composite feature vector at the input of the synergy module. This vector was constructed by concatenating numerical features with image-modality representations, with labels appended to distinguish the origins of features across different branches.

Building on this, we have supplemented two types of averaged SHAP visualizations. The first is the mean absolute SHAP based on latent features, which characterizes the average contribution at the feature dimension level. The second is the grouped-mean SHAP aggregated at the modality level, which represents the relative importance of different modalities in the overall prediction. Both analyses show consistent trends: strain amplitude and cycle number dominate the model

predictions; among microstructural information, orientation and phase distribution contribute more significantly, while the Ni_4Ti_3 descriptor also exerts an independent effect. In contrast, features associated with local lattice distortion exhibit a relatively weaker but still discernible contribution.

Considering that the existing SHAP visualization (Fig. 5a) already conveys the primary conclusions, redundant discussion has been avoided. The two additional sets of mean SHAP results are incorporated to complement the latent-level analysis. These results are presented below and are also included in **Supplementary Fig. 42**.

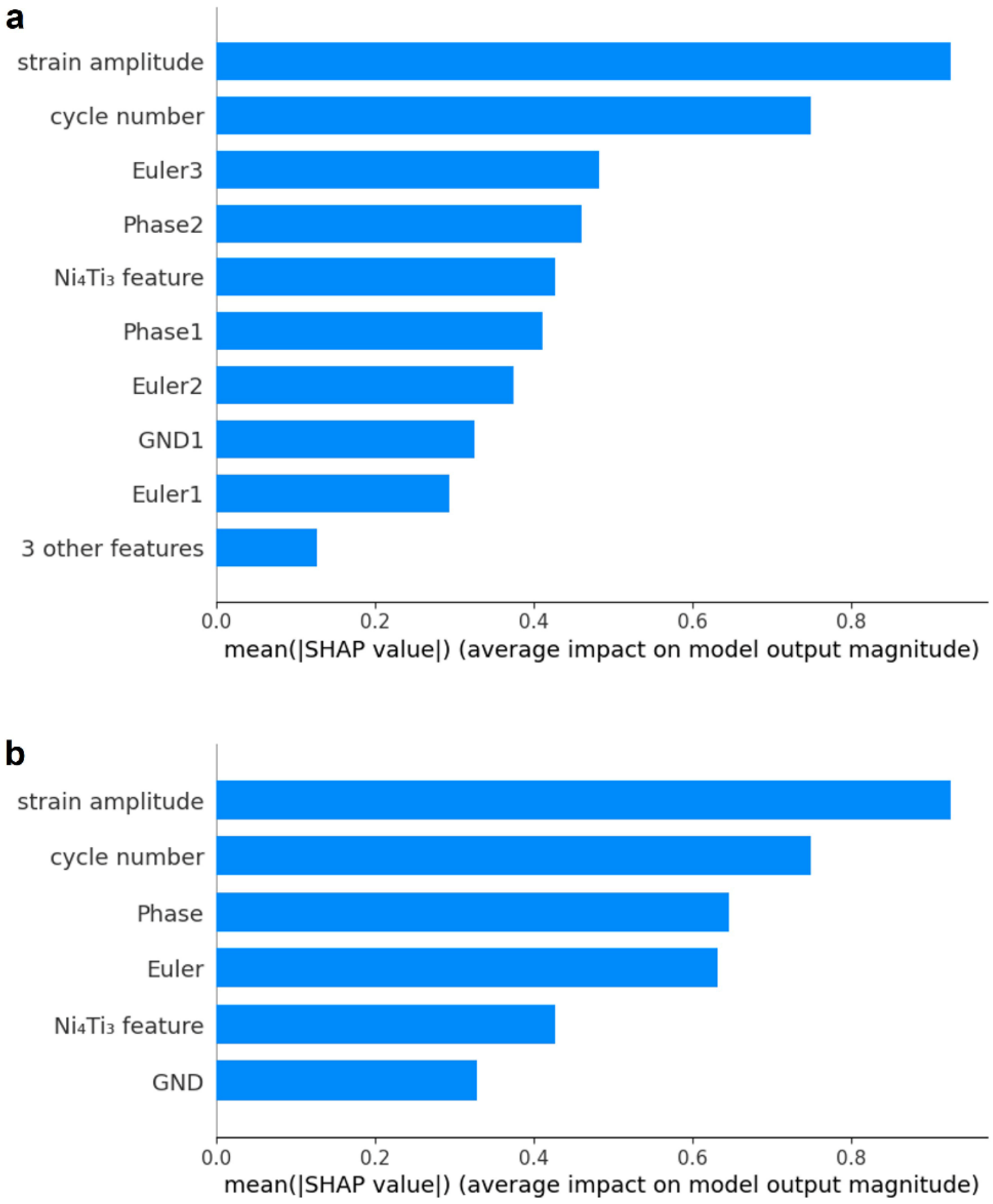

Supplementary Fig. 42 | Global interpretability of residual strain prediction based on SHAP analysis. a Mean absolute SHAP values of individual latent features, ranked by their contributions to the model output. **b** Grouped mean SHAP values aggregated at the modality level. At both levels, strain amplitude and cycle number dominate the prediction, highlighting the primary role of loading conditions in superelastic degradation. Among microstructural descriptors, orientation-related

features and phase information contribute moderately. Specifically, the Ni_4Ti_3 feature captures essential precipitate effects, whereas the GND density exhibits a localized yet discernible impact associated with lattice distortion.

Second, to examine how physical information is encoded internally, we have supplemented UMAP visualizations of the latent features after the synergy module. Specifically, high-dimensional feature vectors obtained after fusion were extracted and projected into a two-dimensional manifold. The resulting embeddings were then colored by the true residual strain, strain amplitude, cycle number, and Ni_4Ti_3 descriptor to characterize the organization of physical information in the latent space. The latent space exhibits relatively continuous and well-organized distributions, indicating that the model has learned stable internal representations associated with degradation behavior (see Fig. R1). Among these, the UMAP result colored by the true residual strain has been incorporated into **Fig. 2j**, where it is compared with the representation prior to the synergy module. Corresponding results will be provided in the following responses.

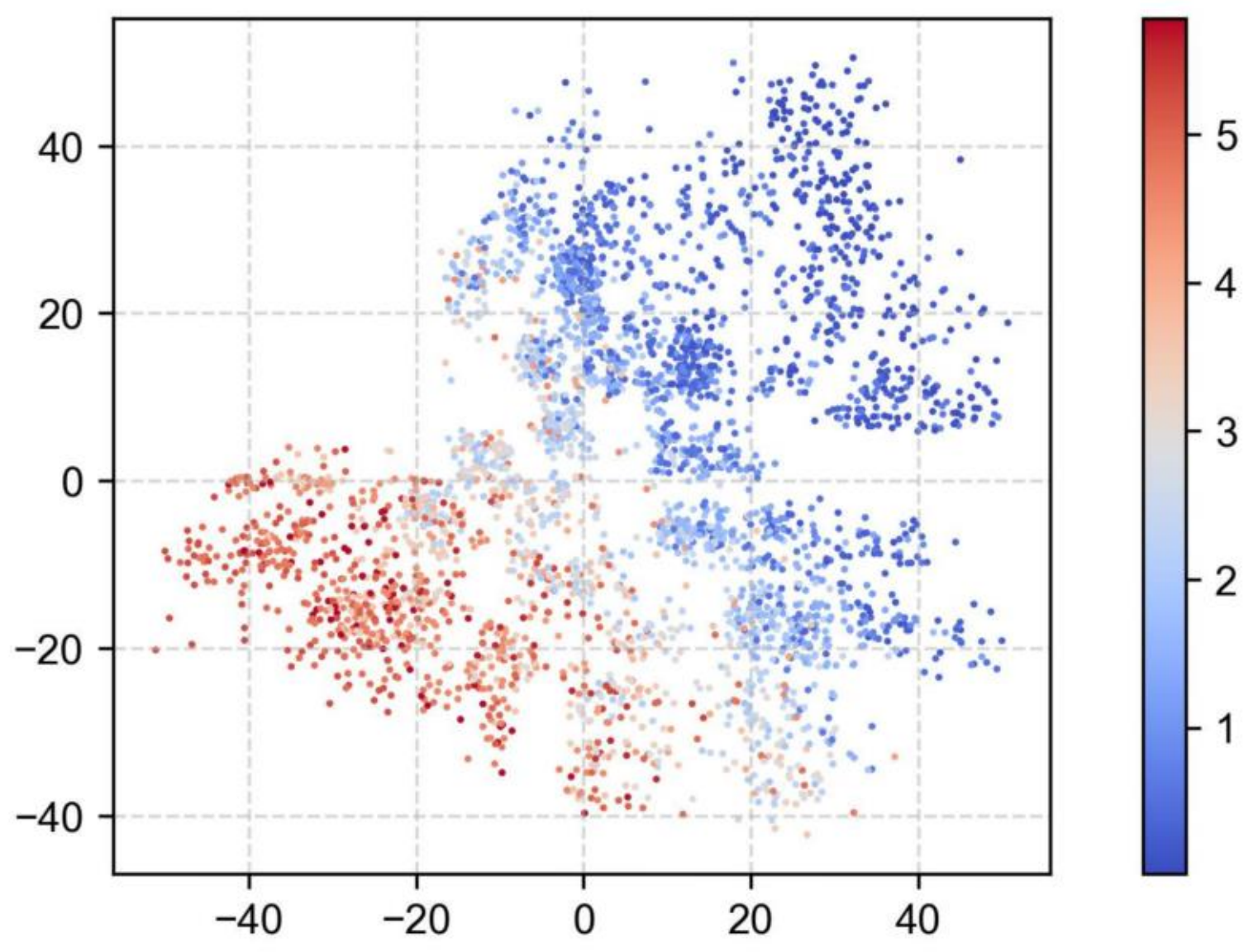

Fig. R1 | Visualization of the latent space. UMAP projection of the feature vectors extracted after the synergy module. Each point represents an individual sample, colored according to its experimental residual strain.

The remaining three coloring schemes, namely strain amplitude, cycle number, and the Ni_4Ti_3 descriptor, are presented collectively in the **Supplementary information (Supplementary Fig. 39)**, together with comparisons before and after the synergy module. These results further demonstrate that strain amplitude forms the most continuous and distinct global gradient in the latent space, with a spatial distribution highly consistent with that of true residual strain, indicating its dominant role in the learned representation. The cycle number also shows a discernible spatial evolution, though with lower separability. In contrast, the Ni_4Ti_3 feature displays a more localized distribution rather than a continuous gradient. This observation is consistent with the subsequent analysis of precipitate effects, where the influence of Ni_4Ti_3 is shown to depend strongly on annealing conditions rather than following a simple monotonic trend. The specific results are shown below:

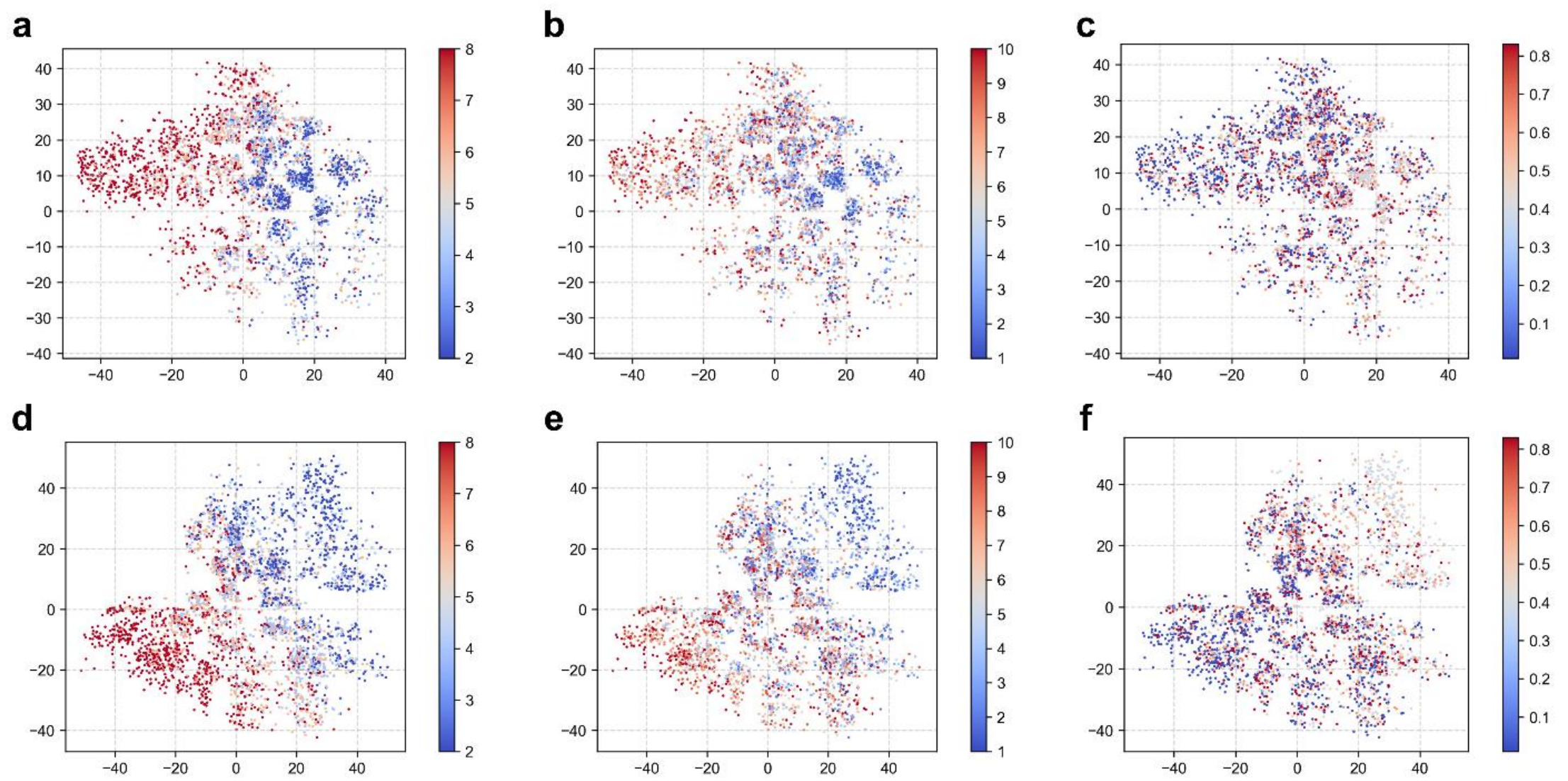

Supplementary Fig. 39 | UMAP visualization of latent spaces before and after feature synergy. Panels **a–c** and **d–f** illustrate the latent representations prior to and following feature fusion, respectively. Clusters are colored by strain amplitude (**a, d**), cycle number (**b, e**), and the Ni_4Ti_3 descriptor (**c, f**). Strain amplitude exhibits the most continuous and well-defined spatial gradient, closely aligning with the residual strain distribution and underscoring its primary role in latent space organization. While cycle number displays a discernible trend, its spatial separation is less pronounced. Notably, samples with intermediate Ni_4Ti_3 characteristic values form

distinct local regions, whereas high and low values show increased clustering or spatial proximity. This distribution is consistent with the experimental observation that both coarsened precipitates and precipitate-free conditions accelerate superelastic degradation. After fusion, the latent space maintains a stable yet more compact topology, indicating that the synergy module effectively enhances the integrated representation of multimodal inputs.

Finally, the **Implementation of interpretive technologies section (Page 32, Lines 21–28 and Page 33, Line 1)** has been expanded to include detailed descriptions of the UMAP implementation, including latent feature extraction, sample selection, and parameter settings. **Other corresponding revisions have also been made throughout the manuscript.** The relevant details are provided below: To achieve a comprehensive interpretable analysis, UMAP, Grad-CAM, and SHAP methods were employed. Following model training, latent feature vectors were extracted from 3,360 original training samples (excluding augmented data) at the corresponding network layer. Dimensionality reduction was performed using the umap-learn package to project the features into a 2D space for visualization. The manifold learning parameters were set to n_neighbors = 15 and min_dist = 0.1, with identical configurations used across all comparative visualizations. Scatter points were colored by true residual strain or other key physical variables, enabling intuitive interpretation of the latent space structure and its relation to deformation behavior.

1.4 In parallel, while the framework is technically sound, it is not entirely clear how much fundamentally new materials insight is gained beyond what is already established in the NiTi literature. Several identified mechanisms, such as the competition between slip and transformation, are already known qualitatively.

1.4 **Response:** Thank you very much for your professional insight. We concur with your perspective that the competition between slip and stress-induced martensitic transformation (SIMT) in NiTi shape memory alloys has been qualitatively discussed.

Previous studies based on techniques such as TEM, synchrotron diffraction, and theoretical modeling have shown that the superelastic degradation (SED) process is typically associated with dislocation accumulation and the formation of transformation-related structures. However, rather than merely reiterating these qualitative observations, our study seeks to transcend conventional limitations by employing a systematic, quantitative evolutionary perspective. By decoupling the synergistic effects of multiple variables, our framework resolves the spatial correlations between microstructure, loading conditions, and degradation behavior. This allows us to quantitatively elucidate the mechanistic transitions in dominant deformation modes during cyclic loading. Our detailed response is organized into three sections: a review of the state-of-the-art, the core scientific contributions of this work, and a summary of the manuscript revisions.

1. Review of existing studies

In experimental characterization, previous studies have employed techniques such as in situ synchrotron XRD, high-resolution digital image correlation (DIC), and TEM **(see Supplementary Table 6)** to observe dislocation accumulation, residual strain evolution, and strain localization in NiTi alloys under cyclic loading. These studies revealed pronounced localized deformation during SED and provided crucial experimental evidence for the interaction between slip and transformation. However, this competition is generally inferred indirectly from static microstructural observations or macroscopic mechanical responses, and thus remains largely qualitative. **Real-time, full-field tracking of the dynamic evolution of both mechanisms remains challenging.**

In theoretical modeling, various constitutive models based on micromechanics or phenomenological theories have successfully simulated cyclic stress-strain responses and degradation trends by incorporating transformation and plastic internal variables. While these models have achieved significant progress in macroscopic predictions, the underlying competition is often simplified as the evolution of model parameters, rather than being directly identified and validated using spatially resolved data from

real microstructures.

In summary, although the interplay between slip and SIMT is a well-known hallmark of SED, existing literature has relied primarily on conceptual or qualitative descriptions. This study bridges this gap by providing, to our knowledge, the first systematic quantification of this competitive evolution through full-field simulations. **The core distinction of our research lies in transforming a classic phenomenological problem into a predictive numerical model, thereby offering a more precise physical foundation for understanding SED.**

Supplementary Table 6 | Research on the SED mechanism of NiTi alloy. Competition defines the analytical depth of the interplay between dislocation slip and martensitic transformation. Spatially resolved indicates whether the study reaches the spatially resolved level of microstructure or deformation

Reference	Method	Competition	Spatially resolved
Prog. Mater. Sci., 2005, 50, 511-678	Review	Conceptual	No
Acta Mater., 2000, 48, 3311–3326	Experiment	Model	No
Prog. Mater. Sci., 2017, 85, 1-42	Review	Conceptual	No
Prog. Mater. Sci., 2018, 98, 249-298	Review	Qualitative	No
Mater. Des., 2017, 134, 111-120	DIC	Qualitative	No
Int. J. Plast., 2015, 72, 60-90	Constitutive model	Model	No
Acta Mater., 2015, 94, 257-270	Synchrotron XRD	Qualitative	No
Acta Mater., 2017, 126, 389-400	TEM	Qualitative	Local
Acta Mater., 2017, 132, 444-454	Cyclic experiments	Qualitative	Local
Acta Mater., 2018, 144, 874-883	XRD+SEM-DIC	Qualitative	Local
Int. J. Plast., 2018, 105, 99-127	Constitutive model	Model	No
Int. J. Plast., 2018, 111, 53-71	Experiment	Qualitative	Local
Acta Mater., 2019, 167, 149-158	Synchrotron XRD	Qualitative	Local

Scr. Mater., 2019, 162, 230-234	Cyclic test + TEM	Qualitative	No
Nat. Commun., 2025, 16, 56775	Microstructure	Qualitative	No
Mater. Des., 2020, 191, 108622	High-resolution DIC	Qualitative	Local
Mater. Sci. Eng. A, 2020, 782, 139271	TEM	Qualitative	Local
Int. J. Plast., 2023, 161, 103487	Cyclic experiments	Model	No
Int. J. Solids Struct., 2023, 280, 112384	Cyclic experiments	Model	No
Int. J. Plast., 2025, 194, 104483	Cyclic experiments	Conceptual	Local

2. Core contributions of this work

This framework enabled us to uncover the following profound insights that have been rarely discussed or remain controversial in the NiTi literature:

Insight 1: Simulation-based identification and validation of martensitic variant transfer across grain boundaries.

We captured the intergranular propagation of martensitic variants during cyclic loading at the simulation level and systematically validated this behavior. The results indicate that variant propagation driven by geometric compatibility is particularly pronounced during the early stages of degradation, especially around the third cycle. By analyzing the propagation characteristics of hotspots, this process can be identified and tracked (see Fig. R2). This finding aligns with recent related research (Acta Mater., 2026, 302, 121623)[8], which reports that transformation pathways begin to stabilize around the third cycle, accompanied by increasingly reproducible behavior in thermal response. These results demonstrate that the proposed framework can capture the evolution of microstructural variants driven by microscale strain gradients derived from Euler orientation information. Such a dynamic process, from local microstructural heterogeneity to global performance degradation, is difficult to resolve using conventional qualitative approaches.

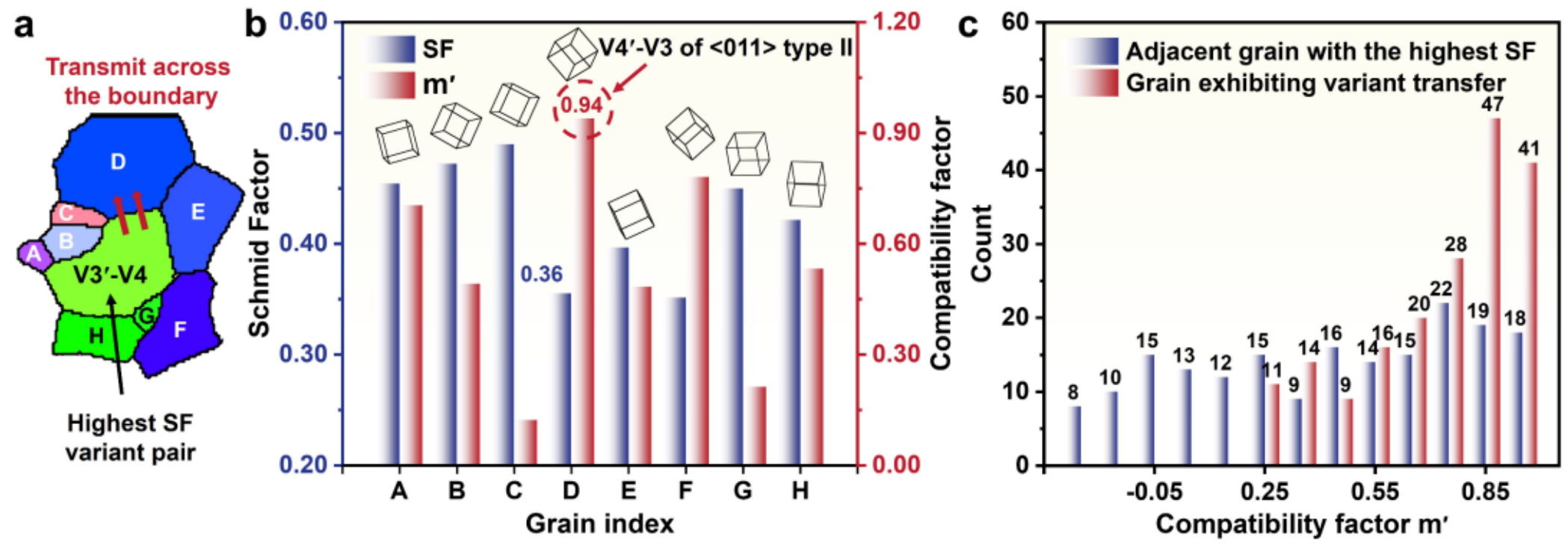

Fig. R2 | Variant transfer across neighboring grains governed by geometric compatibility. a The hotspot grain and its eight neighboring grains A–H were selected for analysis. **b** Comparison of the Schmid factor and geometric compatibility factor m′ of each neighboring grain shows that grain D exhibits the highest m′, with a pathway of V4′–V3 ⟨011⟩ type II variant pair. Such alignment facilitates the propagation of transformation across grains. **c** Among 186 grains, neighboring grains exhibiting actual variant transfer consistently show higher m′ than those with the maximum Schmid factor. It demonstrates that CNN can capture microscale strain gradient from Euler matrices to infer variant transfer directions.

Point 2: Spatial resolution and quantitative characterization of competition between slip and SIMT.

By comparing Grad-CAM heatmaps with Schmid factor distributions, high-sensitivity orientation regions within the EBSD microstructure can be identified. These hotspot regions are further associated with specific slip systems or transformation variants. This capability for spatially resolved analysis under fixed loading stems from the multi-branch feature fusion architecture. During model construction, features from different modalities, including microstructural information and loading conditions, are explicitly integrated while preserving their physical correspondence. Unlike many existing ML studies on metallic materials[9,10], which rely on pre-extracted statistical descriptors or grain-level features, **this structure enables direct visualization under prescribed loading conditions, allowing a clearer characterization of the coupling between microstructure and external loading**.

On this basis, the critical transition interval between the two mechanisms under different strain amplitudes can be identified. For example, at a strain amplitude of approximately 6.38%, the contributions of slip and transformation reach a relative balance. Beyond this threshold, degradation becomes progressively dominated by SIMT in grains with high Schmid factors, characterised by the emergence of {114} austenitic twins during the B2–B19′ cyclic transformation. At lower amplitudes, dislocation slip along the {011}⟨001⟩ and {011}⟨111⟩ systems is preferentially activated, with degradation driven primarily by the progressive accumulation of irreversible defects. This mechanism evolution is further corroborated by DIC full-field strain measurements (see Fig. R3). These findings demonstrate that the framework enables full-field characterization and quantification of competing mechanisms, providing precise guidance for the microstructural optimization of fatigue-resistant NiTi alloys.

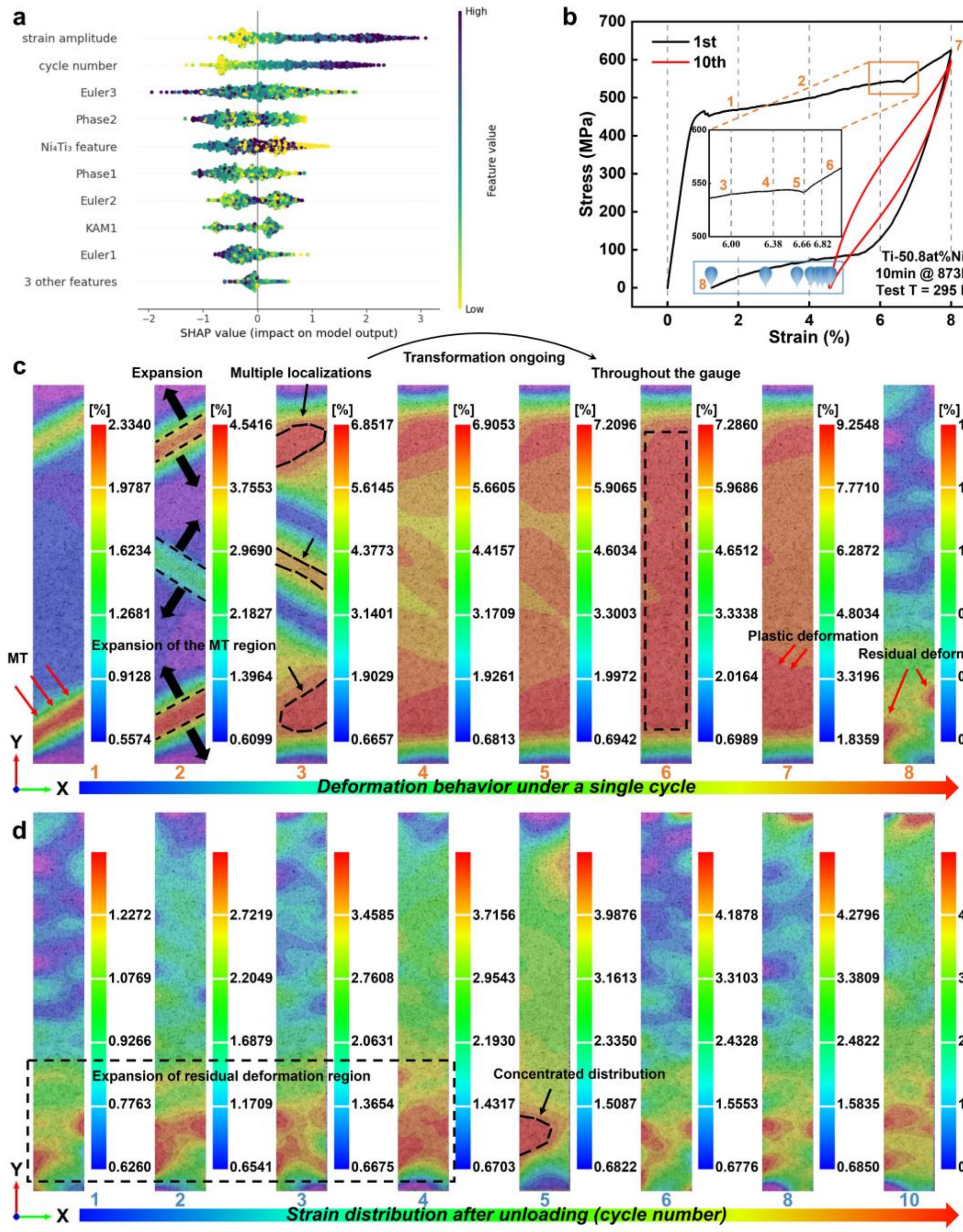

Fig. R3 | Quantitative statistics and mechanistic pathway of superelastic degradation. a SHAP shows that strain amplitude exerts the most decisive influence, followed by the cycle number. **b** Stress-strain curves for the first and tenth cycles. Critical deformation points within a single cycle are labeled 1–8, with blue markers depicting the gradual accumulation of residual strain. **c** DIC maps illustrate key deformation stages corresponding to the markers in (**b**). At 2% strain, the specimen enters the stress plateau and initiates martensitic transformation. The transformation evolves from local nucleation to multiple localizations and ultimately to a fully

transformed gauge section. Notably, the plateau end at 6.66% strain does not coincide with transformation completion; full transformation occurs at approximately 6.82%, and martensitic plastic deformation emerges at 8% strain. **d** DIC maps for successive cycles show the deformation state at the end of each cycle, corresponding to the blue markers in (**b**). The residual deformation region expands continuously during the first five cycles, consistent with the observed orientation-dependent variant transfer.

Point 3: The role of Ni_4Ti_3 precipitates has been revisited.

Contrary to the conventional view that Ni_4Ti_3 precipitates exclusively enhance superelastic stability, this research identifies a dual impact on the degradation behavior (Fig. 6). The results show that finely dispersed Ni_4Ti_3 suppresses dislocation activity through localized stress fields, whereas precipitate coarsening promotes SED, even when most precipitates remain semi-coherent **(Supplementary Figs. 20 and 21)**. Moreover, this effect is not constant but strongly dependent on loading conditions, particularly the applied strain.

This phenomenon can be understood as follows: as the strain amplitude varies, the dominant degradation mechanism also shifts, thereby altering the role of Ni_4Ti_3 precipitates. Specifically, in regimes governed by slip, the dislocation pinning effect becomes a critical factor, whereas under transformation-dominated conditions, its influence on martensite nucleation is more significant. Although such a mechanistic shift is physically plausible, it has seldom undergone systematic investigation in the literature. Utilizing the developed multimodal framework, we employed the SHAP method to quantitatively evaluate the contribution of Ni_4Ti_3 features under diverse amplitudes. **The role of Ni_4Ti_3 in SED should therefore be reconsidered within a framework that couples loading conditions with microstructural evolution.**

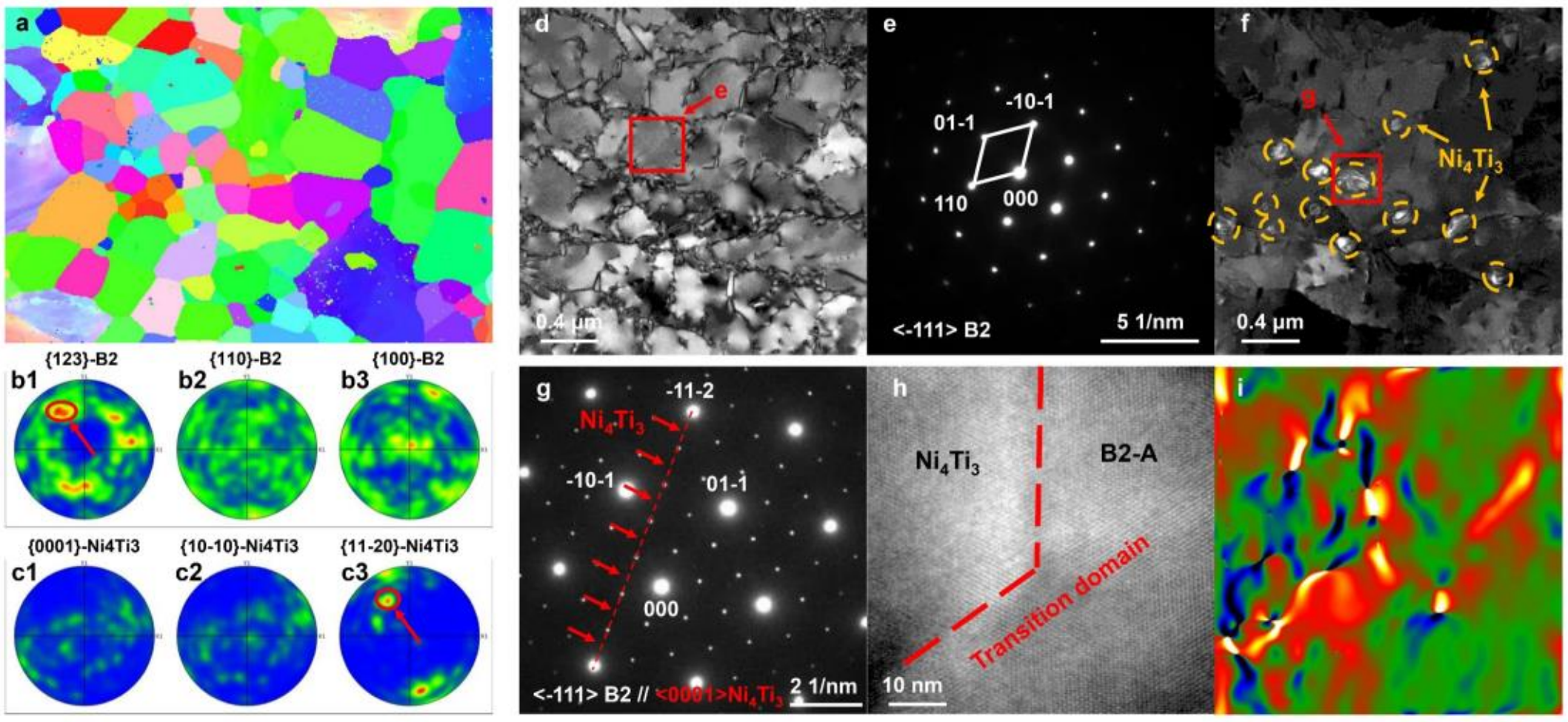

Supplementary Fig. 20 | Orientation relationship between Ni_4Ti_3 precipitates and the B2 austenite matrix. a Orientation distribution map. **b$_1$–b$_3$** {123}, {110}, and {100} pole figures of the B2 austenite matrix. **c$_1$–c$_3$** {0001}, {10-10}, and {11-20} pole figures of the Ni_4Ti_3 precipitates. **d** Bright-field TEM image of the overall region, and (**e**) the selected area electron diffraction (SAED) pattern taken from the red boxed area in (**d**). **f** Corresponding dark-field image, with (**g**) and (**h**) showing the SAED pattern and high-resolution TEM (HRTEM) image from the red boxed region in (**f**), respectively. **i** Geometric phase analysis (Hytch et al., 1998) (GPA) strain map corresponding to (**h**). No common crystallographic plane was identified between Ni_4Ti_3 and the B2 matrix from the pole figures. Nevertheless, the pole figure and SAED analyses reveal a specific orientation relationship of $\{123\}\langle 111\rangle_{B2}$ // $\{11\text{-}20\}\langle 0001\rangle_{Ni4Ti3}$. The dark-field image demonstrates that precipitates are uniformly distributed as nanoscale particles. HRTEM and GPA strain mapping indicate that the formation of Ni_4Ti_3 precipitates induces strain inhomogeneity in the NiTi matrix, with significant local tensile strain in regions of continuous distribution.

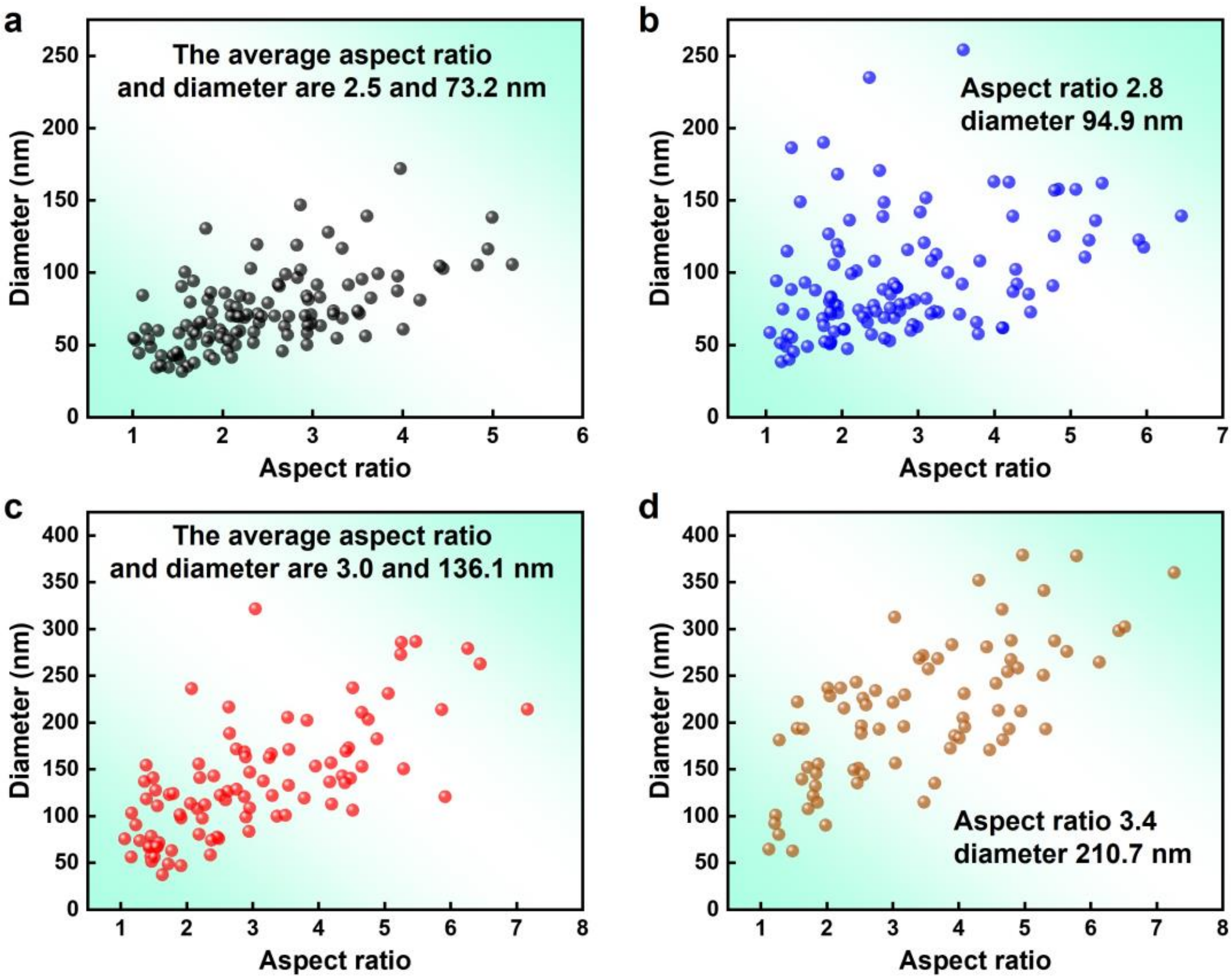

Supplementary Fig. 21 | Quantitative statistics of Ni_4Ti_3 precipitates under different annealing conditions. a–d Aspect ratio–diameter distributions of Ni_4Ti_3 precipitates in Specimens I–IV, corresponding to 500 °C × 10 min, 500 °C × 30 min, 600 °C × 10 min and 600 °C × 30 min, respectively. Each symbol denotes a single precipitate, and the insets show the mean aspect ratio and equivalent diameter. From Specimens I to IV, the data clouds shift toward larger diameters and higher aspect ratios, and the distributions gradually broaden, indicating progressive coarsening and slight elongation of Ni_4Ti_3. The increase in average diameter and aspect ratio is much more pronounced when the annealing temperature rises from 500 °C to 600 °C than when the holding time is extended from 10 min to 30 min, indicating that temperature has a stronger influence on the precipitation behavior than time.

3. Summary of manuscript revisions

The aforementioned table has been incorporated into the **Supplementary information (Supplementary Table 6)**. To improve the clarity of the core findings, the Discussion section has been revised and reorganized. The relevant modifications

(Page 22, Lines 27–28, Page 23 and Page 24, Lines 1–2) are detailed as follows:

On this basis, the competition between slip and SIMT is disentangled across varying strain amplitudes and cyclic stages, yielding a sharper physical landscape than earlier conceptual impressions. Using the Schmid factor (Liu, 2015; Polatidis et al., 2020) as a criterion, we correlated localized hotspots with crystal orientation and stress state. At high amplitudes, degradation is dominated by grains with elevated Schmid factors for SIMT, typified by the emergence of {114} austenite twins during the B2–B19′ cyclic transition. At low amplitudes, dislocation slip on {011}⟨001⟩ and {011}⟨111⟩ systems is preferentially activated, and degradation is driven by the accumulation of irreversible defects. Once this mapping is converted into measurable spatial distributions, we can track the migration of dominant deformation zones across the entire gauge, moving beyond empirical judgments limited to isolated grains or restricted fields of view. The scientific advance arises from transforming previously qualitative, fragmented mechanistic insights into a unified perspective: **SED of NiTi emerges as a competition between mechanisms regulated by loading conditions, with the active pathway selected at the microscale by crystallographic geometric compatibility. This process can now be quantified and predicted in a data-driven manner.**

The longstanding notion (Chen et al., 2019a; Yu et al., 2021a) that Ni_4Ti_3 precipitates are uniformly beneficial is revisited. Through TEM statistics followed by PCA compression (Supplementary Table 3), the influence of Ni_4Ti_3 was found to be conditionally dependent rather than monotonically positive. Under loading regimes that favor SIMT, a moderate dispersion of fine precipitates stabilizes superelasticity by elevating local transformation stresses and strengthening dislocation pinning. Conversely, coarse precipitates coupled with high amplitudes intensify damage and accelerate SED. The pronounced mechanical mismatch at precipitate–matrix interfaces introduces additional stress concentrations (Zhou et al., 2010), while even slight Ni depletion (~0.1 at.%) during nucleation noticeably alters transformation temperatures and stress plateaus (Frenzel et al., 2010), thereby reshaping the

reversible transformation window at room temperature. This underscores that material behavior is not governed by any single rule; treating Ni_4Ti_3 as a standalone variable risks obscuring its detrimental effects under specific conditions. Therefore, this study advocates a systematic analysis method that jointly characterizes multiple factors and their coupling within a multimodal ensemble learning framework, ensuring more reliable and objective assessment.

While the reported training behavior suggests stable convergence, the training and validation loss curves are presented on a linear scale, which limits the ability to rigorously evaluate convergence, particularly during later training stages. Displaying the loss evolution on a logarithmic scale would provide a clearer assessment. Given the high predictive performance, a more explicit assessment of potential overfitting, for example through additional cross-validation or learning-curve analysis, would further strengthen confidence in the model.

1.5 While the reported training behavior suggests stable convergence, the training and validation loss curves are presented on a linear scale, which limits the ability to rigorously evaluate convergence, particularly during later training stages. Displaying the loss evolution on a logarithmic scale would provide a clearer assessment.

1.5 Response: We sincerely appreciate this constructive suggestion. The loss curves in **Fig. 3e** have been converted to a logarithmic scale to better illustrate the convergence behavior during later training stages, as shown here in Fig. R4. The corresponding descriptions have also been updated in the **revised manuscript (Page 11, Lines 6–10)**, as detailed below: The losses of all models dropped rapidly within the first 40 epochs and then stabilized without obvious oscillations or overfitting observed (Fig. 3e and Supplementary Figs. 31, 32), suggesting that the mapping in this task is relatively simple and learnable. MBFFA converged fastest and remained fully stable by the end of training, achieving the lowest final loss (0.02489) among all models.

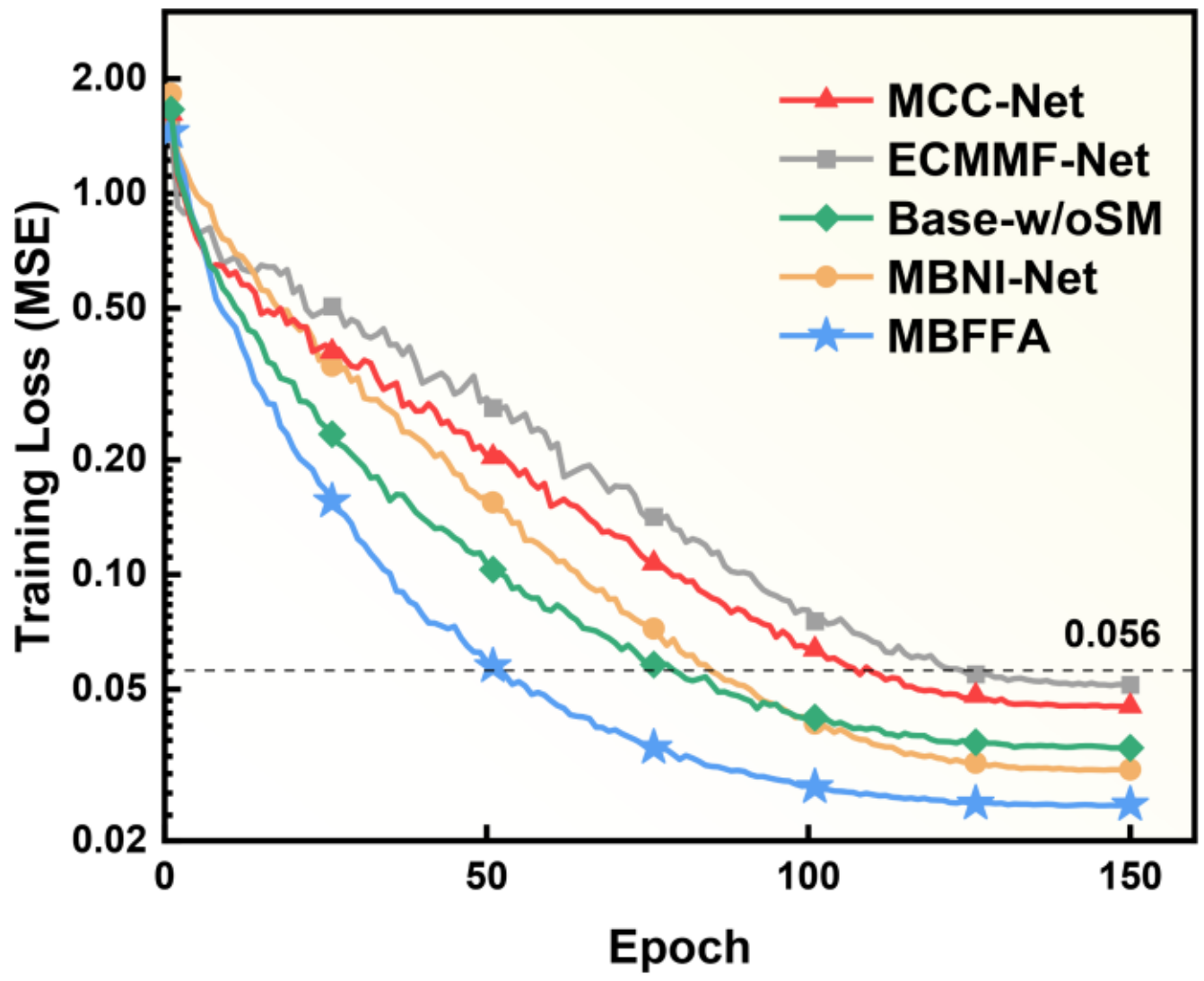

Fig. R4 | The MBFFA converges fastest during training and reaches the lowest final loss.

Additionally, comparative loss curves for the training and validation sets have been included in the Supplementary information **(Supplementary Fig. 31)** to further assess convergence stability.

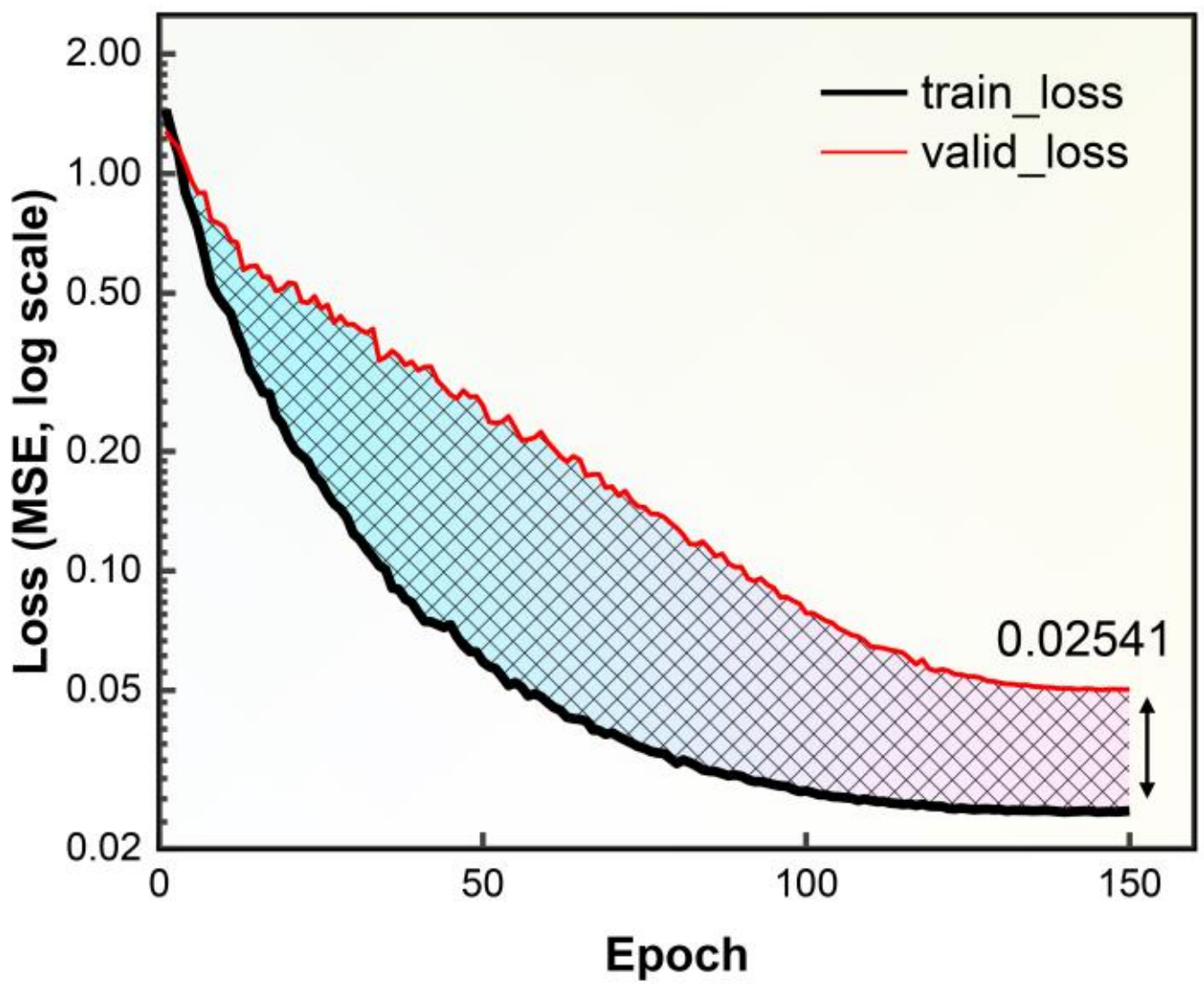

Supplementary Fig. 31 | Evolution of MSE loss during learning. Training and validation losses decrease rapidly and stabilize, followed by minor fluctuations without further reduction. The validation loss remains consistently higher than the training loss, likely due to the high-dimensional nature and relatively small scale of the dataset (Schmidt et al., 2019). At the conclusion of the process, the model had fully converged with a validation loss of 0.0503.

1.6 Given the high predictive performance, a more explicit assessment of potential overfitting, for example through additional cross-validation or learning-curve analysis, would further strengthen confidence in the model.

1.6 Response: We sincerely appreciate your valuable suggestion. Overfitting assessment is essential for enhancing confidence in the model. We have therefore supplemented validation analyses in the revised manuscript and the Supplementary information.

First, as noted above, the data splitting strategy has been clarified in the **Methods section (Page 29, Lines 4–14)**. Data partitioning is performed at the level of original EBSD scan regions to prevent spatial information leakage, with the test set containing only original data and augmentation applied exclusively to the training set. In addition, the training and validation loss curves are provided **(Supplementary Fig. 31)**, showing rapid convergence in the early stages followed by stable behavior without divergence. Taken together with regularization techniques, including early stopping, dropout, and batch normalization, as well as network depth ablation results **(Supplementary Fig. 35)**, the observed gains are independent of model scaling and prolonged training.

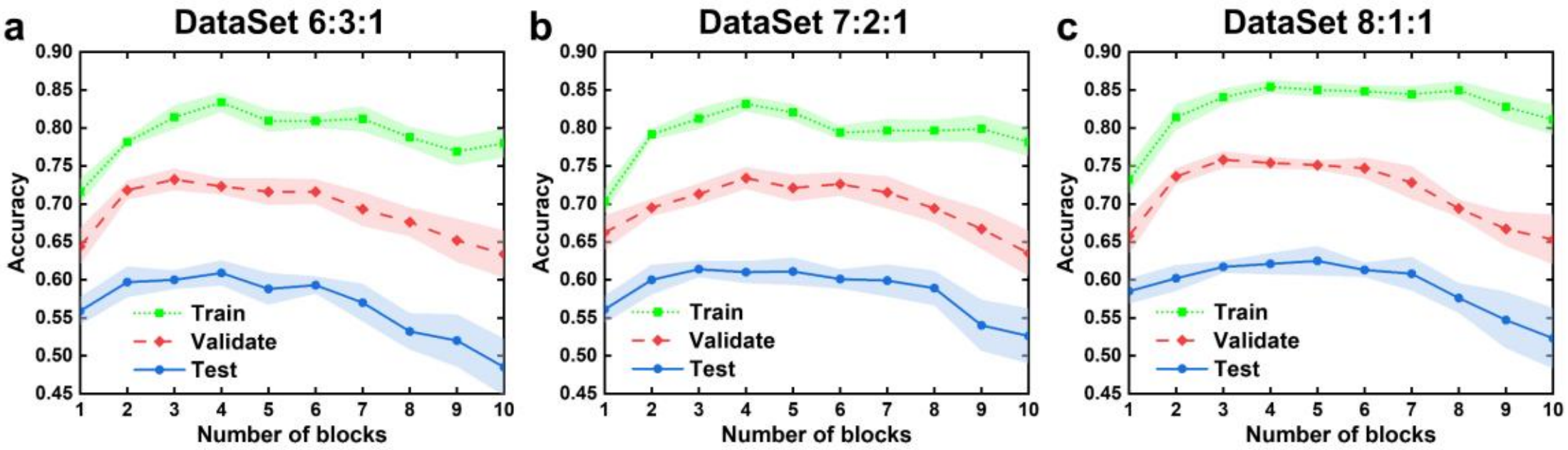

Supplementary Fig. 35 | Sensitivity analysis of data-splitting ratios and network block numbers. Accuracy trajectories for training, validation, and test sets under three partitions: (**a**) 6:3:1, (**b**) 7:2:1, and (**c**) 8:1:1. Accuracy is computed following our previous study (Hu et al., 2025). While the test set was kept constant, increasing the training proportion yielded consistent performance gains, peaking at the 8:1:1 ratio. The influence of network depth showed a parabolic trend, peaking at 3–5 blocks. Further increasing depth provides no additional benefit; instead, it introduces feature

redundancy and representation saturation, leading to reduced stability and a decline in predictive accuracy.

Furthermore, generalization has been quantitatively assessed from two perspectives. (1) Under the 8:1:1 (training/validation/test) split, we conducted 10-fold group cross-validation **(Supplementary Fig. 38)**. Original EBSD regions were used as grouping units, with each test set covering all sample types to ensure reliable evaluation while preventing spatial information leakage. MBFFA consistently achieves the highest R^2 and lowest MSE across all folds, with reduced variance.

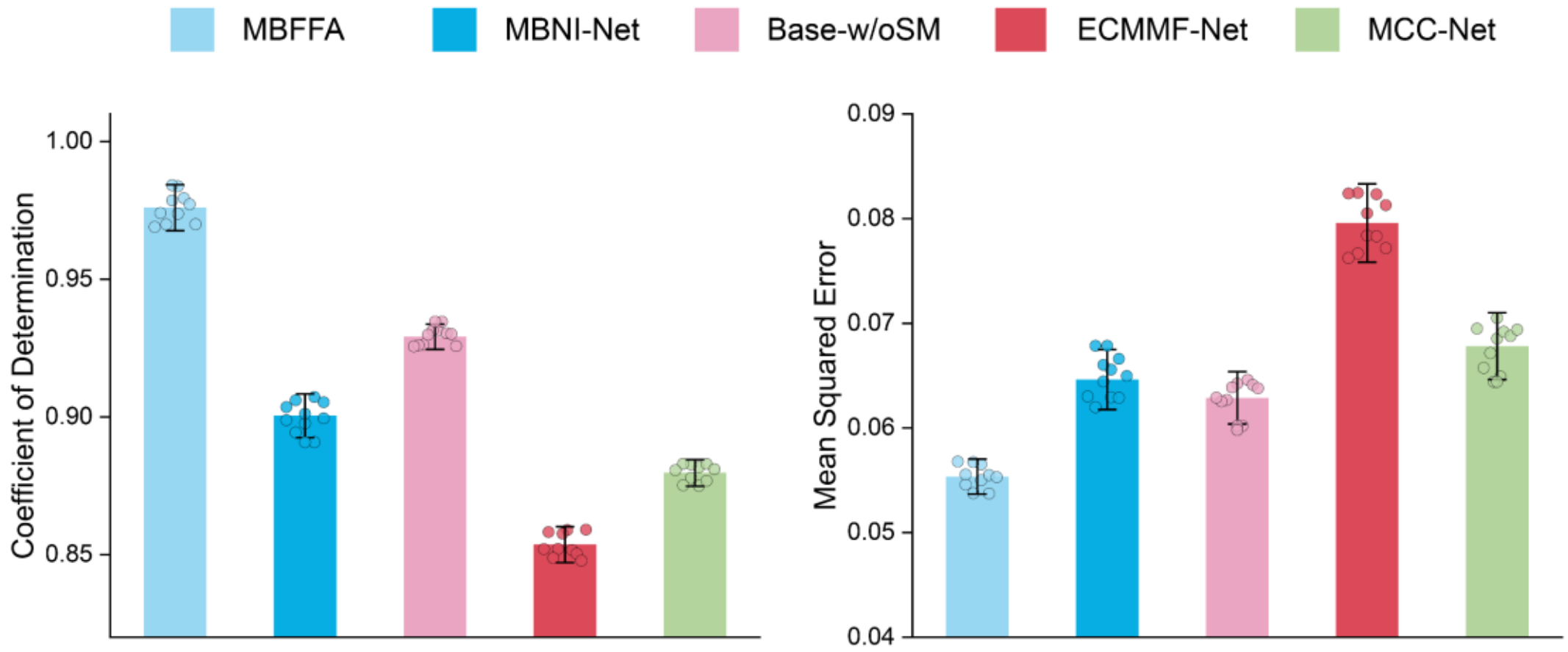

Supplementary Fig. 38 | Model generalization and overfitting assessment based on 10-fold cross-validation. The left and right panels show the coefficient of determination (R^2) and mean squared error (MSE), respectively. Under the fixed 8:1:1 split, 10-fold cross-validation was conducted to evaluate performance stability across data partitions. Samples from the same raw region were grouped to prevent spatial information leakage, and each fold included all sample types in the test set. MBFFA consistently outperformed all benchmarks, achieving the highest R^2 and lowest MSE with the narrowest variance across folds. In contrast, Base-w/oSM and MBNI-Net showed inferior performance, while ECMMF-Net and MCC-Net exhibited both lower accuracy and higher instability. The consistent model ranking across all folds indicates that the results are independent of specific data splits. These findings suggest that MBFFA possesses robust generalization and remains free of significant overfitting despite high-dimensional inputs.

(2) Additionally, learning curve analysis **(Supplementary Fig. 32)** was performed to assess the training data scale. The result is consistent with Supplementary Fig. 35, where performance improves as the training proportion increases from 6:3:1 to 8:1:1. The validation R^2 increases with training size and gradually approaches the training curve. When the proportion exceeds approximately 70%, both curves stabilize and the gap remains nearly constant. This behavior indicates that the model learns stable relationships between multimodal inputs and target responses, **reflecting robust generalization beyond limited-sample fitting**.

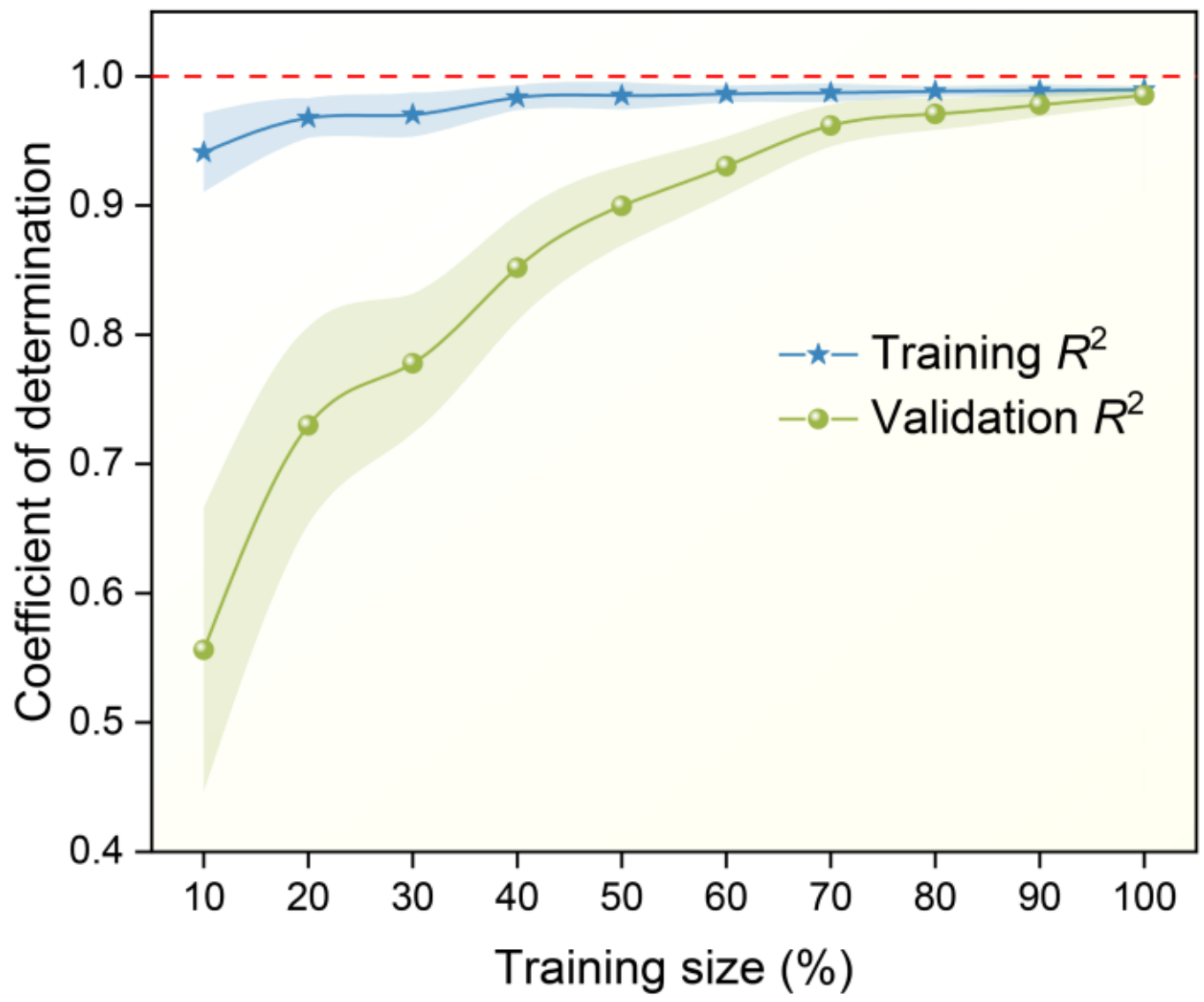

Supplementary Fig. 32 | Learning curve and overfitting assessment. Evolution of R^2 for training (blue stars) and validation (green dots) sets across varying training proportions (10%–100%). Shaded regions represent the standard deviation from random partitions. The narrowing gap and subsequent convergence beyond 70% training data signify that the model effectively learns the underlying multimodal mapping. The close alignment of the two curves at the plateau suggests minimal overfitting and strong generalization performance under the existing data scale.

In summary, analyses of loss evolution, cross-validation, and learning curves highlight the stability of the model. These findings confirm that, under the current data volume and input conditions, the model exhibits no evident overfitting and maintains good generalization.

1.7 The choice of Euler angles as a crystallographic representation also deserves further discussion. Euler space is redundant, which can introduce artificial discontinuities and ambiguity when used as a learning space. Previous studies have shown that quaternion-based representations are often more robust for machine learning tasks involving orientation data. The authors should comment on the potential impact of Euler-angle redundancy on the learned latent representations and whether alternative encodings could improve robustness or interpretability.

1.7 **Response:** Thank you very much for this valuable comment. The choice of orientation representation (Euler angles versus quaternions) has been further assessed through additional analysis and validation, as detailed below.

First, we acknowledge that Euler angles have inherent mathematical limitations. The Euler space is redundant and discontinuous, such that physically similar orientations can manifest as significant numerical discontinuities (e.g., 359° versus 1°). Under certain conditions, gimbal lock-type degeneracies can arise (though not observed in the present data). Moreover, numerical differences in Euler angles do not strictly reflect actual rotational discrepancies. As a result, directly using Euler angles as model inputs may affect the learning process under certain circumstances.

Compared with Euler angles, quaternions provide a continuous, singularity-free parameterization, offering advantages in mitigating coordinate discontinuities and numerical instabilities. However, their double-cover property, where q and $-q$ represent the same rotation, introduces opposite signs but physically equivalent orientations. This can lead to locally inconsistent representations and affect gradient directions during training, often manifested as a pseudo-boundary effect. To address this issue, existing approaches typically impose additional constraints or apply appropriate preprocessing to ensure consistency[11]. Therefore, the practical efficacy of quaternions must be critically evaluated in the context of the specific task.

Given this consideration, we implemented a quaternion-based framework for an ablation study against Euler angles. The quaternion representation yields only marginal performance differences. As reported in **Supplementary Table 9**, the

metrics for re-Quat relative to the base model change from MSE/MAE/R^2 = 0.545/0.168/98.13 to 0.544/0.167/98.15, whereas replacing Euler angles with IPF leads to a more pronounced degradation. We attribute this outcome to the characteristics of the task, in which orientation information is incorporated as a spatial field aligned with the phase and GND maps. Convolutional networks tend to focus on local orientation differences, gradients, and intergranular relationships. Regardless of the chosen parameterization, completely avoiding redundancy or local discontinuities in nonlinear mappings remains a challenge when describing orientations. Recent studies have highlighted that even with quaternions, the processing of large-scale orientation data is hindered by non-Euclidean geometry and representation ambiguities. This has motivated the exploration of higher-dimensional descriptions, such as 6D orientation representations or flattened rotation matrices[12,13], to achieve smoother mappings.

Critically, this research does not fall within orientation regression tasks, which are highly sensitive to the geometric properties of rotational coordinates. The limitations of Euler angles have minimal impact, as the model processes three-channel input analogous to RGB images to recognize spatial patterns. Experimental results support this point: **the model successfully resolves strain gradients associated with variant transfer using only the Euler orientation field, with spatial distributions consistent with geometric compatibility analysis**. Additionally, this representation directly corresponds to raw EBSD data and preserves the full three degrees of freedom of crystallographic orientation. Although quaternions improve local continuity, they offer limited benefit for global spatial feature extraction in this task. Therefore, the Euler-based representation is retained in this study. We have made the following revisions:

First, in the **Dataset generation section (Page 7, Lines 18–25)**, a brief discussion of the redundancy and discontinuity of Euler angles has been added, clarifying why their impact remains limited under the current framework:

Each sequence contained three aligned EBSD mappings: (1) Euler matrix: The Euler

angles (Φ_1, Φ, Φ_2) of each pixel were encoded into a three-channel $H\times W\times 3$ orientation field. Unlike inverse pole figure maps, which are limited by color mapping, this representation preserves the complete crystallographic orientation. **Although Euler angles may exhibit intrinsic redundancy and discontinuities, they are treated here as spatially structured fields. By employing local convolutions, the model primarily captures orientation gradients and their spatial correlations, thereby mitigating the impact of such discontinuities on feature learning**.

Second, in the **Supplementary information (Page 51, Lines 1-22)**, the discussion has been expanded to include a systematic comparison of alternative encodings, including Euler angles, IPF, and quaternions:

Why Euler orientation matrix instead of alternative encodings? We encode the three Euler angles into three channels to construct the Euler matrix that represents the actual crystal orientation, rather than directly using the more readily available IPF map. The IPF map essentially compresses the orientation of a selected reference direction in the crystal frame into RGB colors, where a single color may correspond to multiple crystallographic orientations (Supplementary Fig. 36a). In contrast, the Euler matrix fully describes all three degrees of freedom. Interestingly, this approach unexpectedly introduces a new modality, yet it can still be processed in the same manner as an image matrix. **It should be noted that Euler angle parameterization may introduce redundancy and discontinuities. To address this, we constructed a control model based on quaternion representation, which has been widely adopted as a mathematically robust and non-singular alternative to Euler angles for crystallographic orientation representation and EBSD learning tasks (Jangid et al., 2022; Mason and Schuh, 2008). The results indicate that, compared to the Euler angle representation, it did not yield significant performance improvements. This may be attributed to the fact that, in the current task, model learning is primarily dominated by orientation variations and their spatial distributions, thereby reducing dependence on specific parameterization forms. Therefore, in this study, the Euler matrix is sufficient to characterize the relevant**

information. If future work extends to modeling local orientation, continuous orientation encodings such as quaternions may offer advantages.

re-IPF: In this ablation variant, we replace the Euler matrix with the IPF map as the orientation input.

re-Quat: In this ablation variant, we replace the Euler matrix with the quaternion matrix (encoded into four channels) as the orientation input.

Supplementary Table 9 | Ablation study of model components in MBFFA

Method	MSE (10^{-1})	MAE	R^2 (10^{-2})
w/o-PCA	0.642	0.314	85.19
w/o-GND	0.634	0.307	86.26
w/o-Phase	0.579	0.202	89.89
re-FSM	0.571	0.198	93.52
**re-IPF**	**0.567**	**0.188**	**95.37**
ad/BC	0.558	0.179	97.05
re-KAM	0.552	0.172	98.02
**re-Quat**	**0.544**	**0.167**	**98.15**
**Base**	**0.545**	**0.168**	**98.13**

Overall, we sincerely appreciate your insightful comment, which prompted a more systematic evaluation of alternative encodings. In the revised manuscript, the use of Euler angles is supported by discussion and controlled validation, thereby enhancing the rigor and transparency of the representation selection.

1.8 Relatedly, although the authors emphasize multimodal fusion, the interaction between modalities within the latent space is not clearly examined. The framework appears to merge modalities effectively, but the structure and organization of the resulting latent representation are not investigated, which limits interpretability.

1.8 **Response:** Many thanks for your insightful comment. It is greatly appreciated. While the original paper emphasized the gains from multimodal fusion, it lacked

direct analysis of inter-modality interactions in the latent space and the organization of the fused representations. To address this limitation, we have supplemented the revised manuscript with analyses from three perspectives: (i) the role of the feature synergy module in the fused representation. (ii) the evolution of inter-modality relationships before and after fusion. (iii) the organization of the resulting latent representation.

First, UMAP visualizations of feature vectors before and after the synergy module are shown in **Fig. 2i, j**, colored by true residual strain. After the synergy module, the latent space becomes more organized, with clear and continuous residual strain gradients, indicating improved cross-modal alignment. Accordingly, the following description **(Page 9, Lines 2-6)** has been added: UMAP visualizations (Fig. 2i, j and Supplementary Fig. 41) show that the feature synergy module produces a more organized latent space with a distinct continuous gradient of true residual strain. Combined with the cosine similarity analysis, this indicates that the module effectively facilitates cross-modal representation alignment and promotes a more coherent encoding of deformation-related information.

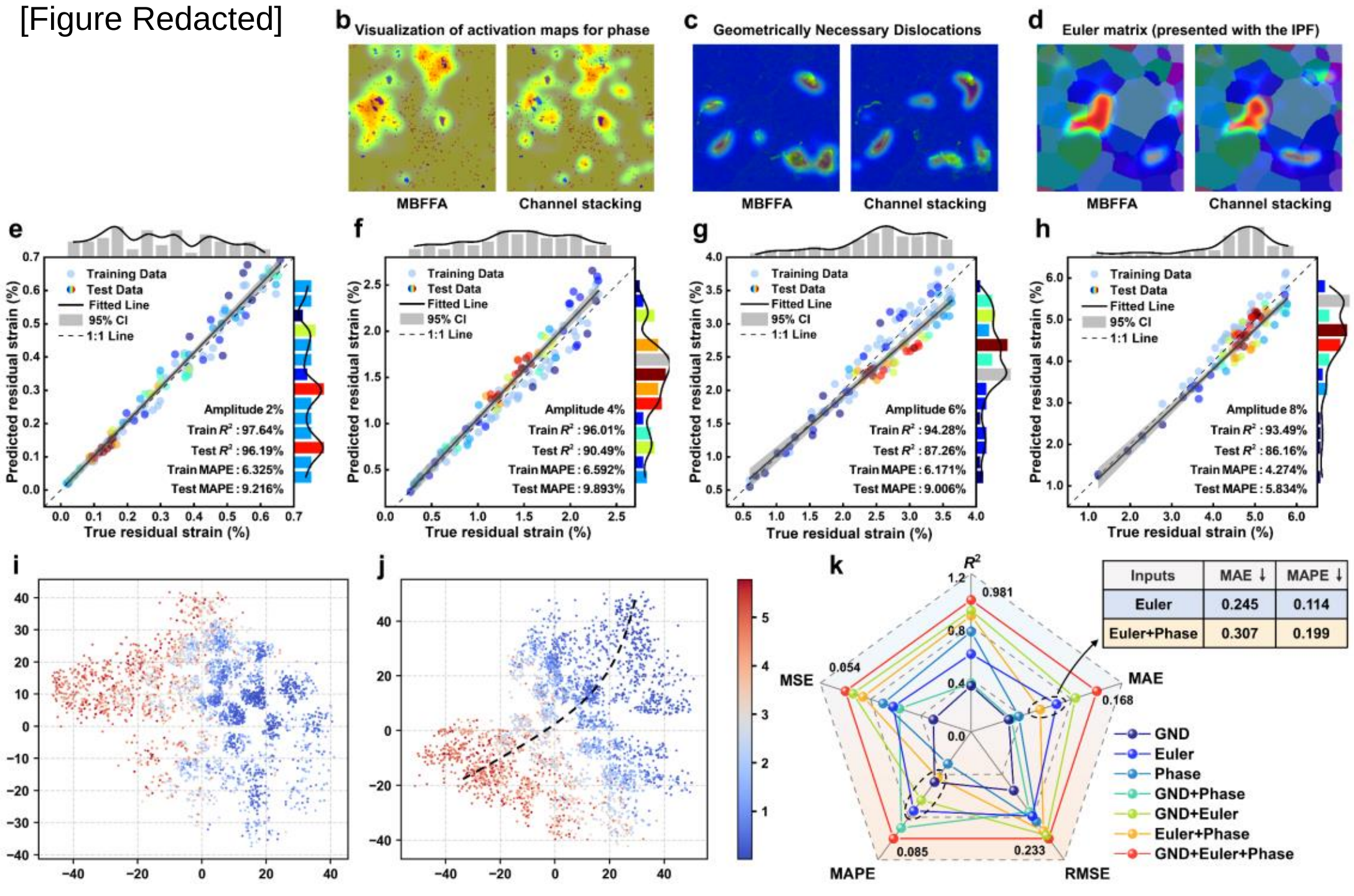

Fig. 2 | Structural evaluation and performance of MBFFA. a The design draws inspiration from the human visual system, where two streams separately process

recognition and localization, enabling complementary information processing. To compare feature extraction strategies, the channel stacking approach (Ren et al., 2023) and MBFFA were visualized using Grad-CAM on the phase images (**b**), GND maps (**c**), and Euler matrices (**d**). The MBFFA focuses more precisely on physically meaningful regions, including phase boundaries, areas of concentrated lattice bending, and orientation discontinuities. **e–h** Across strain amplitudes, the predictions consistently align with experimental trends. **i, j UMAP visualizations of high-dimensional features before and after the feature synergy module, colored by the true residual strain. After fusion, the latent space exhibits a more orderly structure with distinct continuous strain gradients. k** Multi-source inputs demonstrate advantages, but the improvements are not consistent across metrics, highlighting the need for careful feature fusion design.

Second, we have supplemented the **Supplementary information** with a pairwise similarity analysis of the three image modalities **(Supplementary Fig. 41)**, including Euler–GND, Euler–phase, and GND–phase, based on cosine similarity distributions before and after the synergy module. Before fusion, the average similarity of these modality pairs is approximately 0.09–0.14, indicating that the modalities remain largely independent in the latent space. Among them, Euler–GND exhibits slightly higher similarity, suggesting a potential intrinsic correlation between crystallographic orientation and local lattice distortion. After the synergy module, the distributions shift toward higher similarity ranges, with average values increasing to 0.38–0.49, reflecting enhanced cross-modal interaction and successful extraction of shared information. Meanwhile, the distributions retain a finite spread rather than collapsing into identical homogeneous representations, indicating that the module strengthens modality synergy without inducing representation collapse.

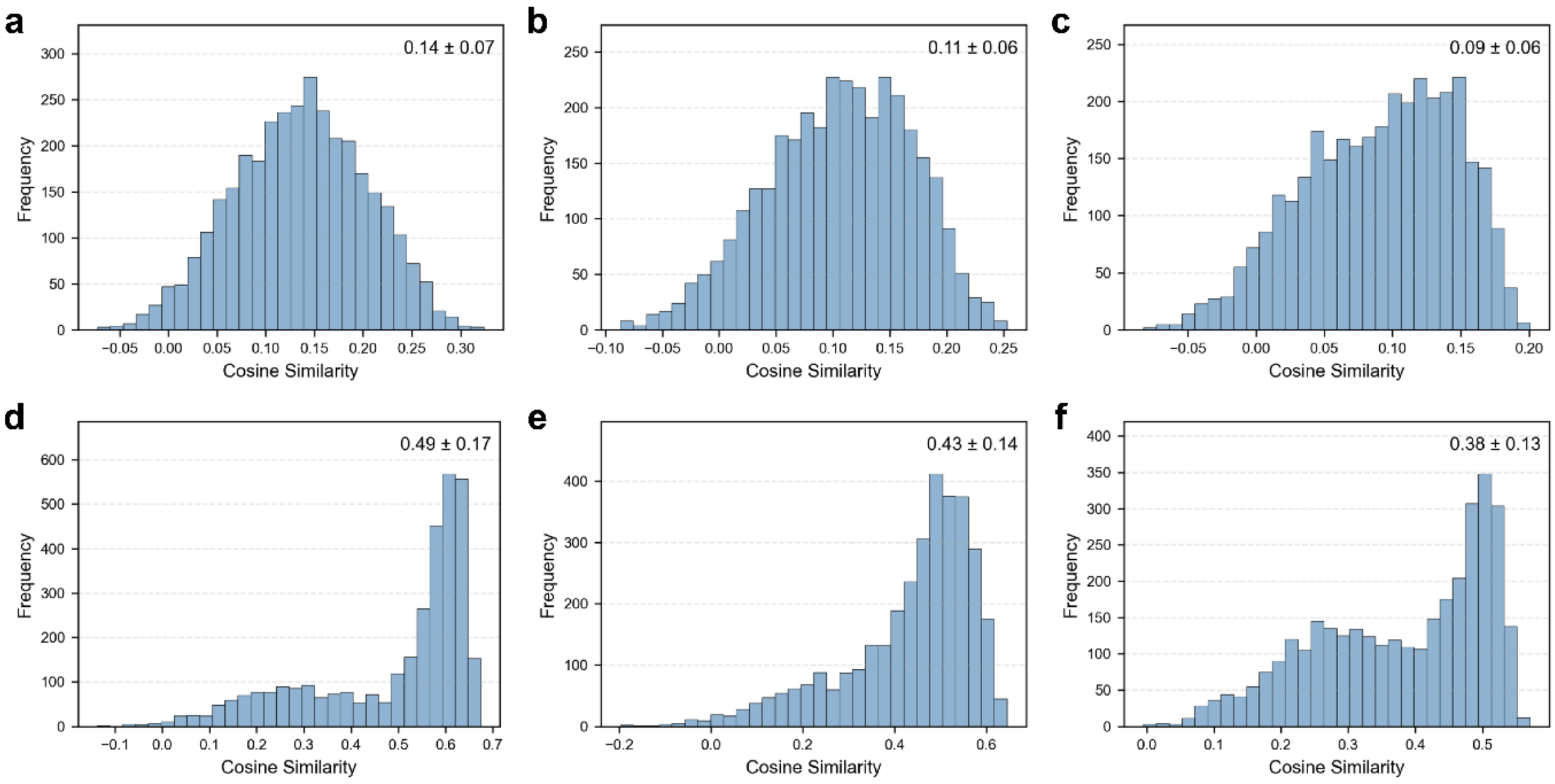

Supplementary Fig. 41 | Evolution of cosine similarity distributions for multimodal features in the latent space. Pairwise cosine similarities (Leitherer et al., 2021) were calculated from training samples for convolutional features of three image modalities: Euler, Phase, and GND. **a–c** show the distributions for Euler–GND, Euler–phase, and GND–phase pairs before the synergy module; **d–f** present the corresponding results after fusion. Before fusion, mean similarities range between 0.09 and 0.14, suggesting strong independence between modalities; the marginally higher Euler–GND similarity points to an intrinsic correlation between crystallographic orientation and localized lattice distortion. Post-fusion, the distributions shift significantly toward higher intervals (0.38–0.49), indicating markedly enhanced cross-modal alignment and successful extraction of shared information. Notably, the distributions maintain a finite width, suggesting that modalities are not compressed into homogeneous spaces. Instead, the synergy module establishes robust inter-modal coupling while preserving the distinct characteristics of each modality, effectively mitigating representation collapse.

Finally, the organization of the latent space was established in our previous ablation studies. In this revision, we compare the number of latent feature representations (n = 1–6) extracted from the convolutional branches (Supplementary Fig. 34). Performance is optimal when three feature representations are extracted from each

image-modality branch, whereas further increasing this number leads to higher errors. **We attribute this to the numerical modality consisting solely of scalar features**. If the image modality representations become excessively rich, they tend to dominate during the fusion process, thereby causing inter-modal scale imbalance and weakening the fusion efficacy[14]. In contrast, a moderate number of feature representations provides a better balance between expressiveness and redundancy.

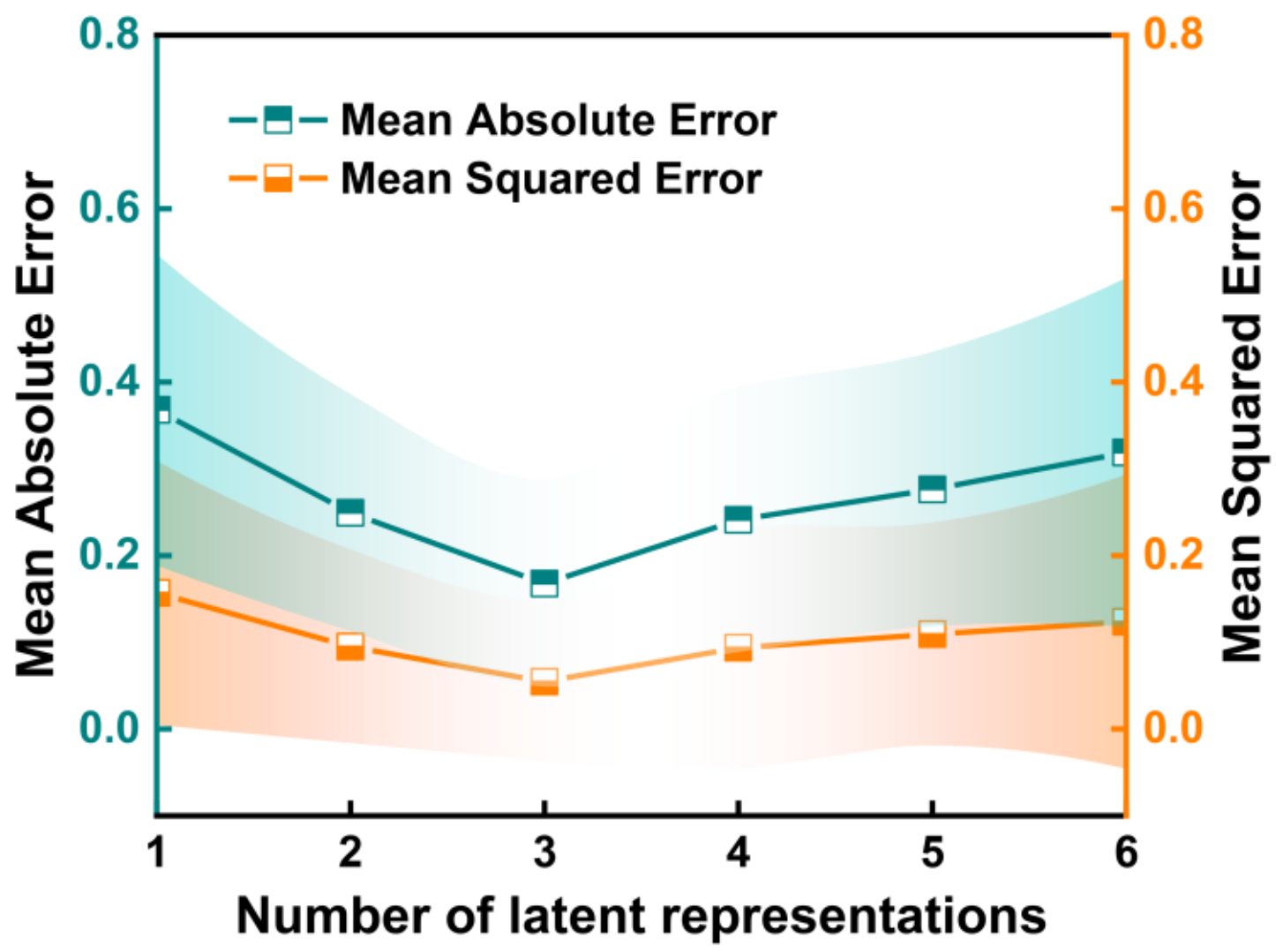

Supplementary Fig. 34 | Ablation study on the number of convolutional feature representations. This figure depicts the evolution of prediction errors under varying counts of latent feature representations (n = 1–6). Optimal performance across both metrics is attained when the image-modality branch extracts three feature representations. Further increases in feature dimensionality result in higher errors, likely due to modality imbalance. Given that the numerical modality comprises only scalars, an overly complex image representation may dominate the fusion stage, leading to scale mismatch and reduced synergy (Na Narong et al., 2025). Consequently, a moderate number of representations strikes an optimal balance between informational expressiveness and redundancy control.

For a more in-depth analysis, UMAP comparisons **(Supplementary Fig. 40)** under three settings (n = 2, 3, and 4) are shown. At n = 2, the representation space is compressed, with obvious mixing in the transition region and insufficient separability

for cycle number and Ni_4Ti_3 features, resulting in limited representational capacity. At n = 4, the latent manifold exhibits evident fragmentation, with samples of similar residual strain dispersed across multiple local clusters, suggesting that redundant features may undermine the stability of the representation.

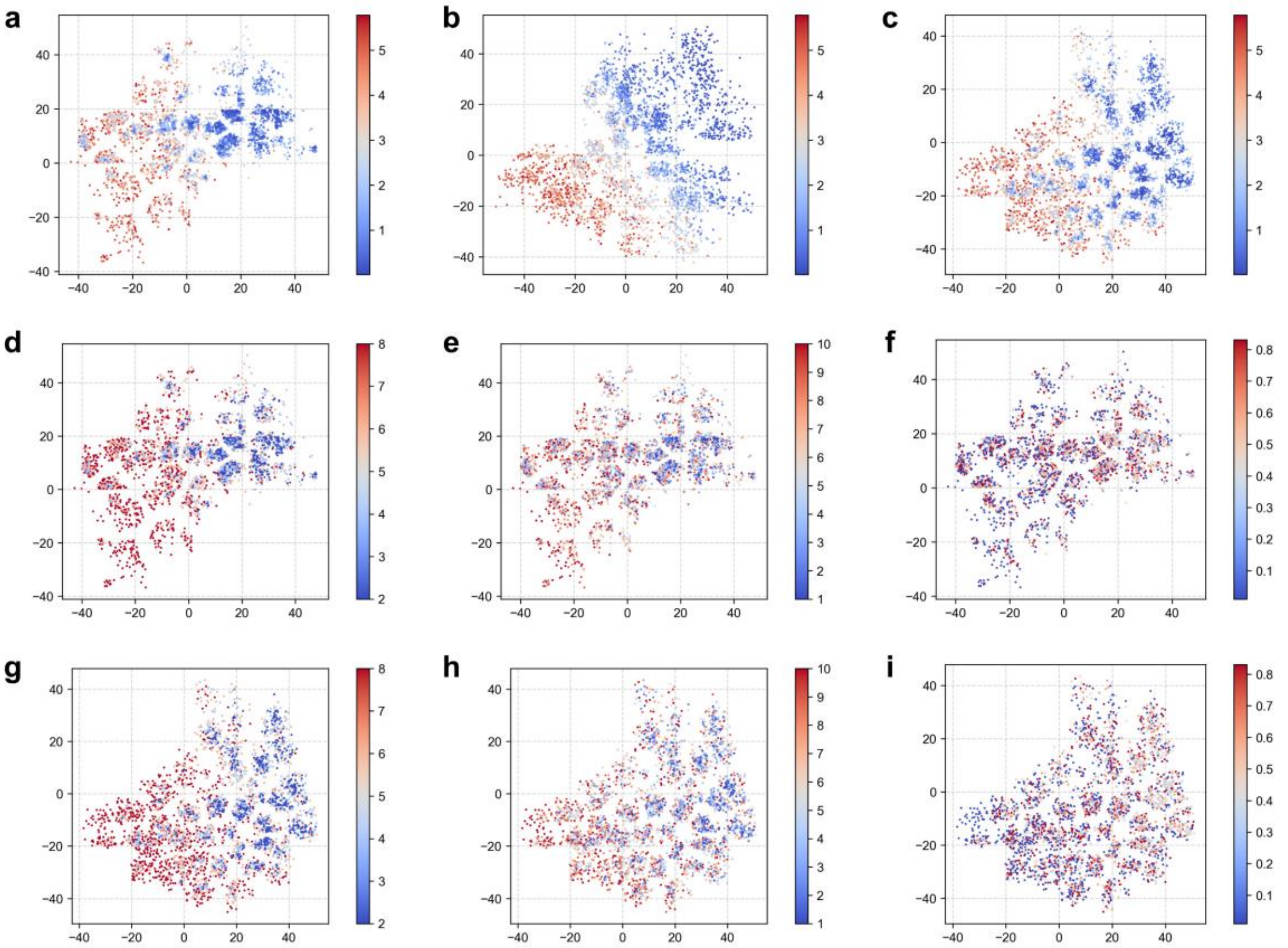

Supplementary Fig. 40 | UMAP manifold evolution and multi-physical mapping across feature dimensions. This figure evaluates how the number of latent feature representations (n = 2, 3, 4) extracted by the convolutional branch influences latent space organization. **a–c** show embeddings colored by residual strain for n = 2, 3, and 4, respectively. **d–f** and **g–i** illustrate latent spaces for n = 2 and n = 4 mapped against strain amplitude, cycle number, and Ni_4Ti_3 descriptor. As shown in **a–c**, n = 3 yields the most continuous residual strain gradient, characterized by smooth transitions and minimal overlap. Conversely, n = 2 yields a compressed distribution with significant mixing in transition zones. Combined with **d–f**, it is evident that while strain amplitude forms a dominant partition, information regarding cycle number and Ni_4Ti_3 remains insufficiently resolved, limiting representational capacity. At n = 4, the embedding becomes fragmented and disjointed, with similar residual strain values

dispersed across isolated clusters. Although the global structure governed by strain amplitude is preserved (as seen in **g–i**), local variations associated with cycle number and Ni_4Ti_3 are amplified, suggesting that redundant features degrade representational stability.

In summary, through these supplementary analyses, we hope to clearly demonstrate that the proposed framework effectively merges multimodal data and forms synergistic representations in the latent space that are structured, interpretable, and physically grounded with respect to the output.

1.9 Given the emphasis on deformation mechanisms and local plasticity, the inclusion of geometrically necessary dislocation (GND) maps could be considered. GND density provides a more direct physical measure of lattice curvature and plastic incompatibility than KAM alone and could further strengthen the physical grounding of the framework.

1.9 **Response:** We sincerely appreciate your insightful suggestion. As noted, GND density provides a more direct physical measure than KAM for characterizing lattice curvature and plastic incompatibility, thereby strengthening the physical foundation of our framework. We have implemented a comprehensive revision. Specifically, all relevant EBSD data were reprocessed, and the model was fully retrained accordingly. The manuscript, figures, captions, and Supplementary information have been updated to ensure consistency with the revised framework.

To illustrate these revisions, we briefly outline two representative modifications. In the **Dataset generation section (Page 7, Lines 26-28)**, we have made corresponding adjustments to the data construction: (3) GND map: Quantifies the spatial distribution of geometrically necessary dislocation density, reflecting local lattice curvature and crystallographic heterogeneity within the initial microstructure.

In the **Model evaluation section (Page 9, Lines 7-11)**, we have also implemented revisions: Input configurations from EBSD sources were evaluated. Among single-source settings, the phase image performed best (R^2 = 0.745, MAE = 0.441),

outperforming methods using only the Euler matrix or the GND image. Dual inputs delivered significant improvements: MAE values of 0.523, 0.307, and 0.202 for GND + phase, Euler + phase, and Euler + GND, respectively.

Given that the revisions related to GND extend throughout the manuscript and Supplementary information, we do not list all individual revisions to figures and text here. Two key points are highlighted regarding the incorporation of GND.

(1) To assess the practical impact, we conducted a multi-level analysis. First, the average GND densities under different annealing conditions were quantified and, together with representative maps **(Supplementary Fig. 36c)**. Second, comparative Grad-CAM visualizations under different feature extraction strategies were provided **(Fig. 2b-d and Supplementary Figs. 45 and 46)**. The results show that, compared with channel stacking and KAM-based approaches, the GND-based scheme produces sharper boundaries and more stable localization in spatial responses, while also influencing phase and orientation channels. Furthermore, local interpretability analysis based on SHAP was supplemented **(Supplementary Fig. 43)**. The results indicate that GND-related features (GND1) exhibit stronger positive contributions under mild annealing conditions, which diminish with increasing annealing intensity. It indicates that the model effectively captures information related to lattice curvature and local strain concentration from GND maps. Collectively, the introduction of GND brings substantial improvements at both the prediction and interpretability level.

Meanwhile, although GND has been incorporated into the prediction framework and its effectiveness validated through multiple analyses, a systematic investigation of the microscopic dislocation dynamics underlying GND evolution (e.g., multiscale characterization as performed for crystal orientations or Ni_4Ti_3 precipitates) has not been conducted due to experimental constraints and manuscript scope. **This limitation has been explicitly acknowledged in the revised manuscript and identified as an important direction for future work.**

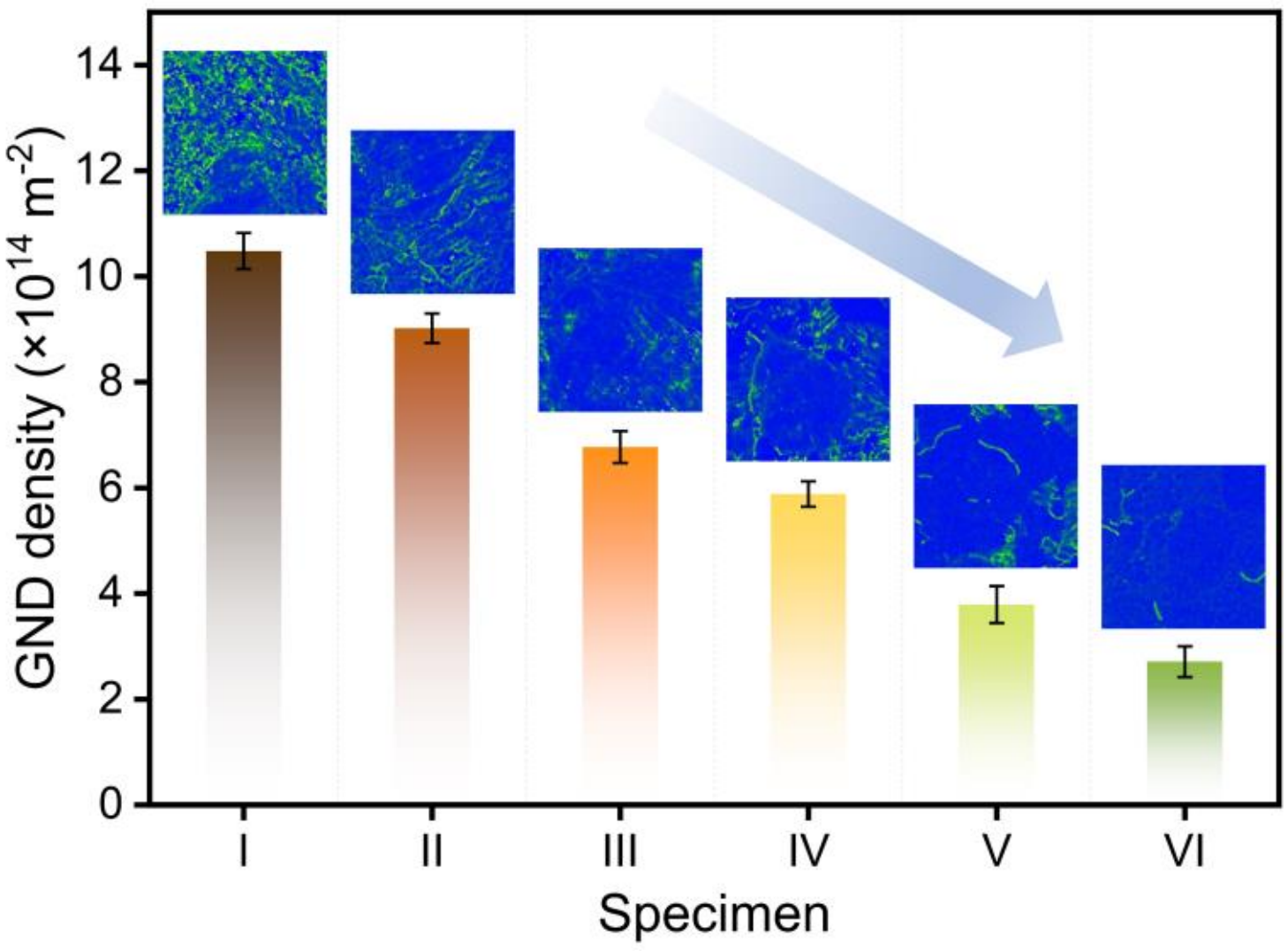

Fig. R5 | Mean GND density for specimens I–VI subjected to various heat treatments, with representative GND maps for each specimen shown above.

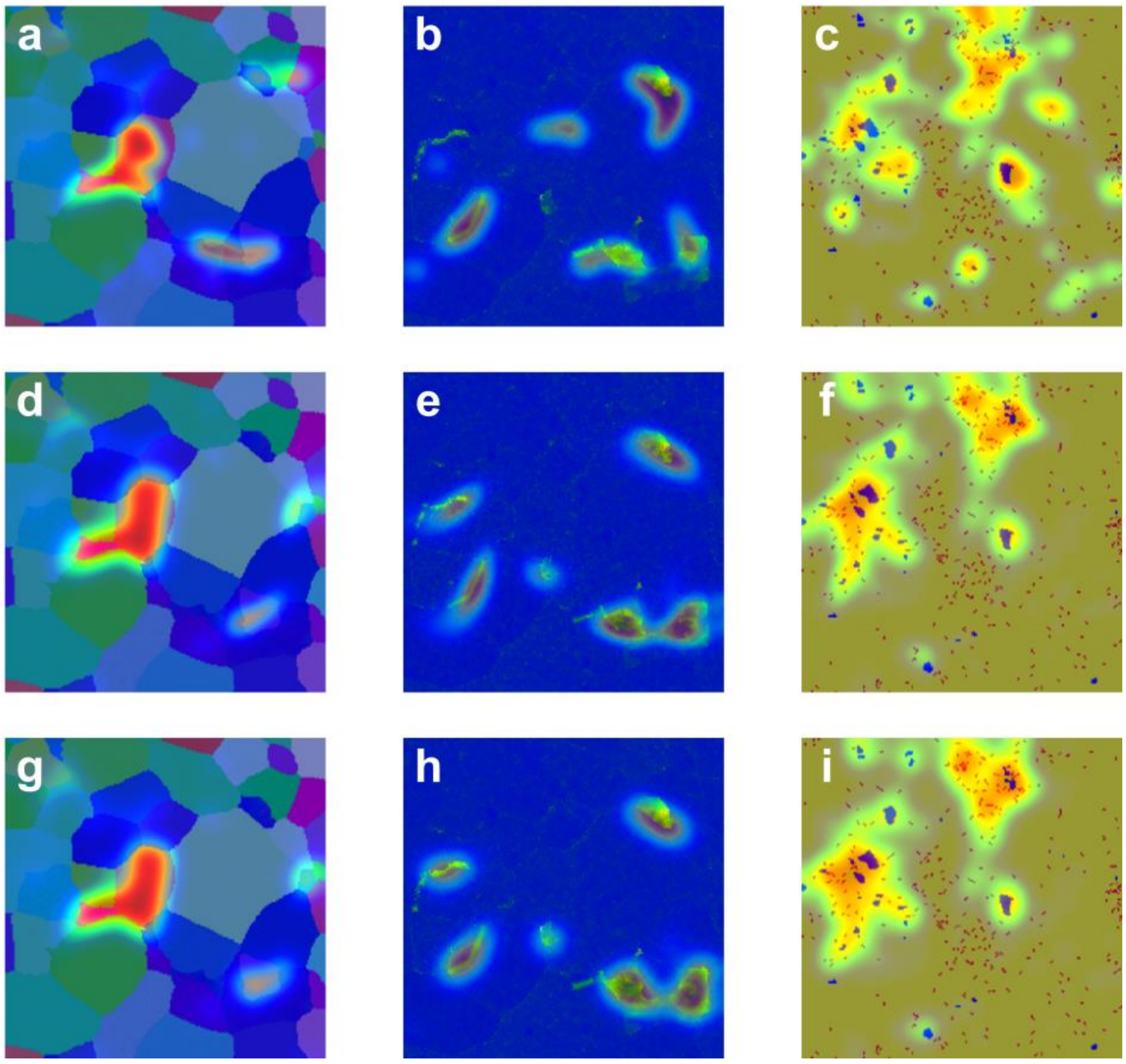

Supplementary Fig. 45 | Comparison of Grad-CAM visualizations under varied feature extraction strategies. a–c Heatmaps generated using the channel stacking strategy for orientation (IPF), GND, and phase maps. **d–f** Results using the previous method (KAM-based), corresponding to Euler, KAM, and phase maps, respectively; **g–i** Results from the proposed method. Comparison shows that channel stacking produces a dispersed response with poor feature correspondence. The KAM-based approach reflects orientation gradients but suffers from spatial misalignment. Conversely, the proposed GND-based method achieves well-defined boundaries and stable localization, capturing dislocation-induced lattice curvature and plastic incompatibility more effectively. Crucially, when individual modalities are activated in isolation, the transition from KAM to GND also modulates the response distributions of the phase and orientation channels, revealing a degree of coupling among modalities.

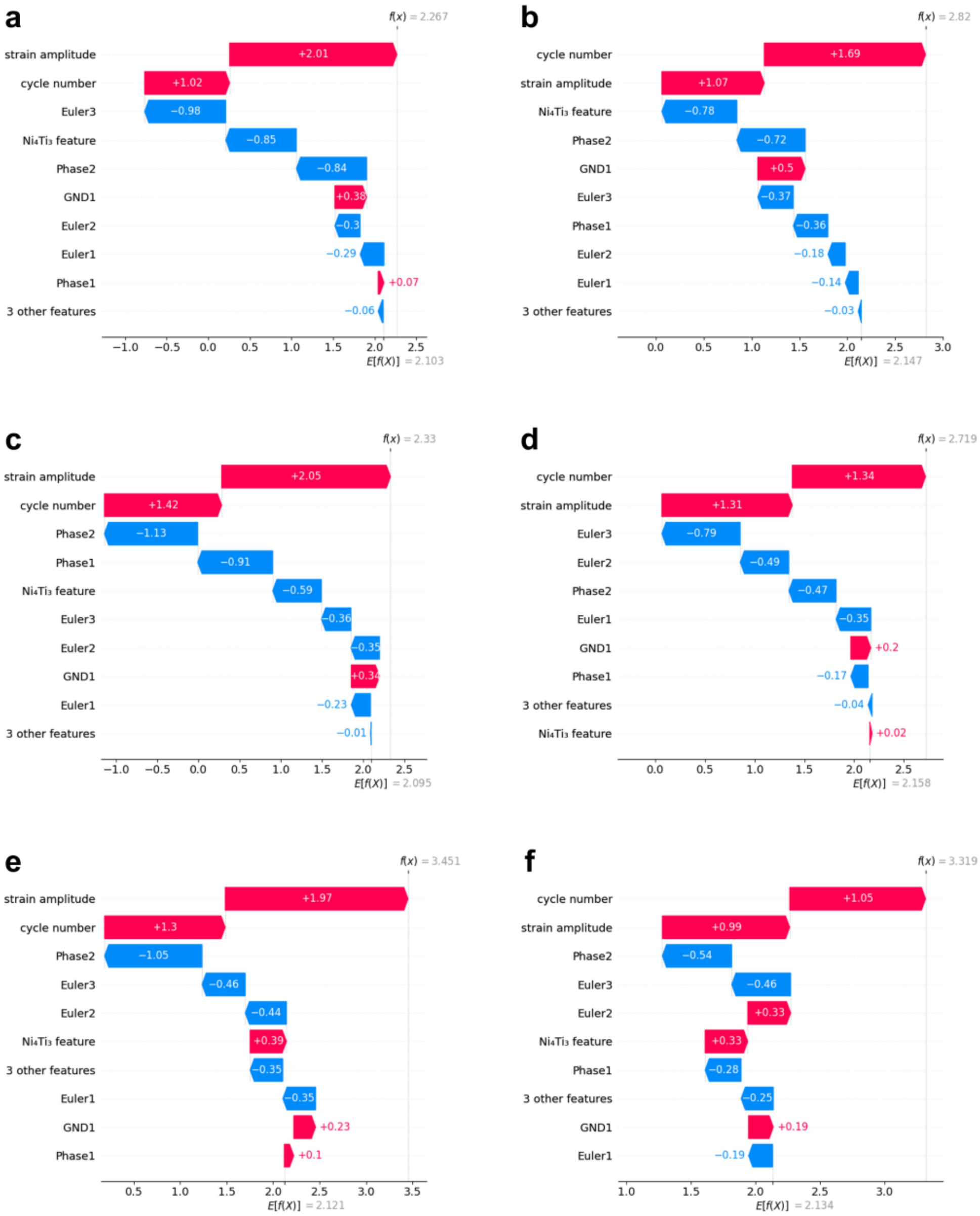

Supplementary Fig. 43 | Local interpretability of residual strain predictions across six annealing conditions. Results are shown for specimens I–VI at 6% strain after 10 cycles. **a–f** SHAP waterfall plots detailing feature attributions for each specimen. Overall, strain amplitude and cycle number act as the dominant positive contributors across all specimens. The Ni_4Ti_3 feature generally provides a strong negative contribution, confirming that fine, dispersed precipitates mitigate degradation. In contrast, the contribution of the GND1 feature is more pronounced in lightly annealed specimens and decreases at higher annealing intensities. This trend suggests that GND1 effectively captures localized strain concentrations that gradually relax during annealing.

(2) We compared the GND, KAM, and their fusion strategies in the **Supplementary information (Supplementary Table 10)**. Under the same architecture, replacing KAM with GND yields a modest performance improvement. However, simply introducing GND alongside KAM (KAM+GND) results in reduced performance. We reduced the number of feature representations per channel from three (v1) to two (v2). The performance degradation persists, indicating that it is not due to increased feature dimensions. Based on prior observations, further expansion to higher-dimensional settings was not pursued, as redundancy typically increases with feature count, and existing results already identify three representations as optimal. The degradation is more likely attributable to interference from redundant information due to the partial overlap and inconsistency between KAM and GND.

Based on this comparative analysis, GND provides a more explicit physical representation of local plastic deformation than KAM. Accordingly, in the revised manuscript, we have updated the framework to use GND to characterize localized plasticity, thereby enhancing the physical grounding while maintaining the streamlined architecture of the model.

Supplementary Table 10 | Comparative prediction performance of GND and KAM features under varied fusion strategies. KAM+GND v1 and v2 denote configurations with three and two feature representations per channel, respectively. GND alone achieves a slight improvement over KAM. However, incorporating GND into a fused representation degrades performance. Reducing the number of feature representations (v2) does not mitigate this effect, indicating that the degradation is not primarily caused by increased feature dimensionality, but is more likely attributed to feature redundancy and physical inconsistency

Configuration	MSE (10^{-1})			MAE		
	Train	Val	Test	Train	Val	Test
KAM+GND v2	0.365	0.708	0.826	0.137	0.189	0.210

KAM+GND v1	0.298	0.589	0.695	0.126	0.172	0.191
KAM	0.235	0.496	0.552	0.115	0.160	0.172
GND	0.249	0.503	0.545	0.116	0.160	0.168

We sincerely thank you again for this valuable suggestion. This comment has significantly improved the manuscript, strengthening its physical foundation, methodological utility, and novelty. We hope that the above revisions clearly demonstrate the role and significance of GND characterization in this study.

1.10 The current analysis relies entirely on two-dimensional EBSD data, whereas superelastic degradation and transformation pathways are inherently three-dimensional phenomena. A brief discussion of how three-dimensional microstructural effects might influence the conclusions would be valuable.

1.10 **Response:** We sincerely appreciate this insightful and forward-looking comment. This issue addresses a fundamental challenge in the microstructural characterization of SMAs. The crystal deformation, SED, and phase transformation pathways are intrinsically three-dimensional processes, whereas current EBSD-based characterization is inherently limited by its two-dimensional nature. It inevitably overlooks the out-of-plane stress components and the influence of subsurface grains. For example, the Schmid factors and geometric compatibility factors calculated in this study primarily reflect apparent crystallographic constraints. **In real 3D microstructures, however, the surrounding grain environment governs the stress states of subsurface grains and influences both variant selection and strain transfer across grain boundaries.** This limitation may introduce deviations between the theoretical predictions and the actual deformation behavior.

In the revised manuscript, this issue is addressed jointly with Comment 14 (data-driven correlations versus physical causality) and Comment 17 (the influence of EBSD preprocessing). Instead of a simplified treatment, this consolidation stems from the methodological connections among these three issues.

The interpretable ML framework is designed to identify high-dimensional correlation structures in multimodal data. When applied to examine existing mechanistic hypotheses, it provides quantitative, spatially resolved evidence, supported by Schmid factor and geometric compatibility analyses. On the other hand, when based on 2D EBSD data, preprocessing procedures (e.g., denoising intensity and step size) may affect hotspot localization accuracy and the characterization of subtle orientation gradients. Accordingly, a dedicated paragraph has been added to the **Discussion section (Page 25, Lines 21-28 and Page 26, Lines 1-3)** to explicitly clarify these limitations and their potential implications. We further note that future work can combine advanced characterization techniques, including 3D EBSD and X-ray tomography, to address the gap between planar characterization and bulk deformation behavior. The specific revisions are as follows:

Furthermore, as the present work relies on 2D EBSD data, the bulk stress states and intergranular constraints governing transformation pathways and slip-SIMT competition are inevitably simplified (Abdolvand et al., 2018; Hestroffer et al., 2024). This approximation neglects out-of-plane stress components and subsurface grain interactions, potentially constraining the representativeness of Schmid factor analysis and the interpretation of microstructural evolution. In this context, EBSD preprocessing (e.g., denoising intensity and step size) directly influences the precision of hotspot localization and may even obscure subtle orientation gradients. While the identified physical trends are unlikely to be attributable to preprocessing variations and are supported by multiscale experiments and simulations, systematic evaluation of model sensitivity remains essential for further optimization.

1.11 The use of PCA to reduce precipitate statistics to a single descriptor is reasonable, but the manuscript does not report how much variance is retained or lost through this reduction.

1.11 **Response:** We apologize for omitting the critical information regarding the

variance contribution of Principal Component Analysis (PCA) in the original manuscript.

During the construction of the Ni_4Ti_3 precipitate descriptor using PCA, the first principal component (PC1) retains 83.45% of the variance from the original precipitation statistics, losing only approximately 16.55% of the information. The single descriptor can adequately represent the main characteristics of the precipitation. Accordingly, the following revisions have been made:

1. We have clarified the variance proportion retained by PC1 in the **Dataset generation section (Page 8, Lines 1-5)**, thereby explicitly indicating the extent of information preservation during dimensionality reduction. The relevant revision is provided below: The precipitation state was represented by a single numerical descriptor using principal component analysis (PCA) (Abid et al., 2018) of Ni_4Ti_3 precipitate statistics obtained from transmission electron microscopy. The first principal component, which captures 83.45% of the standardized variance, was used as the precipitate descriptor in the numerical branch.

2. **A dedicated section has been added to the Supplementary information.** The relevant content is provided below:

Construction of the Ni_4Ti_3 precipitate descriptor by principal component analysis: To encode the precipitation state as a single numerical input while avoiding redundancy among correlated TEM statistics, we reduced the Ni_4Ti_3 measurements to a one-dimensional descriptor using principal component analysis (PCA) (Gorgannejad et al., 2019; Niezgoda et al., 2011). TEM observations were first summarized into statistical features describing diameter, aspect ratio, and projected area, followed by PCA performed on the correlation matrix of the dataset. The explained variance ratio of component *k* was calculated as $\eta_k = \lambda_k / \sum_j \lambda_j$, where λ_k is the eigenvalue associated with principal component *k*. **The first principal component (PC1) captured the dominant covariance of precipitate evolution and explained 83.45% of the total variance, whereas PC2 and PC3 accounted for 15.23% and 1.32%, respectively.** Consequently, the one-component representation retained 83.45% of the

original variance while discarding 16.55%. The corresponding loading matrix is provided in **Supplementary Table 3**.

The precipitate descriptor used in the numerical branch was defined as the PC1 score. Given the arbitrary sign of PCA eigenvectors, PC1 was normalized using a sigmoid function such that a larger value corresponds to a coarser precipitation state. For the conditions in which no precipitates were observed, a small constant value of 0.01 was assigned to avoid zero-valued inputs that may induce numerical instability during network training. This dimensionality reduction preserves the essential microstructural information while preventing overweighting of mutually correlated precipitate features in the numerical branch. Consistently, the unreduced precipitate statistics lead to inferior predictive performance **(Supplementary Table 9)**.

Supplementary Table 3 | PCA loading matrix and explained variance for the Ni_4Ti_3 precipitate descriptor

Principal component	Diameter loading	Aspect ratio loading	Projected area loading	Explained variance ratio
PC1	0.5893	0.5212	0.6173	0.8345
PC2	-0.5008	0.8352	-0.2271	0.1523
PC3	-0.6340	-0.1753	0.7532	0.0132

Supplementary Table 9 | Ablation study of model components in MBFFA (partial results). w/o-PCA: We omit the PCA step and directly concatenate all individual Ni_4Ti_3 precipitate features into the numerical input branch

Method	MSE (10^{-1})	MAE	R2 (10^{-2})
w/o-PCA	0.642	0.314	85.19
Base	0.545	0.168	98.13

1.12 The choice of residual strain as a single scalar output is experimentally convenient and justified, but it necessarily compresses the complexity of superelastic degradation into one metric. Other degradation descriptors, such as hysteresis width, transformation stress evolution, or energy dissipation per cycle, are not considered. A short justification of this choice, or a discussion of possible multi-output extensions, would strengthen the work.

1.12 Response: We greatly appreciate your thoughtful comment. Describing superelastic degradation (SED) using a single scalar inevitably compresses characteristics of the cyclic response. In the revised manuscript, we clarify the rationale for adopting residual strain as the primary descriptor and expand the Supplementary information to incorporate additional degradation indicators and alternative prediction targets.

The selection of residual strain as the primary output is motivated by its status as a well-established and widely adopted descriptor of cyclic functional degradation in NiTi alloys. Previous studies have shown that SED typically manifests as a gradual accumulation of residual strain, accompanied by reductions in forward transformation start stress (i.e., the onset stress for SIMT, also termed transformation stress) and hysteresis loop area (dissipated energy). Residual strain, transformation stress, and dissipated energy are identified as three key indicators for characterizing cyclic functional degradation[15].

More importantly, residual strain exhibits the most direct correspondence with accumulated irreversible deformation. Such irreversible strain arises from dislocation activity, transformation-induced plasticity, and the retention of residual martensite or other transformed structures[16]. The residual strain serves as a direct scalar measure of the progressive loss of recoverable deformation capacity. In contrast, although transformation stress and dissipated energy also contain important information, they depend more strongly on hysteresis loop morphology and are more sensitive to testing conditions, including temperature, strain rate, and loading path[17,18]. For example, dissipated energy may exhibit non-monotonic dependence on strain rate.

Based on these considerations, residual strain is adopted as the single label in this study, while other indicators are treated as candidates for multi-output extensions.

Four corresponding revisions have been made to the manuscript. First, in the **Dataset generation section (Page 8, Lines 5-8)**, we state the rationale for selecting residual strain as the degradation descriptor. The relevant modifications are as follows: The target label was the residual strain measured at the end of each cycle, which was adopted as the degradation metric because it provides a direct and consistently extractable measure of the accumulated irreversibility during cyclic SED.

Second, in the **Room temperature cyclic tensile loading and unloading section (Page 28, Lines 8-12)**, we add clarification indicating that residual strain is more robust than descriptors dependent on hysteresis loop morphology. The relevant modifications are as follows: Residual strain (ε_{res}), defined as the strain at the intersection of the unloading curve with the strain axis, was selected as the primary degradation metric. Compared with hysteresis-based descriptors such as hysteresis width or dissipated energy, ε_{res} provides a more robust measure of the cumulative loss of recoverable deformation capacity.

Third, in the revised **Supplementary information (Supplementary Figs. 10, 11 and Supplementary Table 1)**, statistical results for transformation stress and dissipated energy have been added:

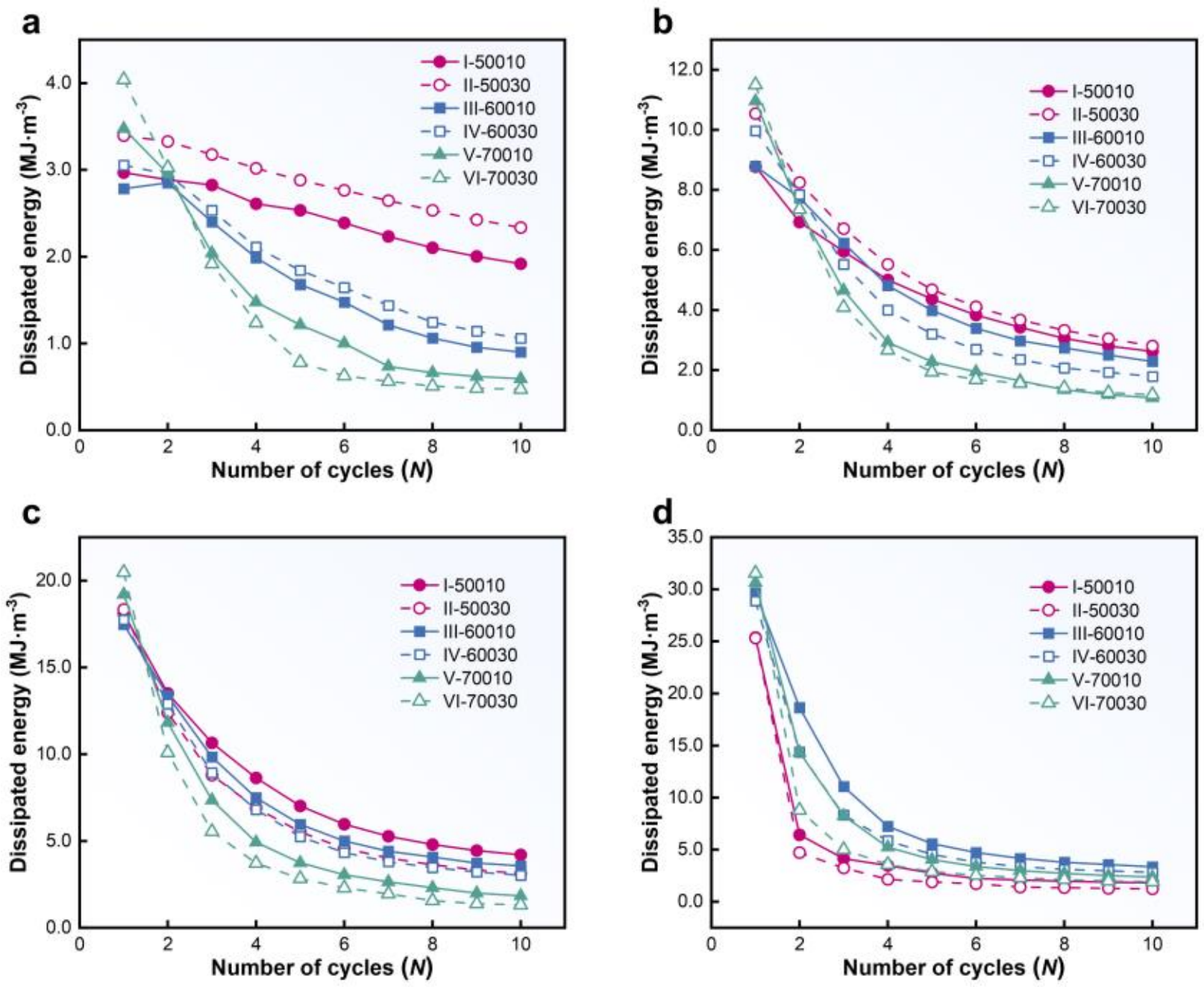

Supplementary Fig. 10 | Dissipated energy density of NiTi alloys under various annealing conditions. Evolution of dissipated energy density with increasing cycle number at strain amplitudes of (**a**) 2%, (**b**) 4%, (**c**) 6%, (**d**) 8%. These measurements were conducted under room-temperature quasi-static conditions.

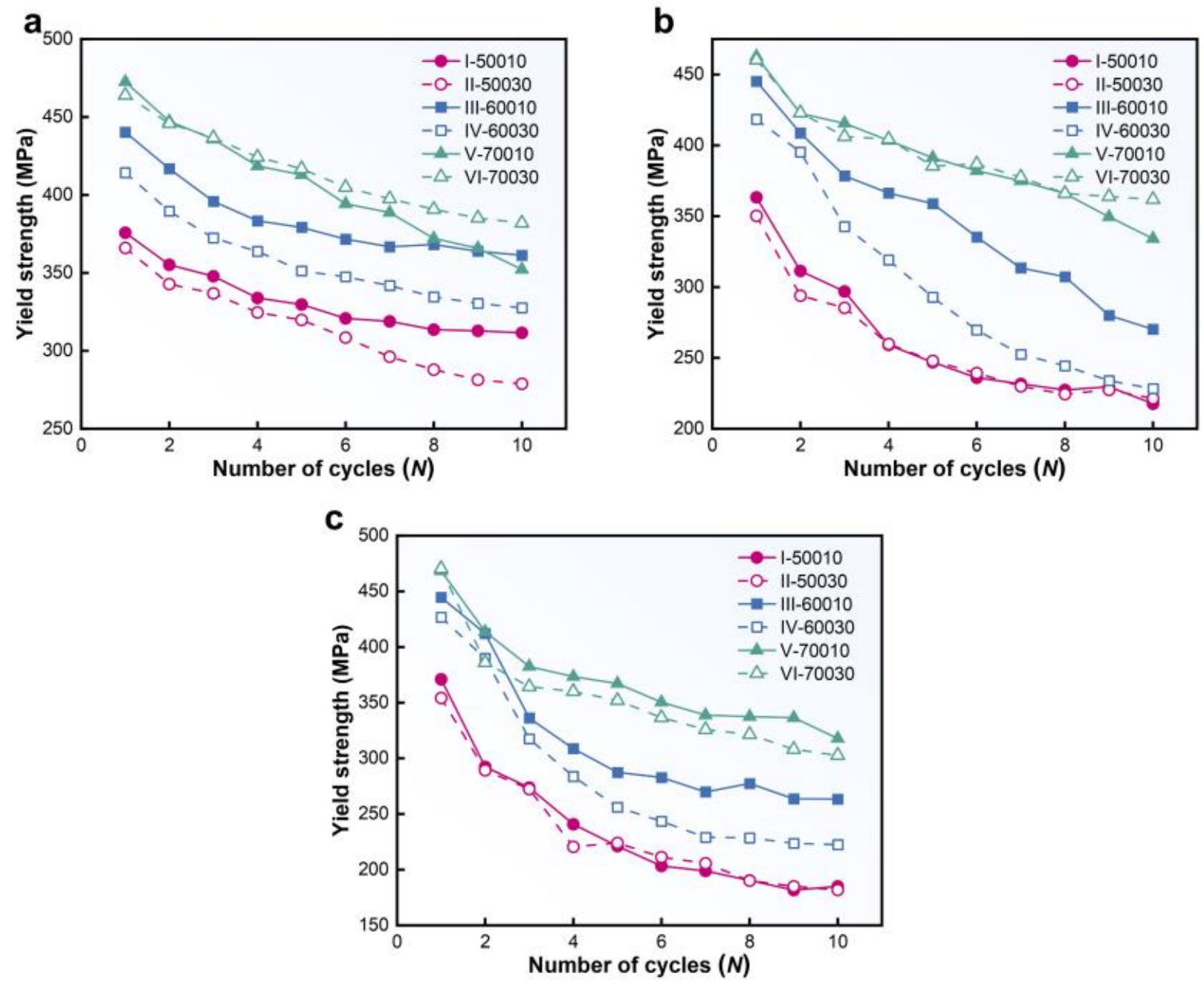

Supplementary Fig. 11 | Forward transformation start stress of NiTi alloys under various annealing conditions. Evolution of transformation stress with increasing cycle number at strain amplitudes of (**a**) 2%, (**b**) 4%, (**c**) 6%. These measurements were conducted under room-temperature quasi-static conditions.

Supplementary Table 1 | Transformation stress of NiTi specimens (I–VI) during the first loading cycle at a strain amplitude of 8%

Specimen	I	II	III	IV	V	VI
Transformation stress (MPa)	374.36	360.76	457.68	415.48	491.24	453.41

Fourth, prediction experiments using transformation stress and dissipated energy as alternative targets are presented in the **Supplementary information (Supplementary Figs. 50 and 51)**:

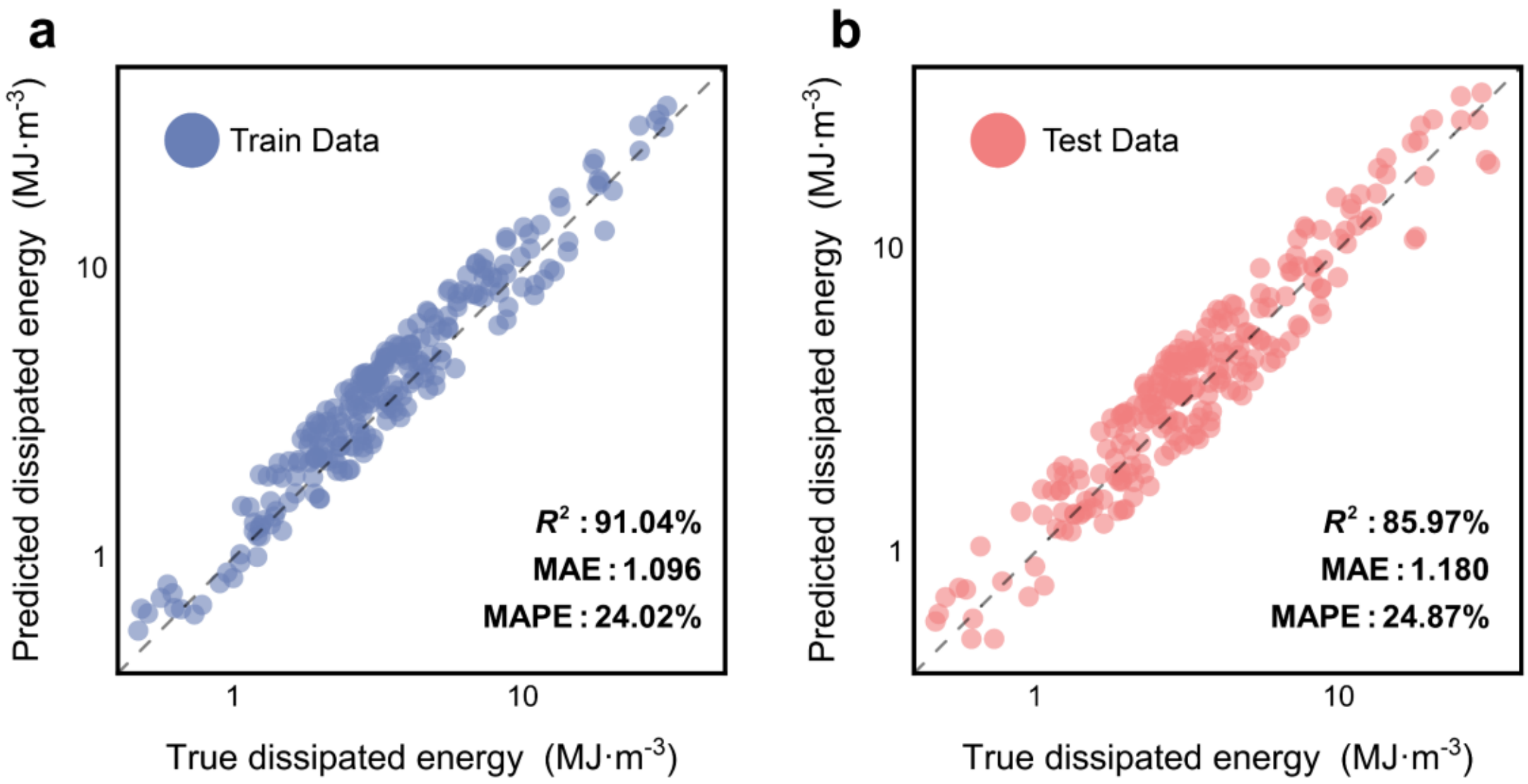

Supplementary Fig. 50 | Predictive performance of dissipated energy. a, b Comparison between predicted and experimental dissipated energy for the (**a**) training and (**b**) test datasets. Both axes are presented on a $\log_{10}$ scale. The dashed line denotes ideal agreement ($y = x$). Model performance is quantified by R^2, MAE, and MAPE, yielding 91.04%, 1.096, and 24.02% for the training set, and 85.97%, 1.180, and 24.87% for the test set, respectively.

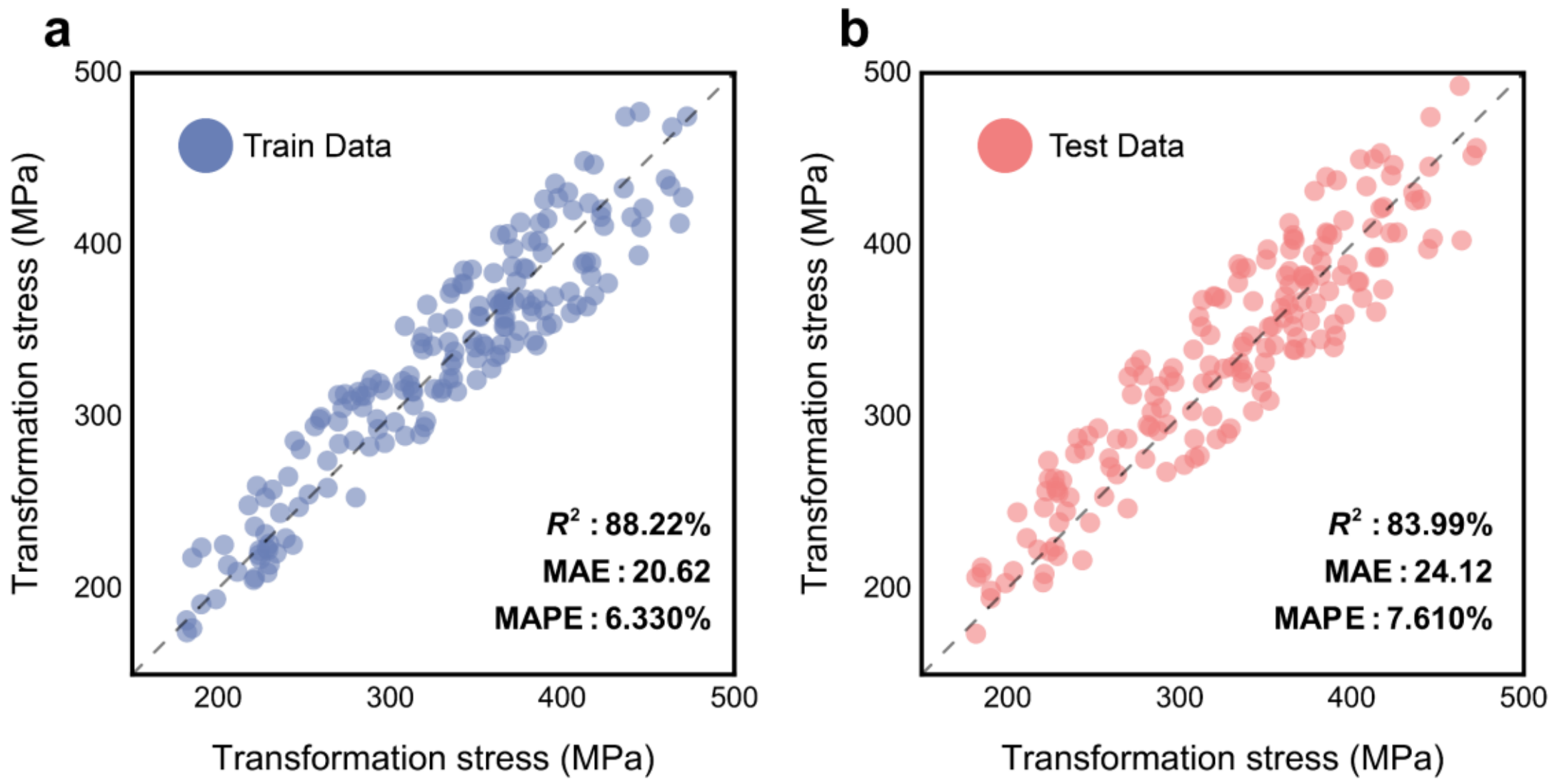

Supplementary Fig. 51 | Predictive performance of forward transformation start stress. a, **b** Comparison between predicted and experimental transformation stress for the (**a**) training and (**b**) test datasets. The dashed line denotes ideal agreement ($y = x$). Model performance is quantified by R^2, MAE, and MAPE, yielding 88.22%, 20.62, and 6.33% for the training set, and 83.99%, 24.12, and 7.61% for the test set, respectively.

Finally, while the interpretability tools link input features to the predicted output, the manuscript does not clearly explain how changes in the latent space correspond to changes in the physical output. It therefore remains unclear how variations in encoded microstructure or other modalities translate quantitatively into predictive residual strain evolution. Explicitly connecting latent feature trends to output sensitivity would further support the claim that the model provides mechanistic insight rather than only accurate predictions. Because the output variable is directly related to well-established degradation mechanisms, there is also a risk that the ML interpretation primarily reflects known correlations rather than uncovering new causal structure. A discussion acknowledging this limitation would help position the contribution more clearly as quantitative and spatially resolved rather than purely confirmatory.

1.13 Finally, while the interpretability tools link input features to the predicted output, the manuscript does not clearly explain how changes in the latent space correspond to changes in the physical output. It therefore remains unclear how variations in encoded microstructure or other modalities translate quantitatively into predictive residual strain evolution. Explicitly connecting latent feature trends to output sensitivity would further support the claim that the model provides mechanistic insight rather than only accurate predictions.

1.13 Response: We sincerely appreciate this important comment. While the original manuscript linked input features to predictions using Grad-CAM and SHAP, it did not sufficiently elucidate how changes in the fused latent space quantitatively relate to the evolution of predictive residual strain. In the revised manuscript, we establish the connection between latent representation, output sensitivity, and performance.

Specifically, latent representations from the fusion layer are tracked throughout training, and two quantitative metrics are introduced. The first is the inter-modality distance, defined as the average cosine distance among the three image modalities, which characterizes the degree of multimodal alignment. The second is the latent–output RDM correlation, defined as the Spearman correlation between the pairwise distance matrix in the latent space and that of the true residual strain, measuring how effectively the latent representation captures variations in the physical output. By jointly analyzing these metrics with the test R^2, we can assess whether improved multimodal alignment yields a latent space that more faithfully reflects the evolution of residual strain.

The results **(Supplementary Fig. 33)** show that, as training progresses, inter-modality distance decreases steadily, while both the latent–output RDM correlation and test R^2 increase in tandem. A strong negative correlation is observed between inter-modality distance and latent–output RDM correlation ($R = -0.94$), alongside a strong positive correlation between latent–output RDM correlation and test R^2 ($R = 0.95$). The optimization of the latent space is not merely a numerical rearrangement, but a physically meaningful reorganization in which multimodal information becomes

progressively aligned and more effectively resolves residual strain differences. **In other words, microstructural and loading features are organized in a manner consistent with output evolution, directly translating into improved predictive performance.**

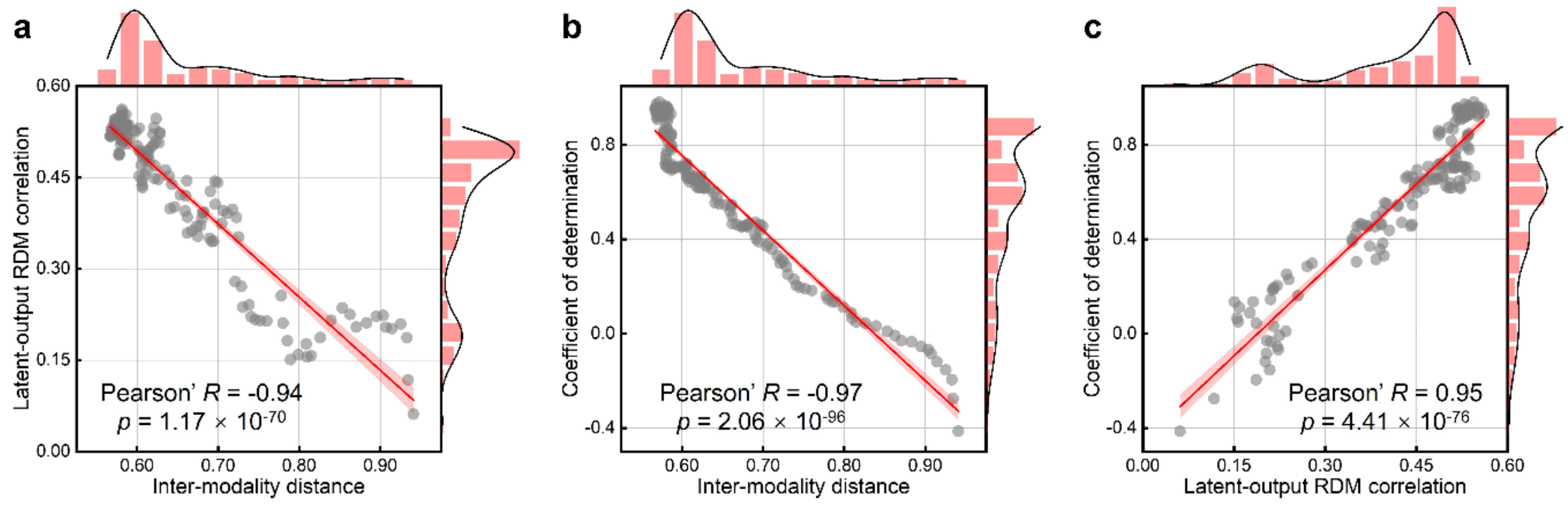

Supplementary Fig. 33 | Quantitative association between multimodal representation evolution and prediction performance. Each point represents a training stage. Metrics are computed using training samples and evaluated on an independent test set. As training progresses, inter-modality distance decreases steadily, accompanied by concurrent increases in latent–output RDM correlation and test R^2. These trends indicate that reduced inter-modal separation leads to a latent space that more faithfully captures residual strain differences, thereby enhancing predictive performance.

To address this issue, **a new section entitled "Multimodal representation and performance" has been added to the Supplementary information**. The specific content is provided below.

Multimodal representation and performance: To quantitatively assess multimodal integration during training, two representational metrics were introduced. The inter-modality distance is defined as the mean pairwise cosine distance (1−cosine similarity) among the three image modalities. In addition, following the Representational Dissimilarity Matrix (RDM) theory (Mehrer et al., 2020), a latent representation distance matrix $D_{ij}^{\text{latent}} = 1 - \cos(r_i, r_j)$ and a corresponding physical output dissimilarity matrix $D_{ij}^{\text{out}} = |y_i - y_j|$ were constructed for all training samples.

The latent–output RDM correlation was then determined by calculating the Spearman correlation coefficient between the upper triangular elements of these two matrices. This metric quantifies the consistency between inter-sample distances in the latent space and the corresponding differences in residual strain:

$$\rho_{\mathrm{RDM}} = \mathrm{SpearmanCorr}(\mathrm{upper}(D^{\mathrm{latent}}), \mathrm{upper}(D^{\mathrm{out}})) \tag{1}$$

Clear associations are observed among the representational and performance metrics: as inter-modality distance diminishes, the latent–output RDM correlation increases (R = −0.94), accompanied by a concurrent rise in test R^2. Furthermore, the latent–output RDM correlation is significantly positively correlated with predictive accuracy (R = 0.95). These findings demonstrate that latent space optimization during training manifests as both the convergence of multimodal features and enhanced consistency between inter-sample distances and true residual strain gradients. In other words, when latent representations better reflect relative differences in residual strain, model prediction accuracy improves accordingly.

1.14 Because the output variable is directly related to well-established degradation mechanisms, there is also a risk that the ML interpretation primarily reflects known correlations rather than uncovering new causal structure. A discussion acknowledging this limitation would help position the contribution more clearly as quantitative and spatially resolved rather than purely confirmatory.

1.14 **Response:** We sincerely appreciate this insightful and constructive comment. The relationship between ML interpretability and physical causality has been reconsidered. The interpretability methods employed are primarily based on statistical correlations between features and targets. More precisely, these methods reflect the high-dimensional conditional correlations, contribution decompositions, and spatial response patterns learned by the model.

Accordingly, the limitations are discussed, and the positioning of this work is more clearly defined. Specifically, for the slip–SIMT competition, the primary contribution lies in formulating it as a spatial landscape that evolves with loading. Regarding the

role of Ni_4Ti_3 precipitates, SHAP analysis provides a uniquely valuable perspective. The result indicates that coarsened precipitates exacerbate SED. This transition from qualitative interpretation to quantitative attribution provides new evidence for the role of precipitates in the degradation process. Meanwhile, these data-driven trends are supported by complementary evidence from TEM statistical analysis, Schmid factor calculations, and molecular dynamics simulations. **The scientific value of this work is demonstrated by revealing the relative contribution weights and spatial evolution patterns of these mechanisms under complex multi-factor coupling conditions.** Such insights are difficult to assess through traditional experimental approaches. As noted above, this issue was considered alongside the limitations of 2D EBSD characterization and the sensitivity to EBSD preprocessing. Three key aspects were addressed in the **Discussion section**:

Revision 1 (Page 22, Lines 17-26): The complexity is manifested in the fact that localized deformation in NiTi precludes meaningful in-situ diffraction characterization throughout a continuous loading sequence (Chowdhury and Sehitoglu, 2017; Paranjape et al., 2017; Racek et al., 2020; Robertson et al., 2012). Against this backdrop, the interpretable ML framework demonstrates a quasi-in-situ mechanistic reconstruction approach, compensating for the lack of true full-field observation through either experimental techniques or theoretical modeling. Unlike molecular dynamics simulations, which hinge on precise potentials and idealized initial configurations(Ko et al., 2015), this framework spontaneously learns patterns from real microstructures and corroborates them with independent findings. Such reconstruction bridges the existing observational gap and lays the foundation for calibrating multiscale theoretical models (Yu et al., 2015; Zadeh et al., 2025).

Revision 2 (Page 23, Lines 6-14): Once this mapping is converted into measurable spatial distributions, we can track the migration of dominant deformation zones across the entire gauge, moving beyond empirical judgments limited to isolated grains or restricted fields of view. The scientific advance arises from transforming previously qualitative, fragmented mechanistic insights into a unified perspective: SED of NiTi

emerges as a competition between mechanisms regulated by loading conditions, with the active pathway selected at the microscale by crystallographic geometric compatibility. This process can now be quantified and predicted in a data-driven manner.

Revision 3 (Page 25, Lines 17-21): Admittedly, the interpretative method identifies and quantifies high-dimensional correlations in multimodal data. When applied to examine qualitative mechanistic hypotheses, it provides spatially resolved, quantitative evidence rather than independently establishing rigorous causal laws, although the findings are strongly corroborated by Schmid factor analysis and TEM observations.

1.15 Some specimens appear to be partially recrystallized, and the rationale behind this microstructural state is not entirely clear.

1.15 Response: Thanks for your valuable reminder. Some annealed samples exhibit partially recrystallized, which warrants further clarification in the revised manuscript. The material used in this study was a cold-rolled NiTi sheet subjected to short-time annealing (10–30 min). The resulting microstructure should be interpreted as a partially recovered and partially recrystallized state. Under such conditions, retained rolling textures, local orientation gradients, and residual internal stresses are expected characteristics.

For cold-worked NiTi alloys, previous studies have shown that a partially recrystallized state may even enhance cyclic stability. For example, Lee et al.[19] reported that Ni-rich NiTi sheets subjected to severe plastic deformation followed by short-time annealing (5 min) developed partially recrystallized microstructures, which exhibited excellent superelasticity and cyclic stability. In contrast, prolonged annealing promoted microstructural coarsening, leading to increased residual strain during cycling. Chen et al.[20] demonstrated that incompletely recrystallized microstructures formed at 600 °C for 30 min facilitate the nucleation of Ni_4Ti_3 precipitates due to the presence of dislocations and subgrain boundaries, thereby

significantly improving functional stability. Li et al.[21] noted that partially recrystallized NiTi maintains stable texture characteristics over multiple cycles, with less degradation of the stress plateau than its nanocrystalline counterparts. **Collectively, the presence of dislocations, substructures, and retained deformation textures under short-time annealing is consistent with the microstructural evolution of NiTi alloys and represents an expected outcome.** Additionally, the representative annealing regimes selected in this study were not arbitrary, but were identified through preliminary screening of a broader parameter space. Specifically, a series of annealing conditions involving higher temperatures and longer holding times (including solution and aging treatments, totaling ~32 conditions) were systematically investigated, and their mechanical and functional responses (e.g., tensile strength and cyclic stability) were comprehensively evaluated (see Fig. R6). Although fully recrystallized microstructures exhibit greater uniformity and fewer defects, they often display pronounced SED during cyclic loading and, in some cases, even lose stable superelasticity entirely. Therefore, they were not included as representative results here. And the selected regimes represent characteristic microstructural states with distinct precipitate configurations and matrix features.

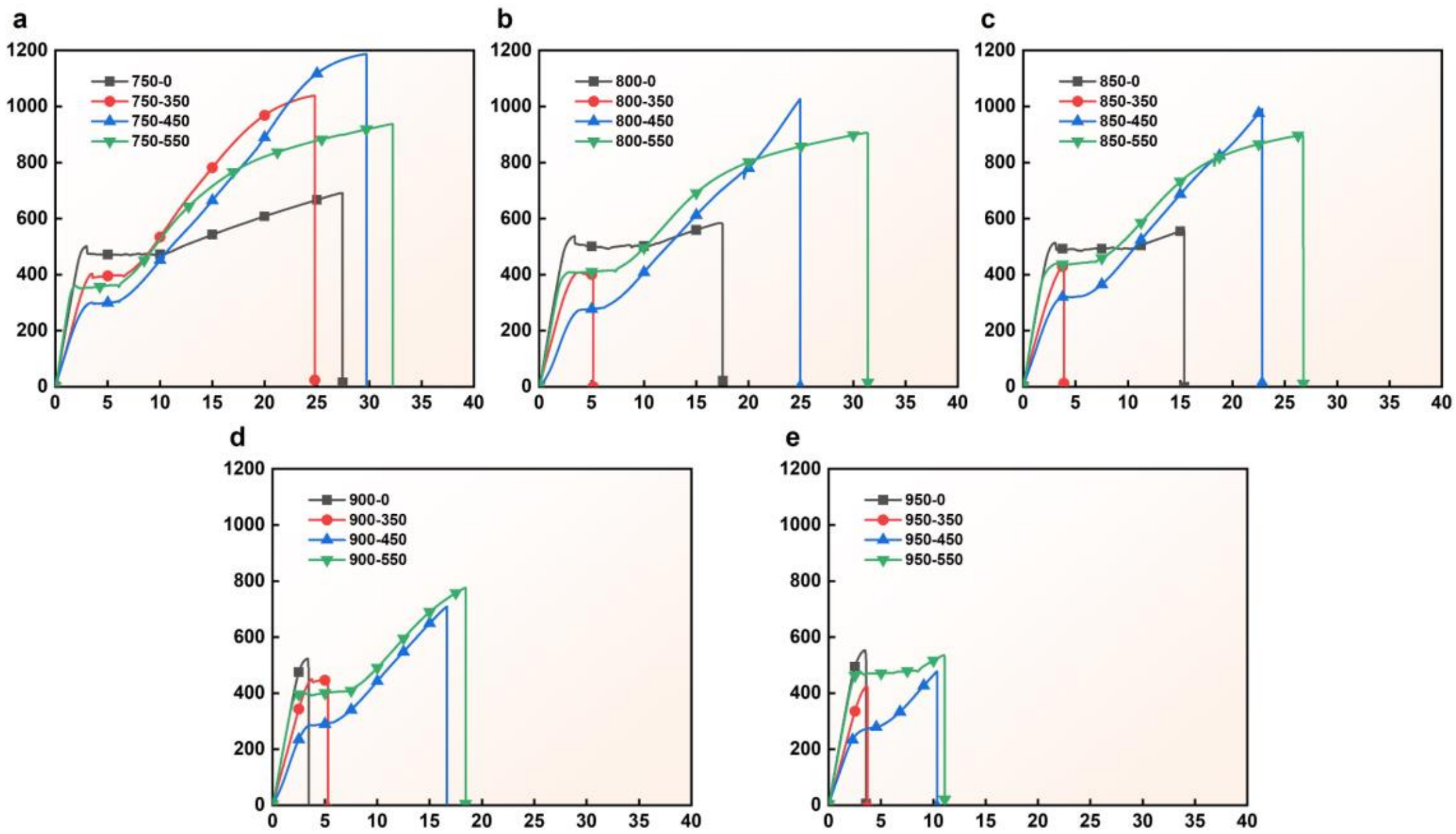

Fig. R6 | Mechanical property under different solution-aging treatment conditions. Engineering stress–strain curves (strain, %; stress, MPa) are presented.

Here, "750–350" denotes solution treatment at 750 °C for 1 h followed by aging at 350 °C for 30 min, whereas "0" indicates annealing without subsequent aging. All specimens were subjected to three parallel tests to ensure reproducibility.

Corresponding clarifications are provided in the **Supplementary information**:

(1) **In the caption of Supplementary Fig. 15**, the selection criteria for the annealing regimes have been clarified.

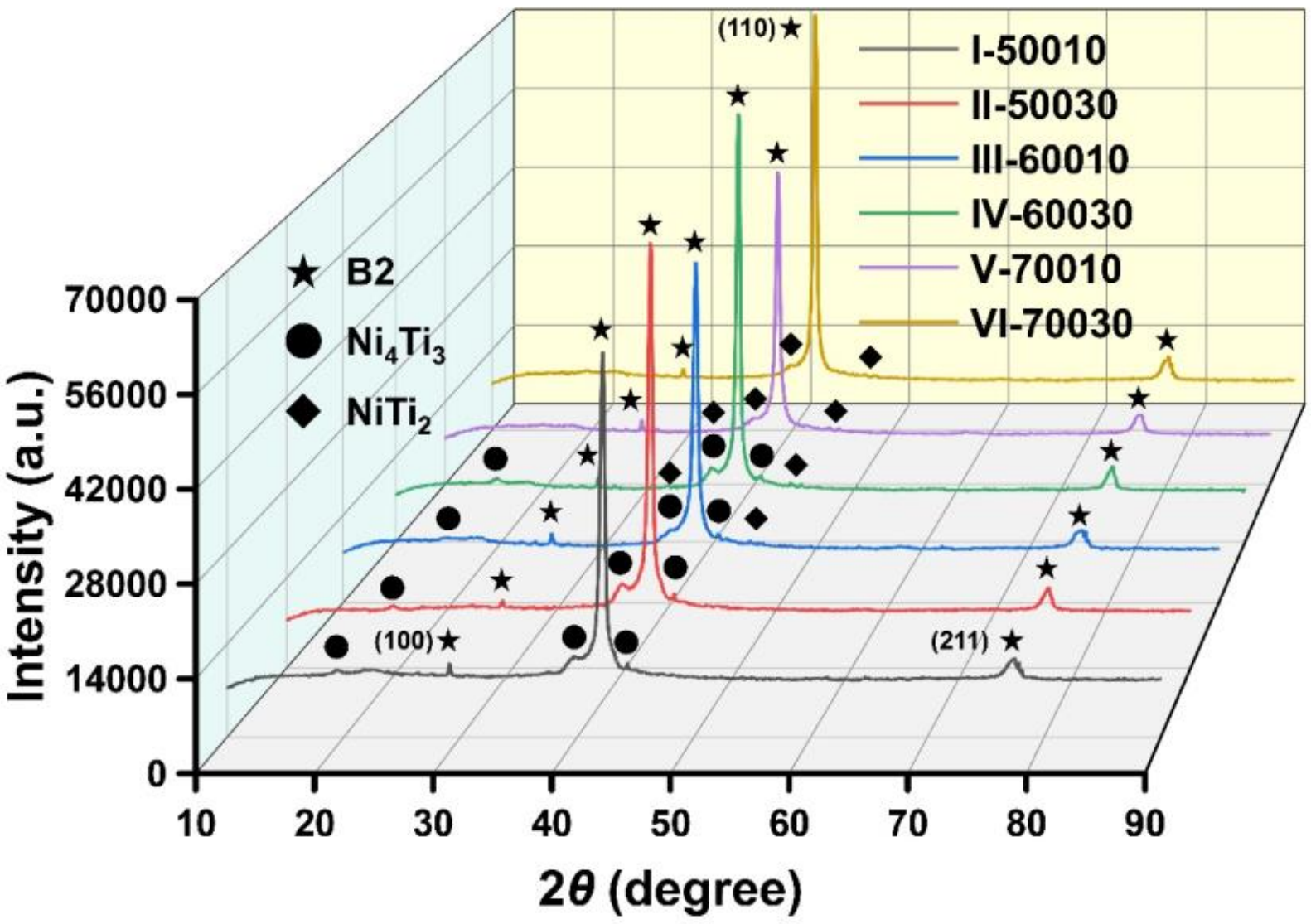

Supplementary Fig. 15 | XRD patterns of NiTi alloys annealed under various short-term annealing conditions. Specimens I–VI correspond to distinct annealing regimes: I–50010 (500 °C, 10 min), II–50030 (500 °C, 30 min), III–60010 (600 °C, 10 min), IV–60030 (600 °C, 30 min), V–70010 (700 °C, 10 min), and VI–70030 (700 °C, 30 min). These six conditions were selected after a preliminary screening of a broader range of annealing parameters, considering their overall mechanical and functional responses (e.g., tensile strength and cyclic stability). They represent distinct microstructural states involving variations in precipitate characteristics, while all specimens retain a B2 austenitic matrix at room temperature. With increasing annealing temperature, fine Ni_4Ti_3 precipitates dominate at 500 °C; a multiphase microstructure containing both Ni_4Ti_3 and $NiTi_2$ is observed at 600 °C, whereas $NiTi_2$ becomes the prevailing phase at 700 °C. Prolonging the holding time mainly promotes the coarsening and redistribution of precipitates without altering their types.

(2) **In the Electron backscatter diffraction section (Supplementary Fig. 17)**, the roles of residual dislocations and substructures in matrix strengthening are clarified with additional references. The relevant content is provided below.

Electron backscatter diffraction: The EBSD results from Specimen IV are presented as a representative example. The microstructure exhibits a multiphase configuration comprising the B2 matrix (yellow) together with uniformly dispersed Ni_4Ti_3 (red) and $NiTi_2$ (blue) precipitates. Due to cold-rolling deformation prior to annealing, residual internal stresses were not fully relieved, resulting in a pronounced {101} texture. This microstructural state, characterized by a certain density of residual dislocations and refined grain structures, is instrumental in strengthening the matrix against plastic slip during stress-induced martensitic transformation, thereby enhancing superelastic stability (Delville et al., 2011; Li et al., 2025). Nevertheless, the overall structure remains predominantly uniform, with equiaxed grains averaging approximately 10.3 μm. In the phase image, $NiTi_2$ precipitates are generally identifiable, while Ni_4Ti_3 can only be effectively resolved by EBSD when sufficiently large (>200 nm). EBSD struggles to fully analyze smaller Ni_4Ti_3 precipitates. This is why we incorporated TEM results into the numerical feature to achieve a more comprehensive microstructural characterization.

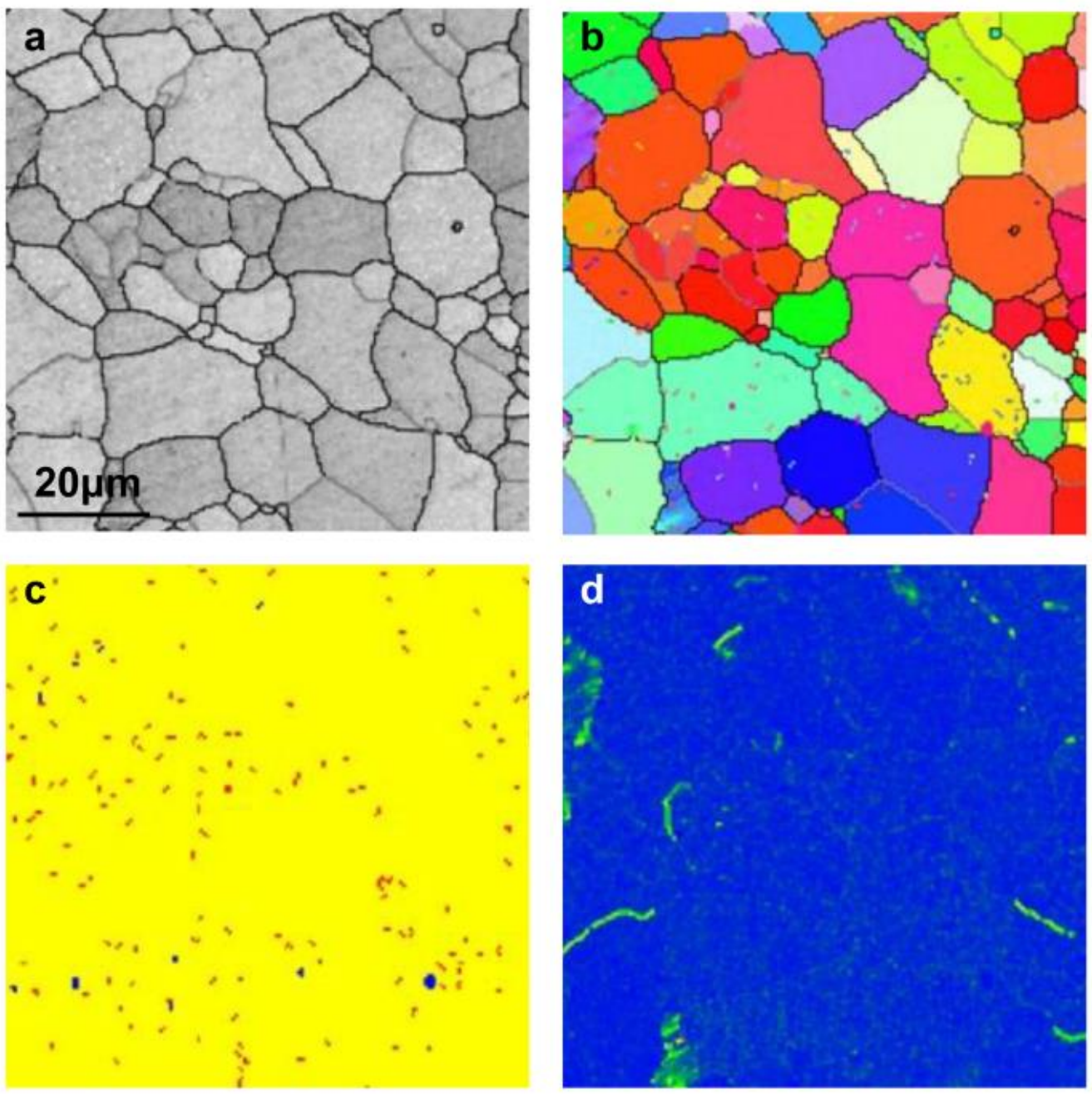

Supplementary Fig. 17 | EBSD characterization of specimen IV annealed at 600 °C for 30 min. a Band contrast image. **b** Inverse pole figure (IPF) map showing an equiaxed grain structure. **c** Phase map indicating that the matrix is primarily B2 austenite. **d** GND map revealing the spatial distribution of geometrically necessary dislocation density and local lattice curvature.

1.16 In addition to microstructural maps, it may be beneficial to incorporate macroscopic mechanical descriptors, such as yield strength, as additional input modalities. These parameters are known to influence cyclic response and degradation behavior and may not be fully captured by microstructural maps alone, potentially improving predictive robustness.

1.16 Response: We sincerely thank you for this insightful suggestion. Beyond microstructural characterization, introducing macroscale mechanical descriptors such as yield strength and plateau stress holds promise for providing additional information to the model, thereby enhancing its capacity to characterize cyclic response and degradation behavior. Such parameters reflect slip resistance, transformation driving force, and thermo-mechanical coupling, and thus possess clear physical significance for engineering predictions.

As noted in our response to Reviewer 2, we initially considered introducing internal variables $z_c = \int_0^t |\dot{z}(s)|\, ds$ to characterize the accumulated volume fraction of residual martensite, along with derived variables and macroscopic descriptors to capture history effects and mechanical response. However, after careful consideration, this approach was not adopted. Instead, the model inputs were deliberately restricted to the initial microstructure and loading conditions. This study aims to examine whether the model can directly learn SED laws from raw data, thereby avoiding the need to include variables derived from macroscopic responses. Furthermore, macroscopic mechanical parameters are not independent sources of information, as they are closely related to microstructural factors, including precipitate characteristics, grain size, and initial internal stresses. **Accordingly, we prefer to construct the model with minimal prior assumptions, enabling it to capture intrinsic relationships among microstructure, loading conditions, and material properties.** In the revised manuscript, ablation experiments were conducted to evaluate the effect of incorporating macroscopic descriptors **(Supplementary Fig. 52)**. Conventional yield strength and forward transformation onset stress were introduced as extra scalar inputs into the numerical branch. Specifically, conventional yield strength was obtained from uniaxial tensile tests at 200 °C, where stress-induced transformation does not occur and aging effects are negligible. The forward transformation onset stress was extracted from cyclic tensile loading–unloading curves, while yield strength was determined from the first loading. Introducing these macroscopic descriptors improves predictive robustness to a limited extent, with a stronger contribution from the forward transformation onset stress. This demonstrates that specimen-scale mechanical parameters effectively supplement microstructural features.

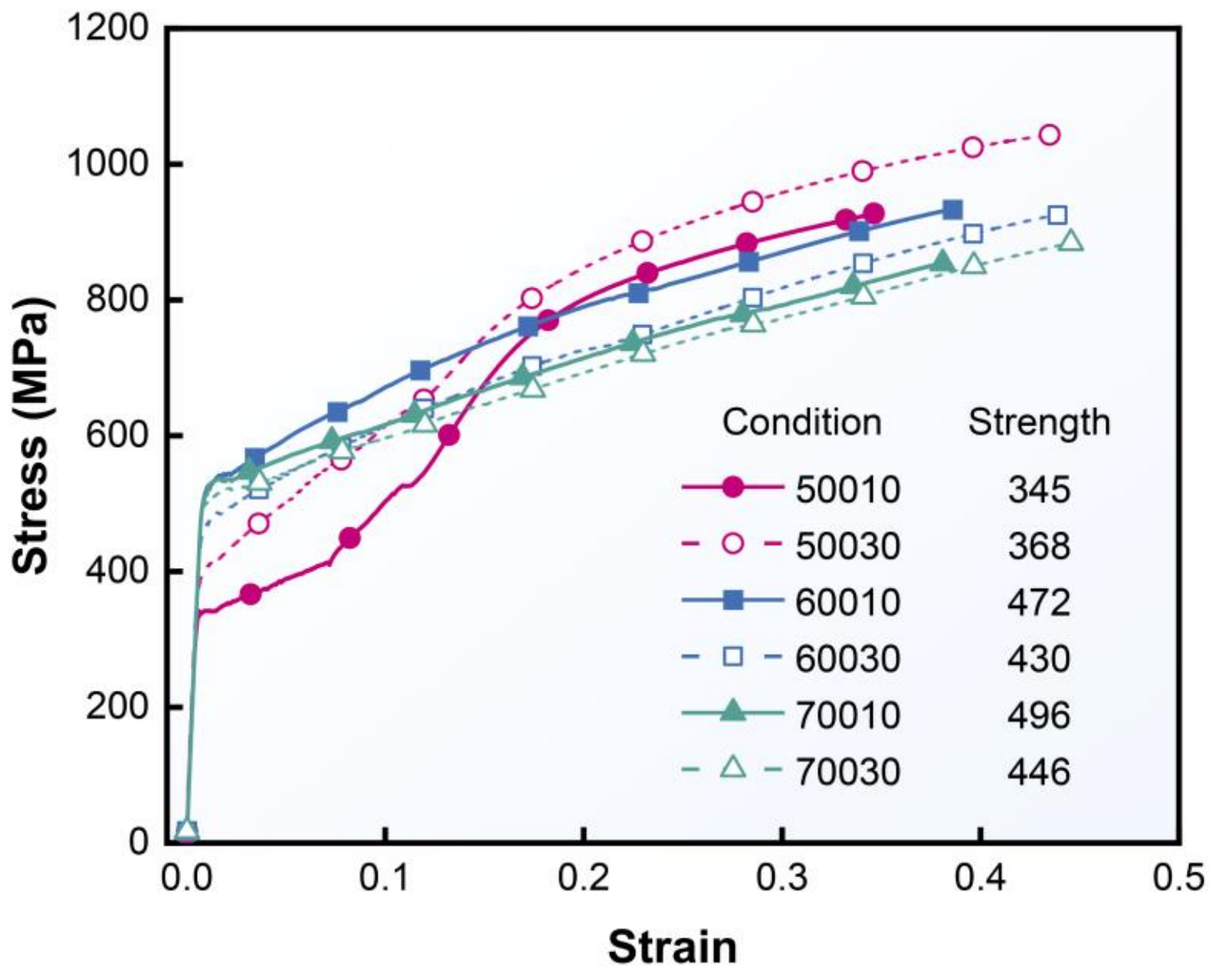

Supplementary Fig. 22 | Tensile testing at 200 °C is used to determine the critical stress $\sigma_{\text{app,slip}}$ for slip (conventional yield strength). At this temperature, no stress-induced transformation occurs and aging effects are still negligible.

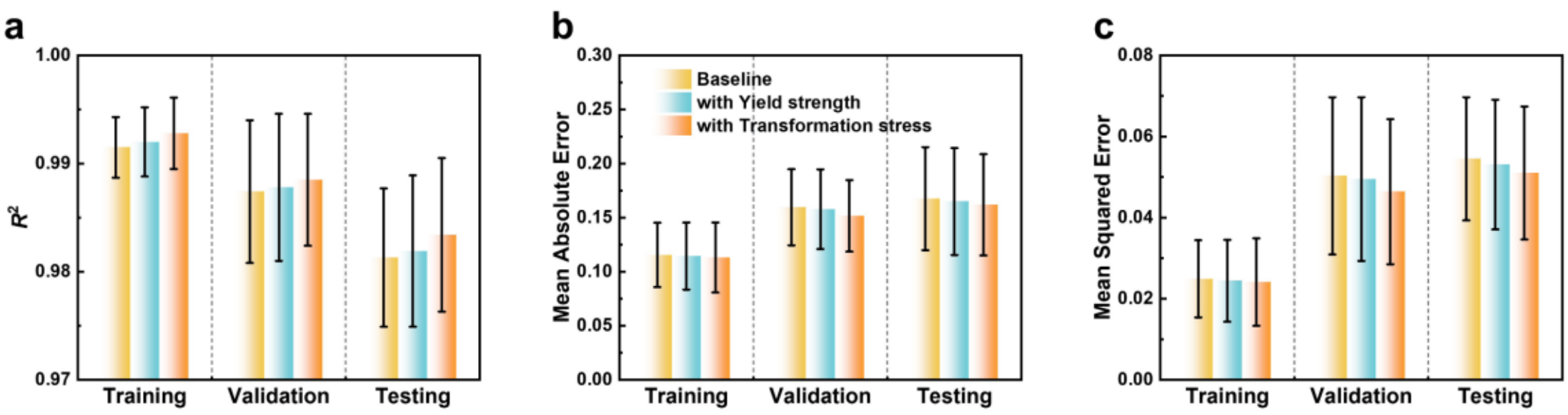

Supplementary Fig. 52 | Ablation study with additional mechanical descriptors. **a-c** Predictive performance metrics (R^2, MAE, and MSE) for models integrated with either conventional yield stress or forward transformation start stress. Results are obtained by incorporating either the conventional yield stress (determined at 200 °C from the critical stress for austenite slip) or the transformation start stress as additional scalar inputs. The transformation stress is extracted from cyclic tensile loading–unloading curves, while the conventional yield stress is defined from the first loading cycle. Both descriptors facilitate incremental improvements in performance, with the transformation start stress providing a slightly greater benefit.

Nonetheless, given the scientific objectives and model interpretability of this study, we have retained the main model with inputs primarily focused on the initial microstructure and loading conditions, treating the aforementioned results as validation of its extensibility. Furthermore, the constructed architecture is inherently open to new modalities, consistent with the grain size extension results presented in the manuscript **(Fig. 7f-i)**. **Looking ahead, integrating macroscopic descriptors with temporally microstructural information during deformation represents a promising direction. Such integration may enable the transition from outcome prediction to a temporal modeling framework for the full-field evolution of microstructure–deformation.**

Thank you again for your valuable suggestion. Relevant clarifications and ablation results are incorporated into the revised manuscript and Supplementary information.

1.17 Because the model relies heavily on EBSD-derived maps, a brief discussion of sensitivity to EBSD preprocessing steps (e.g., denoising, step size) would strengthen confidence in the robustness of the conclusions.

1.17 **Response:** We sincerely appreciate this important and constructive comment. The EBSD step size determines the spatial sampling resolution and may influence the quantification of local orientation gradients. In addition, denoising procedures can affect the preservation of subtle orientation variations and local microstructural features. Following this suggestion, we have supplemented the **Discussion section** with relevant limitations, noting that the current analysis is primarily based on 2D EBSD data and that denoising intensity and step size can affect hotspot localization and the characterization of subtle orientation gradients. Meanwhile, the EBSD acquisition and post-processing workflow is clarified in the **Methods section**. Specifically, all Euler, phase, and GND maps used for modeling were acquired under consistent EBSD conditions and processed with a unified preprocessing pipeline to minimize preprocessing-induced variations. It is further acknowledged that systematic sensitivity analyses regarding step sizes and denoising intensities remain to be

explored in future work. This issue relates to 2D characterization (Comment 10) and interpretable ML (Comment 14) and is therefore addressed in a unified manner.

The revision in the **Discussion section (Page 25, Lines 26-28 and Page 26, Lines 1-8)** is as follows:

In this context, EBSD preprocessing (e.g., denoising intensity and step size) directly influences the precision of hotspot localization and may even obscure subtle orientation gradients. While the identified physical trends are unlikely to be attributable to preprocessing variations and are supported by multiscale experiments and simulations, systematic evaluation of model sensitivity remains essential for further optimization. Future research should integrate 3D EBSD, in situ diffraction, and X-ray tomography to bridge the gap between planar characterization and bulk deformation behavior. Augmented with 3D structural information and enhanced robustness to noise, the framework will occupy a superior vantage point for deconstructing the origin of functional fatigue in SMAs.

The revision to the **EBSD experiment section (Page 32, Lines 11-19)** is as follows:

All EBSD data underwent a standardized preprocessing workflow prior to image export, including correction of isolated noise and unindexed pixels, as well as consistent smoothing and denoising. This procedure minimized the impact of preprocessing while avoiding excessive reconstruction or over-smoothing that could obscure local microstructural features. The original images were subsequently cropped into six sub-images of 336 × 336 pixels, a size that balances grain-morphology integrity with local structural detail. Data augmentation was applied to these cropped images using offline operations such as rotation and mirroring (Supplementary Fig. 30).

Finally, we are very grateful for your valuable comments and the time given. We have carefully revised and polished the article to meet the high standards of the journal. Once again, we express our gratitude for your patience and professionalism, and sincerely convey our respect and admiration here.

Reviewer 2: This study addresses the critical issue of superelastic degradation (SED) in NiTi alloys by constructing an interpretable multimodal learning framework that integrates innovation, rigor, and practicality. The research methodology combines multimodal data, interpretable machine learning, experiments, and simulations, successfully revealing the competitive mechanism between dislocation slip and stress-induced martensitic transformation (SIMT) as well as the dual role of precipitates. The conclusions are reliable and hold significant engineering value. This work reveals the mapping relationship between the featured microstructures at the microscopic scale and superelastic degradation behavior at the macroscopic scale, and is important for practical applications. Thus, I recommend this paper to be published after the following comments are addressed by the authors.

2.0 **Response:** Thank you very much for your review and effort. We really appreciate it. The questions raised include both specific clarifications and broader technical suggestions. Based on these questions and suggestions, we have made significant revisions to further improve the overall quality of the manuscript. Below, we will address each point individually.

2.1 In page 16, is there a labeling error for Fig. 5f?

2.1 **Response:** We sincerely appreciate this careful observation and apologize for the labeling error in the original manuscript (the correct reference is Fig. 5e, not Fig. 5f). **The sentence (Page 17, Lines 5–7)** has been corrected as follows: Statistical analysis of 186 grains reveals that grains undergoing variant transfer exhibit significantly higher m′ with the original hotspot grain than neighboring grains with the highest Schmid factor (Fig. 5e). In addition, all figures and their corresponding labels have been carefully checked throughout the manuscript to avoid similar issues.

2.2 Five metrics are used in Fig. 2j to evaluate the effectiveness of input combinations. As the authors mentioned, comparing the Euler input with Euler+Phase dual inputs, the Euler is superior in some metrics while the Euler+Phase input performs better in others. Then how is the conclusion that 'Euler+Phase < Euler' drawn?

2.2 Response: Thank you very much for your valuable reminder. We agree that the simplified expression "Euler + Phase < Euler" in the original manuscript was imprecise. A clear trade-off exists between the Euler input and Euler + phase input across the five evaluation metrics. Specifically, the Euler input yields lower MAE and MAPE, whereas the Euler + phase combination achieves better performance on R^2, RMSE, and MSE. Consequently, the inequality is not statistically supported and has been revised to ensure a more rigorous presentation.

Our intention was to highlight that increasing input channels (or modalities) does not necessarily yield consistent performance gains across all metrics. Instead, it can introduce redundant or noisy information. Accordingly, under multi-source data conditions, effective feature decoupling and integration are essential. To address this issue, we referred to several studies published in Nature Communications (Nat. Commun., 2021, 12, 3394; Nat. Commun., 2024, 15, 4853; Nat. Commun., 2024, 15, 8625; Nat. Commun., 2024, 15, 9662; Nat. Commun., 2025, 16, 9195)[22-26], where trade-offs across evaluation metrics are commonly presented using multi-metric tables, embedded tables within figures, or heatmaps. Therefore, **Fig. 2j (now is Fig. 2k in the revised manuscript) and its caption** have been revised. The oversimplified expression "Euler + Phase < Euler" was removed, and an embedded table presents the MAE and MAPE results, as shown below.

[Figure Redacted]

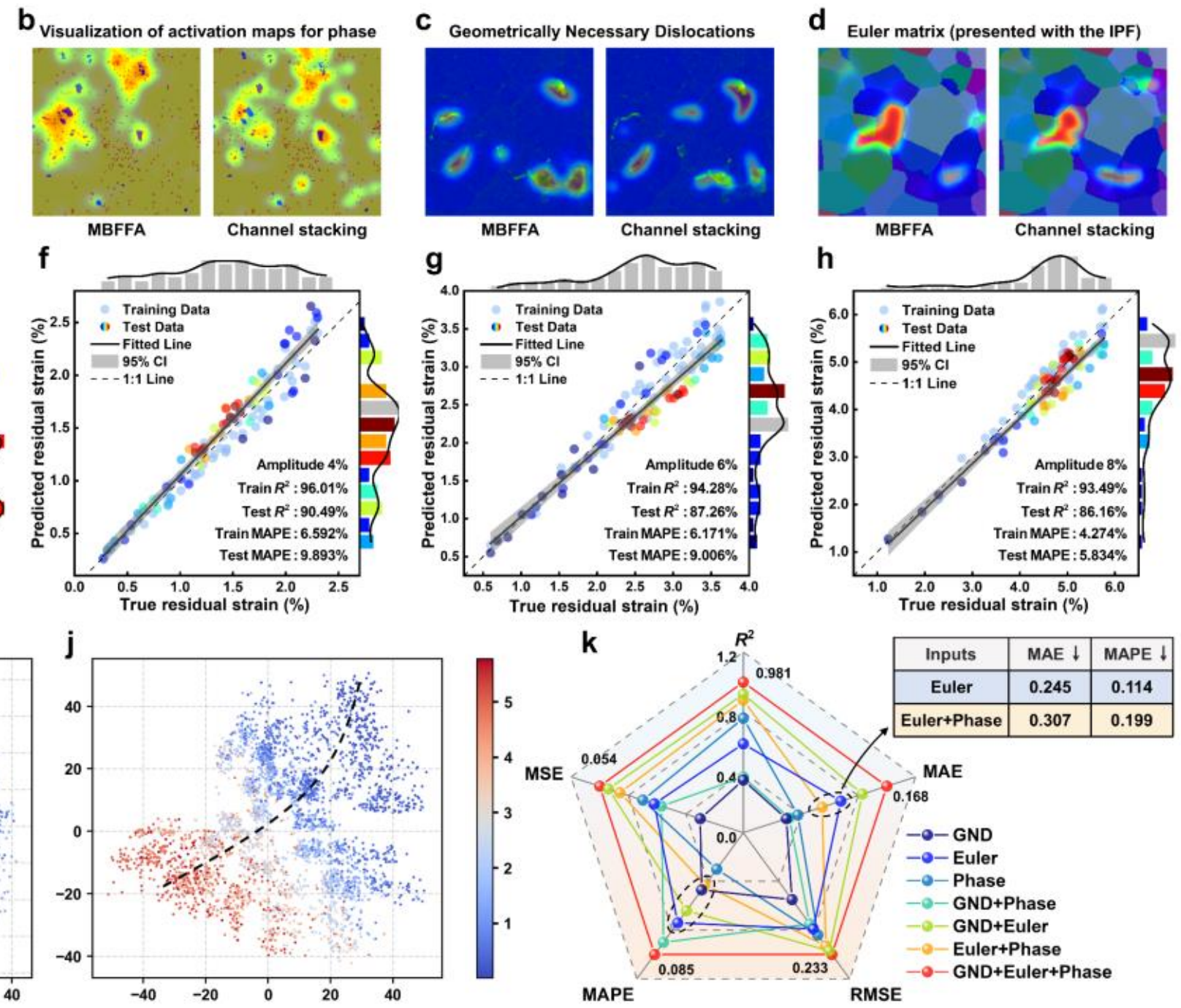

Fig. 2 | Structural evaluation and performance of MBFFA. a The design draws inspiration from the human visual system, where two streams separately process recognition and localization, enabling complementary information processing. To compare feature extraction strategies, the channel stacking approach (Ren et al., 2023) and MBFFA were visualized using Grad-CAM on the phase images (**b**), GND maps (**c**), and Euler matrices (**d**). The MBFFA focuses more precisely on physically meaningful regions, including phase boundaries, areas of concentrated lattice bending, and orientation discontinuities. **e–h** Across strain amplitudes, the predictions consistently align with experimental trends. **i, j** UMAP visualizations of high-dimensional features before and after the feature synergy module, colored by the true residual strain. After fusion, the latent space exhibits a more orderly structure with distinct continuous strain gradients. **k Multi-source inputs demonstrate advantages, but the improvements are not consistent across metrics, highlighting the need for careful feature fusion design**.

Furthermore, **the corresponding description in the manuscript (Page 9, Lines 12–18)** has been updated: Nevertheless, combined inputs did not consistently outperform a single source, regardless of information type. For example, compared with the Euler

matrix alone, adding the phase input increased MAE (from 0.245 to 0.307) and MAPE (from 0.114 to 0.199), suggesting that an additional channel can introduce redundancy or noise. The result underscores the necessity of rational feature decoupling and integration under multi-source data scenarios.

2.3 In fact, superelastic degradation not only depends on the magnitude of the external load but also significantly relies on the loading rate (Int. J. Plasticity, 2015, 72, 60-90). This is an important issue in engineering applications. Is it possible to further incorporate this factor into the framework established in this paper?

2.3 **Response:** We sincerely appreciate this important and insightful comment. Superelastic degradation is governed not only by the magnitude of applied loading, but also by thermo-mechanical conditions such as strain rate and temperature. This issue is of significant engineering relevance, motivating an emphasis on extensibility.

The current dataset was constructed at room temperature and under quasi-static strain-rate conditions. Nevertheless, the proposed framework exhibits considerable extensibility. Unlike conventional multimodal approaches, such as channel stacking or early fusion, which combine heterogeneous information at the input stage and may introduce semantic interference and inconsistency[27], **the present framework emphasizes decoupled representation and synergistic integration of distinct modal features.** Specifically, spatially distributed information is extracted through convolutional branches, while scalar external variables are incorporated via independent numerical branches, followed by alignment and mergence within a synergy module. This enables newly introduced variables to be seamlessly integrated into the corresponding branches without altering the existing model structure. Consequently, external factors such as strain rate and temperature can be naturally incorporated into the framework.

To validate this, additional strain-rate experiments were conducted under cyclic tensile loading–unloading at 1×10^{-3} s^{-1}, 1×10^{-2} s^{-1}, and 3×10^{-2} s^{-1} **(Supplementary Figs. 2-4 and 7-9 are presented below)**. The result **(Supplementary Fig. 48)** shows

that after incorporating strain rate, the model captures the evolution of residual strain, demonstrating robust adaptability to variations in loading rate.

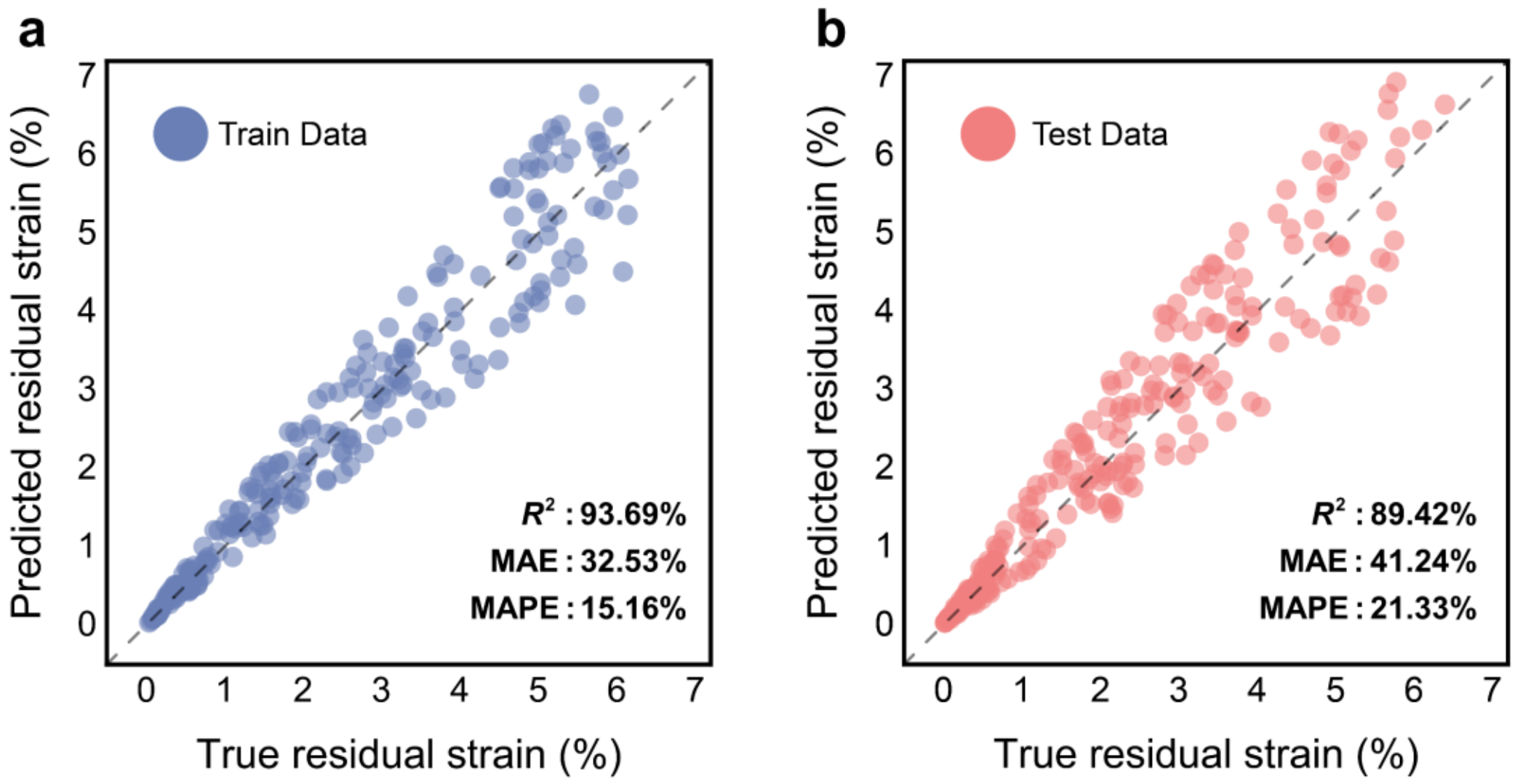

Supplementary Fig. 48 | Predictive performance of residual strain across varying strain rates. a, **b** Comparison between predicted and experimental residual strain for the (**a**) training and (**b**) test datasets at room temperature. Predicted residual strain is plotted against experimental values for cyclic tensile loading–unloading tests performed at four strain rates (5×10^{-4} s^{-1}, 1×10^{-3} s^{-1}, 1×10^{-2} s^{-1}, and 3×10^{-2} s^{-1}). The dashed line denotes ideal agreement ($y = x$). Model performance is quantified by R^2, MAE, and MAPE, yielding 93.69%, 32.53%, and 15.16% for the training set, and 89.42%, 41.24%, and 21.33% for the test set, respectively.

To further evaluate extensibility, additional experiments were conducted at different ambient temperatures for two annealing regimes (500 °C/10 min and 500 °C/30 min) **(Supplementary Fig. 5)**. The results **(Supplementary Fig. 49)** indicate that the model reliably captures degradation trends, supporting its generalizability with respect to thermo-mechanical variables.

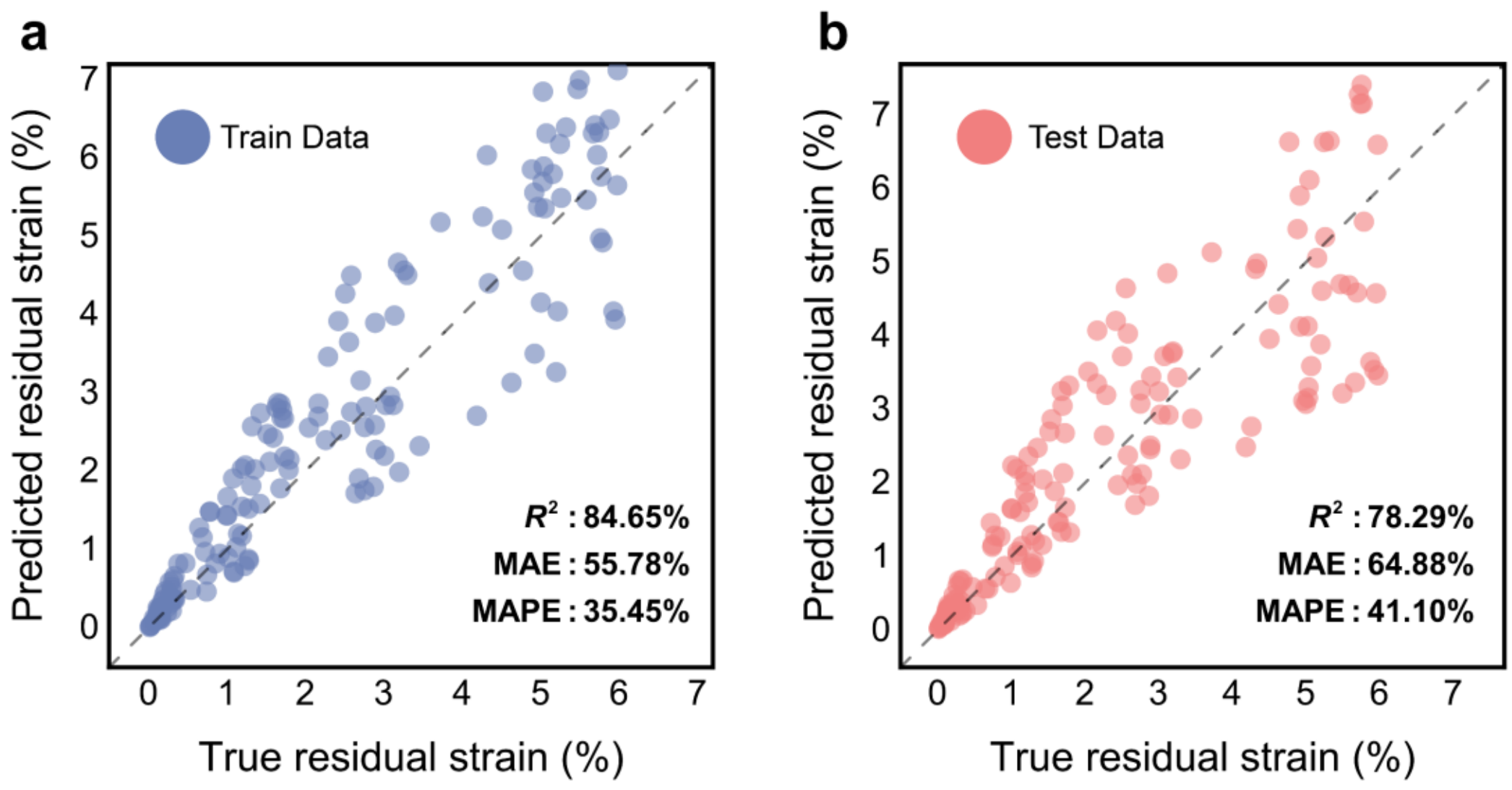

Supplementary Fig. 49 | Predictive performance of residual strain under different temperature conditions. a, **b** Comparison between predicted and experimental residual strain for the (**a**) training and (**b**) test datasets. The results are obtained from cyclic tensile loading–unloading tests conducted at room temperature and 40 °C under a quasi-static strain rate (5×10^{-4} s^{-1}). The dashed line denotes ideal agreement ($y = x$). Model performance is quantified by R^2, MAE, and MAPE, yielding 84.65%, 55.78%, and 35.45% for the training set, and 78.29%, 64.88%, and 41.10% for the test set, respectively.

Although the above experiments demonstrate that the framework can accommodate such external variables, these variables are not incorporated as core inputs into the revised main model. **This is because the primary objective is to establish interpretable relationships between microstructural features, loading conditions and superelastic degradation, and the current model already embodies the core architecture and modeling philosophy of the framework.** Accordingly, the paper focuses on mechanistic analyses of crystallographic orientation and precipitates, supported by experimental characterization. For macroscopic variables such as strain rate and temperature, corresponding analyses of deformation mechanisms are not yet established. Without such support, incorporating them into equivalent-level fusion modeling may introduce inconsistencies between inputs and physical interpretation. In

this revision, these variables are treated as validation inputs to demonstrate extensibility. The Discussion further notes that coupling macroscopic environmental factors represents an important direction toward full-condition predictive modeling.

In the **Discussion section (Page 24, Lines 25–28 and Page 25, Lines 1–2)**, extensibility to temperature and strain rate is further elaborated: Although thermo-mechanical coupling is not systematically investigated here, the present approach enables seamless integration of external factors such as temperature and strain rate (Supplementary Figs. 48, 49) to address more demanding scenarios (Song et al., 2023). By establishing a unified coordinate system for microstructure, loading, and properties, this work formulates a generalizable protocol for design and analysis rather than a model restricted to a specific dataset.

Meanwhile, the **Supplementary information (Supplementary Figs. 2-5 and 7-9)** includes stress–strain curves under varying strain rates and temperatures, residual strain statistics, and corresponding performance evaluations.

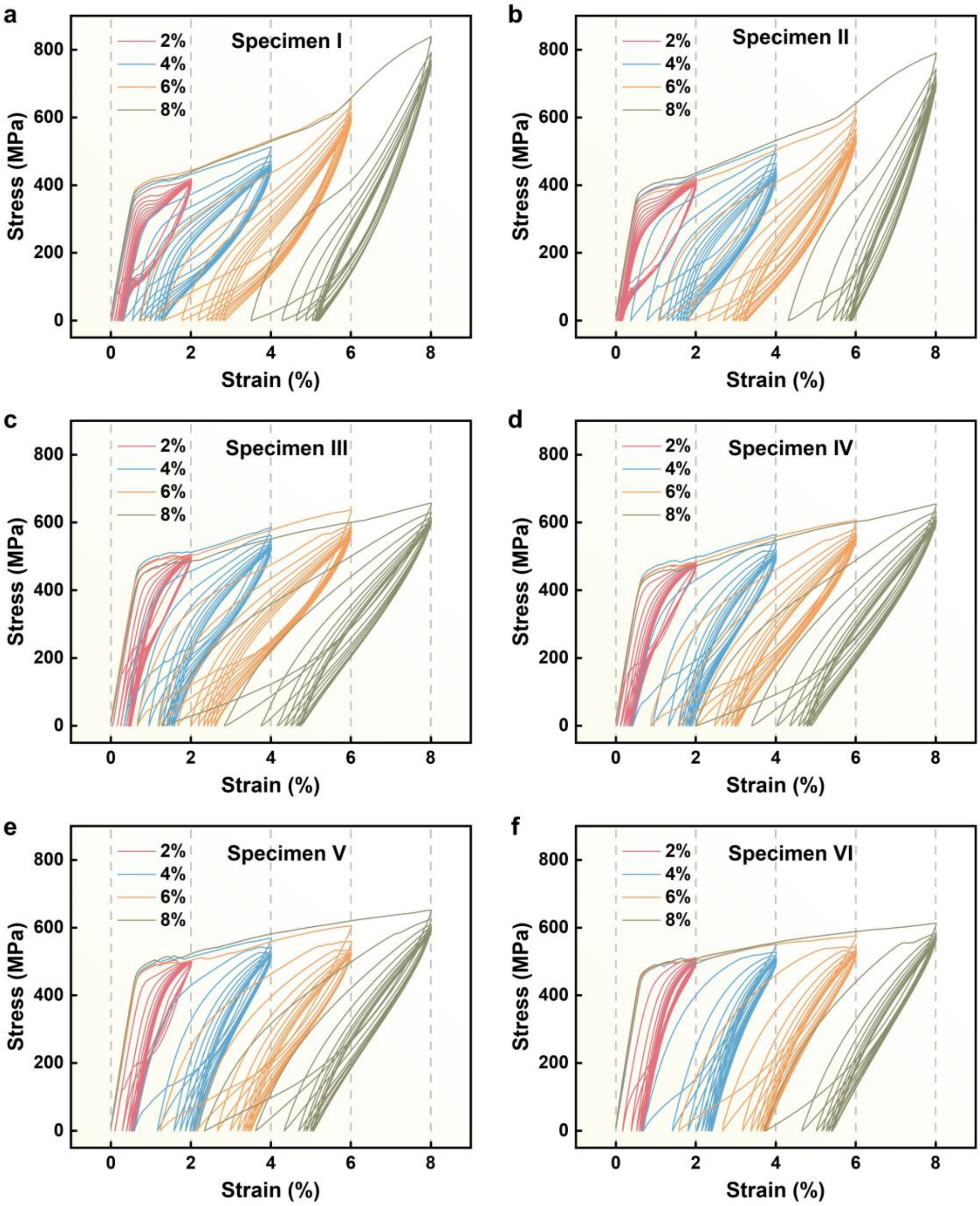

Supplementary Fig. 2 | Cyclic tensile loading–unloading engineering stress–strain curves of NiTi alloys at a strain rate of 10^{-3} s^{-1}. (**a**–**f**) correspond to specimens I–VI, respectively, showing the cyclic test results under strain amplitudes of 2%, 4%, 6%, and 8%.

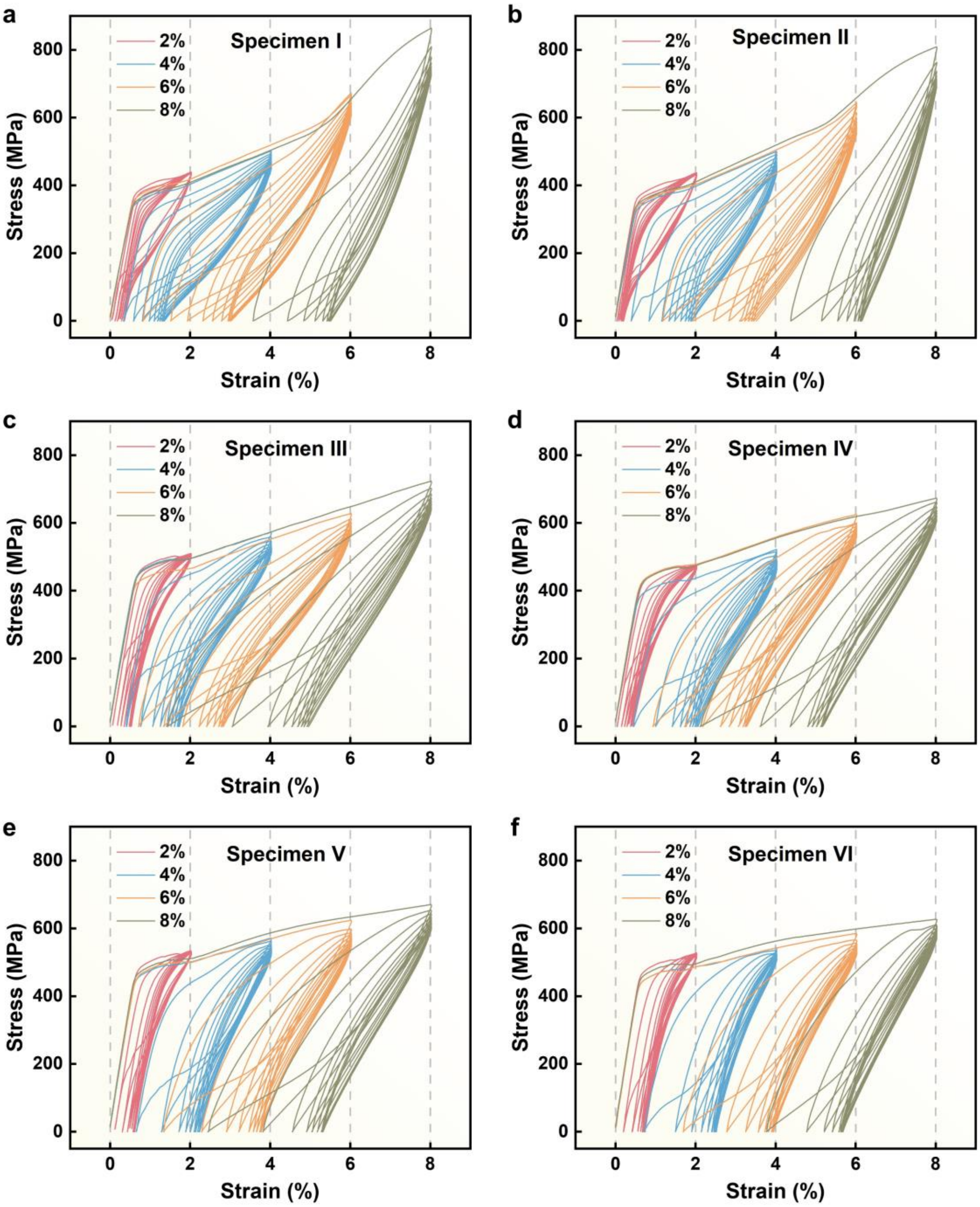

Supplementary Fig. 3 | Cyclic tensile loading–unloading engineering stress–strain curves of NiTi alloys at a strain rate of 10^{-2} s^{-1}. (**a**–**f**) correspond to specimens I–VI, respectively, showing the cyclic test results under strain amplitudes of 2%, 4%, 6%, and 8%.

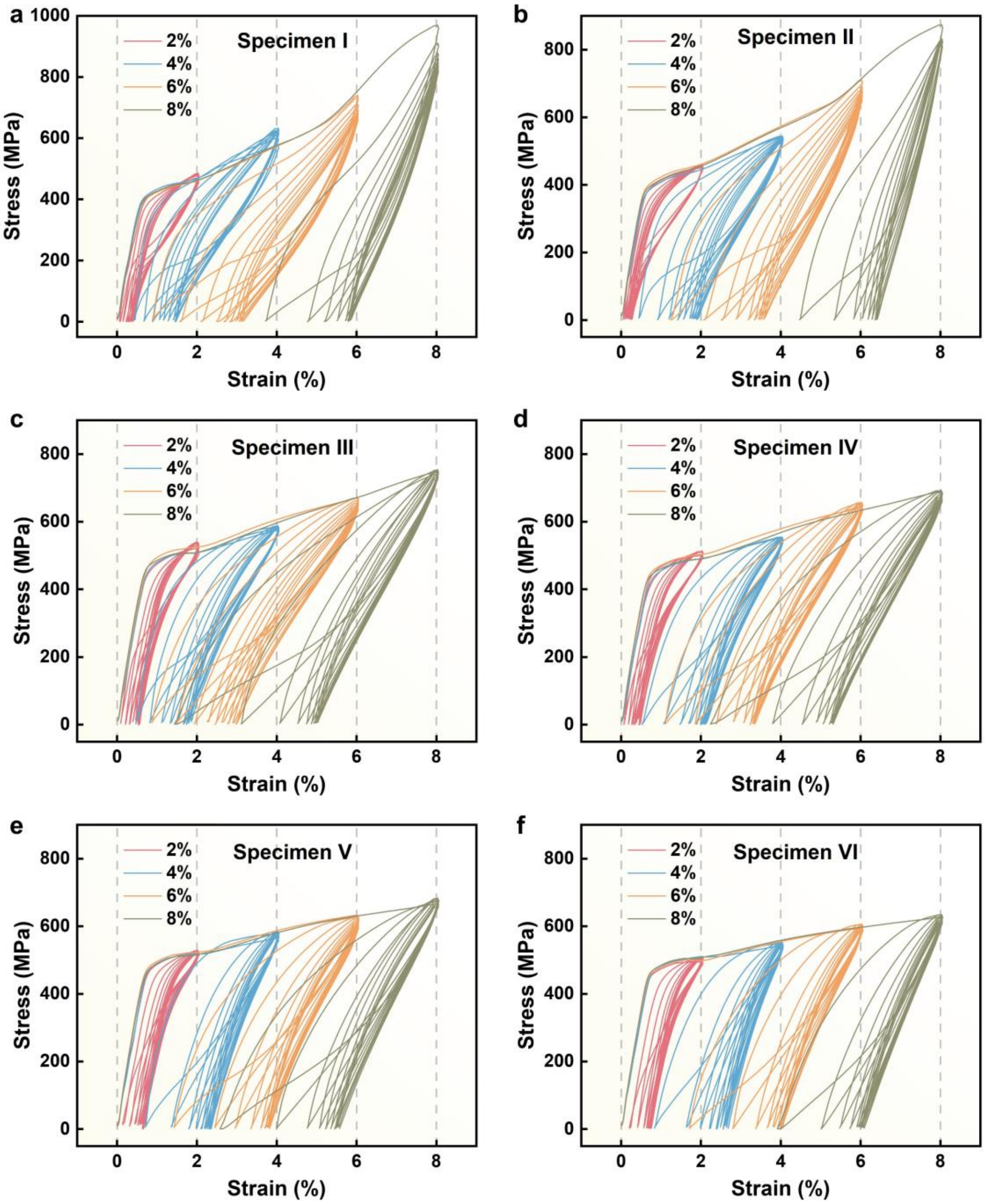

Supplementary Fig. 4 | Cyclic tensile loading–unloading engineering stress–strain curves of NiTi alloys at a strain rate of 3×10^{-2} s^{-1}. (**a**–**f**) correspond to specimens I–VI, respectively, showing the cyclic test results under strain amplitudes of 2%, 4%, 6%, and 8%. Due to equipment overshoot at this high strain rate, the raw data were denoised to ensure accurate identification of cyclic characteristics.

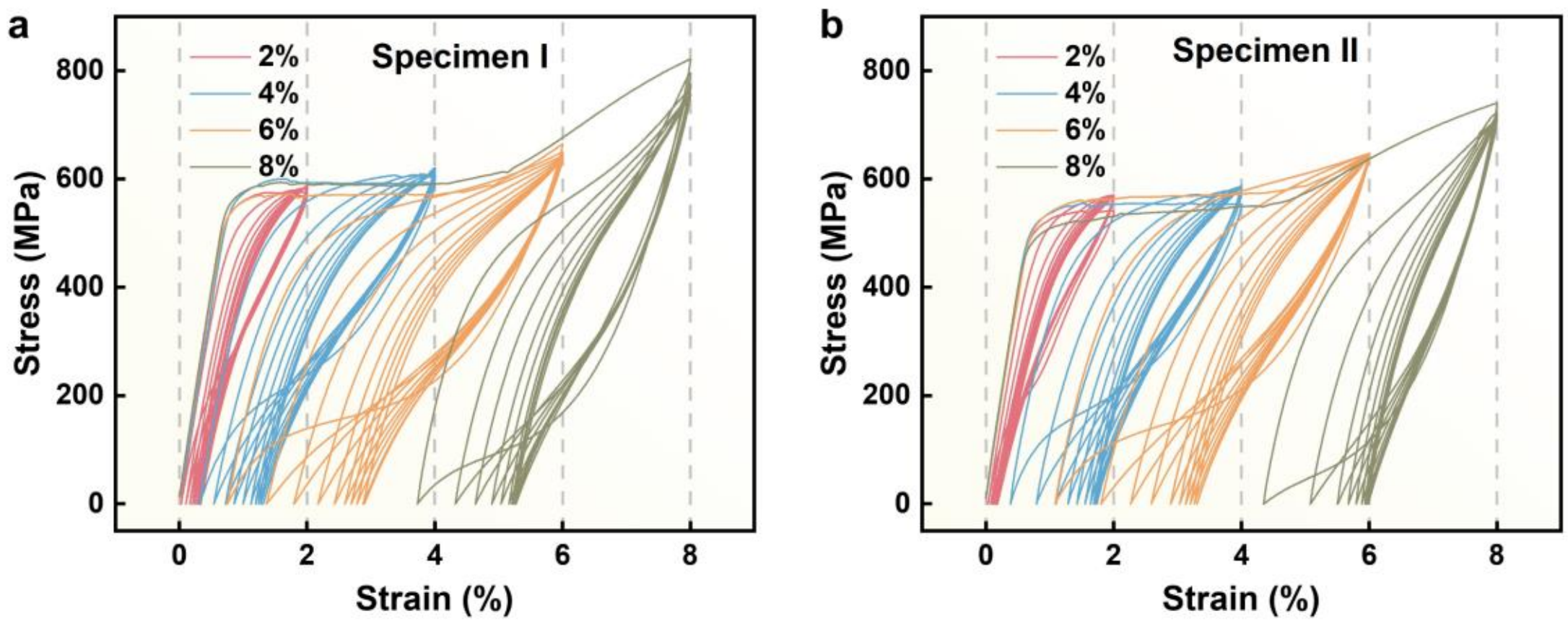

Supplementary Fig. 5 | Cyclic tensile loading–unloading engineering stress–strain curves of NiTi alloys at 40 °C under a strain rate of 5 × 10^{-4} s^{-1}. (**a, b**) correspond to specimens I and II, respectively, showing the cyclic test results under strain amplitudes of 2%, 4%, 6%, and 8%.

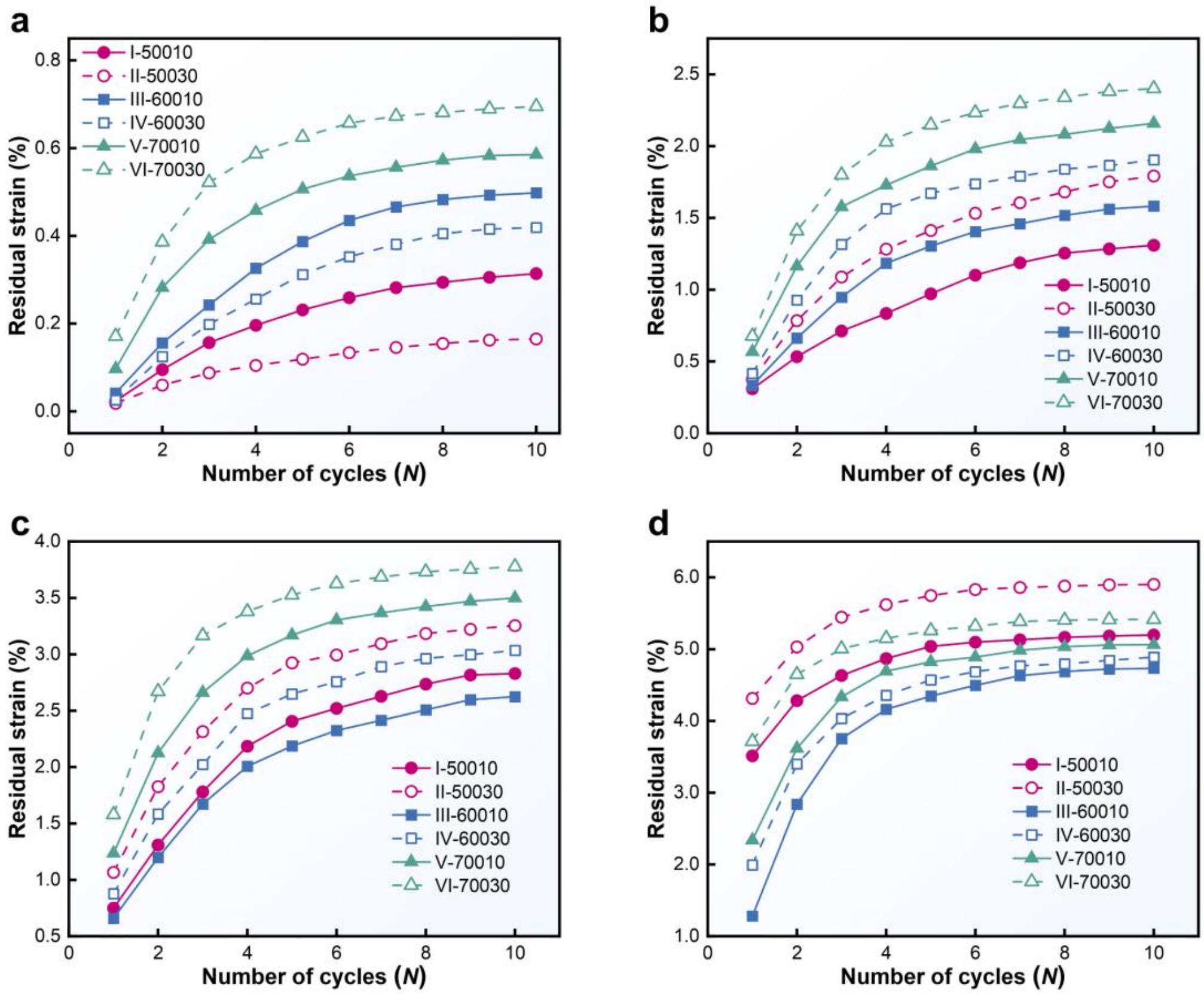

Supplementary Fig. 7 | Residual strain accumulation of NiTi alloys under various annealing conditions at a strain rate of 10^{-3} s^{-1}. Evolution of residual strain with increasing cycle number at strain amplitudes of (**a**) 2%, (**b**) 4%, (**c**) 6%, (**d**) 8%.

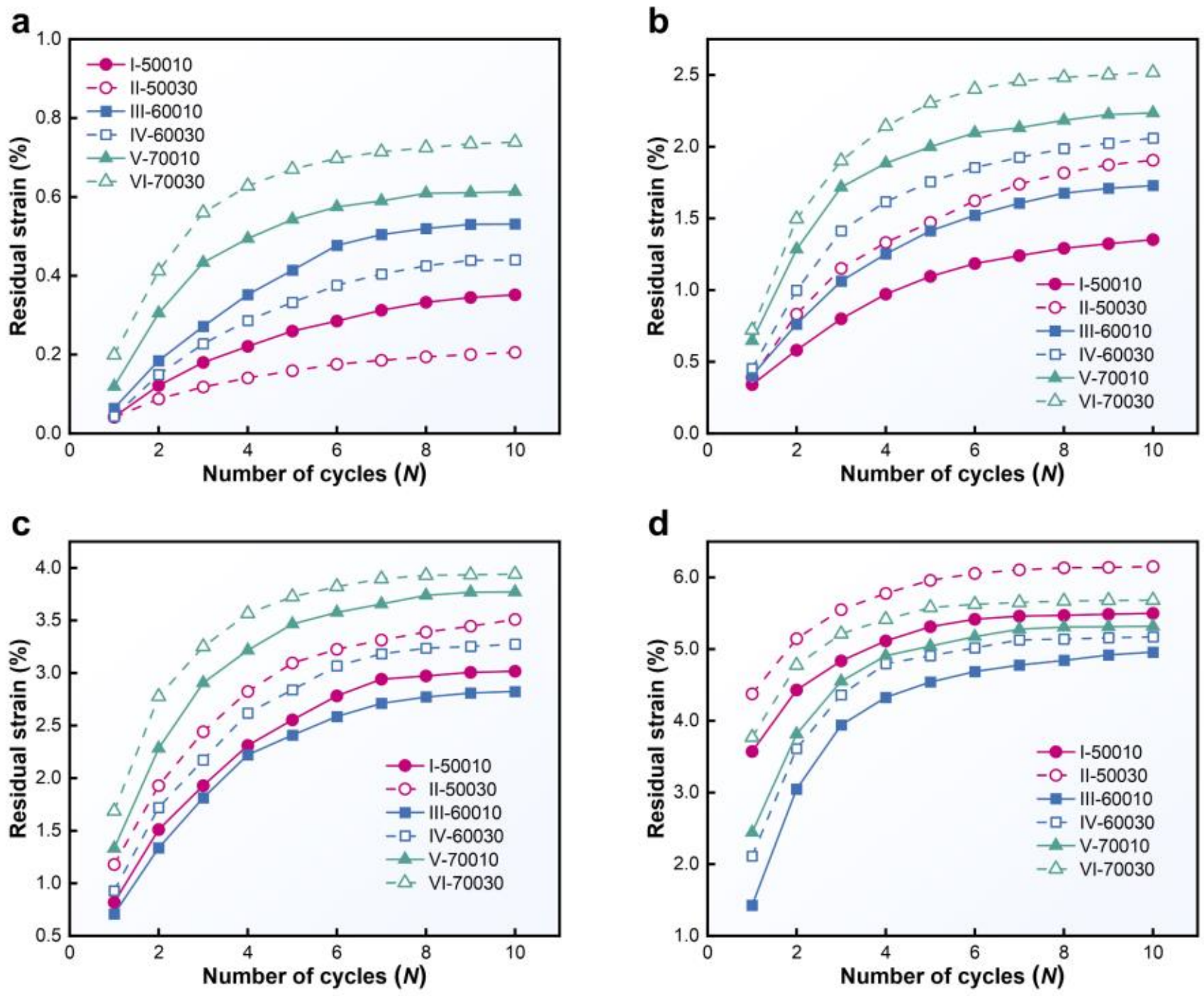

Supplementary Fig. 8 | Residual strain accumulation of NiTi alloys under various annealing conditions at a strain rate of 10^{-2} s^{-1}. Evolution of residual strain with increasing cycle number at strain amplitudes of (**a**) 2%, (**b**) 4%, (**c**) 6%, (**d**) 8%.

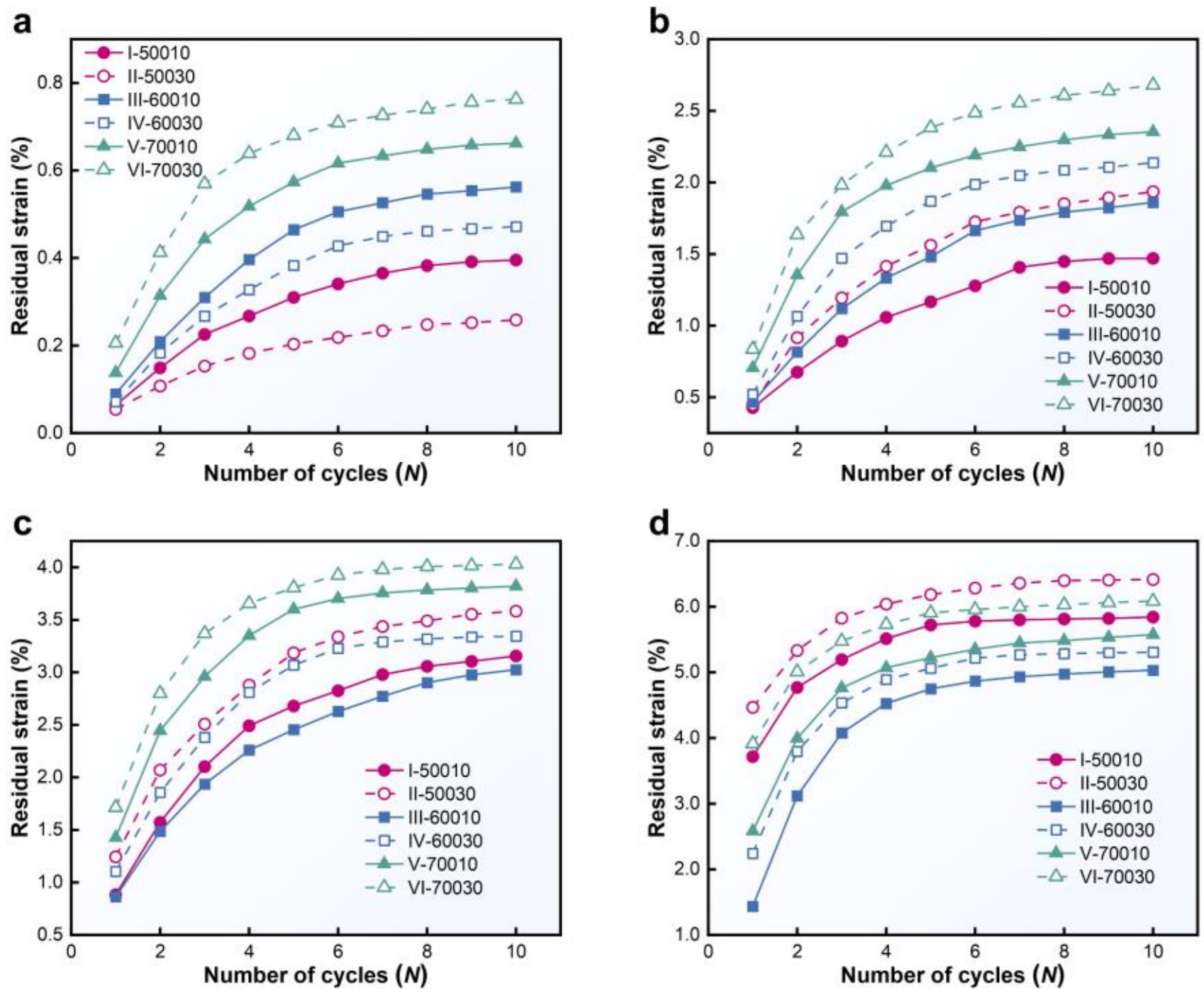

Supplementary Fig. 9 | Residual strain accumulation of NiTi alloys under various annealing conditions at a strain rate of 3×10^{-2} s^{-1}. Evolution of residual strain with increasing cycle number at strain amplitudes of (**a**) 2%, (**b**) 4%, (**c**) 6%, (**d**) 8%.

2.4 As is well known, when Ni_4Ti_3 precipitates are present in NiTi shape memory alloys, they typically induce a two-step phase transformation involving B2 to R and then R to B19'. Was this phenomenon also observed in the experiments conducted in this study?

2.4 Response: We sincerely appreciate this insightful and professional comment. The presence of Ni_4Ti_3 precipitates is well known to significantly influence phase transformation pathways in NiTi alloys. To systematically address this issue and distinguish between different driving mechanisms, we have incorporated additional DSC and in situ tensile XRD results into the Supplementary information. These results enable a separate analysis of thermally and stress-induced phase transformations. The relevant details are provided below.

First, regarding thermally induced phase transformation, the DSC results **(Supplementary Fig. 16)** exhibit clear temperature-dependent behavior. For samples annealed at 500 °C, well-separated dual exothermic peaks are observed during cooling, corresponding to the typical B2→R→B19′ two-step transformation. In contrast, as the annealing temperature increases to 600 °C and 700 °C, the dual-peak feature gradually weakens and tends to merge, indicating a diminished contribution from the R-phase transformation. Notably, under the room temperature cyclic loading conditions employed in this study, all specimens remain in a fully austenitic (B2) state. The austenite finish temperatures (A_f) range from −13.77 °C to 5.43 °C **(Supplementary Table 2)**, which are well below room temperature. This observation is further corroborated by the EBSD results.

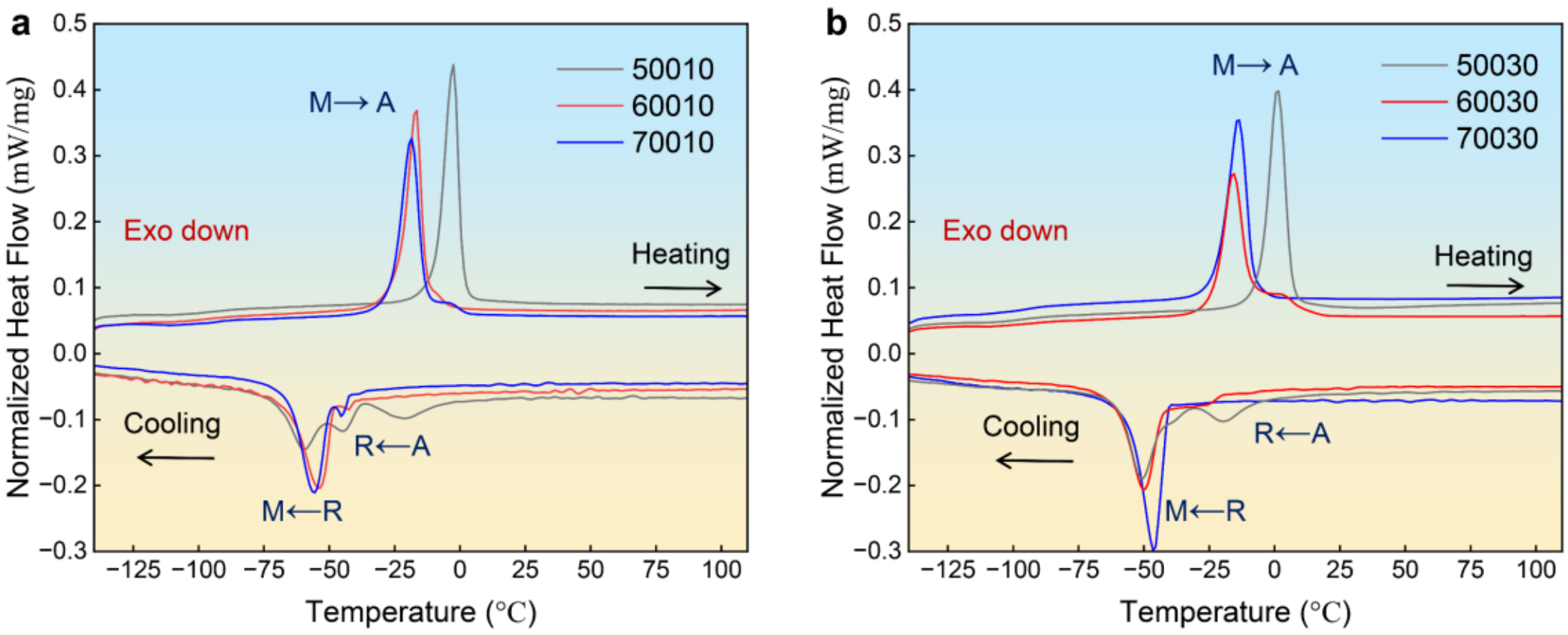

Supplementary Fig. 16 | DSC characterization of phase transformation behavior. **a** and **b** show the DSC curves for samples I, III, V and II, IV, VI, respectively. All samples remain in B2 austenite at room temperature, with the austenite finish temperatures (A_f) ranging from -13.77 °C to 5.43 °C. During heating, no distinct or well-separated double endothermic peaks are observed, indicating that the reverse transformation predominantly follows a single-step pathway. During cooling, samples annealed at 500 °C exhibit clearly separated dual exothermic peaks, characteristic of the B2→R→B19′ two-step transformation. This behavior is attributed to local internal stress fields introduced by Ni_4Ti_3 precipitates and their modulation of the local Ni concentration in the matrix (Zhou et al., 2010). With annealing temperatures increasing to 600 °C and 700 °C, the double-peak feature gradually weakens and tends to merge, indicating a transition toward a single-step B2→B19′ transformation. This evolution is associated with precipitate coarsening and redistribution, leading to reduced matrix-precipitate coherency, together with residual stress relaxation during high-temperature annealing, which collectively diminish the driving force for R phase formation.

Supplementary Table 2 | Transformation temperatures of NiTi alloys under different annealing conditions. A_s and A_f denote the start and finish temperatures of the austenite transformation during heating, while M_s and M_f represent those of the martensitic transformation during cooling

Condition	A_s (°C)	A_f (°C)	M_s (°C)	M_f (°C)
Specimen I	-9.460	1.030	-38.55	-69.74
Specimen II	-4.470	5.430	-45.15	-58.43
Specimen III	-23.57	-13.00	-48.56	-65.91
Specimen IV	-22.67	-10.61	-44.07	-59.28
Specimen V	-26.12	-13.77	-43.51	-65.00
Specimen VI	-21.80	-9.710	-42.36	-54.33

Secondly, regarding stress-induced phase transformation under room temperature tensile loading, no pronounced B2→R→B19′ two-step transformation is observed. Typically, stress-induced R-phase transformation manifests as a precursor plateau or a noticeable change in slope prior to the main transformation plateau. However, such a feature is absent in the present results **(Supplementary Fig. 1)**. Consistently, in-situ tensile XRD results **(Supplementary Fig. 23)** were measured from the specimen annealed at 500 °C for 30 min, which exhibits the most pronounced R phase transformation in DSC. The results show a progressive increase in the B19′ diffraction peaks accompanied by a continuous decrease in the B2(110) peak during loading. Notably, no discernible splitting of the B2(110) reflection into R phase peaks (e.g., (300) or (211)) is observed[28,29], nor are stable R-phase signatures detected. These observations indicate that the transformation is dominated by a direct B2→B19′ pathway under the present conditions. While a transient R-phase contribution cannot be completely excluded, it does not develop into a distinct two-step transformation.

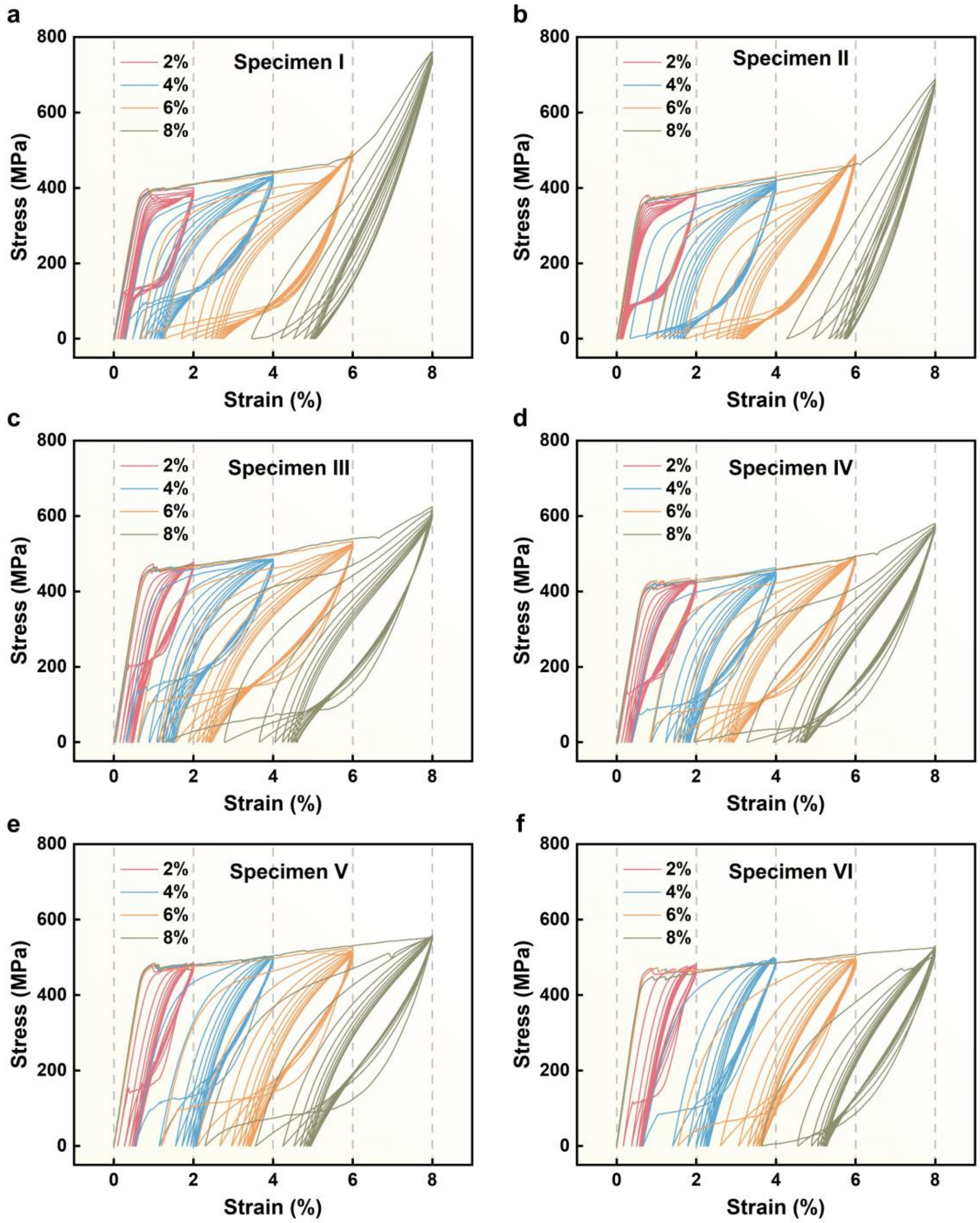

Supplementary Fig. 1 | Cyclic tensile loading–unloading engineering stress–strain curves of NiTi alloys under different annealing conditions. (**a–f**) correspond to specimens I–VI, respectively, showing the cyclic test results under strain amplitudes of 2%, 4%, 6%, and 8%.

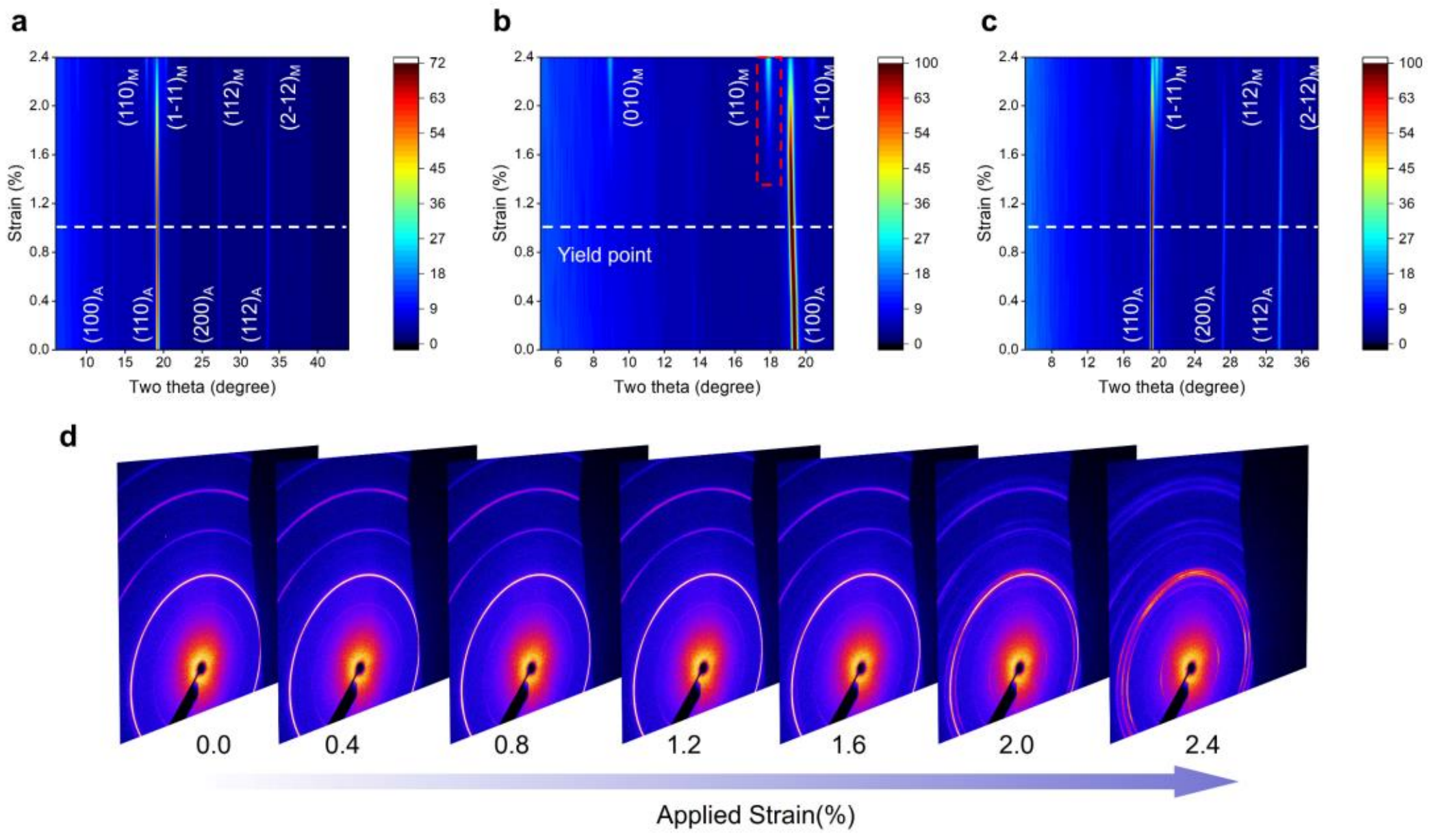

Supplementary Fig. 23 | Phase evolution during tensile loading characterized by in-situ XRD for the specimen annealed at 500 °C for 30 min. Measurements were performed using a NANOPIX-WE system with a Mo rotating anode X-ray source (λ = 0.7093 Å). **a–c** Evolution of diffraction intensity with strain along the FS, LD, and TD directions; B2 austenite (A) and B19′ martensite (M) are indicated, and dashed lines mark the yield point from the stress–strain curves. **d** 2D diffraction rings at different applied strains (0–2.4%). With increasing strain, the B19′ (1-11) and (2-12) reflections intensify within the 18.5° to 21.5° 2θ range, accompanied by attenuation of the B2 (110) peak. Notably, no discernible splitting of the B2(110) reflection into R-phase peaks (e.g., (300) or (211)) is observed, nor are any stable R-phase signatures detected (Chen et al., 2025; Chien et al., 2016). Although subtle shoulder features emerge at high strains along the LD direction, their transient nature and negligible intensity preclude their identification as a B2→R→B19′ pathway. These results demonstrate that, despite the presence of Ni_4Ti_3 precipitates, the transformation is dominated by a direct B2→B19′ pathway within the investigated thermomechanical window.

Furthermore, even if an intermediate R-phase transformation occurs during loading, its impact on the main conclusions remains limited. This is because the modeling and analysis are based on initial EBSD microstructures. Interpretability methods, such as

Grad-CAM and SHAP, operate on these descriptors to quantify their contributions to the final SED response. Accordingly, the intermediate R-phase transformation should be regarded as a transitional step in the transformation pathway. This interpretation is indirectly supported by TEM and EBSD observations of unloading samples **(Supplementary Figs. 24 and 25)**. Under high strain amplitudes, the microstructure is dominated by {114} austenite twins rather than residual martensite, suggesting that they originate from specific transformation pathways[30] (e.g., martensite variant reorientation). Although these two cases (R phase transformation and martensite variant reorientation during reverse transformation) correspond to different transformation processes during loading and unloading and do not imply mechanistic equivalence. **Nevertheless, they collectively indicate that multiple intermediate pathways may exist, without altering the dominant role of initial orientation and precipitate distribution in governing SED.**

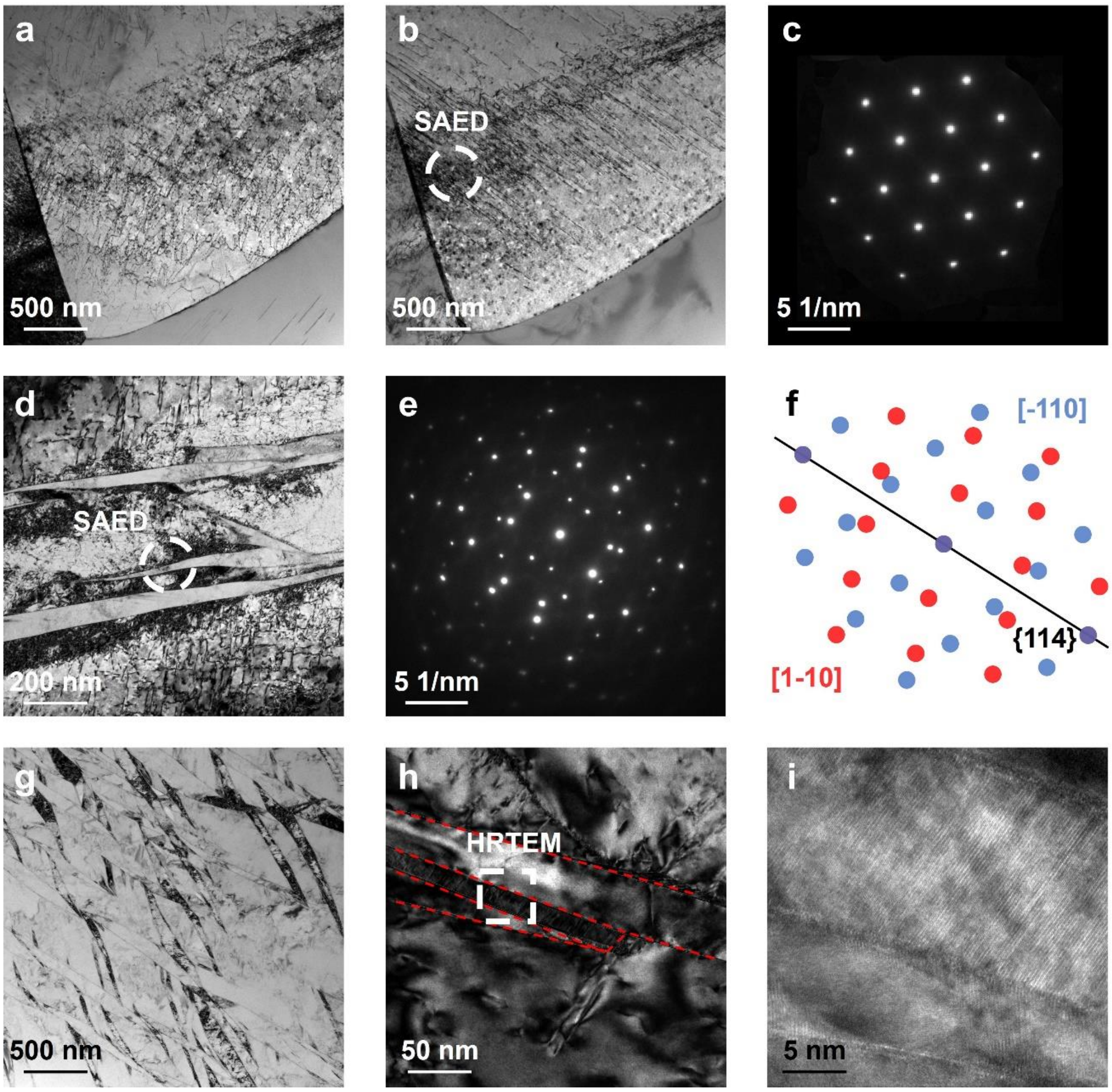

Supplementary Fig. 24 | TEM characterization of NiTi after cyclic loading at different strain amplitudes. a, **b** The bright-field images of the specimen cycled at 4% strain amplitude under different diffraction conditions, revealing a high density of dislocations but no discernible austenite twins or retained martensite plates. **c** SAED pattern from the region marked in (**b**). **d**–**f** TEM results for the specimen cycled at 6% strain amplitude, showing the appearance of {114} B2 austenite twins. **g** BF image of the specimen cycled at 8% strain, displaying extensive austenite twins. **h** Local observation of a representative twin band, and (**i**) corresponding HRTEM image from the boxed region in (**h**), where the twin boundaries are clearly resolved. Overall, superelastic degradation of the NiTi alloy under cyclic loading is primarily governed by dislocation accumulation and austenite twinning, with no retained martensite detected over the entire strain amplitude range.

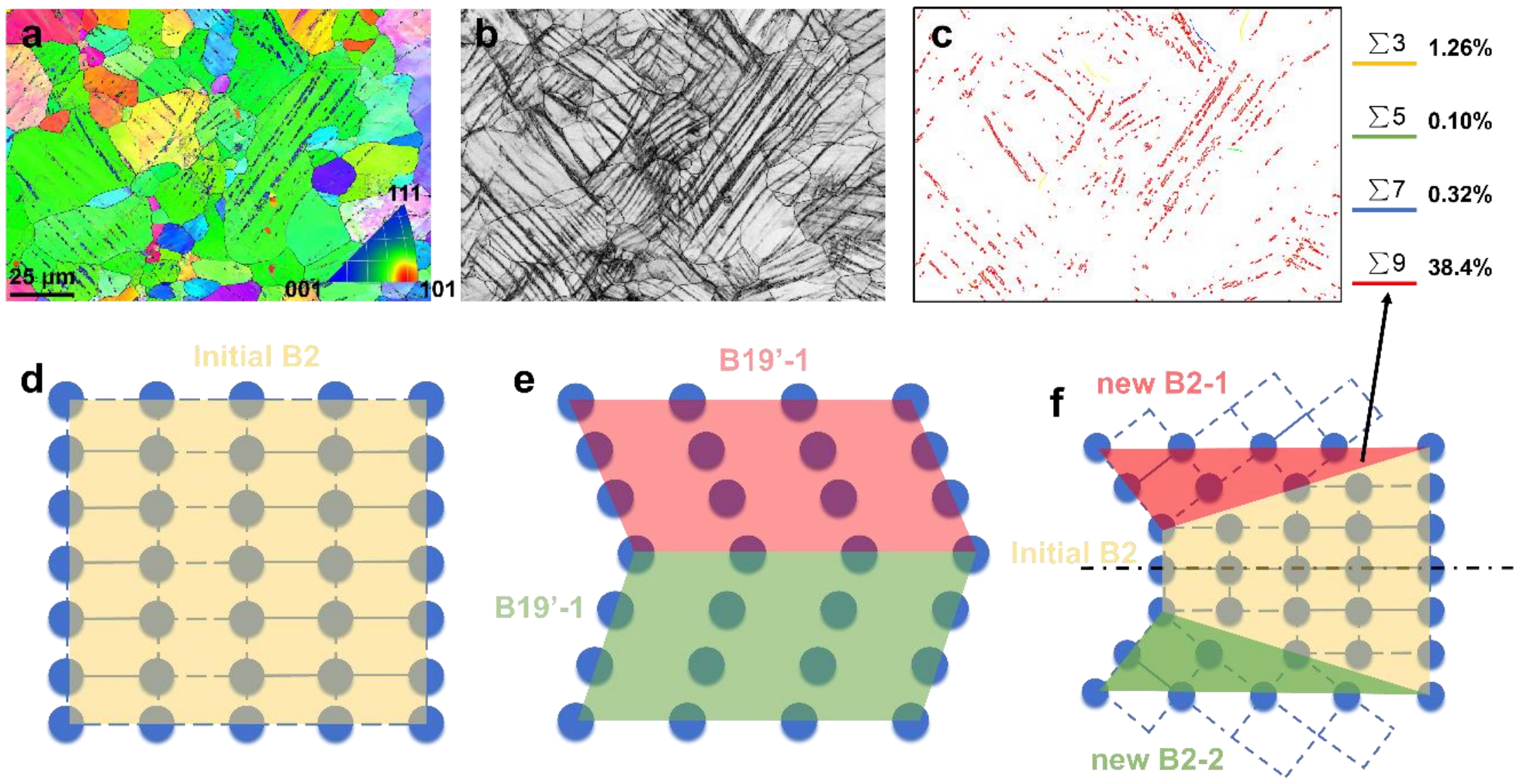

Supplementary Fig. 25 | EBSD characterization of the NiTi alloy annealed at 700 °C for 30 min after 5 cycles at 8% strain amplitude. a IPF map showing the development of a pronounced {101} texture. **b** Band contrast image, where dense deformation bands aligned along specific orientations traverse the grains after high-strain cycling. **c** Coincidence site lattice (CSL) boundary map revealing the extensive formation of Σ9 boundaries, reaching 38.4%, indicative of substantial grain boundary reconfiguration during deformation. We provide a schematic illustration of the formation mechanism of Σ9 special boundaries in B2 austenite during cyclic phase transformation. **d** The initial B2 phase (schematized using the {011} plane) exhibits a regular lattice. **e** Cyclic loading induces the formation of a composite B19′ martensite twin structure. **f** Subsequent reverse transformation produces two newly formed B2 regions with a relative lattice rotation that satisfies the Σ9 relation, thereby generating high densities of Σ9 boundaries at the microscale.

Thanks again for your valuable comment. The DSC and in-situ XRD results, together with the corresponding discussion, have been incorporated into the Supplementary information, further strengthening the analytical framework of this study.

2.5 There are some issues with the formulation of Equation (4). The meaning of Alpha on the right side of the equation should be clarified. Additionally, D_{klmn} is not an Eshelby tensor itself, but rather a quantity constructed from the Eshelby tensor. Additionally, since the orientations of different Ni_4Ti_3 precipitates are not uniform, this can lead to the induction of various internal stress fields with different orientations. How does this paper account for this aspect? It is also recommended to include the explicit expressions of D_{klmn} and the lattice mismatch strain in the supplementary materials.

2.5.1 There are some issues with the formulation of Equation (4). The meaning of Alpha on the right side of the equation should be clarified.

2.5.1 **Response:** We sincerely appreciate this helpful comment. The equation referred to is the Eshelby-based local stress correction (Equation (2) in the revised manuscript; Equation (5) in the Supplementary information). We have clarified that α_{ij}' is the Schmid orientation tensor (projection tensor) of the considered deformation system, which projects the precipitate-induced internal stress field onto the resolved shear stress. The revision is incorporated into the **revised manuscript (Page 31, Lines 2–3)** as follows: Where α_{ij}' is the orientation tensor expressed in the principal coordinate system of the precipitate.

2.5.2 Additionally, D_{klmn} is not an Eshelby tensor itself, but rather a quantity constructed from the Eshelby tensor.

2.5.2 **Response:** We sincerely appreciate your professional reminder. In the classical sense, the Eshelby tensor is the constant tensor S_{klmn} defined within an ellipsoidal inclusion. However, the tensor $D_{klmn}(x)$ employed here is an exterior field transmission tensor derived from the Eshelby inclusion solution. It varies with spatial position x and relates the precipitate misfit eigenstrain ε_{mn}^{p} to the induced elastic field in the surrounding matrix. In the revised manuscript, a concise description is

provided in the **Calculation and correction of the Schmid factor section**, while detailed mathematical derivations and parameter definitions are given in the **Supplementary information**. The corresponding revisions comprise two parts.

The revision in the **manuscript (Page 31, Lines 3–5)** is as follows: $D_{klmn}(x)$ is a fourth-order interaction tensor constructed from the Eshelby tensor that relates the precipitate eigenstrain to the induced elastic field in the matrix at any position x.

The revision in the **Supplementary information (Page 28, Lines 4–7)** is as follows: The tensor $D_{klmn}(x)$ is constructed from the Eshelby tensor for the stress fields exterior to the inhomogeneity. This tensor is a function of the position vector outside the precipitate, x, the geometry of the precipitate, and the elastic constants of the precipitate (Tirry and Schryvers, 2005).

2.5.3 Additionally, since the orientations of different Ni_4Ti_3 precipitates are not uniform, this can lead to the induction of various internal stress fields with different orientations. How does this paper account for this aspect?

2.5.3 **Response:** We agree that Ni_4Ti_3 precipitates can appear as multiple symmetry-related orientation variants, thereby inducing localized internal stress fields with diverse orientations in the matrix. Experimentally, pole figure and SAED analyses confirm a specific orientation relationship between Ni_4Ti_3 and the B2 matrix {123}<111>$_{B2}$ // {11-20}<0001>$_{Ni4Ti3}$, with the precipitates exhibiting a uniform distribution **(Supplementary Fig. 20)**.

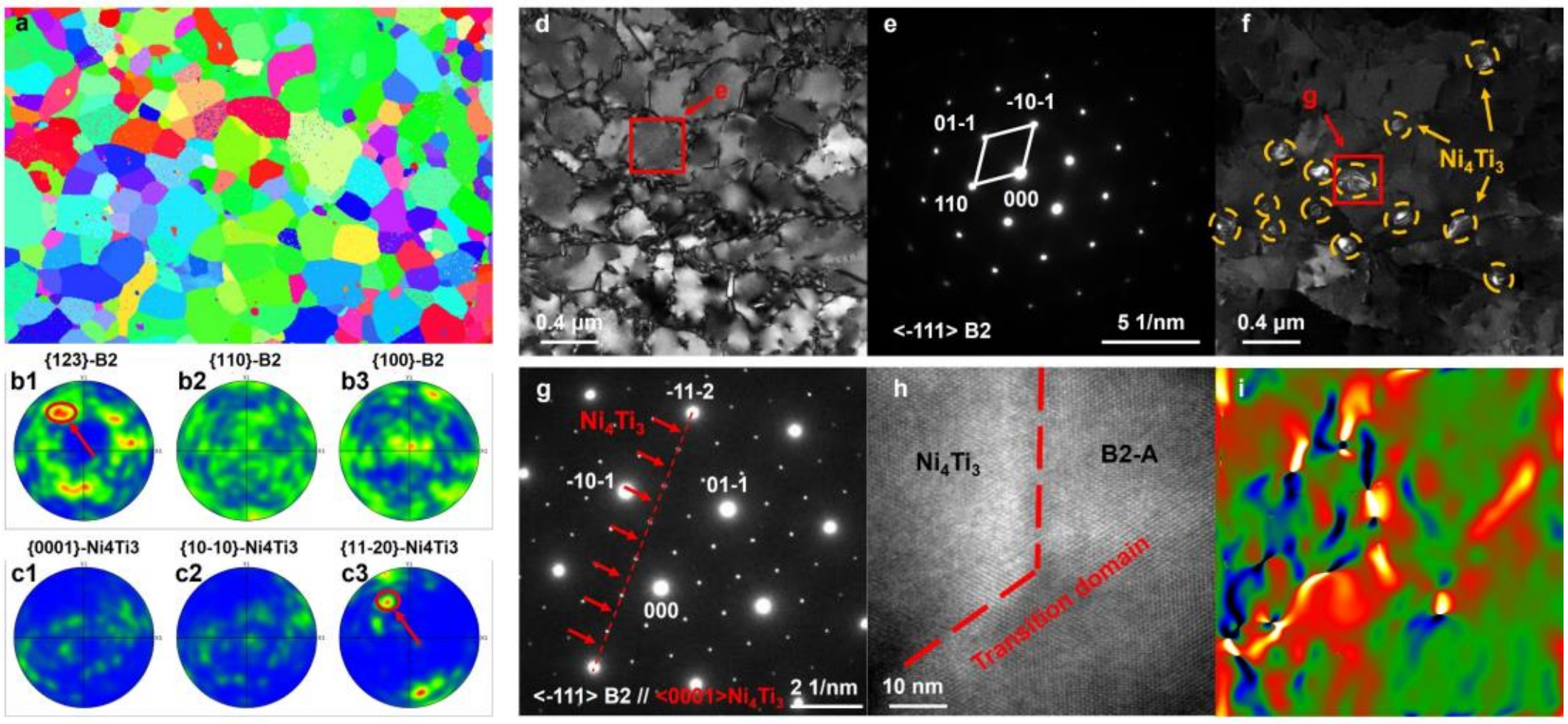

Supplementary Fig. 20 | Orientation relationship between Ni_4Ti_3 precipitates and the B2 austenite matrix. a Orientation distribution map. **b_1–b_3** {123}, {110}, and {100} pole figures of the B2 austenite matrix. **c_1–c_3** {0001}, {10-10}, and {11-20} pole figures of the Ni_4Ti_3 precipitates. **d** Bright-field TEM image of the overall region, and (**e**) the selected area electron diffraction (SAED) pattern taken from the red boxed area in (**d**). **f** Corresponding dark-field image, with (**g**) and (**h**) showing the SAED pattern and high-resolution TEM (HRTEM) image from the red boxed region in (**f**), respectively. **i** Geometric phase analysis (Hytch et al., 1998) (GPA) strain map corresponding to (**h**). No common crystallographic plane was identified between Ni_4Ti_3 and the B2 matrix from the pole figures. Nevertheless, the pole figure and SAED analyses reveal a specific orientation relationship of $\{123\}\langle 111\rangle_{B2}$ // $\{11\text{-}20\}\langle 0001\rangle_{Ni4Ti3}$. The dark-field image demonstrates that precipitates are uniformly distributed as nanoscale particles. HRTEM and GPA strain mapping indicate that the formation of Ni_4Ti_3 precipitates induces strain inhomogeneity in the NiTi matrix, with significant local tensile strain in regions of continuous distribution.

This work introduces precipitation-induced internal stresses at the EBSD pixel scale. For each pixel, based on the local B2 orientation and the orientation relationship between Ni_4Ti_3/B2, the transformation of the precipitate principal coordinate system relative to the sample frame is determined, enabling calculation of the precipitate-induced internal stress. This internal stress is then combined with the

applied stress to define the local effective stress, which is projected onto candidate deformation systems (slip systems or martensitic transformation associated with the variant pair, CVP) to obtain the local resolved shear stress and the corrected Schmid factor. Since the spatial distribution and volume fractions of individual symmetry-related precipitate variants cannot be quantitatively resolved at the current EBSD resolution, a mean-field approximation based on variant averaging is adopted. **This approach represents the collective effect of multiple variants as an effective correction to the resolved shear stress.** Similar treatments of precipitate variants and averaged internal effective shear stress have been reported in microstructural mechanical analyses of NiTi precipitation hardening based on Eshelby theory[31,32].

We have clarified this assumption and added the corresponding explanations in the **Supplementary information (Page 27, Lines 24–30 and Page 28, Lines 1–2)** as follows: It should be clarified that Ni_4Ti_3 precipitates follow a specific orientation relationship with the B2 matrix, as verified by the pole figure and SAED analyses (Supplementary Fig. 20). Accordingly, although multiple symmetry-related precipitate variants may exist, the precipitate principal coordinate system in the specimen frame is determined from the local B2 orientation together with this orientation relationship (Li et al., 2021). In the present implementation, the precipitate-induced internal stress correction is evaluated at each EBSD pixel using the corresponding matrix orientation and is then applied to each martensitic variant to obtain the corrected Schmid factor. Since the explicit population of Ni_4Ti_3 variants is not resolved, the variant diversity is represented in terms of an effective contribution to the resolved shear stress.

2.5.4 It is also recommended to include the explicit expressions of D_{klmn} and the lattice mismatch strain in the supplementary materials.

2.5.4 Response: Thanks again for your valuable suggestion. To more clearly illustrate the derivation process and parameter definitions, we have added explicit expressions for $D_{klmn}(x)$ and lattice mismatch strain ε^{p}_{mn} to the **Supplementary information (Page 28, Lines 4–20)**. Their descriptions are as follows:

The tensor $D_{klmn}(x)$ is constructed from the Eshelby tensor for the stress fields exterior to the inhomogeneity. This tensor is a function of the position vector outside the precipitate, x, the geometry of the precipitate, and the elastic constants of the precipitate (Tirry and Schryvers, 2005). For an isotropic elastic medium, it is given by:

$$\begin{aligned} 8\pi(1-v)D_{klmn}(x) &= \psi_{,mnkl}(x) - 2v\delta_{mn}\phi_{,kl}(x) - \\ &(1-v)[\phi_{,kj}(x)\delta_{ln} + \phi_{,lm}(x)\delta_{kn} + \phi_{,kn}(x)\delta_{lm} + \phi_{,ln}(x)\delta_{km}] \end{aligned} \quad (2)$$

with the scalar potentials:

$$\psi(x) = \int_{\Omega} \left| x - x' \right| \mathrm{d}x', \ \phi(x) = \int_{\Omega} \frac{1}{\left| x - x' \right|} \mathrm{d}x' \quad (3)$$

Where Ω denotes the precipitate domain, and x' is the integration variable within Ω. v is the Poisson's ratio of the B2 matrix. δ_{mn} is the Kronecker delta, defined as $\delta_{mn} = 1$ when $m = n$ and $\delta_{mn} = 0$ otherwise. The comma notation denotes partial differentiation with respect to the components of the field point x. For example, $\psi_{,mnkl}(x) = \dfrac{\partial^4 \psi}{\partial x_m \partial x_n \partial x_k \partial x_l}$. $\varepsilon^{\mathrm{p}}_{mn}$ represents the intrinsic lattice-misfit strain of the Ni_4Ti_3 precipitate, corresponding to the stress-free transformation strain arising from the crystallographic lattice correspondence between the B2 matrix and the rhombohedral Ni_4Ti_3 phase (Gall et al., 1999). In the principal coordinate system of the precipitate (with the 3-axis aligned with its symmetry axis), the misfit strain tensor is approximated as a uniform diagonal form:

$$\varepsilon^{\mathrm{p}}_{mn} = \begin{bmatrix} \varepsilon^{\mathrm{p}}_{11} & 0 & 0 \\ 0 & \varepsilon^{\mathrm{p}}_{22} & 0 \\ 0 & 0 & \varepsilon^{\mathrm{p}}_{33} \end{bmatrix} = \begin{bmatrix} -4.17 \times 10^{-3} & 0 & 0 \\ 0 & -4.17 \times 10^{-3} & 0 \\ 0 & 0 & -2.57 \times 10^{-2} \end{bmatrix} \quad (4)$$

2.6 In recent years, several multi-scale models have also been developed, which to some extent reveal the relationship between the characteristic microstructure of NiTi shape memory alloys and the macroscopic superelastic degradation (J. Mech. Phys. Solids, 2015, 82, 97-136; Int. J. Solids Struct., 2023, 280, 112384). I suggest that the authors provide a brief review of theses works.

2.6 Response: We sincerely appreciate your highlighting of these important multiscale modeling studies on NiTi SMAs. Physically based multiscale constitutive frameworks provide a rigorous basis for understanding how microstructural features, such as crystallographic orientation and texture, govern the macroscopic evolution of cyclic SED behavior.

After carefully studying the studies you mentioned, we draw the following insights: Yu et al. (J. Mech. Phys. Solids, 2015, 82, 97–136) established a micromechanical constitutive model for cyclic SED of NiTi single crystals, emphasizing the role of crystallographic anisotropy in response evolution and providing a mechanistic explanation for macroscopic degradation. In parallel, Kan et al. (Int. J. Solids Struct., 2023, 280, 112384), through combining experiments and multiscale modeling, demonstrated that texture and microstructure characteristics of polycrystalline materials, as well as the coupling between phase transformation and slip, lead to distinct macroscopic degradation pathways, particularly manifested in tensile–compressive asymmetry. **Following this review, we clarified the complementary roles of data-driven approaches and physics-based constitutive models in revealing degradation mechanisms of NiTi alloys.** In addition to other relevant studies, two representative works have been incorporated and discussed in the Introduction and Discussion sections. The rationale for these revisions is as follows:

First, the work of Yu et al. (JMPS, 2015)[33] primarily originates from physical mechanisms, using constitutive models to interpret and predict degradation processes. In contrast, our research begins with real multimodal data, from which microstructural representations are extracted using ML, and feature contributions are quantified via Grad-CAM and SHAP. **From a methodological perspective, the former follows a**

top-down paradigm, whereas the latter adopts a bottom-up strategy. Importantly, these two approaches are complementary. The data-driven framework can provide clues for feature extraction and validation of multiscale models, while multiscale models offer physical mechanism guidance for interpreting correlations revealed by ML.

Second, Kan and Kang (Int. J. Plasticity, 2010)[34] developed a sophisticated thermodynamic constitutive framework to analyze the uniaxial phase transformation ratcheting behavior in NiTi alloys. They introduced an internal variable $z_c = \int_0^t |\dot{z}(s)|\, ds$ to characterize the accumulated volume fraction of residual martensite (more precisely, the accumulated change in volume fraction), thereby encoding the historical effect of phase transformation during cycling. This variable governs the evolution of transformation stress, residual martensite fraction, and transformation-induced plasticity. This approach provides important inspiration for the present study. In the early stages, introducing similar cumulative variables was also considered. However, we ultimately did not introduce explicit internal variables and instead used experimentally accessible descriptors (e.g., cycle number and strain amplitude) to represent loading history. Because we intend to ensure consistency in a data-driven manner while maintaining experimental observability. The model is expected to learn implicit representations of history effects while preserving interpretability under the available data conditions.

Finally, the revisions to the **Introduction and Discussion sections** are as follows:

1. In the **Introduction section (Page 3, Lines 23–28 and Page 4, Lines 1–4)**: Pioneers have predominantly employed phenomenological or semi-empirical models, such as parameterized constitutive equations, to describe cyclic stress-strain behavior (Kan and Kang, 2010; Otsuka and Ren, 2005; Yu et al., 2021b; Zadeh et al., 2025). These approaches are typically constructed within the framework of continuum mechanics, building upon predefined physical assumptions and requiring extensive experimental calibration and parameter optimization. However, as noted above, variable control in these studies is constrained. More importantly, parameter fitting

often requires manual feature extraction or the spatial averaging of microstructural images (Pei et al., 2021) to obtain usable inputs. While this digitization simplifies the modeling process, it frequently introduces subjective bias and compromises the integrity of spatial structural information.

2. In the **Discussion section (Page 22, Lines 19–26)**: Against this backdrop, the interpretable ML framework demonstrates a quasi-in-situ mechanistic reconstruction approach, compensating for the lack of true full-field observation through either experimental techniques or theoretical modeling. Unlike molecular dynamics simulations, which hinge on precise potentials and idealized initial configurations (Ko et al., 2015), this framework spontaneously learns patterns from real microstructures and corroborates them with independent findings. Such reconstruction bridges the existing observational gap and lays the foundation for calibrating multiscale theoretical models (Yu et al., 2015; Zadeh et al., 2025).

Finally, many thanks for the valuable comments and time given. We are impressed by how constructive and targeted each of your suggestions is. Based on this, we have carefully revised and polished the manuscript to meet the journal's high standards. Once again, we express our sincere gratitude and admiration.

References

1. Sutou, Y. et al. Effect of grain size and texture on pseudoelasticity in Cu–Al–Mn-based shape memory wire. *Acta Mater.* **53**, 4121–4133 (2005).
2. Weidner, A. et al. In situ characterization of the functional degradation of a $[00\bar{1}]$ orientated Fe–Mn–Al–Ni single crystal under compression using acoustic emission measurements. *Acta Mater.* **220**, 117333 (2021).
3. Song, K., Xu, S., Kainuma, R. & Omori, T. Texture evolution in polycrystalline FeMnAlNi shape-memory alloy sheets. *Acta Mater.* **301**, 121554 (2025).
4. Ren, D., Wang, C., Wei, X., Lai, Q. & Xu, W. Building a quantitative composition-microstructure-property relationship of dual-phase steels via multimodal data mining. *Acta Mater.* **252**, 118954 (2023).
5. Wang, X. et al. Transformer-based multimodal learning for predicting mechanical properties in heat-treated stainless steel. *Mater. Des.* **259**, 114800 (2025).
6. Arghavani, J., Auricchio, F., Naghdabadi, R., Reali, A. & Sohrabpour, S. A 3-D phenomenological constitutive model for shape memory alloys under multiaxial loadings. *Int. J. Plast.* **26**, 976–991 (2010).
7. Gorji, M., Mozaffar, M., Heidenreich, J., Cao, J. & Mohr, D. On the potential of recurrent neural networks for modeling path dependent plasticity. *J. Mech. Phys. Solids* **143**, 27 (2020).
8. Choi, W. et al. Finely tunable thermal expansion of NiTi by stress-induced martensitic transformation and thermomechanical training. *Acta Mater.* **302**, 12 (2026).
9. Yang, B., Vassilev-Galindo, V. & Llorca, J. Application of machine learning to assess the influence of microstructure on twin nucleation in Mg alloys. *npj Comput. Mater.* **10**, 26 (2024).
10. Guru, M., Bohlen, J., Aydin, R. & Ben Khalifa, N. Machine learning pipeline for Structure-Property modeling in Mg-alloys using microstructure and texture descriptors. *Acta Mater.* **295**, 26 (2025).
11. Jangid, D. K. et al. Adaptable physics-based super-resolution for electron backscatter diffraction maps. *npj Comput. Mater.* **8**, 255 (2022).
12. Cobo, A., Valle, R., Buenaposada, J. M. & Baumela, L. On the representation and methodology for wide and short range head pose estimation. *Pattern Recognit.* **149**, 110263 (2024).
13. Zhou, Y. et al. On the Continuity of Rotation Representations in Neural Networks. In: *32nd IEEE/CVF Conference on Computer Vision and Pattern Recognition (CVPR)*). IEEE Computer Soc (2019).
14. Narong, T., Zachko, Z., Torrisi, S. & Billinge, S. Interpretable multimodal machine learning analysis of X-ray absorption near-edge spectra and pair distribution functions. *npj Comput. Mater.* **11**, 12 (2025).
15. Song, D., Gong, S., Xu, B. & Yu, C. Cyclic functional degradation of NiTi shape memory alloy wires in wide ranges of strain rate and ambient temperature. *International Journal of Fatigue* **191**, 108683 (2025).
16. Delville, R., Malard, B., Pilch, J., Sittner, P. & Schryvers, D. Transmission electron microscopy investigation of dislocation slip during superelastic cycling

of Ni–Ti wires. *Int. J. Plast.* **27**, 282–297 (2011).
17. He, Y. J. & Sun, Q. P. On non-monotonic rate dependence of stress hysteresis of superelastic shape memory alloy bars. *International Journal of Solids and Structures* **48**, 1688–1695 (2011).
18. Kan, Q., Yu, C., Kang, G., Li, J. & Yan, W. Experimental observations on rate-dependent cyclic deformation of super-elastic NiTi shape memory alloy. *Mech. Mater.* **97**, 48–58 (2016).
19. Lee, T.-J. & Kim, W.-J. Effect of severe plastic deformation and post-deformation heat treatment on the microstructure and superelastic properties of Ti-50.8 at.% Ni alloy. *Materials* **15**, 7822 (2022).
20. Chen, W. et al. Superelasticity of geometrically graded NiTi shape memory alloys. *Metals* **13**, 1518 (2023).
21. Li, P., Liu, Y., Shi, X. & Liu, P. Micromechanism of superelastic instability of nanocrystalline NiTi shape memory alloys. *J. Mater. Res. Technol.* **37**, 5360–5368 (2025).
22. Wang, Q. S. et al. Leveraging supervised learning for functionally informed fine-mapping of cis-eQTLs identifies an additional 20,913 putative causal eQTLs. *Nat. Commun.* **12**, 3394 (2021).
23. Salomon, A. et al. A machine learning contest enhances automated freezing of gait detection and reveals time-of-day effects. *Nat. Commun.* **15**, 4853 (2024).
24. Tsang, T. K., Du, Q., Cowling, B. J. & Viboud, C. An adaptive weight ensemble approach to forecast influenza activity in an irregular seasonality context. *Nat. Commun.* **15**, 8625 (2024).
25. Guo, D. et al. Integrating spoken instructions into flight trajectory prediction to optimize automation in air traffic control. *Nat. Commun.* **15**, 9662 (2024).
26. Tan, M. et al. Image-based DNA sequencing encoding for detecting low-mosaicism somatic mobile element insertions. *Nat. Commun.* **16**, 9195 (2025).
27. Li, S.-W., Xu, L.-C., Zhang, C., Zhang, S.-Q. & Hong, X. Reaction performance prediction with an extrapolative and interpretable graph model based on chemical knowledge. *Nat. Commun.* **14**, 3569 (2023).
28. Chien, C. et al. Characteristics of the strain glass transition in as-quenched and 250 °C early-aged Ti48.7Ni51.3 shape memory alloy. *Acta Mater.* **120**, 159–167 (2016).
29. Chen, Y. et al. Lattice distortion and atomic shuffle coupling of the R phase under grain size confinement in a Ni48Ti50Fe2 alloy. *Acta Mater.* **292**, 14 (2025).
30. Heller, L. et al. Beyond the strain recoverability of martensitic transformation in NiTi. *Int. J. Plast.* **116**, 232–264 (2019).
31. Baxevanis, T., Cox, A. & Lagoudas, D. C. Micromechanics of precipitated near-equiatomic Ni-rich NiTi shape memory alloys. *Acta Mechanica* **225**, 1167–1185 (2014).
32. Gall, K., Sehitoglu, H., Chumlyakov, Y., Zuev, Y. & Karaman, I. The role of coherent precipitates in martensitic transformations in single crystal and polycrystalline Ti-50.8at%Ni. *Scr. Mater.* **39**, 699–705 (1998).

33. Yu, C., Kang, G. & Kan, Q. A micromechanical constitutive model for anisotropic cyclic deformation of super-elastic NiTi shape memory alloy single crystals. *J. Mech. Phys. Solids* **82**, 97–136 (2015).
34. Kan, Q. & Kang, G. Constitutive model for uniaxial transformation ratchetting of super-elastic NiTi shape memory alloy at room temperature. *Int. J. Plast.* **26**, 441–465 (2010).

Detailed Response to Reviewers' Comments

Thanks to the reviewers for their valuable comments and suggestions on our manuscript. We hope that the careful revisions made to improve the accuracy and clarity of the presentation have adequately addressed the constructive concerns you raised. We again thank you for your time in reviewing our manuscript and making it a much more robust and impactful study. The modifications based on the comments, along with detailed answers to those comments, are as follows.

Note that:

(1) The reply to Reviewer 1's comments is listed on Pages 1-9;

(2) The reply to Reviewer 2's comments is listed on Page 10;

The colours of the text correspond to responses to Reviewer 1 - **Colour 1**, Reviewer 2 – **Colour 2**, with all revisions highlighted accordingly.

Reviewer 1: The authors did an excellent job leveraging the reviewer comments and scientific discussion to significantly improve the manuscript. Overall, the revised version is substantially stronger, both technically and scientifically, compared to the original submission. In particular, I appreciate the considerable effort dedicated to strengthening the interpretability analysis, latent-space characterization, generalization assessment, and clarification of the data partitioning strategy. The additions made in response to the review comments clearly improve the rigor, transparency, and overall impact of the work.

The revised manuscript now provides a much more convincing demonstration that the framework captures physically meaningful structure–deformation relationships rather than relying on spurious correlations or potential information leakage. The clarification regarding the partitioning of the dataset at the original EBSD scan-region level is especially important given the very high predictive performance reported. The additional cross-validation, learning-curve analysis, and convergence assessments also help strengthen confidence in the robustness and stability of the model. I also

believe the newly added latent-space visualizations, grouped SHAP analyses, and multimodal representation analysis substantially improve the interpretability aspect of the study and were important additions to the manuscript as suggested by reviewer 1.

The quantitative analysis of the transition between slip-dominated and transformation-dominated regimes, together with the discussion of the loading-dependent role of Ni4Ti3 precipitates, adds significantly more mechanistic depth than in the original version. The efforts to connect the learned representations to geometric compatibility, local deformation evolution, and variant-transfer behavior provide a much clearer mechanistic narrative and improve the overall scientific value of the work.

That said, I would still encourage the authors to remain somewhat cautious in the framing of the conceptual novelty and the broader generality of the approach. The work is clearly strong, technically sophisticated, and impactful, but several of the identified mechanisms remain broadly consistent with existing qualitative understanding in the NiTi literature. In my opinion, the manuscript is strongest when positioned as an interpretable multimodal framework capable of quantitatively resolving and visualizing degradation processes that were previously difficult to characterize spatially and systematically, rather than as the discovery of fundamentally new deformation physics. Similarly, while the discussion regarding extensibility has been significantly improved, the experimental validation remains largely confined to the NiTi system and related loading conditions, and I would recommend maintaining a relatively measured tone regarding broader transferability.

Overall, I believe the authors addressed the major concerns raised in the original review in a serious, thoughtful, and technically rigorous manner, and the manuscript has been significantly strengthened as a result.

1.0 **Response:** We sincerely thank you for the careful re-evaluation and encouraging comments. We appreciate the recognition of our revisions and the valuable suggestion regarding cautious framing. We have further moderated the relevant statements on conceptual novelty and broader generality. The detailed responses are provided below.

1.1 That said, I would still encourage the authors to remain somewhat cautious in the framing of the conceptual novelty and the broader generality of the approach. The work is clearly strong, technically sophisticated, and impactful, but several of the identified mechanisms remain broadly consistent with existing qualitative understanding in the NiTi literature. In my opinion, the manuscript is strongest when positioned as an interpretable multimodal framework capable of quantitatively resolving and visualizing degradation processes that were previously difficult to characterize spatially and systematically, rather than as the discovery of fundamentally new deformation physics. Similarly, while the discussion regarding extensibility has been significantly improved, the experimental validation remains largely confined to the NiTi system and related loading conditions, and I would recommend maintaining a relatively measured tone regarding broader transferability.

1.1 Response: Thank you very much for your insightful and constructive feedback. Your comment addresses two interrelated yet distinct, pivotal issues: first, the precise positioning of our conceptual innovation; and second, the scope of methodological extensibility and the boundary conditions supported by our current evidence. We fully agree that all claims must be rigorously grounded in the actual research findings and the validation range, avoiding any conflation of architectural potential with universally proven conclusions. In light of the valuable suggestion, we have carefully re-examined the manuscript and systematically revised the relevant text across the Abstract, Introduction, Results, and Discussion sections. Our detailed responses are provided below.

1. Conceptual innovation and contribution of this work

You pointed out that this paper would be more appropriately positioned as an interpretable multimodal framework for quantitative analysis and visualization of material degradation, rather than as a novel discovery in fundamental deformation physics. We entirely agree with this astute assessment. From our perspective, the primary contribution lies in the methodological advancement of proposing an interpretable multimodal learning framework tailored to capture the complex

relationships among microstructures, loading conditions, and macroscopic performance.

Specifically, compared with traditional multimodal architectures applied to metallic materials, **our framework establishes a more explicit, physically meaningful mapping from raw multi-source microstructural data to macroscopic mechanical responses**. In particular, we introduce the concept of channel independence for feature decoupling within the domain of multimodal fusion for metallic materials. By maintaining relative independence among distinct input branches, the model effectively prevents cross-interference between microstructural features and loading parameters. This architecture enables highly targeted feature importance analysis and spatial visualization through controlled-variable experiments (e.g., evaluating degradation while fixing specific strain amplitudes or cycle numbers), which underpins the unique capacity for quantitative and visual analysis.

At the same time, we wish to clarify that positioning the contribution this way does not imply that the mechanistic insights merely repeat existing knowledge. The feature attributions and spatial visualizations generated by the framework emerge directly from the underlying multimodal data distributions themselves, free from prior physical assumptions or manual preconceptions. The framework thus serves as an exploratory bridge between raw data with physical mechanisms. On this basis, **we further cross-validate these purely data-driven attributions against established physical mechanisms** using Schmid factor analysis, transmission electron microscopy (TEM) observations, and molecular dynamics (MD) simulations. As noted in the comment, avoiding the framing of this work as a discovery of new physics is scientifically appropriate. Meanwhile, the intrinsic capability of this framework to uncover latent features highlights its significant potential value for identifying correlations and subtle physical trends across broader datasets.

2. Scope of method extension and applicability boundaries

We also acknowledge the reminder regarding broader transferability. The evidence presented in this study primarily supports two dimensions of extensibility. First, we

have validated the predictive accuracy and interpretability across diverse initial NiTi microstructures, strain amplitudes, cyclic stages, and variable-amplitude loading histories, including evaluations under boundary-transcending conditions (i.e., strain amplitudes and cycle counts outside the training distribution). These results confirm that the framework is reasonably adaptable to the NiTi-related degradation problem and to similar cyclic loading scenarios.

Second, the framework features a modular design that permits the seamless integration of new variables with distinct physical meanings without altering the core architecture. For instance, additional parameters such as grain size, temperature, and strain rate can be readily encoded through the numerical branch using our established approach. This indicates the potential of the framework for future expansion in terms of input variables, although it is not yet sufficient to demonstrate universal applicability across different material systems. **While the original manuscript did not explicitly claim universal applicability across different material systems, we recognize that certain phrasing might still lead to such an interpretation.** Consequently, we have further refined these statements to eliminate ambiguity.

Furthermore, we examined how framework-based studies typically describe the scope of method extension, and clarified the distinction between a proof-of-concept demonstration using a representative material system and proven generalizability across diverse material families. For example, a recent study (Lee et al., 2025, Nat. Commun. 16, 931) on the additive manufacturing of Ti-6Al-4V published focused its experimental validation on a single Ti-6Al-4V alloy system, while leaving broader system validation for future work. Similarly, another multi-objective active learning framework (Jablonka et al., 2021, Nat. Commun. 12, 2312) validated its method using a specific polymer system, cautiously framing its extension to other material systems as a direction for future validation rather than an already demonstrated result.

Based on these considerations, we have carefully revised statements related to Extensibility and generalization. The manuscript now emphasizes the modular design of the framework, its demonstrated adaptability within NiTi-related scenarios, and its

potential extension to other materials problems. We believe that this distinction preserves the value of the current study as a validation case, while avoiding overinterpretation beyond the available evidence regarding cross-system applicability.

3. Summary of revisions

In summary, the revised manuscript includes two principal sets of changes.

Regarding the positioning of conceptual novelty, the specific revisions are as follows:

Revision 1 (Page 2, Lines 4–6): In this study, an interpretable learning framework was proposed to predict the SED of NiTi alloys and uncover the degradation mechanisms using interpretability analysis methods.

Revision 2 (Page 4, Line 28 and Page 5, Lines 1–2): We visualize the competition between slip and SIMT across different strain amplitudes and cyclic stages, and further clarify the dual role of Ni_4Ti_3 precipitates in either promoting or suppressing SED.

Revision 3 (Page 6, Lines 12–15): When coupled with manifold learning, feature attribution methods, and molecular dynamics simulations, it elucidates the roles of grain orientation and precipitates in the degradation process.

Revision 4 (Page 12, Lines 17–22): Although the approach yields respectable accuracy in predicting SED, elucidating the underlying physical processes is still far from straightforward. To address this, we probed the governing factors by combining heatmap visualization and Shapley Additive Explanations (SHAP) analysis with molecular dynamics simulations. This study presents a spatially resolved and systematic analysis of how the dominant degradation mode evolves with strain amplitude under cyclic loading.

Revision 5 (Page 14, Lines 14–16): In systems where multiple mechanisms compete and coexist, this approach is particularly practical for tracking the transition of dominant modes during deformation.

Revision 6 (Page 15, Lines 4–7): e, i, m Grad-CAM heatmaps generated from the Euler matrix branch for the 2% strain amplitude after 1, 3 and 10 cycles, respectively,

where hotspots indicate orientation regions contributing most to degradation prediction.

Revision 7 (Page 15, Lines 9–13 and Page 16, Lines 1–2): The transition of dominant degradation modes under specific loading conditions is revealed: at high strain amplitude, hotspots strongly coincide with high Schmid factor regions for SIMT, indicating transformation-driven degradation; while at low strain amplitude, {011}⟨001⟩ and {011}⟨111⟩ dislocation slip dominates. Furthermore, (d) is derived from the first cycle at 6.38% strain amplitude, representing a transitional state between the two competing mechanisms.

Revision 8 (Page 22, Lines 21–28 and Page 23, Line 1): Against this backdrop, the interpretable ML framework demonstrates a quasi-in-situ mechanistic reconstruction by resolving and visualizing degradation processes, thereby offering an alternative path to full-field observations that are otherwise elusive through traditional experimental or theoretical routes. Unlike molecular dynamics simulations, which hinge on precise potentials and idealized initial configurations, this approach spontaneously extracts statistical patterns directly from real microstructures. By corroborating these learned patterns with independent experimental and simulation findings, this study narrows the existing observational gap and lays the foundation for future calibration of multiscale theoretical models.

Revision 9 (Page 23, Lines 12–16): Together, these results integrate previously qualitative and fragmented mechanistic insights into a more unified perspective: SED of NiTi emerges as a competition between mechanisms regulated by loading conditions, with the active pathway selected at the microscale by crystallographic geometric compatibility.

Revision 10 (Page 24, Lines 19–21): Notably, the significance of this interpretable framework extends beyond merely elucidating degradation mechanisms; its architecture also embodies a clear physical hierarchy.

Regarding generalizability and applicability boundaries, the specific revisions are as follows:

Revision 11 (Page 5, Lines 2–3): Finally, a model is extensible if it can be effectively adapted to related scenarios (Fig. 1h).

Revision 12 (Page 6, Lines 15–18): Extensibility validation encompasses strain amplitude and cycle number extrapolations, alongside generalization assessments under diverse loading conditions (e.g., strain rate and temperature), further demonstrating the practical relevance of the framework.

Revision 13 (Page 20, Lines 25–26): This demonstrates the transferability of our framework under complex loading histories.

Revision 14 (Page 21, Lines 5–6): The structural descriptor stabilizes model performance under limited data.

Revision 15 (Page 21, Lines 10–11): Fig. 7 | Extensibility and robustness validation of the multimodal framework. a, b Prediction errors under boundary-transcending conditions.

Revision 16 (Page 22, Lines 4–6): Collectively, the approach demonstrates robust extensibility, and embedding physically meaningful structural variables effectively mitigates performance fluctuations.

Revision 17 (Page 24, Lines 16–18): Overall, transforming mechanism maps into design criteria signals a shift in NiTi design from trial-and-error toward refined guidance grounded in verifiable principles.

Revision 18 (Page 25, Lines 9–11): Consequently, even with limited experimental data and cost, it holds great promise for evaluating fatigue damage and functional degradation over thousands to tens of thousands of loading cycles.

In addition, there is one revision that is related to both:

Revision 19 (Page 5, Lines 3–5): Our study delineates a promising avenue for AI-driven assessment and interpretation of functional stability in SMAs, and orients future explorations in this emerging direction of materials research.

Once again, we sincerely thank you for helping us more clearly distinguish among the discovery of new mechanisms, the quantitative refinement of existing mechanistic

understanding, and the extension of framework capabilities across different material systems. We believe that these revisions present the methodological contributions, evidentiary boundaries, and scope of applicability of this study in a more objective, cautious, and precise manner.

Reviewer 2: The authors have addressed all my comments in the revised manuscript. Thus, I recommend this paper to be published in its current form.

2.0 Response: Thank you for your time and constructive feedback on our manuscript. We sincerely appreciate your confirmation that all concerns have been addressed and are grateful for your support throughout the review process.
